# Genome-wide analyses of late pollen-preferred genes conserved in various rice cultivars and functional identification of a gene involved in the key processes of late pollen development

**DOI:** 10.1186/s12284-018-0219-0

**Published:** 2018-04-23

**Authors:** Sunok Moon, Moe Moe Oo, Backki Kim, Hee-Jong Koh, Sung Aeong Oh, Gihwan Yi, Gynheung An, Soon Ki Park, Ki-Hong Jung

**Affiliations:** 1 Graduate School of Biotechnology and Crop Biotech Institute, Kyung Hee University, Yongin, 446-701 South Korea; 2 School of Applied Biosciences, Kyungpook National University, Daegu, 702-701 South Korea; 3 Department of Plant Science, Research Institute of Agriculture and Life Sciences, and Plant Genomics and Breeding Institute, Seoul National University, Seoul, 151-921 South Korea; 4 College of Agriculture and Life Science, Daegu, 702-701 South Korea

**Keywords:** GUS assay, Pollen maturation process, Pollen germination process, Rice, T-DNA insertional mutant, CRISPR/Cas9, Network analysis

## Abstract

**Background:**

Understanding late pollen development, including the maturation and pollination process, is a key component in maintaining crop yields. Transcriptome data obtained through microarray or RNA-seq technologies can provide useful insight into those developmental processes. Six series of microarray data from a public transcriptome database, the Gene Expression Omnibus of the National Center for Biotechnology Information, are related to anther and pollen development.

**Results:**

We performed a systematic and functional study across the rice genome of genes that are preferentially expressed in the late stages of pollen development, including maturation and germination. By comparing the transcriptomes of sporophytes and male gametes over time, we identified 627 late pollen-preferred genes that are conserved among japonica and indica rice cultivars. Functional classification analysis with a MapMan tool kit revealed a significant association between cell wall organization/metabolism and mature pollen grains. Comparative analysis of rice and *Arabidopsis* demonstrated that genes involved in cell wall modifications and the metabolism of major carbohydrates are unique to rice. We used the *GUS* reporter system to monitor the expression of eight of those genes. In addition, we evaluated the significance of our candidate genes, using T-DNA insertional mutant population and the CRISPR/Cas9 system. Mutants from T-DNA insertion and CRISPR/Cas9 systems of a rice gene encoding glycerophosphoryl diester phosphodiesterase are defective in their male gamete transfer.

**Conclusion:**

Through the global analyses of the late pollen-preferred genes from rice, we found several biological features of these genes. First, biological process related to cell wall organization and modification is over-represented in these genes to support rapid tube growth. Second, comparative analysis of late pollen preferred genes between rice and *Arabidopsis* provide a significant insight on the evolutional disparateness in cell wall biogenesis and storage reserves of pollen. In addition, these candidates might be useful targets for future examinations of late pollen development, and will be a valuable resource for accelerating the understanding of molecular mechanisms for pollen maturation and germination processes in rice.

**Electronic supplementary material:**

The online version of this article (10.1186/s12284-018-0219-0) contains supplementary material, which is available to authorized users.

## Background

The life cycles of all land plants alternate between diploid sporophyte and haploid gametophyte. By the time gametophyte development is finished, a floret of rice contains one carpel with a single egg cell, plus six stamens, each filled with approximately 1000 pollen grains (Prasad et al. 2006; Zhang et al. 2011). Pollen development covers several steps, from stamen meristem specification to pollen grain formation and pollination, and various cell and tissue types are involved. Not only dynamic changes in gene expression but also post-translational control of proteins are important for those processes (Guan et al. 2014). Development of the gametes is modulated by gametophytic and sporophytic gene expression. In the case of the pollen wall, the tapetum contributes to exine and tryphine formation while the gametophytes are responsible for intine formation (Shi et al. 2015). The tapetum, the innermost anther wall layer, serves as a nutritive tissue that secretes pollen wall components, nutrients, and metabolites (Xu et al. 2014). Many tapetum-expressed genes have been shown to regulate anther and pollen wall development (Xu et al. 2014; Shi et al. 2015). In addition, plant hormones such as auxin and gibberellin (GA) control pollen development in sporophytic tissues (Sakata et al. 2014; Cecchetti et al. 2017).

The male gametophyte originates from a pollen mother cell that undergoes meiosis to produce tetrads of haploid microspores (Owen and Makaroff 1995). After their release from those tetrads, the microspores enlarge and then undergo asymmetric mitosis to form bi-cellular pollen with different cell fates (pollen mitosis I). Afterward, the smaller generative cell divides symmetrically to produce two sperm cells (pollen mitosis II). Tri-cellular mature pollen is further processed to complete its development and is then ready to germinate (Bedinger 1992).

High-throughput microarray data provide a powerful tool for identifying genes at the genome scale that control pollen development. Genome-wide investigations have already been performed with the male gametophytic transcriptomes in *Arabidopsis* and rice (Becker et al. 2003; Honys and Twell 2003; Honys and Twell 2004; Suwabe et al. 2008; Fujita et al. 2010; Wei et al. 2010; Aya et al. 2011). Those studies have revealed that mature pollen grains have a unique transcriptome profile with a higher proportion of selectively expressed genes than in any other tissues (Becker et al. 2003; Wei et al. 2010). This unique transcriptome represents the biological processes required for pollen development, i.e., cell wall metabolism, signalling, and cytoskeleton dynamics. Honys and Twell (2004) and Wei et al. (2010) have reported dynamic changes in the transcriptomes of *Arabidopsis* and rice, based on their sequential stages of pollen development. Patterns of changes in their transcripts are conserved between those two species. The diversity of these transcripts is greatly decreased as the pollen progresses from uni-cellular microspores to mature grains (Wei et al. 2010). The transition from bi-cellular to tri-cellular pollen was also observed by an increase in the proportion of male gametophyte-specific transcripts (Wei et al. 2010). Competition among pollen grains for an egg cell to produce a diploid zygote is a common phenomenon. Because rice pollen begins to germinate within 2 min after pollination (Chen et al. 2008), most of the required components for this are already present in the mature grains. Analysis by Wei et al. (2010) has revealed that the gene expression profiles of mature grains and germinated grains are significantly and positively correlated (*r* = 0.99), suggesting that the former stores a set of transcripts for germination. Moreover, inhibition of RNA synthesis by actinomycin D does not interrupt germination, indicating that the RNAs necessary for germination are already present in mature grains (Mascarenhas 1993; Hao et al. 2005).

Success during the late stages of pollen development is critical if crop yields are to be sustained. Applying microarray technologies with in rice, a model system, several research groups have conducted genome-wide transcriptome analyses and identified genes that are preferentially expressed in the pollen (Fujita et al. 2010; Deveshwar et al. 2011; Russell et al. 2012). Although different numbers of genes -- 453 (Fujita et al., 2010), 735 (Deveshwar et al., 2011), and 1376 (Russell et al., 2012) – have been uncovered in those independent studies, the consistency of expression patterns has not been assayed over a range of growing environments and genetic backgrounds. Using *Arabidopsis*, Suzuki et al. (2008) and Dobritsa et al. (2011) have performed large-scale screening of pollen exine mutants from randomly selected lines and have identified 12 and 14 genes involved in forming the pollen exine. Although numerous gene expression data and genome-wide gene-indexed mutant populations are available for rice, no previous attempts have been described to understand the roles of genes in controlling late pollen development on a large scale. Thus, such systematic approaches might help accelerate our identification of rice genes that function in mature pollen development and germination.

A shift in gene expression exists during pollen development and bi-cellular pollen may be a key point for the regulation of shift (Wei et al. 2010). “Late pollen-preferred genes” was defined as preferentially expressed genes at pollen following the bi-cellular stage. In this study, we identified late pollen-preferred genes conserved in japonica and indica cultivars of rice. Here we provide detailed results from our genome-wide analysis of such genes conserved in two subspecies of rice. We also discuss mutants obtained through T-DNA and gene-editing that show defects in gene-transfer through the male gamete.

## Results

### Meta-expression analysis and genome-wide identification of late pollen-preferred genes conserved in various rice cultivars

**Fig. 1:**
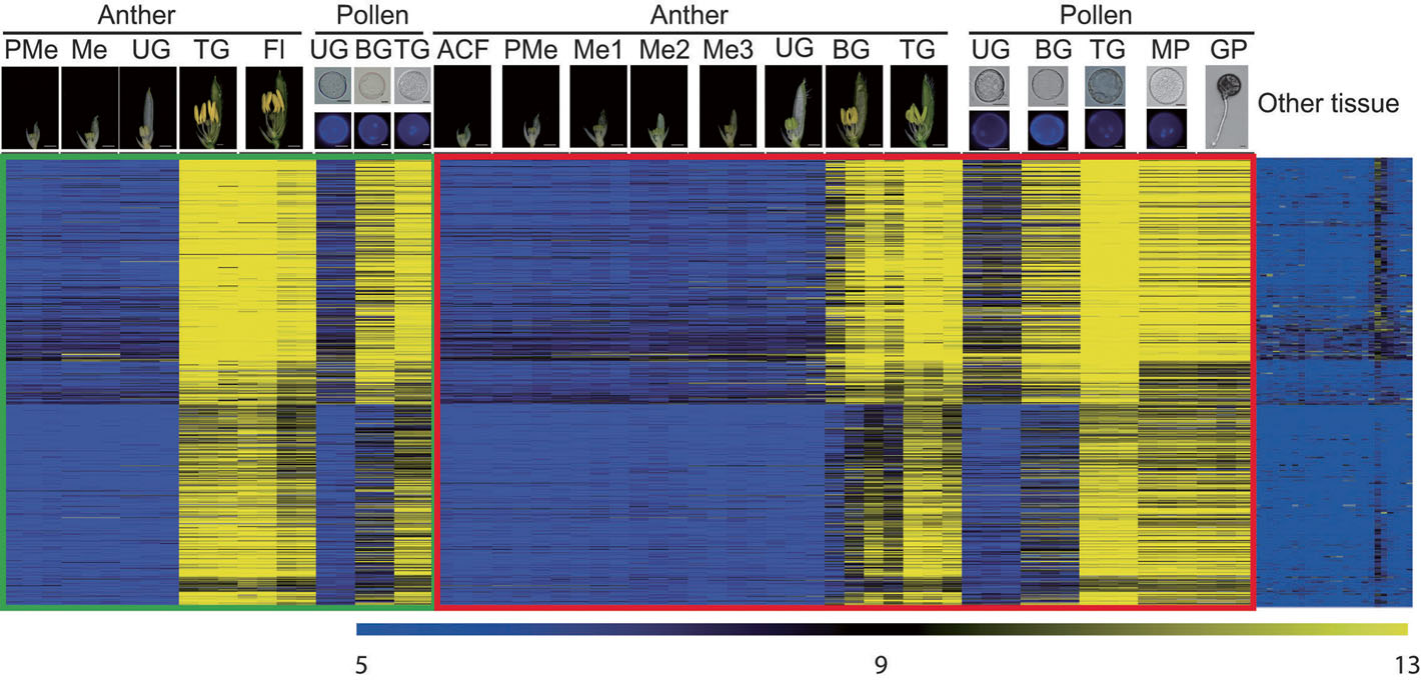
Heatmap expression analysis of 627 late pollen-preferred genes. Expression patterns conserved among indica (green box) and japonica (red box) rice types are shown over several stages of development. Microarray data numbered 22 for indica rice (5 stages for anthers and 3 for pollen) and 42 for japonica rice (8 stages for anthers and 5 for pollen). Anatomical meta-expression data from ROAD were used to check expression patterns in other tissues. ACF, archesporial cell-forming stage; BG, bi-cellular gametophyte stage; Fl, flowering stage; GP, germinating pollen; Me, meiotic stage; Me1, meiotic leptotene stage; Me2, meiotic zygotene-pachytene stage; Me3, meiotic diplotene-tetrad stage; MP, mature pollen stage; PMe, pre-meiosis; TG, tri-cellular pollen stage; UG, uni-cellular gametophyte stage. Yellow color in heatmap indicates high level of expression; dark-blue, low expression

To identify the late pollen-preferred genes that are conserved among japonica and indica cultivars of rice, we examined publicly available Affymetrix rice microarray data. From the National Center for Biotechnology Information Gene Expression Omnibus (NCBI GEO; http://www.ncbi.nlm.nih.gov/geo/), we downloaded and analyzed six series of data prepared from developing anthers and pollen grains (Additional file 1: Table S1) (Edgar et al. 2002). The samples were arranged in order according to the process of anther and pollen development in each cultivar. Intensity values for these six series were first normalized with the Affy package in the R program and then log_2_-transformed. As a control, all of the Affymetrix anatomical meta-expression profiles in the Rice Oligonucleotide Array Database (ROAD;), except for two anther samples, were used to check expression patterns in other tissues/organs (Cao et al. 2012). Afterward, we performed *K*-means clustering (KMC) analysis with the Euclidean Distance Metric and grouped 57,382 probes into 36 clusters based on their expression patterns. Clusters 2 and 35 exhibited the highest expression at the later stages, after bi-cellular pollen (Additional file 2: Figure S1). As a result, we found that 627 genes, represented by 750 probe sets in the rice affymetrix array, showed late pollen-preferred expression patterns in both subspecies (Fig. 1). These gene clusters are listed in Table S2 (Additional file 1).

### Expression patterns for late pollen-preferred genes confirmed through the *GUS* reporter system

**Fig. 2:**
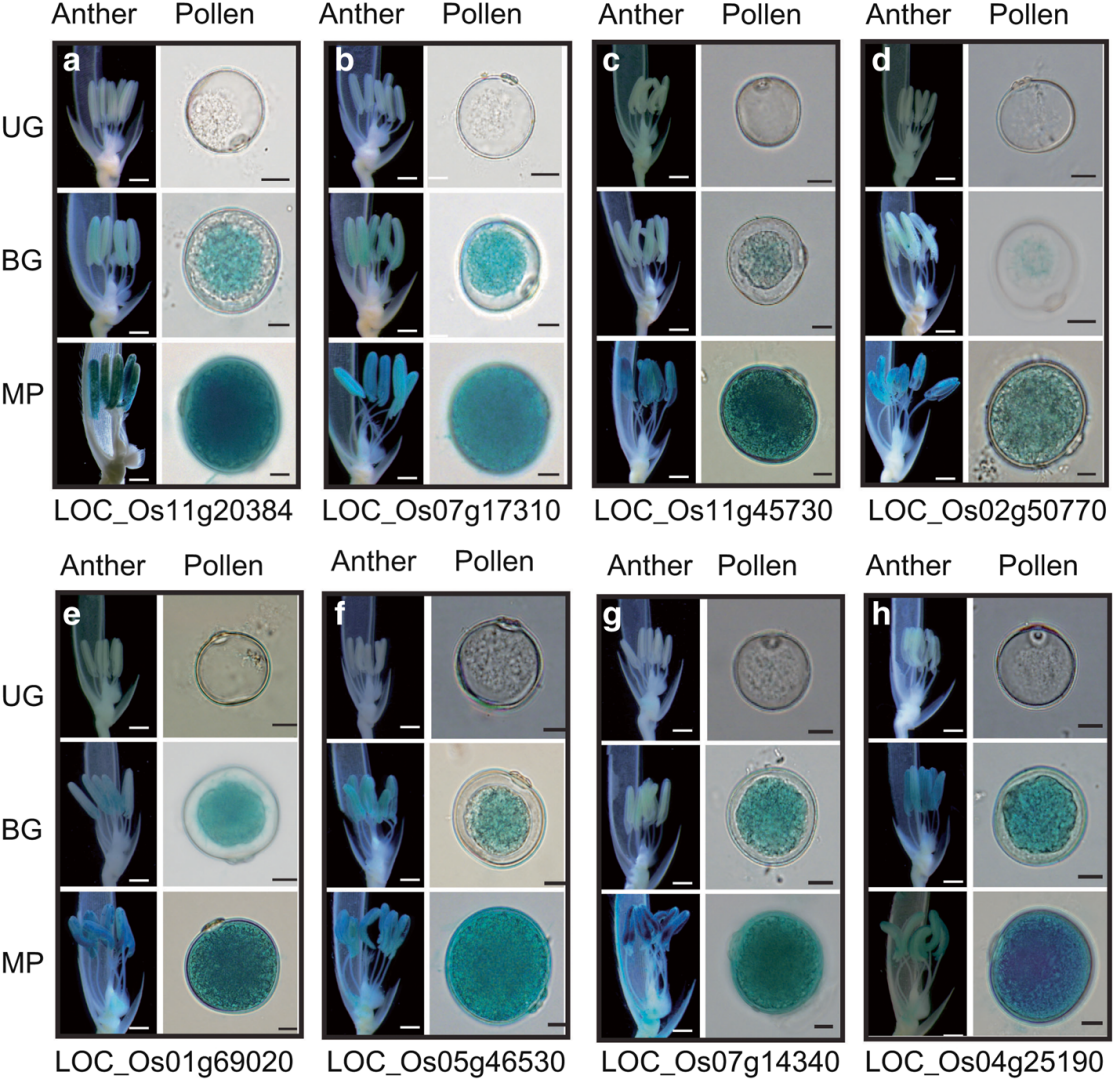
Evaluation of late pollen-preferred genes using *GUS* reporter systems. Images of flower and pollen from promoter trap lines 1A-13,819 having T-DNA insertion within *LOC_Os11g20384* (**a**) and 2D-41,188 harboring T-DNA insertion within *LOC_Os07g17310* (**b**) at sequential stages of pollen development. Images of flower and pollen from transgenic plants harboring promoter::*GUS* reporter constructs. Promoters from *LOC_Os11g45730* (**c**), *LOC_Os02g50770* (**d**), *LOC_Os01g69020* (**e**), *LOC_Os05g46530* (**f**), *LOC_Os07g14340* (**g**), and *LOC_Os04g25190* (**h**) were used to control *GUS* expression. BG, bi-cellular gametophyte stage; MP, mature pollen stage; UG, uni-cellular gametophyte stage. *GUS* expression patterns were used from promoter trap lines and promoter-*GUS* transgenic lines. Bar in flower image = 0.5 mm; bar in pollen image = 10 μm

**Fig. 3:**
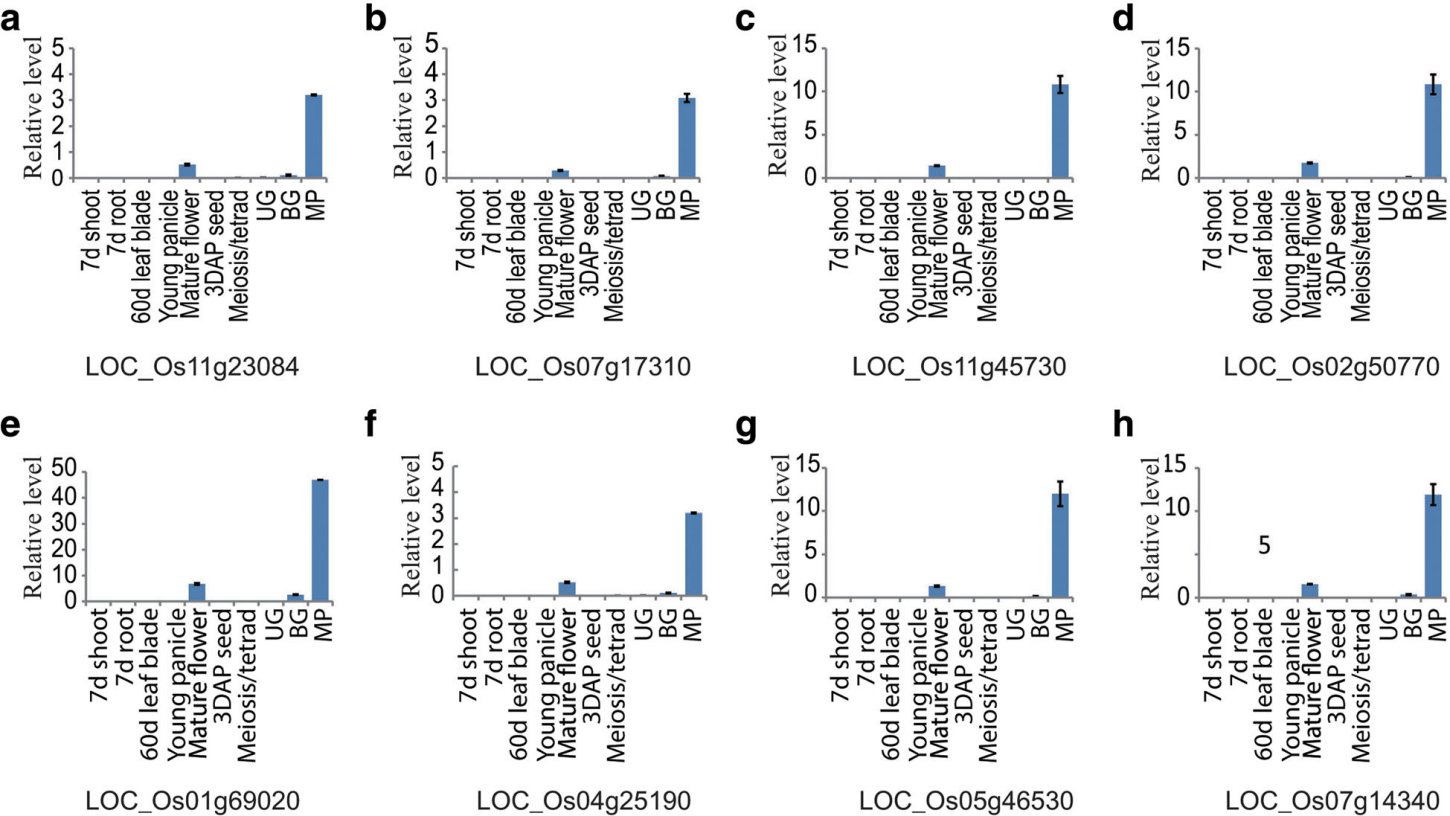
Real-time RT-PCR analysis of 8 genes shown in Fig. 2. Expression patterns of *LOC_Os11g20384* (**a**), *LOC_Os07g17310* (**b**), *LOC_Os11g45730* (**c**), *LOC_Os02g50770* (**d**), *LOC_Os01g69020* (**e**), *LOC_Os05g46530* (**f**), *LOC_Os07g14340* (**g**), and *LOC_Os04g25190* (**h**) were analyzed by quantitative real-time PCR for various organs. Transcript levels were normalized to rice *Ubi5* and calculated by comparative cycle threshold method. Error bars indicate standard deviation (sd). y-axis, relative expression level to rice *Ubi5*; x-axis, sample names used for analyses. 3DAPS, seeds at 3 d after pollination; 7DS, 7-day-old shoots; 7DR, 7-day-old roots; ML, mature leaves; MF, mature flower; YP, young panicles (2 cm long); and anthers at bi-cellular gametophyte stage (BG); tri-cellular mature pollen stage (MP); meiosis and tetrad (M/T); and uni-cellular gametophyte stage (UG)

To verify the patterns of late pollen-preferred expression in our candidate genes, we used two strategies: a promoter trap system and generation of transgenic plants under the control of selected promoters. We have previously described this promoter trap technique using T-DNA that carries the promoterless *GUS* reporter gene in japonica rice (Jeon et al. 2000). In all, 22 lines were chosen that had an in-frame fusion of the promoterless *GUS* in the genic region. Expression of that reporter gene was detected at three developmental stages. Subsequently, we identified two lines showing late pollen-preferred GUS-staining patterns (Fig. 2a and b), as had been expected from our meta-expression analysis depicted in Fig. 1. The efficiency of *GUS* expression was 9.09% (2/22), which was approximately 16-fold higher than the 0.58% we had previously found (Jeong et al. 2002). This was because, for our current investigation, we chose lines with T-DNA insertions within the late pollen-preferred genes whereas earlier studies used randomly selected samples (Jeong et al. 2002). The T-DNAs were inserted into LOC_Os11g20384, encoding SacI homology domain-containing protein (Line 1A-13,819), and into LOC_Os07g17310, encoding B12D protein (Line 2D-41,188). Schematic diagrams of those T-DNA insertions are presented in Figure S2 (Additional file 2). In a separate examination, we used transgenic plants harboring promoter and *GUS* fusion constructs. Six genes in Clusters 2 and 35 were chosen that exhibited expression values above 13 log_2_ in mature pollen; their locus numbers and promoter regions are shown in Table S3 (Additional file 1). Their promoter activities had already been tested in *Arabidopsis* (Oo et al. 2014). *LOC_Os11g45730* encodes pectinesterase; *LOC_Os02g50770*, plant peroxidase family protein; *LOC_Os01g69020*, cell division protein FtsZ family protein; *LOC_Os05g46530,* plant invertase/pectin methylesterase (PME) inhibitor domain-containing protein; *LOC_Os07g14340*, pectinesterase inhibitor domain-containing protein; and *LOC_Os04g25190,* pollen allergen Lol p2 family protein. The GUS expression patterns within transgenic lines for these genes were monitored in rice at three sequential stages of pollen development (Fig. 2c-g). Strong GUS activity was detected at the mature pollen stage, as indicated in the heatmap. The pattern of late pollen-preferred expression by these genes, as shown in Fig. 2, was reconfirmed by real-time PCR analysis. For all eight genes, strong expression was detected at the tri-cellular mature pollen stage (Fig. 3). All of our results demonstrated that the meta-expression data for these genes are a valuable source of novel promoters for controlling traits associated with late pollen development or germination and for discovering the novel functions of such genes.

### Biological processes specific to late pollen-preferred genes

We conducted a Gene Ontology (GO) enrichment analysis in ROAD (Jung et al. 2008) to query 627 late pollen-preferred genes belonging to Clusters 2 and 35. Our objective was to examine GO enrichment within the category of ‘biological process’ with the ROAD GO enrichment tool. As a result, we identified 547 GO terms assigned to 308 genes; the other 319 genes did not have GO annotations (Additional file 1: Table S4). Significant terms in that category were selected with hypergeometric *p*-values ≤0.05 and enrichment values of at least two-fold. Of these, 16 GO terms were over-represented in late pollen-preferred genes (Table 1). Significantly enriched terms were found for biological processes corresponding to phosphatidylcholine metabolism (22.3 GO fold-enrichment value), clathrin coat assembly (19.7), GA metabolism (18.1), cell wall modifications (17.3), phosphatidyinositol metabolism (12.1), glycogen biosynthesis (10.9), starch biosynthesis (9.7), polysaccharide catabolism (8.5), sexual reproduction (7.4), cytoskeleton organization (7.0), cellulose biosynthesis (6.4), potassium ion transport (3.2), and ATP biosynthesis (2.9). The most abundant terms were for ‘protein amino acid phosphorylation’, representing 56 genes. This was followed by 34 genes for transport and 23 for carbohydrate metabolism.

**Table 1:** Analysis of significantly enriched Gene Ontology terms for genes preferentially expressed in late from rice

GO category	No. of GO repeats^a^	No. of GO repeats in queried genes^b^	No. of expected GO repeats^c^	Fold-enrichment value^d^
Phosphatidylcholine metabolic process	13	4	0.2	22.3
Clathrin coat assembly	11	3	0.2	19.7
Gibberellin metabolic process	20	5	0.3	18.1
Cell wall modification	46	11	0.6	17.3
Phosphatidylinositol metabolic process	18	3	0.3	12.1
Glycogen biosynthetic process	20	3	0.3	10.9
Starch biosynthetic process	15	2	0.2	9.7
Polysaccharide catabolic process	34	4	0.5	8.5
Sexual reproduction	39	4	0.5	7.4
Cytoskeleton organization	31	3	0.4	7.0
Cellulose biosynthetic process	34	3	0.5	6.4
Potassium ion transport	203	9	2.8	3.2
ATP biosynthetic process	99	4	1.4	2.9
Carbohydrate metabolic process	600	23	8.3	2.8
Protein amino acid phosphorylation	1593	56	22.0	2.5
Transport	978	34	13.5	2.5

Total number of GO terms in rice genome is 39,571; total number of GO terms in queried pollen-preferred genes, 547

^a^number of selected GO Slim terms annotated in rice genome

^b^observed number of selected GO Slim terms in queried pollen-preferred genes

^c^expected number of selected GO Slim terms in queried pollen-preferred genes

^d^relative ratio of observed number to expected number for a selected GO Slim term

### Late pollen-preferred genes functionally classified through MapMan analysis

**Fig. 4:**
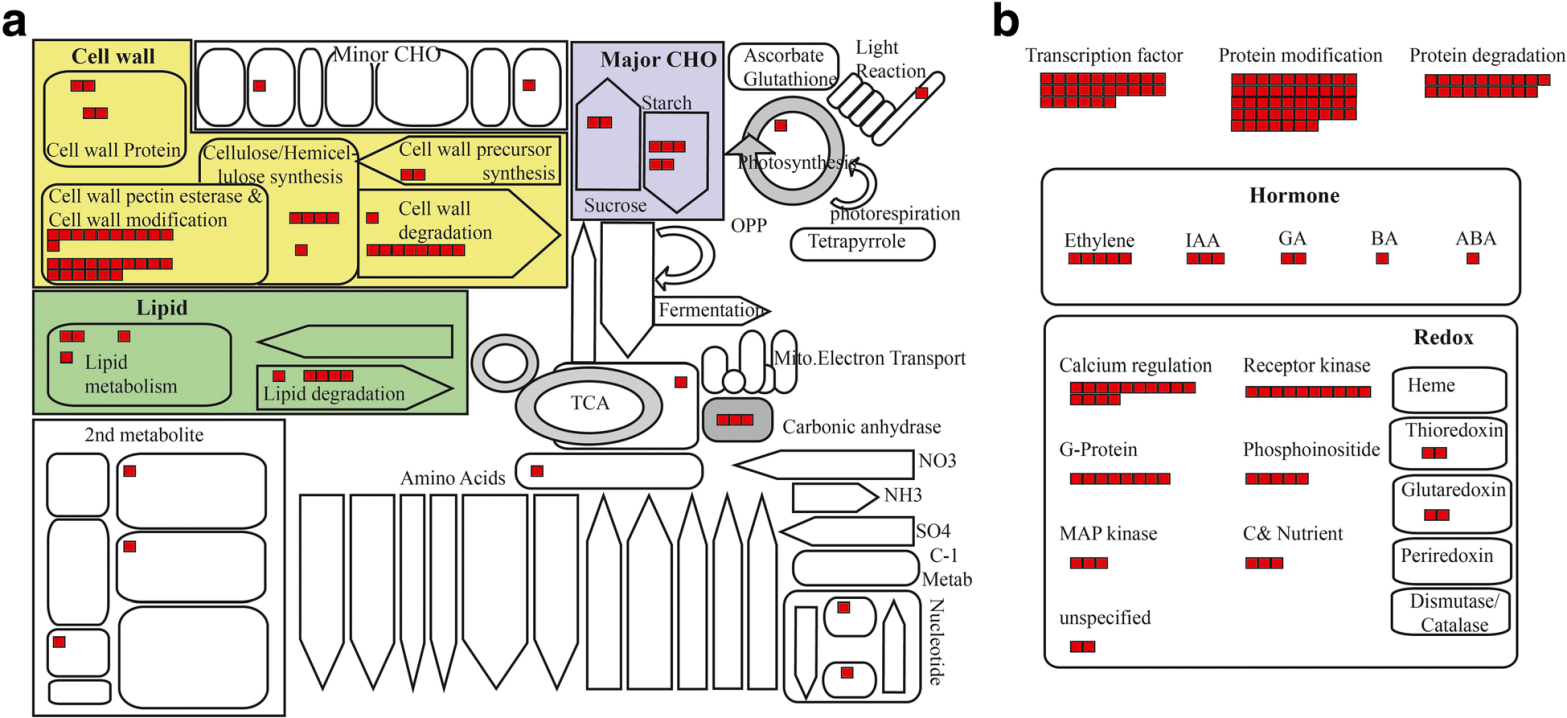
MapMan analysis of late pollen-preferred genes. **a** Metabolic overview and (**b**) regulation overview were analyzed with 627 genes. Cell wall organization and modification, lipid metabolism, and carbohydrate metabolism were dominant MapMan terms in metabolic overview. Red boxes in these overviews indicate late pollen-preferential. Genes associated with transcription factors, protein modification, and protein degradation were identified from regulation overview. In addition, diverse hormone responses, signaling pathway components, and redox responses for late pollen development were visualized. Detailed information about overviews is shown in Table S5.

MapMan allows one to group genes into different functional categories and visualize data through diagrams (Yoo et al. 2015). To classify these late pollen-preferred genes, we primarily used the metabolism and regulation overviews installed in the MapMan tool kit (Fig. 4a). In the metabolism overview, cell wall organization and modification (48 genes), lipid metabolism (10), and metabolism of major carbohydrates (9) were the most common (Additional file 1: Table S5). The predominance of the cell wall organization and modification terms was well-matched with our finding that GO terms related to those processes were enriched in the biological process category (Table 1). In particular, cell wall-modifying enzymes such as pectin methylesterases (PMEs) and cellulose synthases were functionally well-characterized during pollen tube growth because the cell walls of those tubes consist of callose in the inner layer plus pectin, cellulose, and hemicellulose in the outer layer (Krichevsky et al. 2007). Our list included 11 PMEs and 4 cellulose synthases, implying that those genes are involved in pollen maturation and germination in rice.

Our regulation overview showed that genes associated with transcriptional regulation, protein modification, and protein degradation were the most frequently coupled with late pollen-preferred genes (Additional file 1: Table S5). Of them, 26 genes were assigned to the transcription category and 47 genes were related to protein modification (Fig. 4b; Additional file 1: Table S5). For protein degradation, 19 genes were found. We also found 12 genes in the category of hormone metabolism – five for ethylene synthesis, three for auxin, and two for GA (Additional file 1: Table S6).

### Comparative analysis of late pollen-preferred genes from rice and *Arabidopsis*

Late pollen-preferred genes in *Arabidopsis* were identified using a meta-anatomical expression database that we developed from the *Arabidopsis* Affymetrix array data deposited in the NCBI GEO database (See Materials and Methods). This produced 773 genes (Additional file 2: Figures S3 and S4; Additional file 1: Table S7). We then searched for orthologs between rice and *Arabidopsis* with a database of orthologous groups among rice, *Arabidopsis*, *Brachypodium*, maize (*Zea mays*), *Populus*, *Vitis vinifera*, and *Sorghum bicolor* from the Rice Genome Annotated Project (RGAP; http://rice.plantbiology.msu.edu/annotation_pseudo_ortho.shtml). Rice orthologs for 380 *Arabidopsis* genes were identified based on the sequence information. Among them, orthologs for 175 *Arabidopsis* genes exhibited patterns of late pollen-preferential expression (Additional file 1: Table S8). Another search based on 627 rice late pollen-preferred genes led to the identification of 220 *Arabidopsis* orthologs (Additional file 1: Table S9). Of them, 133 had *Arabidopsis* orthologs with late pollen-preferential expression. Therefore, all of these results indicated that functional conservancy was approximately 20%, with 175 (22.64%) of 773 *Arabidopsis* having rice orthologs and 133 (21.21%) of 627 rice genes having *Arabidopsis* orthologs. We believe that genes unique to each species, i.e., 407 in rice and 393 in *Arabidopsis*, will be good targets for uncovering the evolutionary developmental differences in mature and germinating pollens between those two plant systems.

### Enriched biological processes in late pollen-preferred genes compared between rice and *Arabidopsis*

**Fig. 5:**
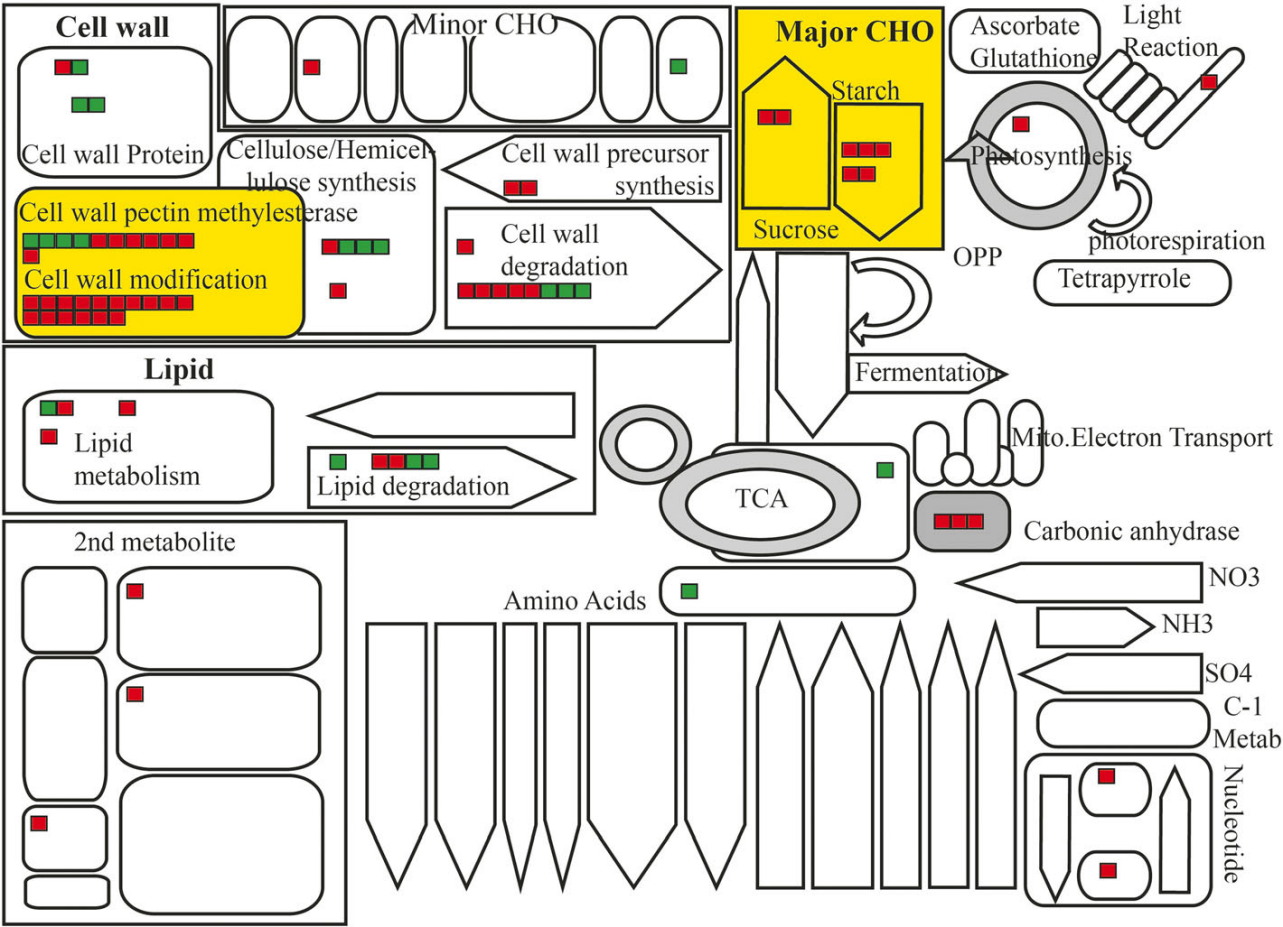
Enriched metabolic pathways in rice late pollen-preferred genes compared to those of *Arabidopsis*. Red squares, genes unique to rice; green squares, conserved genes in terms of their sequence homology and expression patterns between 2 plant systems

Through our comparative genome and expression analyses, we determined that approximately 20% of late pollen-preferred genes are conserved between rice and *Arabidopsis* in terms of their sequence homology and expression patterns. We performed MapMan analysis for rice divergent genes without ortholog(s) in *Arabidopsis* as well as common genes conserved between the two. Several MapMan terms did not overlap between the divergent and common genes. Moreover, MapMan terms for cell wall modification and major carbohydrate metabolism were found only in the rice divergent genes (Fig. 5).

### Genetic study of T-DNA insertional mutants for a male-gene transfer defective (*MTD1*) gene involved in late pollen development

**Fig. 6:**
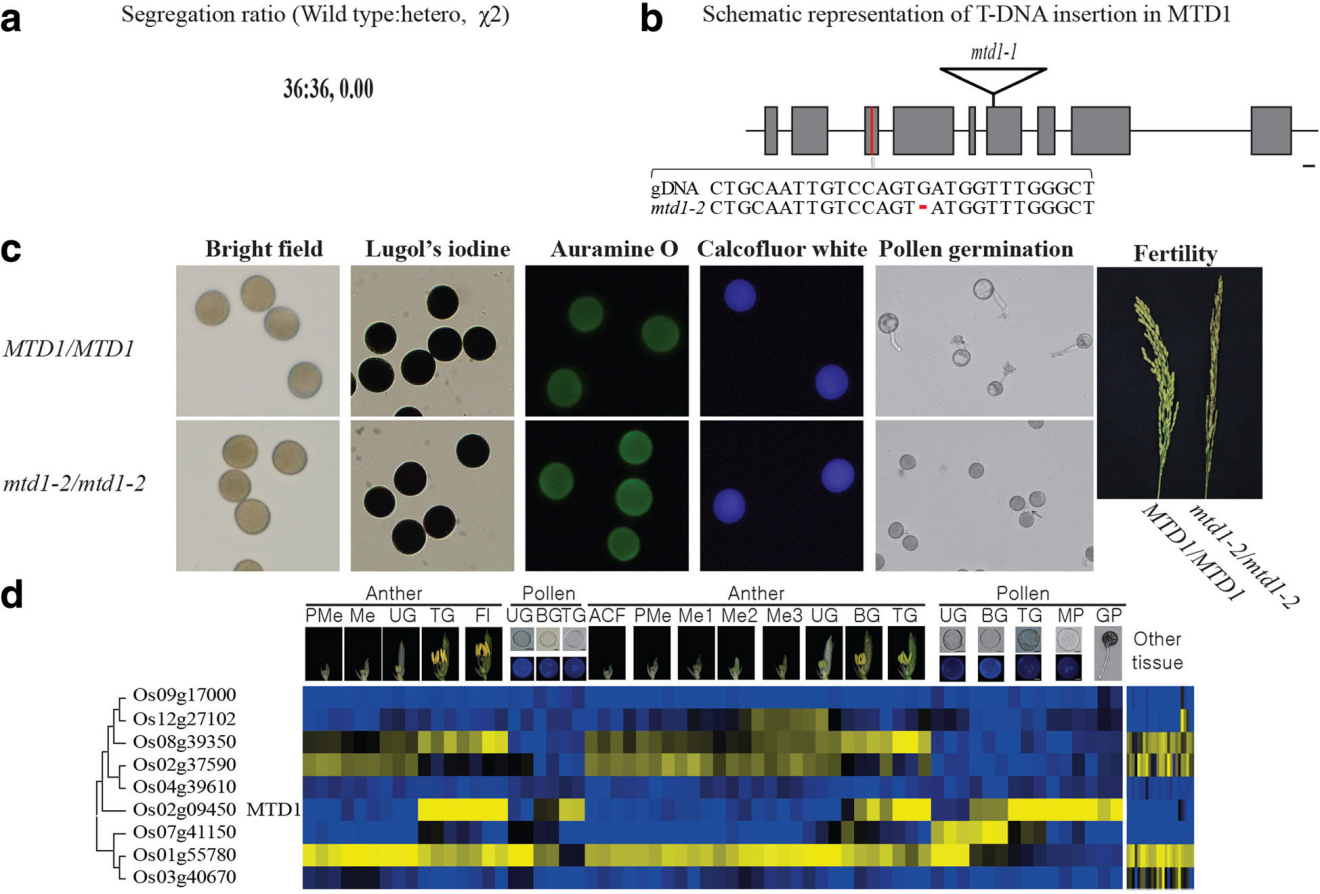
Estimation of functional dominancy by MTD genes through phylogenomic analysis (**a**) segregation ratios of heterozygotes and wild-type segregants in mutants of *MTD1* by T-DNA insertion, and results of Chi-square test (χ^2^). **b** Schematic view of *MTD1* structure and mutated position by T-DNA insertions and deletion generated by CRISPR/Cas9. Grey boxes indicate exon; lines, UTR or intron; reverse triangles, T-DNA; red box, target region of CRISPR/Cas9. Genomic DNA sequences from wild type and *gtd1–2* are shown. **c** Phenotypes of pollen grains from wild type and *mtd1–2*. Germinated pollen and grains stained with Lugol’s iodine, auramine O, or calcofluor white were observed under bright field and fluorescence microscopes. Bar = 20 μm. Panicles at filling stage of wild type and *mtd1–2* were observed. **d** Expression patterns according to developmental stages of pollen were analyzed using 22 microarray data from indica rice (5 stages for anthers, 3 for pollen) and 42 microarray data from japonica rice (8 stages for anthers, 5 for pollen). Anatomical meta-expression data from ROAD were used to check expression patterns in other tissues. ACF, archesporial cell-forming stage; BG, bi-cellular gametophyte stage; Fl, flowering stage; GP, germinating pollen; Me, meiotic stage; Me1, meiotic leptotene stage; Me2, meiotic zygotene-pachytene stage; Me3, meiotic diplotene-tetrad stage; MP, mature pollen stage; PMe, pre-meiosis; TG, tri-cellular pollen stage; UG, uni-cellular gametophyte stage. Yellow color in heatmap indicates high level of expression; dark-blue, low expression

To identify the functional significance of our candidates, we analyzed T-DNA insertion lines for 28 genes (An et al. 2003; Ryu et al. 2004; Jeong et al. 2006). Until now, a mutant line was identified because of distortions in segregation ratios, which were close to 1:1:0 (wild type:heterozygote:homozygote) (Fig. 6a). The gene that causes those distortions in corresponding mutants was named *Male*-*gene Transfer Defective* (*MTD*)*.* We then called the first gene *MTD1. MTD1/LOC_Os02g09450* encodes glycerophosphoryl diester phosphodiesterase (GPD), with 767 amino acids. It has nine exons and eight introns (Fig. 6b). We also identified a T-DNA insertional mutant of this gene (*mtd1–1*), which has a T-DNA insertion in the sixth exon (Fig. 6b)*.*

To clarify how *MTD1* participates in gene-transfer through the male gametophyte, we performed reciprocal crosses between heterozygotes and the wild type (WT). From this, we determined that *mtd1–1* mutants have a defect in gene-transfer through the male gametophyte (Table 2). That is, a cross between the male (pollen) from an *MTD1*/*mtd1–1* heterozygous plant and the female (ovary) of the WT shows a complete defect (0/35) in gene-transfer through the male gametophyte. In contrast, a cross between the female from an *MTD1*/*mtd1* heterozygous plant and a WT male leads to successful gene-transfer through the female gametophyte. To examine any morphological defects, we observed mature grains under a bright field microscope but did not found any differences between WT and *mtd1–1* mutant pollen.

**Table 2:** Genetic transmission analysis of *MTD1*/*mtd1* by reciprocal crossing

Genotype (female x male)	Wild type	Hetero	TE
*MTD1/mtd1–1* X WT	33	28	84.8
WT X *MTD1/mtd1–1*	35	0	0.0

Segregation ratio was analyzed in F1 progeny of represented crosses. The transmission efficiency (TE) represents the percentage of *mtd1–1* mutant allele successfully transmitted through male or female gametes

To confirm the phenotype of *mtd1* and understand the function of *MTD1*, we utilized the CRISPR/Cas9 system, selecting the third exon as a target region of the guide RNA (Fig. 6b). From sequence analysis of the primary transgenic lines, we obtained a homozygote with a 1-bp deletion in the *MTD1* coding sequence. That deletion mutant was named *mtd1–2.* Because we already knew that *mtd1–1* has a defect in gene-transfer through the male gametophyte, we checked the mature pollen status of *mtd1–*2 by staining with auramine O, calcofluor white, and Lugol’s iodine (Moon et al. 2013). No differences between WT and mutant pollen were observed with regard to the accumulation of exine (stained by auramine O), intine (calcofluor white), or starch (Lugol’s iodine) (Fig. 6c). This meant that *MTD1* has no essential role in the production of mature pollen. Therefore, we speculated that this gene might function at the progamic phase, i.e., during pollen germination, pollen tube elongation, and commutation with female organs. We then tested in vitro pollen germination of *mtd1–2*/*mtd1–2*. Whereas the WT pollen had elongated tubes, the *mtd1–2* pollen exhibited only a small protrusion of those tubes (Fig. 6c). Because of this defect, homozygotic plants of *mtd1–2* were sterile.

We also found that there are 13 genes encoding GPD in rice, and nine of them are clustered more closely with MTD1 (Fig. 6d). Of these, *MTD1* was predominantly expressed in late pollen stage, which supported our supposition that it has a primary role in this developmental process, as revealed in the loss-of-function mutants (Fig. 6d).

## Discussion

### In silico validation of late pollen-preferred genes in various rice cultivars

We delved into the large collection of microarray data focused on anther and pollen development in rice. Having identified candidate genes with late pollen-preferred expression that are conserved among japonica and indica rice enabled us to investigate relevant developmental processes across a more diverse range of varieties than would be possible from examinations with only single variety. We have recently reported that at least 10% of genes showing light-responsive expression patterns differ among japonica (‘Nipponbare’, ‘TP309’, and ‘LG’) and indica (‘IR24’) cultivars (Jung et al. 2008). By analyzing their late pollen-preferred genes, we found that at least 17.7% of the top 300 in each cultivar do not overlap. This result proved the relevance of our candidate genes for studying late pollen development in japonica and indica rice. In addition, we determined that late pollen-preferred genes that were not conserved among rice types would be good targets for investigating late pollen development within each cultivar.

### Hormone metabolism and late pollen-preferred genes

Our MapMan analysis revealed 12 genes within the category of hormone metabolism – five for ethylene synthesis, three for auxin, and two for GA (Additional file 1: Table S6). All of these hormones are involved in late pollen development. For example, in *Petunia hybrida*, the level of 1-aminocyclopropane-1-carboxylic acid (ACC) in anthers is very low until the day before anthesis, when it then increases by up to 100-fold (Lindstrom et al. 1999), possibly as a result of activity by pPHACS2, an ACC synthase. Research with the ethylene-insensitive mutant *Never ripe* (*Nr*) has shown that ethylene plays a significant role in thermotolerance by regulating the level of sucrose in tomato (*Solanum lycopersicum*) (Firon et al. 2012). Auxin is also necessary for pollen maturation. For example, the auxin biosynthesis-defective *yuc2 yuc6* double mutant cannot produce grains (Cheng et al. 2006). The importance of auxin perception has also been demonstrated in a loss-of-function study with auxin receptor genes. There, the pollen grains from *tir1* and *afb1afb2afb3* multiple mutants age prematurely (Cecchetti et al. 2008). Gibberellic acid also has an essential role; GA-deficient mutants are mainly defective in pollen germination and elongation, while GA-insensitive mutants are mainly defective in pollen development (Chhun et al. 2007). *LOC_Os02g17780* encodes ent-copalyl diphosphate synthase1 (OsCPS1) while *LOC_Os06g02019* encodes ent-kaurenoic acid oxidase (OsKAO). Both enzymes are active in GA biosynthesis (Sakamoto et al. 2004), and mutants of both genes have significantly reduced frequencies of transmission through the male gametophytes, indicating that pollen germination and elongation essentially depend upon the de novo synthesis of GA in rice (Chhun et al. 2007). In addition, expression of *OsCPS1* and *OsKAO* peaks at the mature pollen stage (Chhun et al. 2007; Hirano et al. 2008). We also checked the expression patterns of other genes involved in GA biosynthesis and signalling pathways by using our meta-expression data with OsCPS1 and OsKAO (Additional file 2: Fig. S5). Among them, *GA3ox1*, *GA20ox3*, and *KO2* also showed late pollen-preferred expression patterns. Application of GA suppresses low temperature-induced male sterility (Sakata et al. 2014). Because pollen development is a complicated process accomplished by both microspores and the tapetum, various hormones, e.g., ethylene, auxin, and GA, might be involved in this process. Further experiments should focus on how hormone synthesis and signaling participate in the functioning of late pollen-preferred genes identified here.

### Major metabolic processes of cell wall organization during late pollen development in rice and *Arabidopsis*

During the late stages of pollen development in rice, cell wall organization is the most common MapMan term. Similar results have been reported for *Arabidopsis* pollen-preferred genes. One of the most distinctive structural features of a pollen grain is its double wall layer -- intine and exine (Vizcay-Barrena and Wilson, 2006). While the tapetum plays a pivotal role in exine formation, intine synthesis is largely dependent upon activity by the microspore (Nakamura et al. 2010; Yeung et al. 2011). The inner cell wall, intine, is simply composed of cellulose, pectin, and various other proteins (Noher de Halac et al., 2003). During pollen germination, the pollen tubes produce a new wall with two layers, the inner callosic layer and the outer layer, which again contains mainly cellulose, pectin, and other proteins (Donaldson and Knox, 2012). Among the late pollen-preferred genes found in our examination, 45 from *Arabidopsis* and 48 from rice are related with cell wall synthesis, proving their conserved roles in both species (Additional file 1: Table S10).

Our list shows that five genes from *Arabidopsis* and four from rice are related to cellulose synthesis. Mutations in any of three unique types of components within the cellulose synthase (CESA) complex -- CESA1, CESA3, or CESA6 -- result in the deformation of pollen grains owing to a deficiency in cellulose (Persson et al. 2007). Moreover, two *Arabidopsis* cellulose synthase-like D (CSLD) genes that are preferentially expressed in late pollen -- *CSLD1* and *CSLD4* -- have functions in pollen tube growth (Wang et al. 2011). Proteins from both are located in the Golgi apparatus and are transported to the plasma membrane in the tip region of the elongating tube, where cellulose is actively synthesized (Wang et al. 2011). In *csld1* and *csld4* mutants, the pollen tube wall is disorganized and exhibits less cellulose deposition. All of those observations suggest that the roles of rice homologs are conserved in their late pollen-preferred expression.

Among the genes investigated here, nine from *Arabidopsis* and five from rice are involved in the synthesis of cell wall proteins. In *Arabidopsis*, pollen-preferred *Arabinogalactan glycoprotein* (*AGP*)*6* and *AGP11* encode arabinogalactan glycoproteins associated with the cell wall. More than 50% of the grains from *agp6 agp11* are collapsed while the remainder display reduced germination and elongation (Coimbra et al. 2009; Coimbra et al. 2010). *Brassica campestris* male fertility 8, another pollen-specific AGP gene, functions in maintaining normal intine formation (Lin et al. 2014). One fasciclin-like AGP (FLA) gene, *FLA3*, is also involved in the development of intine walls (Li et al. 2010). All of this is evidence of conserved, pollen-preferred roles by rice homologs.

The grains of *Arabidopsis* and rice have 13 and 11 pollen-preferred PMEs, respectively. Among the 60 PME genes in the *Arabidopsis* ATH1 genome array, 18 are expressed in the pollen (Bosch et al. 2005). In many species, including tobacco (*Nicotiana tabacum*) and *Arabidopsis*, the apical end of the pollen tube is one layer composed mainly of pectin (Leroux et al. 2015). Pectins are synthesized in the Golgi apparatus and deposited as highly methyl-esterified polymers (Leroux et al. 2015). The PMEs de-esterify pectins and their activity can lead to cell-wall loosening or stiffening, depending on the apoplastic pH (Tian et al. 2006). Moreover, PME activity in pollen from a *vangard1* mutation is only 82% of that in the WT, and grains of the former have reduced tube growth (Jiang et al. 2005). Another pollen-specific PME, AtPPME1, is associated with slower pollen growth and irregularly shaped tubes (Tian et al. 2006). Rapid pollen tube growth after pollination might require strong activity by PME enzymes. In fact, such growth is completed within 50 min for rice while pollen tube discharge occurs in 4.0 to 9.5 h for *Arabidopsis* (Faure et al. 2002; Chen et al. 2008). Therefore, for both species, numerous genes related to cell wall organization and modification must be expressed in the mature grains in order to support rapid tube growth.

The biochemical function of MTD1 in plant pollen development has not yet been determined. One such gene from Arabidopsis, SHAVEN3 (SHV3), is required for root hair elongation (Parker et al. 2000; Jones et al. 2006). Furthermore, SHV3 and its paralogs are important factors for primary cell wall organization (Hayashi et al. 2008). Both SHV3-like 4 (SVL4) and SVL5 are specifically expressed in Arabidopsis pollen (Lalanne et al. 2004), suggesting conserved roles in pollen development for MTD1 in rice and SVL4 and SVL5 in Arabidopsis. More detailed functions should be analyzed in future molecular and biochemical studies.

### Cell wall modifications and metabolism of major carbohydrates for late pollen development are more important in rice than in *Arabidopsis*

Among the 16 genes related to cell wall modifications, six encode expansin proteins that are known to have cell-wall loosening activity. Although three expansin genes show a pattern of late pollen-preferred expression in *Arabidopsis* (Mollet et al. 2013), the six in rice are not homologous to those in *Arabidopsis*. Research with maize has indicated that pollen extracts have expansin-like activity that is unique to the cell walls of grass species but not eudicots (Cosgrove et al. 1997). Expansin facilitates pollen tube penetration through the stigma and style (Tabuchi et al. 2011). Maize pollen β-expansin preferentially binds to xylans and also solubilizes feruloylated arabinoxylan from cell walls in grasses (Tabuchi et al. 2011). Arabinoxylan is enriched in those grass cell walls (Sampedro et al. 2015). Thus, the arabinoxylan–cellulose interaction might be a target of grass pollen β-expansins (Sampedro et al. 2015). These distinct differences in structure between grass and dicot pollen cell walls can explain why MapMan terms related to cell wall modifications are more enriched in rice divergent genes than in those of *Arabidopsis* (Cho and Kende, 1997**;** Sampedro et al. 2015).

In *Arabidopsis* pollen, the development of starch grains begins at the vacuolated microspore stage, and grains are highly accumulated at the bi-cellular stage. At pollen maturity, only a few starch grains remain in the *Arabidopsis* plastid (Kuang and Musgrave, 1996; Tang et al. 2009). This contrasts with the features of rice pollen, where starch granules are abundant at the mature stage (Zhang et al. 2010). The higher starch content in mature rice pollen might explain why genes related to major carbohydrate metabolism are enriched in rice**.** Among the genes with MapMan terms related to carbohydrate metabolism that occur only in rice divergent genes, two are involved in the starch synthesis pathway while five genes are part of the pathway for starch degradation. We found one starch synthase and one ADP-glucose pyrophosphorylase (AGPase) in the starch synthesis pathway (Additional file 1: Table S5). Among them, *OsAGPL4* encodes AGPase and is involved in starch accumulation during pollen development. The *osagl4* mutant pollen exhibits a starch deficiency in pollen grains that causes defects in gene-transfer through the male gametophyte (Lee et al. 2016). Three amylase and two invertase genes participate in the starch degradation pathway. Because rapid growth by pollen tubes is a highly energy-consuming process, storage materials within pollen grains are mobilized for fuel (Goetz et al. 2017). Amylase catalyzes the hydrolysis of starch into sugar and invertase participates in the hydrolysis of sucrose into glucose and fructose. Regarding these genes, one starch degradation-defective mutant, tomato *alpha-Glucan water dikinase* (*LeGWD)*/*legwd*, interrupts gene-transfer through the male gametophyte (Nashilevitz et al. 2009). These differences between rice and *Arabidopsis* in their storage reserves for late pollen might explain why genes related to the metabolism of major carbohydrates are enriched in rice divergent genes, as coupled with late pollen development.

## Conclusion

By comparing the publicly available transcriptomes of sporophytes and male gametes throughout their development, we identified 627 late pollen-preferred genes that are conserved among japonica and indica cultivars of rice. We then performed global analyses of these candidate genes to study the processes of late pollen development, including maturation and germination. Functional classification analysis with a MapMan tool kit revealed that cell wall organization and metabolism are significantly associated with that stage. Comparative analysis of late pollen-preferred genes from rice and *Arabidopsis* demonstrated that those involved in cell wall modifications and major carbohydrate metabolisms are more important in rice. To evaluate the significance of our candidate genes, we used T-DNA insertional mutant population and the CRISPR/Cas9 system to determine the function of a rice gene encoding GPD protein by identifying defects in transfer through the male gamete. These candidates might be useful targets for future examinations of late pollen development in rice. Our results provide new tools to understand late pollen development.

## Methods

### Collection of microarray data and identification of mature pollen-preferred genes

We used 64 publicly available rice Affymetrix microarray data prepared from anthers and pollen in NCBI GEO to identify late pollen-preferred genes. For the comparative transcriptome analyses between rice and *Arabidopsis*, we downloaded *Arabidopsis* Affymetrix microarray data series GSE5630, GSE5631, GSE5632, GSE5633, GSE6162, GSE6696, GSE12316, GSE17343, and GSE27281. To examine these data, we used the Affy package encoded by R language to normalize the signal intensity and then transformed them to log_2_ values. The normalized data with averaged Affymetrix anatomical meta-expression data were then used for further investigations, e.g., KMC analysis, heatmap construction, and identification of the late pollen-preferred genes (Chandran et al. 2016).

### MapMan analysis

The MapMan program allows one to group genes into different functional categories and visualize data through various diagrams (Jung and An, 2012). To obtain their functional classifications, we uploaded RGAP locus IDs for 627 rice late pollen-preferred genes to the MapMan tool kit. We then investigated the metabolism and regulation overviews based on diverse overviews installed in that kit (Fig. 4). All data are detailed in Table S5 (Additional file 1).

### Histochemical GUS assay and microscopic analyses

Histochemical GUS-staining was performed as described by Jefferson et al. (1987) and Dai et al. (1996). The assayed flowers and pollen grains were photographed with an Olympus BX61 microscope (Olympus, Tokyo, Japan).

### RNA isolation and RT-PCR analysis

Total RNAs were extracted with Tri Reagent (MRC Inc., Cincinnati, OH, USA). Complementary DNA (cDNA) was synthesized as described previously (Lee and An, 2015). To evaluate the expression patterns of eight pollen-preferred genes that showed *GUS* activity, we prepared samples from the shoots and roots of 7-day-old seedlings, mature leaves, young panicles (2 cm), mature flowers, developing seeds at 3 d after pollination, and anthers at four different developmental stages. All primers for RT-PCR are listed in Table S11 (Additional file 1). The ubiquitin 5 gene (*Ubi5*) was used as an internal control.

### Rice T-DNA mutant screening

We searched for lines from our T-DNA insertional mutant population that had insertions within our candidate late pollen-preferred genes (Jeon et al. 2000; An et al. 2003). For *MTD1*, one T-DNA insertional line (*mtd1–1*) was selected. Seedlings were grown in a half-strength Murashige and Skoog (MS) medium. Their DNA was prepared from 7-day-old plants via the hexadecylotrimethylammonium method. Genotypes were determined by PCR using gene-specific primers and T-DNA primers (Additional file 1: Table S11). The T-DNA insertional lines without homozygous progenies or with rare homologous progenies were further genotyped to check for segregation distortion.

### Construction of the CRISPR/Cas9 vector to produce a homozygous mutant

To generate the single guide RNA (sgRNA) and Cas9 expression vector, we synthesized one set of oligomers targeting the third exon of MTD1 and inserted them into the Bsa I sites of the RGEB32 binary vector (Addgene plamid ID: 63142). Ligation products were transformed into *Escherichia coli*. The RGEB32 vector containing the sgRNA and Cas9 expression cassette was transformed into *Agrobacterium tumefaciens* LBA4404.

### Cytochemical analysis

Pollen grains were immersed in various staining solutions. Grains stained for 15 min with 0.1% calcofluor white were then monitored for the presence of intine under a UV light with an Olympus BX61 microscope. Grains stained with 0.001% auramine O in 17% sucrose were examined for exine, using the FITC channel of the Olympus BX61 microscope. Staining with Lugol’s iodine was used to detect the presence of starch.

## Additional files

Additional file 1: **Table S1.** Six series of microarray data comprising 64 slides (GPL2025) associated with anthers/pollen in rice. **Table S2.** Locus IDs and putative functions of late pollen-preferred genes from rice. **Table S3.** Locus IDs and promoter regions of genes used for promoter analysis with *GUS* reporter. **Table S4.** Classification of GO terms for biological processes associated with late pollen-preferred genes. **Table S5.** MapMan classification of late pollen-preferred genes. **Table S6.** Genes related to hormone metabolism term in MapMan. **Table S7.** Late pollen-preferred genes in *Arabidopsis*. **Table S8.** Assignment of rice orthologs to *Arabidopsis* late pollen-preferred genes. Locus numbers are shown in by red. **Table S9.** Assignment of *Arabidopsis* orthologs to rice late pollen-preferred genes. Locus numbers are shown in red. **Table S10.** MapMan terms related to cell wall organization and modifications in rice and *Arabidopsis*. **Table S11.** Primer sequences used in genotyping and real-time PCR. (DOCX 1980 kb)

Additional file 2: **Figure S1.** a. Order of developmental stages for anatomical samples. ACF, formation of archesporial cells; BG, bi-cellular gametophyte; Fl, flowering; Me, meiosis; Me1, meiotic leptotene; Me2, meiotic zygotene-pachytene; Me3, meiotic diplotene-tetrad; MP, mature pollen; PMe, pre-meiosis; GP, germinating pollen; TG, tri-cellular pollen; UG, uni-cellular gametophyte. Red bar, sample containing late pollen. b. Expression graph of 36 clusters after KMC analysis with 57,382 probes. Clusters 2 and 35 exhibited mature pollen-preferential patterns of expression and are marked with red boxes. **Figure S2.** Schematic representation of 3 promoter trap lines for T-DNA insertions. a. T-DNA was inserted into 17th intron of SacI homology domain-containing protein (*LOC_Os11g20384*) in Line 1A-13,819 (*mtd1–1*). b. Line 3A-05916 has T-DNA insertion in B12D protein (*LOC_Os07g17310*). BL, left T-DNA border; RB, right T-DNA border; Gray boxes, exons; lines, introns. **Figure S3.** Expression graph after KMC analysis of meta-expression data from *Arabidopsis*. Clusters marked with red box showed late pollen-preferred patterns. **Figure S4.** Heatmap for expression profiles of late pollen-preferred genes in *Arabidopsis*. **Figure S5.** Heatmap of genes involved in GA biosynthesis and signaling. *CPS*, *GA3ox1*, *KAO*, *GA20ox3*, and *KO2* showed late pollen-preferred expression patterns and are outlined with red boxes. (DOCX 227 kb)

## Abbreviations

ACC: 1-aminocyclopropane-1-carboxylic acid
AGPase: ADP-glucose pyrophosphorylase
CSLD: Cellulose synthase-like D
DAG: Diacylglycerol
DGK: Diacylglycerol kinase
GA: Gibberellic acid
GO: Gene Ontology
GPD: Glycerophosphoryl diester phosphodiesterase
KMC: K-means clustering
MTD: Male-gene Transfer Defective
NCBI GEO: National Center for Biotechnology Information Gene
PLD: Phospholipase
PME: Pectin methylesterase
RGAP: Rice Genome Annotated Project
ROAD: Expression Omnibus the Rice Oligonucleotide Array Database
TF: Transcription factor

## References

[CR1] An S, Park S, Jeong DH, Lee DY, Kang HG, Yu JH (2003). Generation and analysis of end sequence database for T-DNA tagging lines in rice. Plant Physiol.

[CR2] Aya K, Suzuki G, Suwabe K, Hobo T, Takahashi H, Shiono K (2011). Comprehensive network analysis of anther-expressed genes in rice by the combination of 33 laser microdissection and 143 spatiotemporal microarrays. PLoS One.

[CR3] Becker JD, Boavida LC, Carneiro J, Haury M, Feijo JA (2003). Transcriptional profiling of Arabidopsis tissues reveals the unique characteristics of the pollen transcriptome. Plant Physiol.

[CR4] Bedinger P (1992). The remarkable biology of pollen. Plant Cell.

[CR5] Bosch M, Cheung AY, Hepler PK (2005). Pectin methylesterase, a regulator of pollen tube growth. Plant Physiol.

[CR6] Cao P, Jung KH, Choi D, Hwang D, Ronald PC (2012). The Rice oligonucleotide Array database: an atlas of rice gene expression. Rice.

[CR7] Cecchetti V, Altamura MM, Falasca G, Costantino P, Cardarelli M (2008). Auxin regulates Arabidopsis anther dehiscence, pollen maturation, and filament elongation. Plant Cell.

[CR8] Cecchetti V, Celebrin D, Napoli N, Ghelli R, Brunetti P, Costantino P (2017). An auxin maximum in the middle layer controls stamen development and pollen maturation in Arabidopsis. New Phytol.

[CR9] Chandran AKN, Jeong HY, Jung KH, Lee C (2016). Development of functional modules based on co-expression patterns for cell-wall biosynthesis related genes in rice. J Plant Biol.

[CR10] Chen SQ, Wang Z, Liu MX, Xie ZW, Wang HH (2008). Pollen grain germination and pollen tube growth in pistil of rice. Rice Sci.

[CR11] Cheng Y, Dai X, Zhao Y (2006). Auxin biosynthesis by the YUCCA flavin monooxygenases controls the formation of floral organs and vascular tissues in Arabidopsis. Genes Dev.

[CR12] Chhun T, Aya K, Asano K, Yamamoto E, Morinaka Y, Watanabe M (2007). Gibberellin regulates pollen viability and pollen tube growth in rice. Plant Cell.

[CR13] Cho HT, Kende H (1997). Expansins and internodal growth of Deepwater rice. Plant Physiol.

[CR14] Coimbra S, Costa M, Jones B, Mendes MA, Pereira LG (2009). Pollen grain development is compromised in Arabidopsis agp6 agp11 null mutants. J Exp Bot.

[CR15] Coimbra S, Costa M, Mendes MA, Pereira AM, Pinto J, Pereira LG (2010). Early germination of Arabidopsis pollen in a double null mutant for the arabinogalactan protein genes AGP6 and AGP11. Plant Reprod.

[CR16] Cosgrove DJ, Bedinger P, Durachko DM (1997). Group I allergens of grass pollen as cell wall-loosening agents. Proc Natl Acad Sci U S A.

[CR17] Dai Z, Gao J, An K, Lee JM, Edwards GE, An G (1996). Promoter elements controlling developmental and environmental regulation of a tobacco ribosomal protein gene L34. Plant Mol Biol.

[CR18] Deveshwar P, Bovill WD, Sharma R, Able JA, Kapoor S (2011). Analysis of anther transcriptomes to identify genes contributing to meiosis and male gametophyte development in rice. BMC Plant Biol.

[CR19] Dobritsa AA, Geanconteri A, Shrestha J, Carlson A, Kooyers N, Coerper D (2011). A large-scale genetic screen in Arabidopsis to identify genes involved in pollen exine production. Plant Physiol.

[CR20] Donaldson LA, Knox JP (2012). Localization of cell wall polysaccharides in normal and compression wood of radiata pine: relationships with lignification and microfibril orientation. Plant Physiol.

[CR21] Edgar R, Domrachev M, Lash AE (2002). Gene expression omnibus: NCBI gene expression and hybridization array data repository. Nucleic Acids Res.

[CR22] Faure JE, Rotman N, Fortune P, Dumas C (2002). Fertilization in *Arabidopsis thaliana* wild type: developmental stages and time course. Plant J.

[CR23] Firon N, Pressman E, Meir S, Khoury R, Altahan L (2012). Ethylene is involved in maintaining tomato (Solanum lycopersicum) pollen quality under heat-stress conditions. AoB Plants.

[CR24] Fujita M, Horiuchi Y, Ueda Y, Mizuta Y, Kubo T, Yano K (2010). Rice expression atlas in reproductive development. Plant Cell Physiol..

[CR25] Goetz M, Guivarch A, Hirsche J, Bauerfeind MA, Gonzalez MC, Hyun TK (2017). Metabolic control of tobacco pollination by sugars and invertases. Plant Physiol.

[CR26] Guan Y, Meng X, Khanna R, LaMontagne E, Liu Y, Zhang S (2014). Phosphorylation of a WRKY transcription factor by MAPKs is required for pollen development and function in Arabidopsis. PLoS Genet.

[CR27] Hao H, Li Y, Hu Y, Lin J (2005). Inhibition of RNA and protein synthesis in pollen tube development of *Pinus bungeana* by actinomycin D and cycloheximide. New Phytol.

[CR28] Hayashi S, Ishii T, Matsunaga T, Tominaga R, Kuromori T, Wada T (2008). The glycerophosphoryl diester phosphodiesterase-like proteins SHV3 and its homologs play important roles in cell wall organization. Plant Cell Physiol..

[CR29] Hirano K, Aya K, Hobo T, Sakakibara H, Kojima M, Shim RA (2008). Comprehensive transcriptome analysis of phytohormone biosynthesis and signaling genes in microspore/pollen and tapetum of rice. Plant Cell Physiol.

[CR30] Honys D, Twell D (2003). Comparative analysis of the Arabidopsis pollen transcriptome. Plant Physiol.

[CR31] Honys D, Twell D (2004). Transcriptome analysis of haploid male gametophyte development in Arabidopsis. Genome Biol.

[CR32] Jefferson RA, Kavanagh TA, Bevan MW (1987). GUS fusions: beta-glucuronidase as a sensitive and versatile gene fusion marker in higher plants. EMBO J.

[CR33] Jeon JS, Lee S, Jung KH, Jun SH, Jeong DH, Lee J (2000). T-DNA insertional mutagenesis for functional genomics in rice. Plant J.

[CR34] Jeong DH, An S, Kang HG, Moon S, Han JJ, Park S (2002). T-DNA insertional mutagenesis for activation tagging in rice. Plant Physiol.

[CR35] Jeong DH, An S, Park S, Kang HG, Park GG, Kim SR (2006). Generation of a flanking sequence-tag database for activation-tagging lines in japonica rice. Plant J.

[CR36] Jiang, L., Yang, S.L, Xie L.F., Puah, C.S., Zhang, X.Q., Yang, W.C., *et al.* (2005) VANGUARD1 encodes a pectin methylesterase that enhances pollen tube growth in the Arabidopsis style and transmitting tract. Plant Cell 17, 584–596 10.1105/tpc.104.027631 PMC548828 15659637

[CR37] Jones MA, Raymond MJ, Smirnoff N (2006). Analysis of the root-hair morphogenesis transcriptome reveals the molecular identity of six genes with roles in root-hair development in Arabidopsis. Plant J.

[CR38] Jung KH, An G (2012). Application of MapMan and RiceNet drives systematic analyses of the early heat stress transcriptome in rice seedlings. J. Plant Biol..

[CR39] Jung KH, Dardick C, Bartley LE, Cao PJ, Phetsom J, Canlas P (2008). Refinement of light-responsive transcript lists using rice oligonucleotide arrays, evaluation of gene-redundancy. PLoS One.

[CR40] Krichevsky A, Kozlovsky SV, Tian GW, Chen MH, Zaltsman A, Citovsky V (2007). How pollen tubes grow. Dev Biol.

[CR41] Kuang A, Musgrave ME (1996). Dynamics of vegetative cytoplasm during generative cell formation and pollen maturation in *Arabidopsis thaliana*. Protoplasma.

[CR42] Lalanne E, Honys D, Johnson A, Borner GH, Lilley KS, Dupree P (2004). SETH1 and SETH2, two components of the glycosylphosphatidylinositol anchor biosynthetic pathway, are required for pollen germination and tube growth in Arabidopsis. Plant Cell.

[CR43] Lee SK, Eom JS, Hwang SK, Shin D, An G, Okita TW (2016). Plastidic phosphoglucomutase and ADP-glucose pyrophosphorylase mutants impair starch synthesis in rice pollen grains and cause male sterility. J Exp Bot.

[CR44] Lee YS, An G (2015). OsGI controls flowering time by modulating rhythmic flowering time regulators preferentially under short day in rice. J. Plant Biol..

[CR45] Leroux C, Bouton S, Kiefer-Meyer MC, Fabrice TN, Mareck A, Guenin S (2015). PECTIN METHYLESTERASE48 is involved in Arabidopsis pollen grain germination. Plant Physiol.

[CR46] Li J, Yu M, Geng LL, Zhao J (2010). The fasciclin-like arabinogalactan protein gene, FLA3, is involved in microspore development of Arabidopsis. Plant J.

[CR47] Lin S, Dong H, Zhang F, Qiu L, Wang FZ, Cao JS (2014). BcMF8, a putative arabinogalactan protein-encoding gene, contributes to pollen wall development, aperture formation and pollen tube growth in *Brassica campestris*. Ann Bot.

[CR48] Lindstrom JT, Lei CH, Jones ML, Woodson WR (1999). Accumulation of 1-aminocyclopropane-1-carboxylic acid (ACC) in petunia pollen is associated with expression of a pollen-specific ACC synthase late in development. J Am Soc Hortic Sci.

[CR49] Mascarenhas JP (1993). Molecular mechanisms of pollen tube growth and differentiation. Plant Cell.

[CR50] Mollet JC, Leroux C, Dardelle F, Lehner A (2013). Cell wall composition, biosynthesis and remodeling during pollen tube growth. Plants.

[CR51] Moon S, Kim SR, Zhao G, Yi J, Yoo Y, Jin P (2013). Rice GLYCOSYLTRANSFERASE1 encodes a GLYCOSYLTRANSFERASE essential for pollen wall formation. Plant Physiol.

[CR52] Nakamura AT, Longhi-Wagner HM, Scatena VL (2010). Anther and pollen development in some species of Poaceae (Poales). Braz J Biol.

[CR53] Nashilevitz S, Melamed-Bessudo C, Aharoni A, Kossmann J, Wolf S, Levy A (2009). The legwd mutant uncovers the role of starch phosphorylation in pollen development and germination in tomato. Plant J.

[CR54] Noher de Halac I, Cismondi IA, Rodriguez-Garcia MI, Fama G (2003). Distribution of pectins in the pollen apertures of *Oenothera hookeri*.Velans ster/+ster. Biocell.

[CR55] Oo MM, Bae HK, Nguyen TD, Moon S, Oh SA, Kim JH (2014). Evaluation of rice promoters conferring pollen-specific expression in a heterologous system, Arabidopsis. Plant Reprod.

[CR56] Owen HA, Makaroff CA (1995). Ultrastructure of microsporogenesis and microgametogenesis in *Arabidopsis thaliana* (L.) Heynh. Ecotype Wassilewskija (Brassicaceae). Protoplasma.

[CR57] Parker JS, Cavell AC, Dolan L, Roberts K, Grierson CS (2000). Genetic interactions during root hair morphogenesis in Arabidopsis. Plant Cell.

[CR58] Persson S, Paredez A, Carroll A, Palsdottir H, Doblin M, Poindexter P, Khitrov N, Auer M, Somerville CR (2007). Genetic evidence for three unique components in primary cell-wall cellulose synthase complexes in Arabidopsis. Proc Natl Acad Sci U S A.

[CR59] Prasad PVV, Boote KJ, Allen LH, Sheehy JE, Thomas JMG (2006). Species, ecotype and cultivar differences in spikelet fertility and harvest index of rice in response to high temperature stress. Field Crops Res.

[CR60] Russell SD, Gou X, Wong CE, Wang X, Yuan T, Wei X (2012). Genomic profiling of rice sperm cell transcripts reveals conserved and distinct elements in the flowering plant male germ lineage. New Phytol.

[CR61] Ryu CH, You JH, Kang HG, Hur J, Kim YH, Han MJ (2004). Generation of T-DNA tagging lines with a bidirectional gene trap vector and the establishment of an insertion-site database. Plant Mol Biol.

[CR62] Sakamoto T, Miyura K, Itoh H, Tatsumi T, Ueguchi-Tanaka M, Ishiyama K (2004). An overview of gibberellin metabolism enzyme genes and their related mutants in rice. Plant Physiol.

[CR63] Sakata T, Oda S, Tsunaga Y, Shomura H, Kawagishi-Kobayashi M, Aya K (2014). Reduction of gibberellin by low temperature disrupts pollen development in rice. Plant Physiol.

[CR64] Sampedro J, Guttman M, Li LC, Cosgrove DJ (2015). Evolutionary divergence of beta-expansin structure and function in grasses parallels emergence of distinctive primary cell wall traits. Plant J.

[CR65] Shi J, Cui M, Yang L, Kim YJ, Zhang D (2015). Genetic and biochemical mechanisms of pollen wall development. Trends Plant Sci.

[CR66] Suwabe K, Suzuki G, Takahashi H, Shiono K, Endo M, Yano K (2008). Separated transcriptomes of male gametophyte and tapetum in rice: validity of a laser microdissection (LM) microarray. Plant Cell Physiol..

[CR67] Suzuki T, Masaoka K, Nishi M, Nakamura K, Ishiguro S (2008). Identification of Kaonashi mutants showing abnormal pollen exine structure in *Arabidopsis thaliana*. Plant Cell Physiol..

[CR68] Tabuchi A, Li LC, Cosgrove DJ (2011). Matrix solubilization and cell wall weakening by β-expansin (group-1 allergen) from maize pollen. Plant J.

[CR69] Tang LY, Nagata N, Matsushima R, Chen Y, Yoshioka Y, Sakamoto W (2009). Visualization of plastids in pollen grains: involvement of FtsZ1 in pollen plastid division. Plant Cell Physiol..

[CR70] Tian GW, Chen MH, Zaltsman A, Citovsky V (2006). Pollen-specific pectin methylesterase involved in pollen tube growth. Dev Biol.

[CR71] Vizcay-Barrena G, Wilson ZA (2006). Altered tapetal PCD and pollen wall development in the Arabidopsis ms1 mutant. J Exp Bot.

[CR72] Wang W, Wang L, Chen C, Xiong G, Tan XY, Yang KZ (2011). Arabidopsis CSLD1 and CSLD4 are required for cellulose deposition and normal growth of pollen tubes. J Exp Bot.

[CR73] Wei LQ, Xu WY, Deng ZY, Su Z, Xue Y, Wang T (2010). Genome-scale analysis and comparison of gene expression profiles in developing and germinated pollen in *Oryza sativa*. BMC Genomics.

[CR74] Xu J, Ding Z, Vizcay-Barrena G, Shi J, Liang W, Yuan Z (2014). ABORTED MICROSPORES acts as a master regulator of pollen wall formation in Arabidopsis. Plant Cell.

[CR75] Yeung EC, Oinam GS, Yeung SS, Harry I (2011). Anther, pollen and tapetum development in safflower, *Carthamus tinctorius* L. Plant Reprod.

[CR76] Yoo YH, Choi HK, Jung KH (2015). Genome-wide identification and analysis of genes associated with lysigenous aerenchyma formation in rice roots. J. Plant Biol..

[CR77] Zhang DB, Luo X, Zhu L (2011). Cytological analysis and genetic control of rice anther development. J Genet Genom.

[CR78] Zhang H, Liang WQ, Yang XJ, Luo X, Jiang N, Ma H (2010). Carbon starved anther encodes a MYB domain protein that regulates sugar partitioning required for rice pollen development. Plant Cell.

