# Supplementary material for: Genome-wide analyses of late pollen-preferred genes conserved in various rice cultivars and functional identification of a gene involved in the key processes of late pollen development
Source: Rice (N Y). 2018 Apr 23;11:28. doi: 10.1186/s12284-018-0219-0 (PMC5913055; doi:10.1186/s12284-018-0219-0)
Supplement: Supplementary file 1 — Table S1. Six series of microarray data comprising 64 slides (GPL2025) associated with anthers/pollen in rice. Table S2. Locus IDs and putative functions of late pollen-preferred genes from rice. Table S3. Locus IDs and promoter regions of genes used for promoter analysis with GUS reporter. Table S4. Classification of GO terms for biological processes associated with late pollen-preferred genes. Table S5. MapMan classification of late pollen-preferred genes. Table S6. Genes related to hormone metabolism term in MapMan. Table S7. Late pollen-preferred genes in Arabidopsis. Table S8. Assignment of rice orthologs to Arabidopsis late pollen-preferred genes. Locus numbers are shown in by red. Table S9. Assignment of Arabidopsis orthologs to rice late pollen-preferred genes. Locus numbers are shown in red. Table S10. MapMan terms related to cell wall organization and modifications in rice and Arabidopsis. Table S11. Primer sequences used in genotyping and real-time PCR. (DOCX 1980 kb) [file 12284_2018_219_MOESM2_ESM.docx]

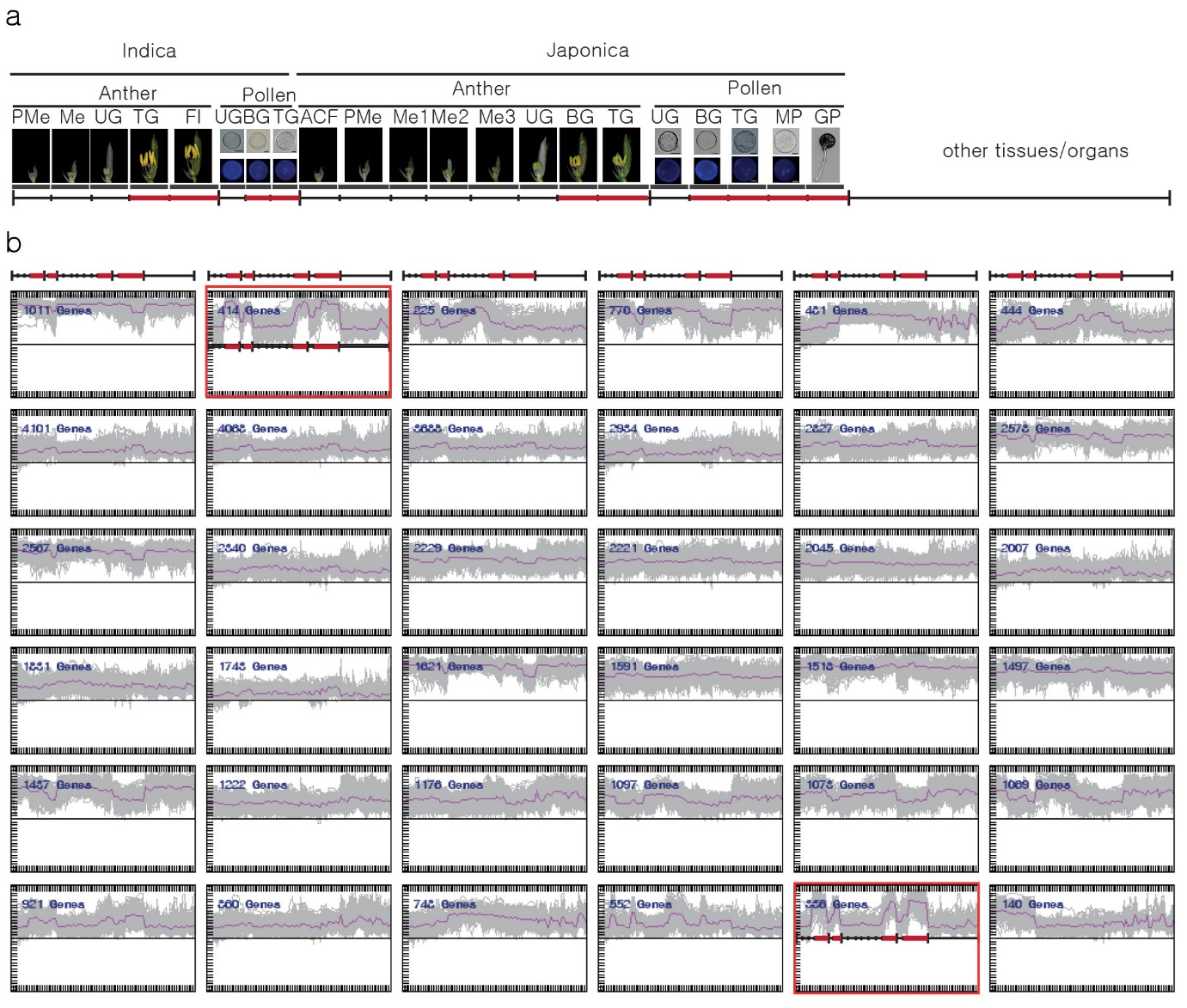


**Figure S1.** a. Order of developmental stages for anatomical samples. ACF, formation of archesporial cells; BG, bi-cellular gametophyte; Fl, flowering; Me, meiosis; Me1, meiotic leptotene; Me2, meiotic zygotene-pachytene; Me3, meiotic diplotene-tetrad; MP, mature pollen; PMe, pre-meiosis; GP, germinating pollen; TG, tri-cellular pollen; UG, uni-cellular gametophyte. Red bar, sample containing late pollen. b. Expression graph of 36 clusters after KMC analysis with 57,382 probes. Clusters 2 and 35 exhibited mature pollen-preferential patterns of expression and are marked with red boxes.


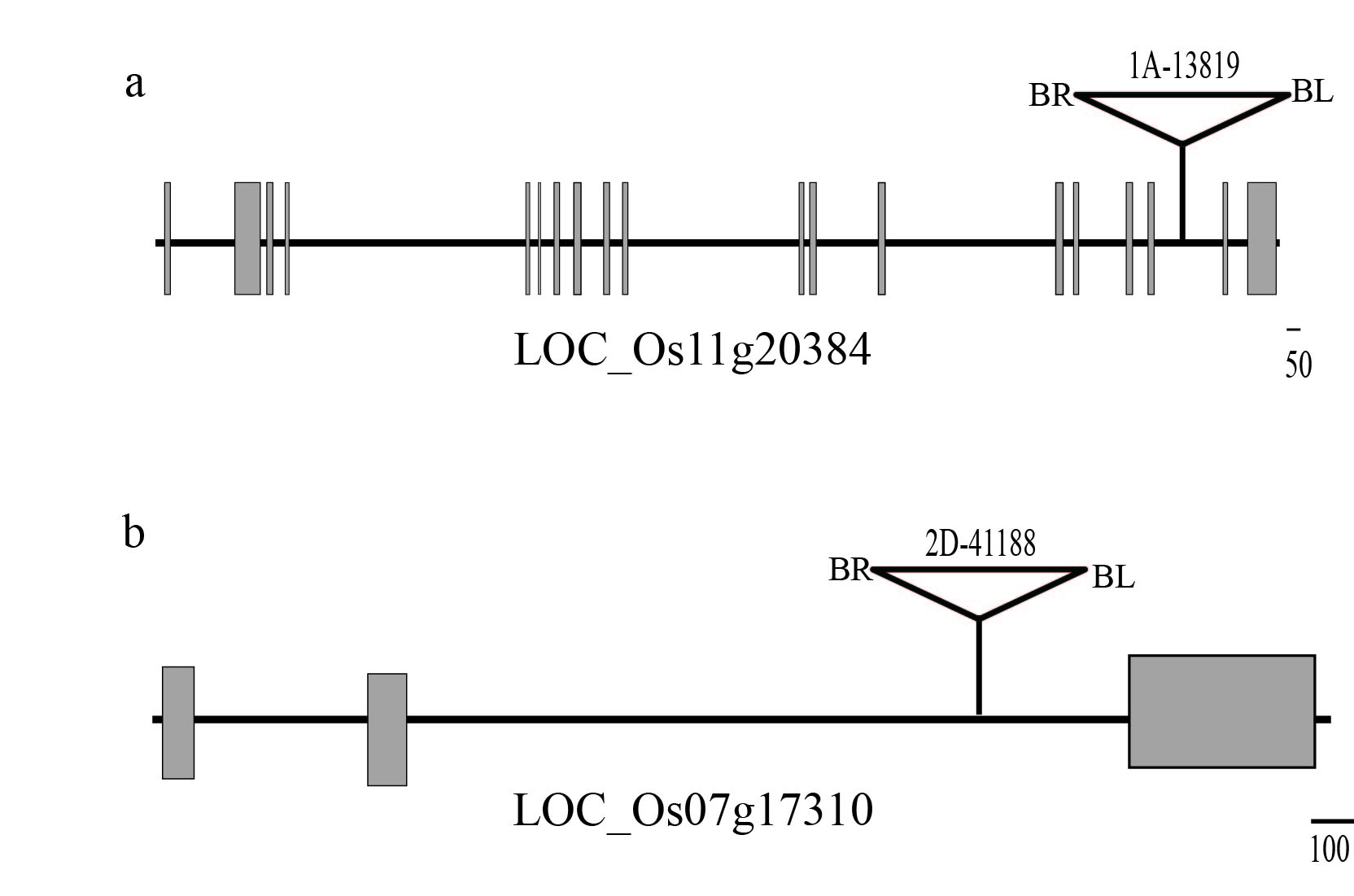


**Figure S2.** Schematic representation of 3 promoter trap lines for T-DNA insertions. a. T-DNA was inserted into 17^th^ intron of SacI homology domain-containing protein (*LOC_Os11g20384*) in Line 1A-13819 (*mtd1-1*). b. Line 3A-05916 has T-DNA insertion in B12D protein (*LOC_Os07g17310*). BL, left T-DNA border; RB, right T-DNA border; Gray boxes, exons; lines, introns.

**
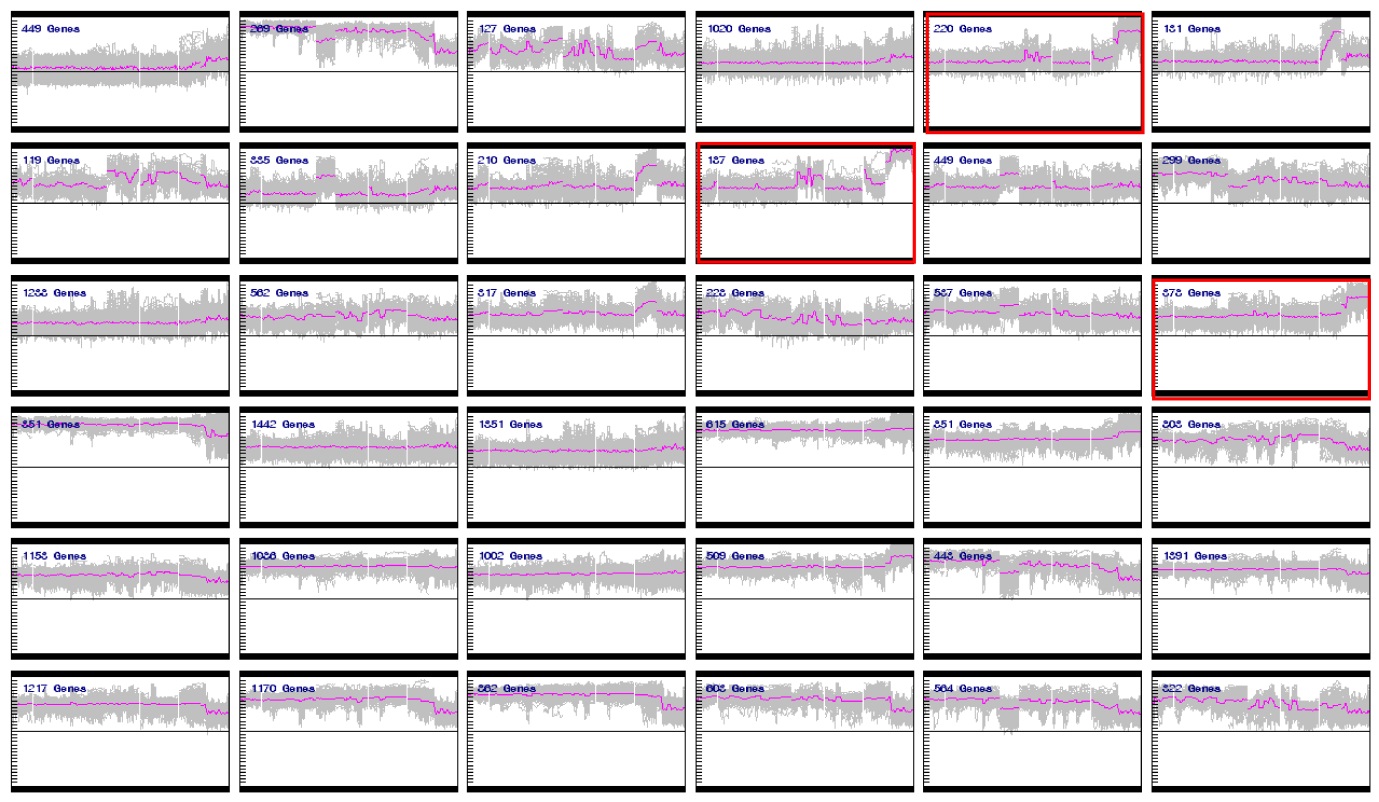
 Figure S3.** Expression graph after KMC analysis of meta-expression data from *Arabidopsis*. Clusters marked with red box showed late pollen-preferred patterns.

**
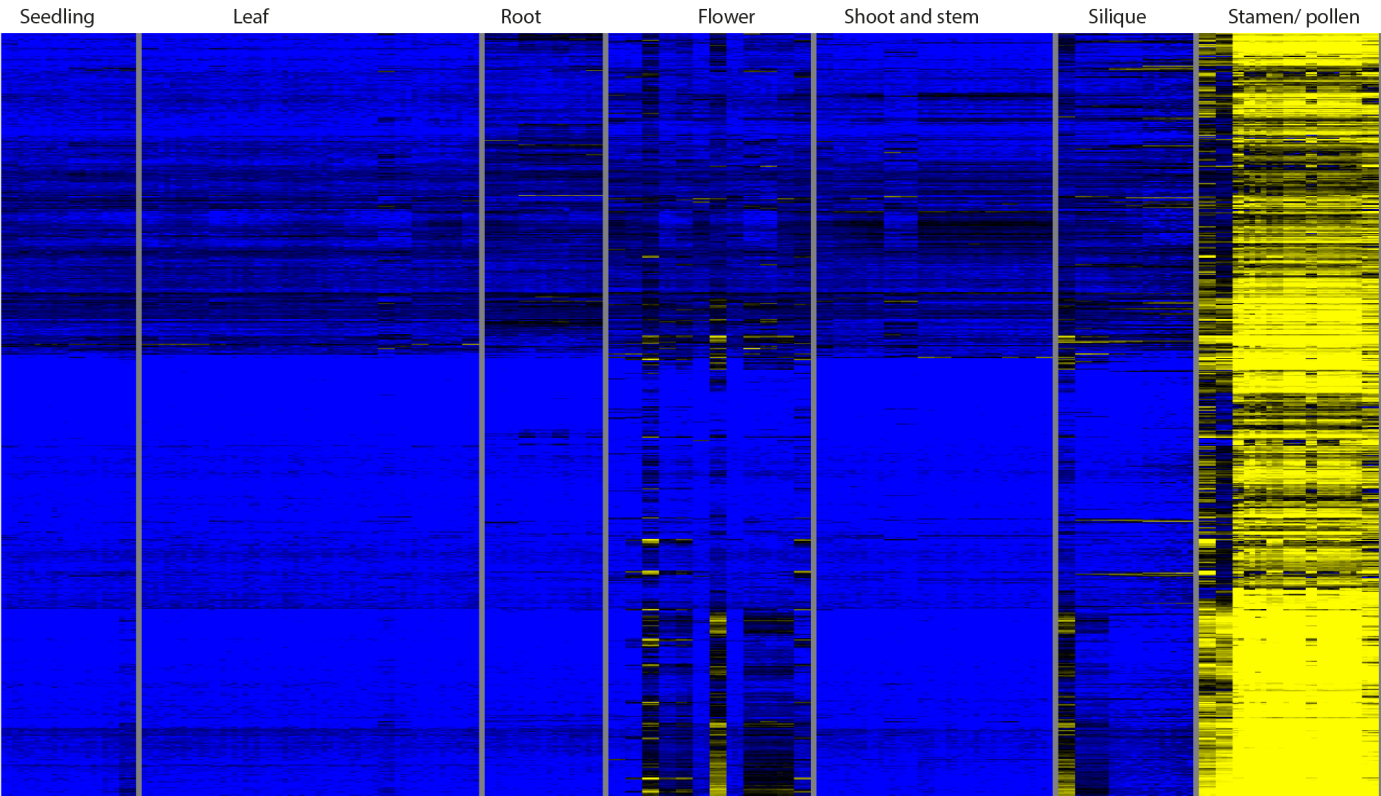
 Figure S4**. Heatmap for expression profiles of late pollen-preferred genes in *Arabidopsis*.

**
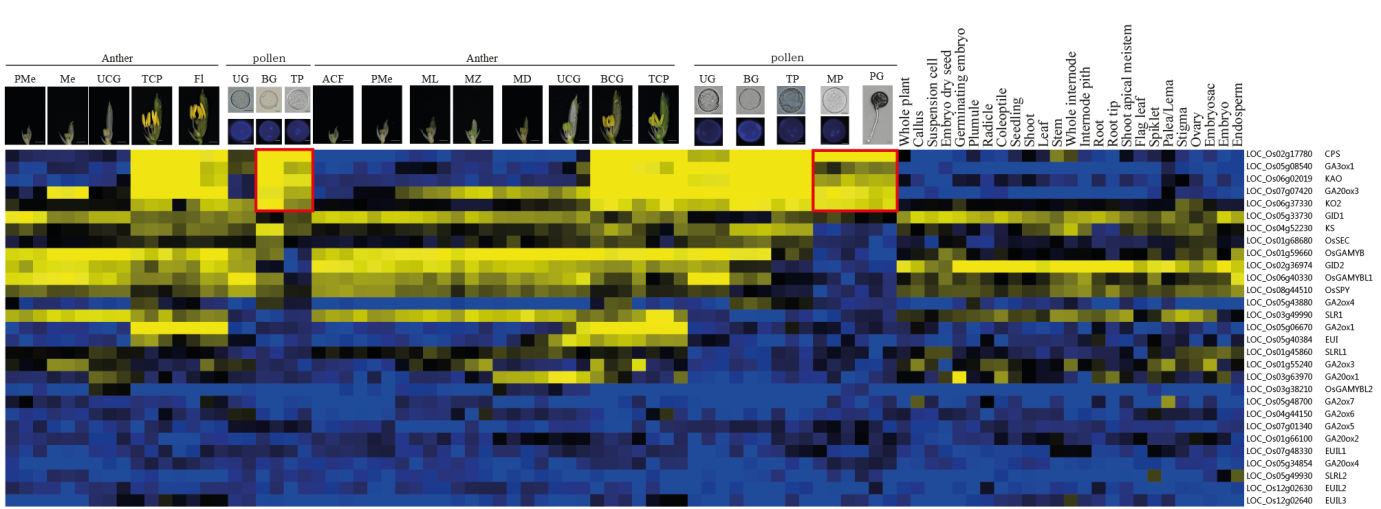
Figure S5.** Heatmap of genes involved in GA biosynthesis and signaling. *CPS*, *GA3ox1*, *KAO*, *GA20ox3*, and *KO2* showed late pollen-preferred expression patterns and are outlined with red boxes.
