# Supplementary material for: Genome-wide analyses of late pollen-preferred genes conserved in various rice cultivars and functional identification of a gene involved in the key processes of late pollen development
Source: Rice (N Y). 2018 Apr 23;11:28. doi: 10.1186/s12284-018-0219-0 (PMC5913055; doi:10.1186/s12284-018-0219-0)
Supplement: Supplementary file 2 — Figure S1. a. Order of developmental stages for anatomical samples. ACF, formation of archesporial cells; BG, bi-cellular gametophyte; Fl, flowering; Me, meiosis; Me1, meiotic leptotene; Me2, meiotic zygotene-pachytene; Me3, meiotic diplotene-tetrad; MP, mature pollen; PMe, pre-meiosis; GP, germinating pollen; TG, tri-cellular pollen; UG, uni-cellular gametophyte. Red bar, sample containing late pollen. b. Expression graph of 36 clusters after KMC analysis with 57,382 probes. Clusters 2 and 35 exhibited mature pollen-preferential patterns of expression and are marked with red boxes. Figure S2. Schematic representation of 3 promoter trap lines for T-DNA insertions. a. T-DNA was inserted into 17th intron of SacI homology domain-containing protein (LOC_Os11g20384) in Line 1A-13,819 (mtd1–1). b. Line 3A-05916 has T-DNA insertion in B12D protein (LOC_Os07g17310). BL, left T-DNA border; RB, right T-DNA border; Gray boxes, exons; lines, introns. Figure S3. Expression graph after KMC analysis of meta-expression data from Arabidopsis. Clusters marked with red box showed late pollen-preferred patterns. Figure S4. Heatmap for expression profiles of late pollen-preferred genes in Arabidopsis. Figure S5. Heatmap of genes involved in GA biosynthesis and signaling. CPS, GA3ox1, KAO, GA20ox3, and KO2 showed late pollen-preferred expression patterns and are outlined with red boxes. (DOCX 227 kb) [file 12284_2018_219_MOESM1_ESM.docx]

**Table S1.** Six series of microarray data comprising 64 slides (GPL2025) associated with anthers/pollen in rice.

| GEO Series No. | Accession No | Tissue | Stage | Cultivar |
| --- | --- | --- | --- | --- |
| GSE27726 | GSM686458 | Anther | Pre-meiotic stage | Indica |
| GSE27726 | GSM686459 | Anther | Pre-meiotic stage | Indica |
| GSE27726 | GSM686460 | Anther | Pre-meiotic stage | Indica |
| GSE27726 | GSM686461 | Anther | Meiotic stage | Indica |
| GSE27726 | GSM686462 | Anther | Meiotic stage | Indica |
| GSE27726 | GSM686463 | Anther | Meiotic stage | Indica |
| GSE27726 | GSM686464 | Anther | Uni-cellular gametopyte stage | Indica |
| GSE27726 | GSM686465 | Anther | Uni-cellular gametopyte stage | Indica |
| GSE27726 | GSM686466 | Anther | Uni-cellular gametopyte stage | Indica |
| GSE27726 | GSM686467 | Anther | Tri-cellular mature pollen stage | Indica |
| GSE27726 | GSM686468 | Anther | Tri-cellular mature pollen stage | Indica |
| GSE27726 | GSM686469 | Anther | Tri-cellular mature pollen stage | Indica |
| GSE19024 | GSM470656 | Anther | Flowering stage | Indica |
| GSE19024 | GSM470657 | Anther | Flowering stage | Indica |
| GSE19024 | GSM470754 | Anther | Flowering stage | Indica |
| GSE19024 | GSM470755 | Anther | Flowering stage | Indica |
| GSE29080 | GSM720598 | Pollen | Uni-cellular gametopyte stage | Indica |
| GSE29080 | GSM720599 | Pollen | Uni-cellular gametopyte stage | Indica |
| GSE29080 | GSM720600 | Pollen | Bi-cellular gametopyte stage | Indica |
| GSE29080 | GSM720601 | Pollen | Bi-cellular gametopyte stage | Indica |
| GSE29080 | GSM720602 | Pollen | Tri-cellular mature pollen stage | Indica |
| GSE29080 | GSM720603 | Pollen | Tri-cellular mature pollen stage | Indica |
| GSE13988 | GSM351427 | Anther | Archesporial cells forming stage | Japonica |
| GEO Series No. | Accession No | Tissue | Stage | Cultivar |
| GSE13988 | GSM351428 | Anther | Archesporial cells forming stage | Japonica |
| GSE13988 | GSM351429 | Anther | Archesporial cells forming stage | Japonica |
| GSE13988 | GSM351430 | Anther | Pre-meiotic stage | Japonica |
| GSE13988 | GSM351431 | Anther | Pre-meiotic stage | Japonica |
| GSE13988 | GSM351432 | Anther | Pre-meiotic stage | Japonica |
| GSE13988 | GSM351433 | Anther | Meiotic leptotene stage | Japonica |
| GSE13988 | GSM351434 | Anther | Meiotic leptotene stage | Japonica |
| GSE13988 | GSM351435 | Anther | Meiotic leptotene stage | Japonica |
| GSE13988 | GSM351436 | Anther | Meiotic leptotene stage | Japonica |
| GSE13988 | GSM351437 | Anther | Meiotic zygotene-pachytene stage | Japonica |
| GSE13988 | GSM351438 | Anther | Meiotic zygotene-pachytene stage | Japonica |
| GSE13988 | GSM351439 | Anther | Meiotic zygotene-pachytene stage | Japonica |
| GSE13988 | GSM351440 | Anther | Meiotic diplotene-tetrad stage | Japonica |
| GSE13988 | GSM351441 | Anther | Meiotic diplotene-tetrad stage | Japonica |
| GSE13988 | GSM351442 | Anther | Meiotic diplotene-tetrad stage | Japonica |
| GSE13988 | GSM351443 | Anther | Meiotic diplotene-tetrad stage | Japonica |
| GSE13988 | GSM351444 | Anther | Uni-cellular gametopyte stage | Japonica |
| GSE13988 | GSM351445 | Anther | Uni-cellular gametopyte stage | Japonica |
| GSE13988 | GSM351446 | Anther | Uni-cellular gametopyte stage | Japonica |
| GSE13988 | GSM351447 | Anther | Bi-cellular gametopyte stage | Japonica |
| GSE13988 | GSM351448 | Anther | Bi-cellular gametopyte stage | Japonica |
| GSE13988 | GSM351449 | Anther | Bi-cellular gametopyte stage | Japonica |
| GSE13988 | GSM351450 | Anther | Tri-cellular mature pollen stage | Japonica |
| GSE13988 | GSM351451 | Anther | Tri-cellular mature pollen stage | Japonica |
| GSE13988 | GSM351452 | Anther | Tri-cellular mature pollen stage | Japonica |
| GEO Series No. | Accession No | Tissue | Stage | Cultivar |
| GSE7951 | GSM195227 | Anther | Flowering stage | Japonica |
| GSE27988 | GSM692536 | Pollen | Uni-cellular gametopyte stage | Japonica |
| GSE27988 | GSM692537 | Pollen | Uni-cellular gametopyte stage | Japonica |
| GSE27988 | GSM692538 | Pollen | Uni-cellular gametopyte stage | Japonica |
| GSE27988 | GSM692539 | Pollen | Bi-cellular gametopyte stage | Japonica |
| GSE27988 | GSM692540 | Pollen | Bi-cellular gametopyte stage | Japonica |
| GSE27988 | GSM692541 | Pollen | Bi-cellular gametopyte stage | Japonica |
| GSE27988 | GSM692542 | Pollen | Tri-cellular immature pollen stage | Japonica |
| GSE27988 | GSM692543 | Pollen | Tri-cellular immature pollen stage | Japonica |
| GSE27988 | GSM692544 | Pollen | Tri-cellular immature pollen stage | Japonica |
| GSE27988 | GSM692545 | Pollen | Mature pollen stage | Japonica |
| GSE27988 | GSM692546 | Pollen | Mature pollen stage | Japonica |
| GSE27988 | GSM692547 | Pollen | Mature pollen stage | Japonica |
| GSE27988 | GSM692548 | Pollen | Pollen germination stage | Japonica |
| GSE27988 | GSM692549 | Pollen | Pollen germination stage | Japonica |
| GSE27988 | GSM692550 | Pollen | Pollen germination stage | Japonica |

**Table S2.** Locus IDs and putative functions of late pollen-preferred genes from rice.

| Locus_id | Putative Function |
| --- | --- |
| LOC_Os06g03390.1 | expressed protein |
| LOC_Os05g20570.1 | invertase/pectin methylesterase inhibitor family protein |
| LOC_Os10g33240.1 | expressed protein |
| LOC_Os03g24160.1 | phosphatidylinositol-4-phosphate 5-kinase |
| LOC_Os01g69240.1 | CBS domain containing membrane protein |
| LOC_Os11g05510.1 | carbonic anhydrase family protein |
| LOC_Os12g07700.1 | nifU |
| LOC_Os05g20150.1 | TKL_IRAK_CrRLK1L-1.11 - The CrRLK1L-1 subfamily has homology to the CrRLK1L homolog |
| LOC_Os03g05770.1 | peroxidase precursor |
| LOC_Os01g60080.1 | monocopper oxidase |
| LOC_Os07g13440.1 | RALFL12 - Rapid ALkalinization Factor RALF family protein precursor |
| LOC_Os02g03520.1 | THION25 - Plant thionin family protein precursor |
| LOC_Os06g35590.1 | reticuline oxidase-like protein precursor |
| LOC_Os04g25150.1 | pollen allergen |
| LOC_Os04g49650.1 | DUF581 domain containing protein |
| LOC_Os11g31400.1 | expressed protein |
| LOC_Os10g17660.1 | profilin domain containing protein |
| LOC_Os09g30030.1 | expressed protein |
| LOC_Os04g37460.1 | glutamate decarboxylase |
| LOC_Os02g51730.1 | dnaJ homolog subfamily C member 7 |
| LOC_Os03g19610.1 | pectinesterase |
| LOC_Os06g38510.1 | pectate lyase precursor |
| LOC_Os01g10440.1 | xylosyltransferase |
| LOC_Os03g06960.1 | vesicle-associated membrane protein |
| Locus_id | Putative Function |
| LOC_Os01g50810.1 | invertase/pectin methylesterase inhibitor family protein |
| LOC_Os08g34340.1 | DUF593 domain containing protein |
| LOC_Os04g42210.1 | GPI-anchored protein |
| LOC_Os12g12860.1 | CAMK_CAMK_like.46 - CAMK includes calcium/calmodulin depedent protein kinases |
| LOC_Os01g12490.1 | flavin monooxygenase |
| LOC_Os06g45160.1 | pollen allergen |
| LOC_Os03g49270.1 | THION36 - Plant thionin family protein precursor |
| LOC_Os10g27480.1 | expressed protein |
| LOC_Os04g46910.1 | actin-depolymerizing factor |
| LOC_Os08g06450.1 | cytidylyltransferase domain containing protein |
| LOC_Os03g01640.1 | expansin precursor |
| LOC_Os11g04840.1 | phosphatidylinositol-4-phosphate 5-kinase |
| LOC_Os05g46740.1 | regulator of chromosome condensation |
| LOC_Os08g37570.1 | spotted leaf 11 |
| LOC_Os07g34130.1 | TBC domain containing protein |
| LOC_Os06g40570.1 | GRAM and C2 domains containing protein |
| LOC_Os03g61410.1 | expressed protein |
| LOC_Os04g25160.1 | pollen allergen |
| LOC_Os02g13570.1 | kinesin motor protein-related |
| LOC_Os04g11130.1 | DEF9 - Defensin and Defensin-like DEFL family |
| LOC_Os01g46850.1 | expressed protein |
| LOC_Os10g32900.1 | CCT motif family protein |
| LOC_Os01g57940.1 | tyrosine protein kinase domain containing protein |
| LOC_Os03g37120.1 | retrotransposon protein, putative, unclassified |
| LOC_Os03g12180.1 | MA3 domain containing protein |
| LOC_Os01g61390.1 | transposon protein, putative, unclassified |
| Locus_id | Putative Function |
| LOC_Os01g10470.1 | RALFL17 - Rapid ALkalinization Factor RALF family protein precursor |
| LOC_Os01g71240.1 | calcium-transporting ATPase, plasma membrane-type |
| LOC_Os01g13270.1 | AGC_PVPK_like_kin82y.4 - ACG kinases include homologs to PKA, PKG and PKC |
| LOC_Os07g02780.1 | STE_MEKK_ste11_MAP3K.20 |
| LOC_Os01g68620.1 | signal peptide peptidase-like 2B |
| LOC_Os01g25460.1 | uncharacterized protein At4g06744 precursor |
| LOC_Os01g69020.1 | retrotransposon protein, putative, unclassified |
| LOC_Os02g50140.1 | caleosin related protein |
| LOC_Os01g27190.1 | C2 domain containing protein |
| LOC_Os01g13440.1 | pollen allergen Cyn d 23 |
| LOC_Os03g12520.1 | STRUBBELIG-RECEPTOR FAMILY 7 precursor |
| LOC_Os10g08022.1 | fructose-bisphospate aldolase isozyme |
| LOC_Os04g35020.1 | CSLH2 - cellulose synthase-like family H |
| LOC_Os10g33650.1 | CK1_CaseinKinase_1.9 - CK1 includes the casein kinase 1 kinases |
| LOC_Os05g11720.1 | zinc finger, C3HC4 type domain containing protein |
| LOC_Os04g54600.1 | DUF617 domain containing protein |
| LOC_Os06g39060.1 | glucan endo-1,3-beta-glucosidase precursor |
| LOC_Os12g23170.1 | Os12bglu38 - beta-glucosidase/beta-mannosidase/exoglucanase homologue |
| LOC_Os05g51750.1 | aspartyl protease family protein |
| LOC_Os03g59440.1 | dirigent |
| LOC_Os06g40580.1 | expressed protein |
| LOC_Os08g07500.1 | DUF617 domain containing protein |
| LOC_Os05g38770.1 | protein kinase APK1B, chloroplast precursor |
| LOC_Os05g30940.1 | vacuolar calcium binding protein |
| LOC_Os01g39970.1 | protein kinase domain containing protein |
| LOC_Os01g23880.1 | expressed protein |
| Locus_id | Putative Function |
| LOC_Os11g04170.1 | CAMK_CAMK_like.42 - CAMK includes calcium/calmodulin depedent protein kinases |
| LOC_Os03g52870.1 | WD-40 repeat family protein |
| LOC_Os12g42660.1 | AGC_AGC_other_GWLd.1 - ACG kinases include homologs to PKA, PKG and PKC |
| LOC_Os11g20384.1 | SacI homology domain containing protein |
| LOC_Os03g18360.1 | BTBT1 - Bric-a-Brac, Tramtrack, Broad Complex BTB domain with tetratricopeptide repeats |
| LOC_Os03g24170.1 | phosphatidylinositol-4-phosphate 5-kinase |
| LOC_Os06g45200.1 | group 3 pollen allergen |
| LOC_Os04g42960.1 | Lung seven transmembrane receptor domain containing protein |
| LOC_Os09g39950.1 | POEI23 - Pollen Ole e I allergen and extensin family protein precursor |
| LOC_Os07g17120.1 | late embryogenesis abundant protein |
| LOC_Os03g10460.1 | expressed protein |
| LOC_Os06g50140.1 | endoglucanase |
| LOC_Os12g27190.1 | expressed protein |
| LOC_Os07g18070.1 | expressed protein |
| LOC_Os04g52950.1 | nitrate-induced NOI protein |
| LOC_Os12g22920.1 | expressed protein |
| LOC_Os12g42650.1 | pollen preferential protein |
| LOC_Os08g39370.1 | citrate transporter |
| LOC_Os09g12620.1 | GPI-anchored protein |
| LOC_Os11g13860.1 | cyclin-dependent kinase |
| LOC_Os02g49510.1 | amino acid transporter |
| LOC_Os11g22350.1 | white-brown complex homolog protein |
| LOC_Os08g39460.1 | AGC_PVPK_like_CDK8.2 - ACG kinases include homologs to PKA, PKG and PKC |
| LOC_Os03g28090.1 | pectinesterase |
| LOC_Os11g32490.1 | expressed protein |
| LOC_Os10g10760.1 | expressed protein |
| Locus_id | Putative Function |
| LOC_Os05g50170.1 | ATPCME |
| LOC_Os07g10550.1 | cyclin-related protein |
| LOC_Os03g51210.1 | expressed protein |
| LOC_Os04g45010.1 | OsPLIM2b - LIM domain protein, putative actin-binding protein and transcription factor |
| LOC_Os08g12160.1 | expressed protein |
| LOC_Os03g18860.1 | pectinesterase |
| LOC_Os12g41170.1 | SEY1 |
| LOC_Os08g04890.1 | OsCML32 - Calmodulin-related calcium sensor protein |
| LOC_Os12g30550.1 | protein of unknown function domain containing protein |
| LOC_Os08g12890.1 | DNA binding protein |
| LOC_Os03g24510.1 | glycosyl transferase 8 domain containing protein |
| LOC_Os03g51620.1 | DUF221 domain containing protein |
| LOC_Os05g38980.1 | respiratory burst oxidase |
| LOC_Os06g44810.1 | expressed protein |
| LOC_Os06g36240.1 | POEI21 - Pollen Ole e I allergen and extensin family protein precursor |
| LOC_Os02g35760.1 | STRUBBELIG-RECEPTOR FAMILY 7 precursor |
| LOC_Os01g05100.1 | expressed protein |
| LOC_Os08g38200.1 | phosphatidylinositol 3- and 4-kinase family protein |
| LOC_Os08g41650.1 | expressed protein |
| LOC_Os04g51440.1 | villin protein |
| LOC_Os06g27850.1 | peroxidase precursor |
| LOC_Os04g55110.1 | expressed protein |
| LOC_Os09g33910.1 | CAMK_CAMK_like.39 - CAMK includes calcium/calmodulin depedent protein kinases |
| LOC_Os07g17310.1 | B12D protein |
| LOC_Os08g38610.1 | pentatricopeptide |
| LOC_Os05g40230.1 | vacuolar ATP synthase subunit E |
| Locus_id | Putative Function |
| LOC_Os12g43700.1 | SCP-like extracellular protein |
| LOC_Os05g11790.1 | CAMK_KIN1/SNF1/Nim1_like.19 - CAMK includes calcium/calmodulin depedent protein kinases |
| LOC_Os03g10334.1 | expressed protein |
| LOC_Os05g46530.1 | invertase/pectin methylesterase inhibitor family protein |
| LOC_Os05g11330.1 | RALFL19 - Rapid ALkalinization Factor RALF family protein precursor |
| LOC_Os02g58800.1 | expressed protein |
| LOC_Os09g12630.1 | expressed protein |
| LOC_Os03g21880.1 | THION28 - Plant thionin family protein precursor |
| LOC_Os05g33580.1 | expressed protein |
| LOC_Os08g39530.1 | expressed protein |
| LOC_Os02g09450.1 | glycerophosphoryl diester phosphodiesterase family protein |
| LOC_Os05g08540.1 | gibberellin 3-beta-dioxygenase 2-2 |
| LOC_Os02g50770.1 | peroxidase precursor |
| LOC_Os11g40550.1 | receptor kinase |
| LOC_Os07g37480.1 | expressed protein |
| LOC_Os02g01310.1 | invertase/pectin methylesterase inhibitor family protein |
| LOC_Os06g46560.1 | myb-like DNA-binding domain containing protein |
| LOC_Os08g33860.1 | zinc finger, C3HC4 type |
| LOC_Os09g32440.1 | endonuclease/exonuclease/phosphatase family domain containing protein |
| LOC_Os04g01330.1 | expressed protein |
| LOC_Os03g61530.1 | invertase/pectin methylesterase inhibitor family protein |
| LOC_Os05g05640.1 | pectinesterase inhibitor domain containing protein |
| LOC_Os10g26600.1 | soluble inorganic pyrophosphatase |
| LOC_Os04g57750.1 | plastocyanin-like domain containing protein |
| LOC_Os09g30040.1 | expressed protein |
| LOC_Os05g51090.1 | nodulin MtN3 family protein |
| Locus_id | Putative Function |
| LOC_Os02g26290.1 | fasciclin-like arabinogalactan protein 8 precursor |
| LOC_Os02g07900.1 | ANTH/ENTH domain containing protein |
| LOC_Os01g48410.1 | ATROPGEF7/ROPGEF7 |
| LOC_Os02g55500.1 | expressed protein |
| LOC_Os03g62210.1 | subtilisin N-terminal Region family protein |
| LOC_Os06g44470.1 | pollen allergen |
| LOC_Os04g21340.1 | expressed protein |
| LOC_Os05g51230.1 | phosphatidylinositol 3- and 4-kinase family protein |
| LOC_Os02g49350.1 | plastocyanin-like domain containing protein |
| LOC_Os05g24770.1 | reticulon domain containing protein |
| LOC_Os06g35320.1 | polygalacturonase |
| LOC_Os04g33710.1 | expressed protein |
| LOC_Os02g02450.1 | transposon protein, putative, unclassified |
| LOC_Os01g20970.1 | invertase/pectin methylesterase inhibitor family protein |
| LOC_Os04g01150.1 | ELMO/CED-12 family protein |
| LOC_Os02g17240.1 | ATROPGEF7/ROPGEF7 |
| LOC_Os01g68540.1 | rho GDP-dissociation inhibitor 1 |
| LOC_Os01g33300.1 | polygalacturonase |
| LOC_Os10g21110.1 | glycosyl hydrolase family 10 protein |
| LOC_Os11g08680.1 | expressed protein |
| LOC_Os06g36210.1 | amino acid transporter |
| LOC_Os01g51610.1 | B3 DNA binding domain containing protein |
| LOC_Os03g08610.1 | leucine-rich repeat family protein |
| LOC_Os05g13830.1 | TsetseEP precursor |
| LOC_Os01g06620.1 | expressed protein |
| LOC_Os02g04030.1 | phosphatidylinositol transfer |
| Locus_id | Putative Function |
| LOC_Os02g32740.1 | SNARE domain containing protein |
| LOC_Os06g48050.1 | expressed protein |
| LOC_Os11g11710.1 | expressed protein |
| LOC_Os08g34900.1 | pectinesterase |
| LOC_Os11g08707.1 | expressed protein |
| LOC_Os03g04620.1 | CCT motif family protein |
| LOC_Os04g25190.1 | pollen allergen |
| LOC_Os07g26480.1 | P21-Rho-binding domain containing protein |
| LOC_Os02g09540.1 | expressed protein |
| LOC_Os02g24430.1 | expressed protein |
| LOC_Os03g58190.1 | expressed protein |
| LOC_Os03g59350.1 | anthocyanin 3-O-beta-glucosyltransferase |
| LOC_Os05g41270.1 | CAMK_CAMK_like.4 - CAMK includes calcium/calmodulin depedent protein kinases |
| LOC_Os06g17450.1 | expressed protein |
| LOC_Os06g45150.1 | pollen allergen |
| LOC_Os06g49470.1 | peptidyl-prolyl cis-trans isomerase |
| LOC_Os07g39930.1 | expressed protein |
| LOC_Os11g44880.1 | kinesin-4 |
| LOC_Os12g43820.1 | GCRP5 - Glycine and cysteine rich family protein precursor |
| LOC_Os02g03480.1 | THION24 - Plant thionin family protein precursor |
| LOC_Os02g03490.1 | major ampullate spidroin 3 |
| LOC_Os02g05670.1 | expressed protein |
| LOC_Os03g27110.1 | hydrolase protein |
| LOC_Os04g47320.1 | uncharacterized mscS family protein |
| LOC_Os05g40740.1 | monocopper oxidase |
| LOC_Os06g08380.1 | 1,3-beta-glucan synthase component domain containing protein |
| Locus_id | Putative Function |
| LOC_Os06g43060.1 | expressed protein |
| LOC_Os02g49340.1 | nitrate-induced NOI protein |
| LOC_Os11g06690.1 | serine esterase family protein |
| LOC_Os11g36740.1 | DUF593 domain containing protein |
| LOC_Os04g42220.1 | GPI-anchored protein |
| LOC_Os08g38250.1 | skin secretory protein xP2 precursor |
| LOC_Os08g38740.1 | glycosyl transferase 8 domain containing protein |
| LOC_Os09g10740.1 | mitochondrial import inner membrane translocase subunit Tim17 |
| LOC_Os02g10300.1 | polygalacturonase |
| LOC_Os04g41250.1 | armadillo/beta-catenin repeat family protein |
| LOC_Os03g04770.1 | beta-amylase |
| LOC_Os12g32760.1 | transporter family protein |
| LOC_Os07g15530.1 | expressed protein |
| LOC_Os06g02019.1 | cytochrome P450 |
| LOC_Os02g03510.1 | fibroin heavy chain precursor |
| LOC_Os12g35690.1 | RALFL5 - Rapid ALkalinization Factor RALF family protein precursor |
| LOC_Os06g04790.1 | HAD superfamily phosphatase |
| LOC_Os06g04200.1 | starch synthase |
| LOC_Os03g53410.1 | protein kinase domain containing protein |
| LOC_Os02g55649.1 | CXXXC8 - Cysteine-rich protein with paired CXXXC motifs precursor |
| LOC_Os04g57270.1 | expressed protein |
| LOC_Os04g57280.1 | expressed protein |
| LOC_Os05g51900.1 | expressed protein |
| LOC_Os08g44660.1 | EF hand family protein |
| LOC_Os01g55940.1 | OsGH3.2 - Probable indole-3-acetic acid-amido synthetase |
| LOC_Os08g42740.2 | IBR domain containing protein |
| Locus_id | Putative Function |
| LOC_Os09g39930.1 | tyrosine protein kinase domain containing protein |
| LOC_Os10g40090.1 | expansin precursor |
| LOC_Os03g64300.1 | THION30 - Plant thionin family protein precursor |
| LOC_Os03g64310.1 | expressed protein |
| LOC_Os01g10890.1 | CAMK_KIN1/SNF1/Nim1_like.8 - CAMK includes calcium/calmodulin depedent protein kinases |
| LOC_Os02g10630.1 | GRAM and C2 domains containing protein |
| LOC_Os02g17780.1 | ent-kaurene synthase, chloroplast precursor |
| LOC_Os09g23899.1 | pollen allergen Cyn d 23 |
| LOC_Os01g51060.1 | hydrolase |
| LOC_Os04g57350.1 | EH domain-containing protein 1 |
| LOC_Os08g43570.1 | beta-galactosidase precursor |
| LOC_Os04g45290.1 | glycosyl hydrolases |
| LOC_Os03g11140.1 | pleckstrin homology domain-containing protein-related taxo |
| LOC_Os01g42024.1 | expressed protein |
| LOC_Os03g62750.1 | inner membrane protein |
| LOC_Os03g01610.1 | expansin precursor |
| LOC_Os06g03980.1 | expressed protein |
| LOC_Os10g25674.1 | mps one binder kinase activator-like 1A |
| LOC_Os07g13980.1 | glucose-1-phosphate adenylyltransferase large subunit |
| LOC_Os10g35930.1 | OsPLIM2c - LIM domain protein, putative actin-binding protein and transcription factor |
| LOC_Os01g67490.1 | OTU-like cysteine protease family protein |
| LOC_Os06g05160.1 | sulfate transporter |
| LOC_Os10g32810.1 | beta-amylase |
| LOC_Os12g41780.1 | glycosyl transferase family 17 protein |
| LOC_Os01g10900.1 | MRH1 |
| LOC_Os06g48300.1 | protein phosphatase 2C |
| Locus_id | Putative Function |
| LOC_Os03g61590.1 | expressed protein |
| LOC_Os07g31830.1 | GTPase activating protein |
| LOC_Os05g50120.1 | CGMC_MAPKCMGC_2.3 - CGMC includes CDA, MAPK, GSK3, and CLKC kinases |
| LOC_Os04g58100.1 | expressed protein |
| LOC_Os03g25460.1 | OsRCI2-4 - Putative low temperature and salt responsive protein |
| LOC_Os08g34780.1 | adenylyl cyclase-associated protein |
| LOC_Os07g09384.1 | expressed protein |
| LOC_Os02g37580.1 | fimbrin-like protein 2 |
| LOC_Os01g02050.1 | phosphoenolpyruvate carboxylase |
| LOC_Os06g29350.1 | myosin |
| LOC_Os02g53990.1 | cyclin-related protein |
| LOC_Os05g31720.1 | stromal membrane-associated protein |
| LOC_Os05g50110.1 | oleosin |
| LOC_Os03g07600.1 | calcium-binding protein |
| LOC_Os02g44470.1 | actin-depolymerizing factor |
| LOC_Os06g01780.1 | ethylene-responsive element-binding protein |
| LOC_Os05g41150.1 | expressed protein |
| LOC_Os02g05040.1 | cyclin-related protein |
| LOC_Os04g46079.1 | ELMO/CED-12 family protein |
| LOC_Os05g39000.1 | KIP1 |
| LOC_Os02g43840.1 | ethylene-responsive element-binding protein |
| LOC_Os06g40890.1 | polygalacturonase |
| LOC_Os06g10480.1 | expressed protein |
| LOC_Os12g43990.1 | expressed protein |
| LOC_Os01g68900.1 | zinc finger, C3HC4 type family protein |
| LOC_Os06g04450.1 | Sec1 family transport protein |
| Locus_id | Putative Function |
| LOC_Os01g74450.1 | aquaporin protein |
| LOC_Os02g32060.1 | hydrolase, NUDIX family, domain containing protein |
| LOC_Os01g61910.1 | KIP1 |
| LOC_Os07g07420.1 | gibberellin 20 oxidase 1-B |
| LOC_Os03g45920.1 | tubulin/FtsZ domain containing protein |
| LOC_Os07g03200.1 | phytosulfokines precursor |
| LOC_Os02g42710.1 | C2 domain containing protein |
| LOC_Os02g02560.1 | UTP--glucose-1-phosphate uridylyltransferase |
| LOC_Os07g01770.1 | expressed protein |
| LOC_Os09g36810.1 | beta-galactosidase precursor |
| LOC_Os02g38040.1 | leucine-rich repeat family protein |
| LOC_Os03g16900.1 | rab GDP dissociation inhibitor alpha |
| LOC_Os01g21970.1 | protein kinase |
| LOC_Os06g44660.1 | fasciclin-like arabinogalactan precursor protein |
| LOC_Os02g42820.1 | OsPLIM2a - LIM domain protein, putative actin-binding protein and transcription factor |
| LOC_Os07g30640.1 | ubiquitin fusion protein |
| LOC_Os03g17030.1 | polyadenylate-binding protein |
| LOC_Os10g32590.1 | universal stress protein domain containing protein |
| LOC_Os08g32920.1 | dynamin-2B |
| LOC_Os10g20550.1 | DEFL70 - Defensin and Defensin-like DEFL family |
| LOC_Os05g51680.2 | SCP-like extracellular protein |
| LOC_Os01g74510.1 | KIP1 |
| LOC_Os10g26470.1 | sucrose transporter, putativ |
| LOC_Os05g05650.1 | C2 domain containing protein |
| LOC_Os01g72280.1 | tetratricopeptide repeat containing protein |
| LOC_Os12g06840.1 | ATEXO70C2 |
| Locus_id | Putative Function |
| LOC_Os02g03400.1 | microtubule associated protein |
| LOC_Os03g59660.1 | clathrin adaptor complex small chain domain containing protein |
| LOC_Os05g23194.1 | expressed protein |
| LOC_Os11g11730.1 | cell wall adhesin |
| LOC_Os08g06440.1 | secretory carrier-associated membrane protein 5 |
| LOC_Os03g43100.2 | expressed protein |
| LOC_Os05g45370.1 | cell cycle control protein |
| LOC_Os05g07720.1 | alliin lyase precursor |
| LOC_Os04g53330.1 | RNA recognition motif containing protein |
| LOC_Os04g49739.1 | purine permease |
| LOC_Os07g13160.1 | expressed protein |
| LOC_Os03g61510.1 | pectinesterase inhibitor domain containing protein |
| LOC_Os07g14340.1 | retrotransposon protein, putative, unclassified |
| LOC_Os11g09030.1 | expressed protein |
| LOC_Os12g38570.1 | transmembrane amino acid transporter protein |
| LOC_Os02g04210.1 | expressed protein |
| LOC_Os02g01270.1 | START domain containing protein |
| LOC_Os07g48790.1 | SNF1-related protein kinase regulatory subunit beta-1 |
| LOC_Os02g48370.1 | ARID/BRIGHT DNA-binding domain-containing protein |
| LOC_Os06g11920.1 | endonuclease/exonuclease/phosphatase family domain containing protein |
| LOC_Os04g02690.1 | pattern formation protein EMB30 |
| LOC_Os09g21370.1 | cysteine proteinase EP-B 1 precursor |
| LOC_Os01g74480.1 | cupin domain containing protein |
| LOC_Os06g11970.1 | OsMADS63 - MADS-box family gene with MIKC* type-box |
| LOC_Os07g28920.1 | expressed protein |
| LOC_Os12g38260.1 | expressed protein |
| Locus_id | Putative Function |
| LOC_Os07g15680.1 | phospholipase D |
| LOC_Os08g25710.1 | CSLD3 - cellulose synthase-like family D |
| LOC_Os08g35770.1 | expressed protein |
| LOC_Os08g15090.1 | diacylglycerol kinase catalytic domain family protein |
| LOC_Os01g58640.1 | nucleotide pyrophosphatase/phosphodiesterase |
| LOC_Os05g37880.1 | growth regulator related protein |
| LOC_Os11g11240.1 | expressed protein |
| LOC_Os05g41950.1 | protein kinase |
| LOC_Os03g07510.1 | expressed protein |
| LOC_Os05g06670.1 | gibberellin 2-oxidase |
| LOC_Os06g51460.1 | white-brown complex homolog protein |
| LOC_Os01g45620.1 | CGMC_MAPKCMGC_2.5 - CGMC includes CDA, MAPK, GSK3, and CLKC kinases |
| LOC_Os01g38500.1 | DUF538 domain containing protein |
| LOC_Os07g49250.1 | thiamine pyrophosphate enzyme, C-terminal TPP binding domain containing protein |
| LOC_Os02g55990.1 | vesicle-associated membrane protein |
| LOC_Os02g04630.1 | sodium/calcium exchanger protein |
| LOC_Os01g26290.1 | expressed protein |
| LOC_Os12g36630.1 | universal stress protein domain containing protein |
| LOC_Os03g06760.1 | exocyst complex component 6 |
| LOC_Os11g28610.1 | transporter family protein |
| LOC_Os05g20914.1 | expressed protein |
| LOC_Os01g50470.1 | regulator of chromosome condensation |
| LOC_Os05g09440.1 | NADP-dependent malic enzyme, chloroplast precursor |
| LOC_Os02g01590.1 | glycosyl hydrolases |
| LOC_Os03g08110.1 | hydrolase protein |
| LOC_Os02g52840.1 | flavonol synthase/flavanone 3-hydroxylase |
| Locus_id | Putative Function |
| LOC_Os01g23370.1 | expressed protein |
| LOC_Os02g58660.1 | ATCHX15 |
| LOC_Os08g23460.1 | harpin-induced protein 1 domain containing protein |
| LOC_Os03g49590.1 | expressed protein |
| LOC_Os09g09630.1 | WRKY112 |
| LOC_Os09g29990.1 | skin secretory protein xP2 precursor |
| LOC_Os11g05970.1 | pyridine nucleotide-disulphide oxidoreductase family protein |
| LOC_Os01g08570.1 | 2-oxoglutarate and iron-dependent oxygenase domain-containing protein2 |
| LOC_Os02g07580.1 | expressed protein |
| LOC_Os02g41940.1 | retrotransposon protein, putative, unclassified |
| LOC_Os12g07874.1 | WD-40 repeat family protein |
| LOC_Os05g51860.1 | expressed protein |
| LOC_Os06g05710.1 | pollen-specific protein |
| LOC_Os08g28700.1 | dnaJ domain containing protein |
| LOC_Os12g03510.1 | PME/invertase inhibitor |
| LOC_Os12g28260.1 | cyclic nucleotide-gated ion channel |
| LOC_Os01g71040.1 | expressed protein |
| LOC_Os02g33740.1 | GPI-anchored protein |
| LOC_Os01g56764.1 | expressed protein |
| LOC_Os04g30300.1 | retrotransposon protein, putative, unclassified |
| LOC_Os09g28150.1 | bifunctional monodehydroascorbate reductase and carbonic anhydrasenectarin-3 precursor |
| LOC_Os03g09190.1 | OsSCP11 - Putative Serine Carboxypeptidase homologue |
| LOC_Os05g19500.1 | ATCHX |
| LOC_Os03g62410.1 | phospholipase D |
| LOC_Os06g29080.1 | expressed protein |
| LOC_Os05g20620.1 | retrotransposon protein, putative, unclassified |
| Locus_id | Putative Function |
| LOC_Os02g58610.1 | protein kinase |
| LOC_Os05g28530.1 | glutaredoxin |
| LOC_Os05g35160.1 | clathrin assembly protein |
| LOC_Os11g42440.1 | expressed protein |
| LOC_Os01g11800.1 | retrotransposon protein, putative, unclassified |
| LOC_Os01g55850.1 | expressed protein |
| LOC_Os01g60140.1 | ATCHX15 |
| LOC_Os02g57790.1 | ZOS2-19 - C2H2 zinc finger protein |
| LOC_Os04g21570.1 | fasciclin-like arabinogalactan precursor protein |
| LOC_Os06g45180.1 | pollen allergen |
| LOC_Os02g50980.1 | expressed protein |
| LOC_Os11g06700.1 | exo70 exocyst complex subunit |
| LOC_Os12g37480.1 | invertase/pectin methylesterase inhibitor family protein |
| LOC_Os04g10500.1 | expressed protein |
| LOC_Os05g40310.1 | expressed protein |
| LOC_Os06g45240.1 | inactive receptor kinase At2g26730 precursor |
| LOC_Os08g38590.1 | OsMADS62 - MADS-box family gene with MIKC* type-box |
| LOC_Os09g28130.1 | carbonic anhydrase family protein |
| LOC_Os12g32310.1 | expressed protein |
| LOC_Os11g29210.1 | resistance-gene-interacting protein |
| LOC_Os10g27370.1 | transposon protein, putative, Pong sub-class |
| LOC_Os07g13450.1 | RALFL13 - Rapid ALkalinization Factor RALF family protein precursor |
| LOC_Os01g70120.1 | expressed protein |
| LOC_Os01g42060.1 | expressed protein |
| LOC_Os06g03830.1 | retinol dehydrogenase |
| LOC_Os10g01560.1 | serine/threonine-protein kinase |
| Locus_id | Putative Function |
| LOC_Os02g31940.1 | potassium transporter |
| LOC_Os07g30210.1 | integral membrane protein DUF6 domain containing protein |
| LOC_Os03g01930.1 | retrotransposon protein, putative, unclassified |
| LOC_Os10g07970.1 | anthocyanidin 5,3-O-glucosyltransferase |
| LOC_Os12g16180.1 | lipase |
| LOC_Os11g32960.1 | zinc finger DHHC domain-containing protein |
| LOC_Os02g40550.1 | Lung seven transmembrane receptor domain containing protein |
| LOC_Os06g10970.1 | xyloglucan fucosyltransferase |
| LOC_Os07g20270.1 | expressed protein |
| LOC_Os04g54860.1 | glutaredoxin |
| LOC_Os03g60470.1 | glycine-rich protein A3 |
| LOC_Os06g08810.1 | NAD dependent epimerase/dehydratase family protein |
| LOC_Os06g39050.1 | syntaxin |
| LOC_Os11g30350.1 | PB1 domain containing protein |
| LOC_Os05g50830.1 | protein kinase family protein |
| LOC_Os04g40570.1 | ABC transporter, ATP-binding protein |
| LOC_Os08g35440.1 | Ser/Thr protein phosphatase family protein |
| LOC_Os10g27170.1 | calmodulin-binding protein |
| LOC_Os06g09860.1 | expressed protein |
| LOC_Os02g06510.1 | myosin heavy chain |
| LOC_Os02g54100.1 | expressed protein |
| LOC_Os03g09160.1 | hydroxyproline-rich glycoprotein family protein |
| LOC_Os03g27900.1 | ZIM motif family protein |
| LOC_Os05g38790.1 | IQ calmodulin-binding motif domain containing protein |
| LOC_Os07g06800.1 | 3-oxo-5-alpha-steroid 4-dehydrogenase |
| LOC_Os09g27710.1 | pollen allergen Cyn d 23 |
| Locus_id | Putative Function |
| LOC_Os09g28450.1 | paramyosin |
| LOC_Os11g43740.1 | OsMADS68 - MADS-box family gene with MIKC* type-box |
| LOC_Os12g30500.1 | DUF593 domain containing protein |
| LOC_Os03g16420.1 | retrotransposon protein, putative, unclassified |
| LOC_Os02g13580.1 | kinesin motor domain containing protein |
| LOC_Os02g50150.1 | caleosin related protein |
| LOC_Os02g10990.1 | FAD binding domain of DNA photolyase domain containing protein |
| LOC_Os06g45190.1 | pollen allergen |
| LOC_Os08g13980.1 | glycosyl hydrolases family 16 |
| LOC_Os02g45890.1 | sulfotransferase domain containing protein |
| LOC_Os02g51310.1 | TCP family transcription factor |
| LOC_Os04g37520.1 | extracellular ligand-gated ion channel |
| LOC_Os05g07880.1 | phospholipase D |
| LOC_Os05g49580.1 | plastocyanin-like domain containing protein |
| LOC_Os12g38450.1 | exostosin family domain containing protein |
| LOC_Os08g07600.1 | expressed protein |
| LOC_Os11g29780.1 | plant-specific domain TIGR01627 family protein |
| LOC_Os06g45184.1 | retrotransposon protein, putative, Ty3-gypsy subclass |
| LOC_Os01g65860.1 | ANTH domain containing protein |
| LOC_Os01g48540.1 | glyoxal oxidase-related |
| LOC_Os07g07130.1 | expressed protein |
| LOC_Os01g14926.1 | protein kinase domain containing protein |
| LOC_Os01g45350.1 | exostosin family protein |
| LOC_Os10g40140.1 | MSP domain containing protein |
| LOC_Os10g04370.1 | OsFBX357 - F-box domain containing protein |
| LOC_Os10g40170.1 | expressed protein |
| Locus_id | Putative Function |
| LOC_Os10g11750.1 | LTPL89 - Protease inhibitor/seed storage/LTP family protein precursor |
| LOC_Os02g36924.1 | OsMADS27 - MADS-box family gene with MIKCc type-box |
| LOC_Os02g06540.1 | transporter family protein |
| LOC_Os09g09350.1 | expressed protein |
| LOC_Os02g55400.1 | ATPase 8, plasma membrane-type |
| LOC_Os05g10800.1 | expressed protein |
| LOC_Os11g40800.1 | expressed protein |
| LOC_Os03g38390.1 | GDSL-like lipase/acylhydrolase |
| LOC_Os02g51600.1 | endonuclease/exonuclease/phosphatase family domain containing protein |
| LOC_Os09g19950.1 | DUF260 domain containing protein |
| LOC_Os03g13120.1 | expressed protein |
| LOC_Os11g23220.1 | myosin |
| LOC_Os05g39600.1 | ATCHX15 |
| LOC_Os06g48980.1 | protein kinase APK1B, chloroplast precursor |
| LOC_Os11g01820.1 | ATCHX |
| LOC_Os10g34680.1 | vacuolar protein sorting-associated protein 52 |
| LOC_Os06g44900.1 | leaf senescence related protein |
| LOC_Os02g32210.1 | expressed protein |
| LOC_Os02g26320.1 | fasciclin-like arabinogalactan precursor protein |
| LOC_Os12g05730.1 | bifunctional monodehydroascorbate reductase and carbonic anhydrasenectarin-3 precursor |
| LOC_Os11g45720.1 | pectinesterase |
| LOC_Os08g12520.1 | expressed protein |
| LOC_Os09g03890.1 | octicosapeptide/Phox/Bem1p |
| LOC_Os09g25650.1 | GEX2 |
| LOC_Os12g30150.1 | CAMK_CAMK_like.47 - CAMK includes calcium/calmodulin depedent protein kinases |
| LOC_Os01g40150.1 | eukaryotic translation initiation factor 5B |
| Locus_id | Putative Function |
| LOC_Os05g09050.1 | MLO domain containing protein |
| LOC_Os06g51130.1 | spotted leaf 11 |
| LOC_Os10g10700.1 | invertase/pectin methylesterase inhibitor family protein |
| LOC_Os11g37720.1 | integral membrane protein DUF6 domain containing protein |
| LOC_Os12g44300.1 | CHX28 |
| LOC_Os08g08070.1 | transporter family protein |
| LOC_Os09g12510.1 | expressed protein |
| LOC_Os02g44080.1 | aquaporin protein |
| LOC_Os08g40990.1 | receptor-like protein kinase 1 |
| LOC_Os07g01780.1 | GTPase activating protein |
| LOC_Os01g71780.1 | WD domain, G-beta repeat domain containing protein |
| LOC_Os07g49100.1 | pectinesterase |
| LOC_Os04g54850.1 | pectinesterase |
| LOC_Os02g44590.1 | OsSub20 - Putative Subtilisin homologue |
| LOC_Os01g13710.1 | transposon protein, putative, unclassified |
| LOC_Os10g25560.1 | XI-I |
| LOC_Os05g48640.1 | ATROPGEF7/ROPGEF7 |
| LOC_Os12g10190.1 | transposon protein, putative, unclassified |
| LOC_Os03g58140.1 | transmembrane BAX inhibitor motif-containing protein |
| LOC_Os09g37300.1 | transporter, monovalent cation:proton antiporter-2 family |
| LOC_Os04g20420.1 | DNA binding protein |
| LOC_Os12g12730.1 | OsCML28 - Calmodulin-related calcium sensor protein |
| LOC_Os04g46670.1 | ZOS4-09 - C2H2 zinc finger protein |
| LOC_Os05g38960.1 | expressed protein |
| LOC_Os07g08060.1 | transmembrane BAX inhibitor motif-containing protein |
| LOC_Os08g02880.1 | CXXXC11 - Cysteine-rich protein with paired CXXXC motifs precursor |
| Locus_id | Putative Function |
| LOC_Os02g02460.1 | transposon protein, putative, unclassified |
| LOC_Os09g26360.1 | pectinesterase |
| LOC_Os08g45230.1 | ENTH domain containing protein |
| LOC_Os02g03550.1 | glycosyl hydrolases family 16 |
| LOC_Os08g02450.1 | ATCHX |
| LOC_Os02g57000.1 | C2 domain containing protein |
| LOC_Os11g45730.1 | pectinesterase |
| LOC_Os12g37660.1 | pectinesterase |
| LOC_Os06g08310.1 | plasma membrane ATPase |
| LOC_Os05g29740.1 | invertase/pectin methylesterase inhibitor family protein |
| LOC_Os08g31080.1 | DUF260 domain containing protein |
| LOC_Os03g04560.1 | expressed protein |
| LOC_Os07g47120.1 | beta-amylase |
| LOC_Os10g33640.1 | AGC_AGC_other_NDRh_TRCd.3 - ACG kinases include homologs to PKA, PKG and PKC |
| LOC_Os11g16350.1 | expressed protein |
| LOC_Os09g16970.1 | expressed protein |
| LOC_Os01g55520.1 | ATROPGEF7/ROPGEF7 |
| LOC_Os01g55440.1 | CAMK_KIN1/SNF1/Nim1_like.1 - CAMK includes calcium/calmodulin depedent protein kinases |
| LOC_Os03g12570.1 | expressed protein |
| LOC_Os02g12660.1 | protein kinase domain containing protein |
| LOC_Os10g39950.1 | viral A-type inclusion protein repeat containing protein |
| LOC_Os03g17270.1 | expressed protein |
| LOC_Os07g13580.1 | glucan endo-1,3-beta-glucosidase precursor |
| LOC_Os02g58520.1 | CAMK_CAMK_like.16 - CAMK includes calcium/calmodulin depedent protein kinases |
| LOC_Os08g30800.1 | KED |
| LOC_Os07g08000.1 | expressed protein |
| Locus_id | Putative Function |
| LOC_Os11g46230.1 | tetratricopeptide repeat domain containing protein |
| LOC_Os01g09730.1 | expressed protein |
| LOC_Os07g45260.1 | glycosyl transferase 8 domain containing protein |
| LOC_Os03g02740.1 | phospholipase D |
| LOC_Os01g59360.1 | CAMK_CAMK_like.10 - CAMK includes calcium/calmodulin depedent protein kinases |
| LOC_Os01g16470.1 | phosphatidylinositol kinase |
| LOC_Os06g03610.1 | TKL_IRAK_CrRLK1L-1.13 - The CrRLK1L-1 subfamily has homology to the CrRLK1L homolog |
| LOC_Os04g32890.1 | retrotransposon protein, putative, unclassified |
| LOC_Os02g35300.1 | transposon protein, putative, unclassified |
| LOC_Os05g16290.1 | transposon protein, putative, unclassified |
| LOC_Os08g38280.1 | mucin-associated surface protein |
| LOC_Os03g44440.1 | cyclic nucleotide-gated ion channel |
| LOC_Os08g44790.1 | expansin precursor |
| LOC_Os06g05260.1 | pectate lyase precursor |
| LOC_Os01g59880.1 | inositol-1, 4, 5-trisphosphate 5-phosphatase |
| LOC_Os02g46180.1 | DUF581 domain containing protein |
| LOC_Os05g48520.1 | glyoxal oxidase-related |
| LOC_Os05g51660.1 | SCP-like extracellular protein |
| LOC_Os08g04650.1 | pectinesterase inhibitor domain containing protein |
| LOC_Os06g22980.1 | CSLD5 - cellulose synthase-like family D |
| LOC_Os04g59310.1 | phospholipase C |
| LOC_Os04g47150.1 | OsSub43 - Putative Subtilisin homologue |
| LOC_Os02g12300.1 | pectate lyase precursor |
| LOC_Os04g58480.1 | EF hand family protein |
| LOC_Os05g40650.1 | ATCHX |
| LOC_Os02g44120.1 | ZOS2-13 - C2H2 zinc finger protein |
| Locus_id | Putative Function |
| LOC_Os05g14750.1 | AGC_PVPK_like_kin82y.12 - ACG kinases include homologs to PKA, PKG and PKC |
| LOC_Os03g08980.1 | expressed protein |
| LOC_Os03g57510.1 | CAMK_CAMK_like.23 - CAMK includes calcium/calmodulin depedent protein kinases |
| LOC_Os06g43000.1 | nitrate-induced NOI protein |
| LOC_Os06g47110.1 | COBRA-like protein precursor |
| LOC_Os01g14940.1 | invertase/pectin methylesterase inhibitor family protein |
| LOC_Os08g36560.1 | emp24/gp25L/p24 family protein |
| LOC_Os05g51670.1 | NAD dependent epimerase/dehydratase family protein |
| LOC_Os12g36040.1 | expansin precursor |
| LOC_Os09g38700.1 | STRUBBELIG-RECEPTOR FAMILY 5 precursor |
| LOC_Os02g01290.1 | expressed protein |
| LOC_Os11g45220.1 | IWS1 homolog A |
| LOC_Os07g01260.1 | la domain containing protein |
| LOC_Os11g02580.1 | esterase |
| LOC_Os06g05209.1 | pectate lyase precursor |
| LOC_Os11g36230.1 | expressed protein |
| LOC_Os05g40190.1 | thioredoxin |
| LOC_Os08g43500.1 | armadillo/beta-catenin repeat family protein |
| LOC_Os11g08400.1 | expressed protein |
| LOC_Os06g44160.1 | heat shock protein DnaJ |
| LOC_Os04g51830.1 | OsHKT1;4 - Na+ transporter |
| LOC_Os01g12680.2 | C4-dicarboxylate transporter/malic acid transport protein |
| LOC_Os02g38170.1 | expressed protein |
| LOC_Os03g44630.1 | plastocyanin-like domain containing protein |
| LOC_Os02g05820.1 | protein kinase domain containing protein |
| LOC_Os09g27040.1 | GEX1 |
| Locus_id | Putative Function |
| LOC_Os12g06570.1 | cyclic nucleotide-gated ion channel |
| LOC_Os04g29050.1 | expressed protein |
| LOC_Os07g30160.1 | trehalose phosphatase |
| LOC_Os05g35050.1 | ctr copper transporter family protein |
| LOC_Os03g53550.1 | retrotransposon protein, putative, unclassified |
| LOC_Os10g42390.1 | zinc finger, C3HC4 type domain containing protein |
| LOC_Os11g05950.1 | expressed protein |
| LOC_Os03g17150.1 | ZOS3-09 - C2H2 zinc finger protein |
| LOC_Os05g45810.1 | calcineurin B |
| LOC_Os03g51990.1 | ACT domain containing protein |
| LOC_Os01g11350.1 | bZIP transcription factor domain containing protein |
| LOC_Os02g36950.1 | uncharacterized Cys-rich domain containing protein |
| LOC_Os04g38560.1 | pectinesterase |
| LOC_Os12g35710.1 | uncharacterized protein At4g06744 precursor |
| LOC_Os06g05730.1 | expressed protein |
| LOC_Os10g39020.1 | fringe-related protein |
| LOC_Os05g40180.1 | serine/threonine-protein kinase stt7, chloroplast precursor |
| LOC_Os09g22000.1 | hydrolase, HAD superfamily, Cof family |
| LOC_Os02g54590.1 | serine threonine kinase |
| LOC_Os02g10530.1 | expressed protein |
| LOC_Os03g27610.1 | patatin |
| LOC_Os04g46490.1 | aquaporin protein |
| LOC_Os02g26390.1 | expressed protein |
| LOC_Os08g01324.1 | expressed protein |
| LOC_Os08g23130.1 | oligopeptide transporter 5 |
| LOC_Os04g40810.1 | dihydrolipoamide acetyltransferase |
| Locus_id | Putative Function |
| LOC_Os01g66860.1 | ankyrin-kinase |
| LOC_Os07g30090.1 | actin-depolymerizing factor |
| LOC_Os06g09230.1 | serine threonine kinase |
| LOC_Os05g28710.1 | expressed protein |
| LOC_Os11g08698.1 | expressed protein |

**Table S3.** Locus IDs and promoter regions of genes used for promoter analysis with *GUS* reporter.

| Locus ID | Putative function | Promoter size |
| --- | --- | --- |
|  |  | (location used in vector) |
| LOC_Os11g45730 | Pectinesterase | 1,520 |
|  |  | (-1,815 to -295) |
| LOC_Os02g50770 | Plant peroxidase family protein | 963 |
|  |  | (-1,038 to -75) |
| LOC_Os01g69020 | Cell division protein FtsZ family protein | 1,779 |
|  |  | (-1,868 to -89) |
| LOC_Os05g46530 | Plant invertase/pectin methylesterase inhibitor domain containing protein | 1,784 |
|  |  | (-1,928 to -144) |
| LOC_Os07g14340 | Pectinesterase inhibitor domain containing protein | 1,634 |
|  |  | (-1,711 to -77) |
| LOC_Os04g25190 | Pollen allergen Lol p2 family protein | 1,498 |
|  |  | (-1,570 to-72) |

**Table S4.** Classification of GO terms for biological processes associated with late pollen-preferred genes.

| Locus ID | RGAP Ver 6 Annotation | GO ID | GO Name |
| --- | --- | --- | --- |
| LOC_Os01g02050 | phosphoenolpyruvate carboxylase | GO:0006099 | tricarboxylic acid cycle |
| LOC_Os01g08570 | 2-oxoglutarate and iron-dependent oxygenase domain-containing protein2 | GO:0055114 | oxidation reduction |
| LOC_Os01g10890 | CAMK_KIN1/SNF1/Nim1_like.8 | GO:0006468 | protein amino acid phosphorylation |
| LOC_Os01g10890 | CAMK_KIN1/SNF1/Nim1_like.8 | GO:0007165 | signal transduction |
| LOC_Os01g10900 | MRH1 | GO:0006468 | protein amino acid phosphorylation |
| LOC_Os01g11350 | bZIP transcription factor domain containing protein | GO:0006355 | regulation of transcription, DNA-dependent |
| LOC_Os01g12490 | flavin monooxygenase | GO:0007186 | G-protein coupled receptor protein signaling pathway |
| LOC_Os01g13270 | AGC_PVPK_like_kin82y.4 - ACG kinases include homologs to PKA, PKG and PKC | GO:0006468 | protein amino acid phosphorylation |
| LOC_Os01g13440 | pollen allergen Cyn d 23 | GO:0016068 | type I hypersensitivity |
| LOC_Os01g13710 | transposon protein, unclassified | GO:0009405 | pathogenesis |
| LOC_Os01g14926 | protein kinase domain containing protein | GO:0006468 | protein amino acid phosphorylation |
| LOC_Os01g21970 | protein kinase | GO:0006468 | protein amino acid phosphorylation |
| LOC_Os01g25460 | uncharacterized protein At4g06744 precursor | GO:0007186 | G-protein coupled receptor protein signaling pathway |
| LOC_Os01g27190 | C2 domain containing protein | GO:0051260 | protein homooligomerization |
| LOC_Os01g33300 | polygalacturonase | GO:0005975 | carbohydrate metabolic process |
| LOC_Os01g33300 | polygalacturonase | GO:0008152 | metabolic process |
| LOC_Os01g39970 | protein kinase domain containing protein | GO:0006950 | response to stress |
| LOC_Os01g39970 | protein kinase domain containing protein | GO:0006468 | protein amino acid phosphorylation |
| LOC_Os01g45620 | CGMC_MAPKCMGC_2.5 - CGMC includes CDA, MAPK, GSK3, and CLKC kinases | GO:0006468 | protein amino acid phosphorylation |
| LOC_Os01g51060 | hydrolase | GO:0006508 | proteolysis |
| LOC_Os01g51610 | B3 DNA binding domain containing protein | GO:0006355 | regulation of transcription, DNA-dependent |
| LOC_Os01g55440 | CAMK_KIN1/SNF1/Nim1_like.1 | GO:0006468 | protein amino acid phosphorylation |
| LOC_Os01g55440 | CAMK_KIN1/SNF1/Nim1_like.1 | GO:0007165 | signal transduction |
| Locus ID | RGAP Ver 6 Annotation | GO ID | GO Name |
| LOC_Os01g55940 | OsGH3.2 - Probable indole-3-acetic acid-amido synthetase | GO:0009416 | response to light stimulus |
| LOC_Os01g55940 | OsGH3.2 - Probable indole-3-acetic acid-amido synthetase | GO:0009733 | response to auxin stimulus |
| LOC_Os01g55940 | OsGH3.2 - Probable indole-3-acetic acid-amido synthetase | GO:0032940 | secretion by cell |
| LOC_Os01g57940 | tyrosine protein kinase domain containing protein | GO:0006468 | protein amino acid phosphorylation |
| LOC_Os01g59360 | CAMK_CAMK_like.10 | GO:0005978 | glycogen biosynthetic process |
| LOC_Os01g59360 | CAMK_CAMK_like.10 | GO:0006468 | protein amino acid phosphorylation |
| LOC_Os01g60140 | ATCHX15 | GO:0006812 | cation transport |
| LOC_Os01g61390 | transposon protein, unclassified | GO:0006810 | transport |
| LOC_Os01g65860 | ANTH domain containing protein | GO:0048268 | clathrin coat assembly |
| LOC_Os01g66860 | serine/threonine protein kinase | GO:0006468 | protein amino acid phosphorylation |
| LOC_Os01g66860 | serine/threonine protein kinase | GO:0009966 | regulation of signal transduction |
| LOC_Os01g68620 | signal peptide peptidase-like 2B | GO:0006835 | dicarboxylic acid transport |
| LOC_Os01g69240 | CBS domain containing membrane protein | GO:0030036 | actin cytoskeleton organization |
| LOC_Os01g71240 | calcium-transporting ATPase, plasma membrane-type | GO:0006754 | ATP biosynthetic process |
| LOC_Os01g71240 | calcium-transporting ATPase, plasma membrane-type | GO:0006812 | cation transport |
| LOC_Os01g71240 | calcium-transporting ATPase, plasma membrane-type | GO:0006816 | calcium ion transport |
| LOC_Os01g71240 | calcium-transporting ATPase, plasma membrane-type | GO:0008152 | metabolic process |
| LOC_Os01g74450 | aquaporin protein | GO:0006810 | transport |
| LOC_Os02g01590 | glycosyl hydrolases | GO:0005975 | carbohydrate metabolic process |
| LOC_Os02g01590 | glycosyl hydrolases | GO:0008152 | metabolic process |
| LOC_Os02g02450 | transposon protein, unclassified | GO:0006810 | transport |
| LOC_Os02g02460 | transposon protein, unclassified | GO:0006810 | transport |
| LOC_Os02g02560 | UTP--glucose-1-phosphate uridylyltransferase | GO:0006508 | proteolysis |
| LOC_Os02g02560 | UTP--glucose-1-phosphate uridylyltransferase | GO:0008152 | metabolic process |
| LOC_Os02g03480 | THION24 - Plant thionin family protein precursor | GO:0006412 | translation |
| LOC_Os02g03480 | THION24 - Plant thionin family protein precursor | GO:0007186 | G-protein coupled receptor protein signaling pathway |
| Locus ID | RGAP Ver 6 Annotation | GO ID | GO Name |
| LOC_Os02g03480 | THION24 - Plant thionin family protein precursor | GO:0016068 | type I hypersensitivity |
| LOC_Os02g03480 | THION24 - Plant thionin family protein precursor | GO:0042309 | homoiothermy |
| LOC_Os02g03480 | THION24 - Plant thionin family protein precursor | GO:0050826 | response to freezing |
| LOC_Os02g03490 | major ampullate spidroin 3 | GO:0050826 | response to freezing |
| LOC_Os02g03490 | major ampullate spidroin 3 | GO:0042309 | homoiothermy |
| LOC_Os02g03510 | fibroin heavy chain precursor | GO:0006412 | translation |
| LOC_Os02g03510 | fibroin heavy chain precursor | GO:0042309 | homoiothermy |
| LOC_Os02g03510 | fibroin heavy chain precursor | GO:0050826 | response to freezing |
| LOC_Os02g03550 | glycosyl hydrolases family 16 | GO:0005975 | carbohydrate metabolic process |
| LOC_Os02g03550 | glycosyl hydrolases family 16 | GO:0006073 | cellular glucan metabolic process |
| LOC_Os02g04030 | phosphatidylinositol transfer | GO:0006810 | transport |
| LOC_Os02g05040 | cyclin-related protein | GO:0009405 | pathogenesis |
| LOC_Os02g05820 | protein kinase domain containing protein | GO:0006950 | response to stress |
| LOC_Os02g05820 | protein kinase domain containing protein | GO:0006468 | protein amino acid phosphorylation |
| LOC_Os02g06540 | transporter family protein | GO:0006810 | transport |
| LOC_Os02g06540 | transporter family protein | GO:0055085 | transmembrane transport |
| LOC_Os02g07900 | ANTH/ENTH domain containing protein | GO:0048268 | clathrin coat assembly |
| LOC_Os02g09450 | glycerophosphoryl diester phosphodiesterase family protein | GO:0006071 | glycerol metabolic process |
| LOC_Os02g09450 | glycerophosphoryl diester phosphodiesterase family protein | GO:0006629 | lipid metabolic process |
| LOC_Os02g10300 | polygalacturonase | GO:0050826 | response to freezing |
| LOC_Os02g10300 | polygalacturonase | GO:0042309 | homoiothermy |
| LOC_Os02g10300 | polygalacturonase | GO:0008152 | metabolic process |
| LOC_Os02g10300 | polygalacturonase | GO:0007047 | cellular cell wall organization |
| LOC_Os02g10300 | polygalacturonase | GO:0005975 | carbohydrate metabolic process |
| LOC_Os02g10990 | FAD binding domain of DNA photolyase domain containing protein | GO:0006281 | DNA repair |
| LOC_Os02g10990 | FAD binding domain of DNA photolyase domain containing protein | GO:0042309 | homoiothermy |
| Locus ID | RGAP Ver 6 Annotation | GO ID | GO Name |
| LOC_Os02g10990 | FAD binding domain of DNA photolyase domain containing protein | GO:0050826 | response to freezing |
| LOC_Os02g12660 | protein kinase domain containing protein | GO:0006468 | protein amino acid phosphorylation |
| LOC_Os02g12660 | protein kinase domain containing protein | GO:0006950 | response to stress |
| LOC_Os02g13570 | kinesin motor protein-related | GO:0007018 | microtubule-based movement |
| LOC_Os02g13580 | kinesin motor domain containing protein | GO:0007018 | microtubule-based movement |
| LOC_Os02g17780 | ent-kaurene synthase, chloroplast precursor | GO:0008152 | metabolic process |
| LOC_Os02g17780 | ent-kaurene synthase, chloroplast precursor | GO:0009685 | gibberellin metabolic process |
| LOC_Os02g17780 | ent-kaurene synthase, chloroplast precursor | GO:0051504 | diterpene phytoalexin precursor biosynthetic process pathway |
| LOC_Os02g24430 | expressed protein | GO:0006810 | transport |
| LOC_Os02g31940 | potassium transporter | GO:0006810 | transport |
| LOC_Os02g31940 | potassium transporter | GO:0006811 | ion transport |
| LOC_Os02g31940 | potassium transporter | GO:0006813 | potassium ion transport |
| LOC_Os02g35300 | transposon protein, unclassified | GO:0006468 | protein amino acid phosphorylation |
| LOC_Os02g35760 | STRUBBELIG-RECEPTOR FAMILY 7 precursor | GO:0006468 | protein amino acid phosphorylation |
| LOC_Os02g36924 | OsMADS27 - MADS-box family gene with MIKCc type-box | GO:0006350 | transcription |
| LOC_Os02g36924 | OsMADS27 - MADS-box family gene with MIKCc type-box | GO:0006355 | regulation of transcription, DNA-dependent |
| LOC_Os02g36924 | OsMADS27 - MADS-box family gene with MIKCc type-box | GO:0045449 | regulation of transcription |
| LOC_Os02g37580 | fimbrin-like protein 2 | GO:0008152 | metabolic process |
| LOC_Os02g44080 | aquaporin protein | GO:0006810 | transport |
| LOC_Os02g44590 | OsSub20 - Putative Subtilisin homologue | GO:0006508 | proteolysis |
| LOC_Os02g44590 | OsSub20 - Putative Subtilisin homologue | GO:0043086 | negative regulation of catalytic activity |
| LOC_Os02g49510 | amino acid transporter | GO:0006865 | amino acid transport |
| LOC_Os02g50770 | peroxidase precursor | GO:0006979 | response to oxidative stress |
| LOC_Os02g50770 | peroxidase precursor | GO:0055114 | oxidation reduction |
| LOC_Os02g54590 | serine threonine kinase | GO:0006468 | protein amino acid phosphorylation |
| LOC_Os02g54590 | serine threonine kinase | GO:0006950 | response to stress |
| Locus ID | RGAP Ver 6 Annotation | GO ID | GO Name |
| LOC_Os02g55400 | ATPase 8, plasma membrane-type | GO:0006754 | ATP biosynthetic process |
| LOC_Os02g55400 | ATPase 8, plasma membrane-type | GO:0006812 | cation transport |
| LOC_Os02g55400 | ATPase 8, plasma membrane-type | GO:0008152 | metabolic process |
| LOC_Os02g55500 | expressed protein | GO:0008150 | biological_process |
| LOC_Os02g55990 | vesicle-associated membrane protein | GO:0006810 | transport |
| LOC_Os02g55990 | vesicle-associated membrane protein | GO:0016192 | vesicle-mediated transport |
| LOC_Os02g57790 | ZOS2-19 - C2H2 zinc finger protein | GO:0006355 | regulation of transcription, DNA-dependent |
| LOC_Os02g57790 | ZOS2-19 - C2H2 zinc finger protein | GO:0006813 | potassium ion transport |
| LOC_Os02g58520 | CAMK_CAMK_like.16 | GO:0006468 | protein amino acid phosphorylation |
| LOC_Os02g58610 | protein kinase | GO:0006468 | protein amino acid phosphorylation |
| LOC_Os02g58660 | ATCHX15 | GO:0006810 | transport |
| LOC_Os02g58660 | ATCHX15 | GO:0006812 | cation transport |
| LOC_Os03g01610 | expansin precursor | GO:0019953 | sexual reproduction |
| LOC_Os03g01610 | expansin precursor | GO:0007047 | cellular cell wall organization |
| LOC_Os03g01640 | expansin precursor | GO:0007047 | cellular cell wall organization |
| LOC_Os03g01640 | expansin precursor | GO:0019953 | sexual reproduction |
| LOC_Os03g02740 | phospholipase D | GO:0008152 | metabolic process |
| LOC_Os03g02740 | phospholipase D | GO:0046470 | phosphatidylcholine metabolic process |
| LOC_Os03g04770 | beta-amylase | GO:0050826 | response to freezing |
| LOC_Os03g04770 | beta-amylase | GO:0042309 | homoiothermy |
| LOC_Os03g04770 | beta-amylase | GO:0008152 | metabolic process |
| LOC_Os03g04770 | beta-amylase | GO:0005975 | carbohydrate metabolic process |
| LOC_Os03g04770 | beta-amylase | GO:0000272 | polysaccharide catabolic process |
| LOC_Os03g05770 | peroxidase precursor | GO:0006979 | response to oxidative stress |
| LOC_Os03g05770 | peroxidase precursor | GO:0055114 | oxidation reduction |
| LOC_Os03g06760 | exocyst complex component 6 | GO:0006904 | vesicle docking involved in exocytosis |
| Locus ID | RGAP Ver 6 Annotation | GO ID | GO Name |
| LOC_Os03g06960 | vesicle-associated membrane protein | GO:0006810 | transport |
| LOC_Os03g06960 | vesicle-associated membrane protein | GO:0016192 | vesicle-mediated transport |
| LOC_Os03g07510 | expressed protein | GO:0045449 | regulation of transcription |
| LOC_Os03g09190 | OsSCP11 - Putative Serine Carboxypeptidase homologue | GO:0006508 | proteolysis |
| LOC_Os03g11140 | pleckstrin homology domain-containing protein-related taxo | GO:0007165 | signal transduction |
| LOC_Os03g12520 | STRUBBELIG-RECEPTOR FAMILY 7 precursor | GO:0006468 | protein amino acid phosphorylation |
| LOC_Os03g12570 | expressed protein | GO:0006306 | DNA methylation |
| LOC_Os03g12570 | expressed protein | GO:0006333 | chromatin assembly or disassembly |
| LOC_Os03g12570 | expressed protein | GO:0006468 | protein amino acid phosphorylation |
| LOC_Os03g16900 | rab GDP dissociation inhibitor alpha | GO:0043087 | regulation of GTPase activity |
| LOC_Os03g16900 | rab GDP dissociation inhibitor alpha | GO:0015031 | protein transport |
| LOC_Os03g18860 | pectinesterase | GO:0042545 | cell wall modification |
| LOC_Os03g19610 | pectinesterase | GO:0042545 | cell wall modification |
| LOC_Os03g21880 | THION28 - Plant thionin family protein precursor | GO:0042309 | homoiothermy |
| LOC_Os03g21880 | THION28 - Plant thionin family protein precursor | GO:0050826 | response to freezing |
| LOC_Os03g24160 | phosphatidylinositol-4-phosphate 5-kinase | GO:0046488 | phosphatidylinositol metabolic process |
| LOC_Os03g24160 | phosphatidylinositol-4-phosphate 5-kinase | GO:0007186 | G-protein coupled receptor protein signaling pathway |
| LOC_Os03g24170 | phosphatidylinositol-4-phosphate 5-kinase | GO:0046488 | phosphatidylinositol metabolic process |
| LOC_Os03g24510 | glycosyl transferase 8 domain containing protein | GO:0008152 | metabolic process |
| LOC_Os03g27610 | patatin | GO:0016042 | lipid catabolic process |
| LOC_Os03g27610 | patatin | GO:0008152 | metabolic process |
| LOC_Os03g27610 | patatin | GO:0006629 | lipid metabolic process |
| LOC_Os03g28090 | pectinesterase | GO:0042545 | cell wall modification |
| LOC_Os03g37120 | retrotransposon protein, unclassified | GO:0006468 | protein amino acid phosphorylation |
| LOC_Os03g37120 | retrotransposon protein, unclassified | GO:0007155 | cell adhesion |
| LOC_Os03g37120 | retrotransposon protein, unclassified | GO:0007186 | G-protein coupled receptor protein signaling pathway |
| Locus ID | RGAP Ver 6 Annotation | GO ID | GO Name |
| LOC_Os03g38390 | GDSL-like lipase/acylhydrolase | GO:0006629 | lipid metabolic process |
| LOC_Os03g44440 | cyclic nucleotide-gated ion channel | GO:0006813 | potassium ion transport |
| LOC_Os03g44440 | cyclic nucleotide-gated ion channel | GO:0006811 | ion transport |
| LOC_Os03g44440 | cyclic nucleotide-gated ion channel | GO:0006810 | transport |
| LOC_Os03g45920 | tubulin/FtsZ domain containing protein | GO:0007017 | microtubule-based process |
| LOC_Os03g45920 | tubulin/FtsZ domain containing protein | GO:0007018 | microtubule-based movement |
| LOC_Os03g45920 | tubulin/FtsZ domain containing protein | GO:0051258 | protein polymerization |
| LOC_Os03g51990 | ACT domain containing protein | GO:0008152 | metabolic process |
| LOC_Os03g53410 | protein kinase domain containing protein | GO:0006412 | translation |
| LOC_Os03g53410 | protein kinase domain containing protein | GO:0006468 | protein amino acid phosphorylation |
| LOC_Os03g57510 | CAMK_CAMK_like.23 | GO:0006468 | protein amino acid phosphorylation |
| LOC_Os03g59350 | anthocyanin 3-O-beta-glucosyltransferase | GO:0008152 | metabolic process |
| LOC_Os03g59660 | clathrin adaptor complex small chain domain containing protein | GO:0006810 | transport |
| LOC_Os03g59660 | clathrin adaptor complex small chain domain containing protein | GO:0006886 | intracellular protein transport |
| LOC_Os03g59660 | clathrin adaptor complex small chain domain containing protein | GO:0015031 | protein transport |
| LOC_Os03g59660 | clathrin adaptor complex small chain domain containing protein | GO:0016192 | vesicle-mediated transport |
| LOC_Os03g60470 | glycine-rich protein A3 | GO:0007186 | G-protein coupled receptor protein signaling pathway |
| LOC_Os03g60470 | glycine-rich protein A3 | GO:0051260 | protein homooligomerization |
| LOC_Os03g61510 | pectinesterase inhibitor domain containing protein | GO:0009306 | protein secretion |
| LOC_Os03g62210 | subtilisin N-terminal Region family protein | GO:0043086 | negative regulation of catalytic activity |
| LOC_Os03g62410 | phospholipase D | GO:0008152 | metabolic process |
| LOC_Os03g62410 | phospholipase D | GO:0046470 | phosphatidylcholine metabolic process |
| LOC_Os03g62750 | inner membrane protein | GO:0051205 | protein insertion into membrane |
| LOC_Os04g01150 | ELMO/CED-12 family protein | GO:0006909 | phagocytosis |
| LOC_Os04g02690 | pattern formation protein EMB30 | GO:0032012 | regulation of ARF protein signal transduction |
| LOC_Os04g11130 | DEF9 - Defensin and Defensin-like DEFL family | GO:0006952 | defense response |
| Locus ID | RGAP Ver 6 Annotation | GO ID | GO Name |
| LOC_Os04g30300 | retrotransposon protein, unclassified | GO:0006278 | RNA-dependent DNA replication |
| LOC_Os04g32890 | retrotransposon protein, unclassified | GO:0006313 | transposition, DNA-mediated |
| LOC_Os04g35020 | CSLH2 - cellulose synthase-like family H | GO:0007047 | cellular cell wall organization |
| LOC_Os04g35020 | CSLH2 - cellulose synthase-like family H | GO:0030244 | cellulose biosynthetic process |
| LOC_Os04g37460 | glutamate decarboxylase | GO:0006536 | glutamate metabolic process |
| LOC_Os04g37460 | glutamate decarboxylase | GO:0019752 | carboxylic acid metabolic process |
| LOC_Os04g38560 | pectinesterase | GO:0042545 | cell wall modification |
| LOC_Os04g40570 | ABC transporter, ATP-binding protein | GO:0006412 | translation |
| LOC_Os04g40570 | ABC transporter, ATP-binding protein | GO:0006810 | transport |
| LOC_Os04g40810 | conserved hypothetical protein | GO:0016068 | type I hypersensitivity |
| LOC_Os04g40810 | conserved hypothetical protein | GO:0006355 | regulation of transcription, DNA-dependent |
| LOC_Os04g41250 | armadillo/beta-catenin repeat family protein | GO:0016567 | protein ubiquitination |
| LOC_Os04g45290 | glycosyl hydrolases | GO:0005975 | carbohydrate metabolic process |
| LOC_Os04g45290 | glycosyl hydrolases | GO:0008152 | metabolic process |
| LOC_Os04g46079 | ELMO/CED-12 family protein | GO:0006909 | phagocytosis |
| LOC_Os04g46490 | aquaporin protein | GO:0006810 | transport |
| LOC_Os04g47150 | OsSub43 - Putative Subtilisin homologue | GO:0006508 | proteolysis |
| LOC_Os04g47150 | OsSub43 - Putative Subtilisin homologue | GO:0043086 | negative regulation of catalytic activity |
| LOC_Os04g51440 | villin protein | GO:0007010 | cytoskeleton organization |
| LOC_Os04g51830 | OsHKT1;4 - Na+ transporter | GO:0006810 | transport |
| LOC_Os04g51830 | OsHKT1;4 - Na+ transporter | GO:0006811 | ion transport |
| LOC_Os04g51830 | OsHKT1;4 - Na+ transporter | GO:0006812 | cation transport |
| LOC_Os04g52950 | nitrate-induced NOI protein | GO:0006334 | nucleosome assembly |
| LOC_Os04g53330 | RNA recognition motif containing protein | GO:0055114 | oxidation reduction |
| LOC_Os04g54850 | pectinesterase | GO:0042545 | cell wall modification |
| LOC_Os04g54860 | glutaredoxin | GO:0045454 | cell redox homeostasis |
| Locus ID | RGAP Ver 6 Annotation | GO ID | GO Name |
| LOC_Os04g54860 | glutaredoxin | GO:0006270 | DNA-dependent DNA replication initiation |
| LOC_Os04g55110 | expressed protein | GO:0015979 | photosynthesis |
| LOC_Os04g59310 | phospholipase C | GO:0006355 | regulation of transcription, DNA-dependent |
| LOC_Os04g59310 | phospholipase C | GO:0006629 | lipid metabolic process |
| LOC_Os04g59310 | phospholipase C | GO:0023034 | intracellular signaling pathway |
| LOC_Os05g06670 | gibberellin 2-oxidase | GO:0009685 | gibberellin metabolic process |
| LOC_Os05g06670 | gibberellin 2-oxidase | GO:0017000 | antibiotic biosynthetic process |
| LOC_Os05g07880 | phospholipase D | GO:0046470 | phosphatidylcholine metabolic process |
| LOC_Os05g07880 | phospholipase D | GO:0008152 | metabolic process |
| LOC_Os05g08540 | gibberellin 3-beta-dioxygenase 2-2 | GO:0009685 | gibberellin metabolic process |
| LOC_Os05g08540 | gibberellin 3-beta-dioxygenase 2-2 | GO:0017000 | antibiotic biosynthetic process |
| LOC_Os05g09050 | MLO domain containing protein | GO:0008219 | cell death |
| LOC_Os05g09440 | NADP-dependent malic enzyme, chloroplast precursor | GO:0006108 | malate metabolic process |
| LOC_Os05g09440 | NADP-dependent malic enzyme, chloroplast precursor | GO:0008152 | metabolic process |
| LOC_Os05g09440 | NADP-dependent malic enzyme, chloroplast precursor | GO:0055114 | oxidation reduction |
| LOC_Os05g10800 | expressed protein | GO:0016068 | type I hypersensitivity |
| LOC_Os05g11790 | CAMK_KIN1/SNF1/Nim1_like.19 | GO:0007165 | signal transduction |
| LOC_Os05g11790 | CAMK_KIN1/SNF1/Nim1_like.19 | GO:0006468 | protein amino acid phosphorylation |
| LOC_Os05g13830 | TsetseEP precursor | GO:0006334 | nucleosome assembly |
| LOC_Os05g13830 | TsetseEP precursor | GO:0006355 | regulation of transcription, DNA-dependent |
| LOC_Os05g14750 | AGC_PVPK_like_kin82y.12 | GO:0006468 | protein amino acid phosphorylation |
| LOC_Os05g14750 | AGC_PVPK_like_kin82y.12 | GO:0006508 | proteolysis |
| LOC_Os05g16290 | transposon protein, unclassified | GO:0006810 | transport |
| LOC_Os05g19500 | ATCHX | GO:0006821 | chloride transport |
| LOC_Os05g19500 | ATCHX | GO:0006812 | cation transport |
| LOC_Os05g19500 | ATCHX | GO:0006810 | transport |
| Locus ID | RGAP Ver 6 Annotation | GO ID | GO Name |
| LOC_Os05g20150 | TKL_IRAK_CrRLK1L-1.11 | GO:0006468 | protein amino acid phosphorylation |
| LOC_Os05g28530 | glutaredoxin | GO:0045454 | cell redox homeostasis |
| LOC_Os05g29740 | invertase/pectin methylesterase inhibitor family protein | GO:0042309 | homoiothermy |
| LOC_Os05g29740 | invertase/pectin methylesterase inhibitor family protein | GO:0050826 | response to freezing |
| LOC_Os05g31720 | stromal membrane-associated protein | GO:0032312 | regulation of ARF GTPase activity |
| LOC_Os05g33580 | expressed protein | GO:0042309 | homoiothermy |
| LOC_Os05g33580 | expressed protein | GO:0050826 | response to freezing |
| LOC_Os05g35050 | ctr copper transporter family protein | GO:0006825 | copper ion transport |
| LOC_Os05g35160 | clathrin assembly protein | GO:0048268 | clathrin coat assembly |
| LOC_Os05g38770 | protein kinase APK1B, chloroplast precursor | GO:0006468 | protein amino acid phosphorylation |
| LOC_Os05g38790 | IQ calmodulin-binding motif domain containing protein | GO:0042309 | homoiothermy |
| LOC_Os05g38790 | IQ calmodulin-binding motif domain containing protein | GO:0050826 | response to freezing |
| LOC_Os05g38980 | respiratory burst oxidase | GO:0055114 | oxidation reduction |
| LOC_Os05g39600 | ATCHX15 | GO:0006810 | transport |
| LOC_Os05g39600 | ATCHX15 | GO:0006812 | cation transport |
| LOC_Os05g40180 | serine/threonine-protein kinase stt7, chloroplast precursor | GO:0006468 | protein amino acid phosphorylation |
| LOC_Os05g40190 | thioredoxin | GO:0045454 | cell redox homeostasis |
| LOC_Os05g40230 | vacuolar ATP synthase subunit E | GO:0015986 | ATP synthesis coupled proton transport |
| LOC_Os05g40650 | ATCHX | GO:0006508 | proteolysis |
| LOC_Os05g40650 | ATCHX | GO:0006810 | transport |
| LOC_Os05g40650 | ATCHX | GO:0006812 | cation transport |
| LOC_Os05g41270 | CAMK_CAMK_like.4 | GO:0006468 | protein amino acid phosphorylation |
| LOC_Os05g41950 | protein kinase | GO:0006468 | protein amino acid phosphorylation |
| LOC_Os05g50120 | CGMC_MAPKCMGC_2.3 | GO:0006468 | protein amino acid phosphorylation |
| LOC_Os05g50830 | protein kinase family protein | GO:0006468 | protein amino acid phosphorylation |
| LOC_Os05g51090 | nodulin MtN3 family protein | GO:0006813 | potassium ion transport |
| Locus ID | RGAP Ver 6 Annotation | GO ID | GO Name |
| LOC_Os05g51090 | nodulin MtN3 family protein | GO:0006810 | transport |
| LOC_Os05g51670 | NAD dependent epimerase/dehydratase family protein | GO:0005975 | carbohydrate metabolic process |
| LOC_Os05g51670 | NAD dependent epimerase/dehydratase family protein | GO:0006012 | galactose metabolic process |
| LOC_Os05g51670 | NAD dependent epimerase/dehydratase family protein | GO:0006694 | steroid biosynthetic process |
| LOC_Os05g51670 | NAD dependent epimerase/dehydratase family protein | GO:0008152 | metabolic process |
| LOC_Os05g51670 | NAD dependent epimerase/dehydratase family protein | GO:0009058 | biosynthetic process |
| LOC_Os05g51670 | NAD dependent epimerase/dehydratase family protein | GO:0044237 | cellular metabolic process |
| LOC_Os05g51670 | NAD dependent epimerase/dehydratase family protein | GO:0045226 | extracellular polysaccharide biosynthetic process |
| LOC_Os05g51750 | aspartyl protease family protein | GO:0006508 | proteolysis |
| LOC_Os05g51750 | aspartyl protease family protein | GO:0005975 | carbohydrate metabolic process |
| LOC_Os06g02019 | cytochrome P450 | GO:0007275 | multicellular organismal development |
| LOC_Os06g02019 | cytochrome P450 | GO:0009685 | gibberellin metabolic process |
| LOC_Os06g02019 | cytochrome P450 | GO:0022900 | electron transport chain |
| LOC_Os06g02019 | cytochrome P450 | GO:0032940 | secretion by cell |
| LOC_Os06g02019 | cytochrome P450 | GO:0055114 | oxidation reduction |
| LOC_Os06g03610 | TKL_IRAK_CrRLK1L-1.13 | GO:0008152 | metabolic process |
| LOC_Os06g03610 | TKL_IRAK_CrRLK1L-1.13 | GO:0006468 | protein amino acid phosphorylation |
| LOC_Os06g03830 | retinol dehydrogenase | GO:0008152 | metabolic process |
| LOC_Os06g03830 | retinol dehydrogenase | GO:0009239 | enterobactin biosynthetic process |
| LOC_Os06g03830 | retinol dehydrogenase | GO:0055114 | oxidation reduction |
| LOC_Os06g04200 | starch synthase | GO:0019252 | starch biosynthetic process |
| LOC_Os06g04200 | starch synthase | GO:0009250 | glucan biosynthetic process |
| LOC_Os06g04200 | starch synthase | GO:0009058 | biosynthetic process |
| LOC_Os06g04200 | starch synthase | GO:0005982 | starch metabolic process |
| LOC_Os06g04450 | Sec1 family transport protein | GO:0016192 | vesicle-mediated transport |
| LOC_Os06g04450 | Sec1 family transport protein | GO:0015031 | protein transport |
| Locus ID | RGAP Ver 6 Annotation | GO ID | GO Name |
| LOC_Os06g04450 | Sec1 family transport protein | GO:0006904 | vesicle docking involved in exocytosis |
| LOC_Os06g04450 | Sec1 family transport protein | GO:0006810 | transport |
| LOC_Os06g05160 | sulfate transporter | GO:0006810 | transport |
| LOC_Os06g05160 | sulfate transporter | GO:0008272 | sulfate transport |
| LOC_Os06g05710 | pollen-specific protein | GO:0042309 | homoiothermy |
| LOC_Os06g05710 | pollen-specific protein | GO:0050826 | response to freezing |
| LOC_Os06g08310 | plasma membrane ATPase | GO:0008152 | metabolic process |
| LOC_Os06g08310 | plasma membrane ATPase | GO:0006812 | cation transport |
| LOC_Os06g08310 | plasma membrane ATPase | GO:0006754 | ATP biosynthetic process |
| LOC_Os06g08380 | 1,3-beta-glucan synthase component domain containing protein | GO:0006075 | 1,3-beta-glucan biosynthetic process |
| LOC_Os06g08810 | NAD dependent epimerase/dehydratase family protein | GO:0045226 | extracellular polysaccharide biosynthetic process |
| LOC_Os06g08810 | NAD dependent epimerase/dehydratase family protein | GO:0044237 | cellular metabolic process |
| LOC_Os06g08810 | NAD dependent epimerase/dehydratase family protein | GO:0009058 | biosynthetic process |
| LOC_Os06g08810 | NAD dependent epimerase/dehydratase family protein | GO:0008152 | metabolic process |
| LOC_Os06g08810 | NAD dependent epimerase/dehydratase family protein | GO:0006694 | steroid biosynthetic process |
| LOC_Os06g08810 | NAD dependent epimerase/dehydratase family protein | GO:0005975 | carbohydrate metabolic process |
| LOC_Os06g09230 | serine threonine kinase | GO:0006468 | protein amino acid phosphorylation |
| LOC_Os06g09230 | serine threonine kinase | GO:0006950 | response to stress |
| LOC_Os06g09860 | expressed protein | GO:0006265 | DNA topological change |
| LOC_Os06g09860 | expressed protein | GO:0006468 | protein amino acid phosphorylation |
| LOC_Os06g10970 | xyloglucan fucosyltransferase | GO:0042546 | cell wall biogenesis |
| LOC_Os06g11970 | OsMADS63 - MADS-box family gene with MIKC* type-box | GO:0045449 | regulation of transcription |
| LOC_Os06g11970 | OsMADS63 - MADS-box family gene with MIKC* type-box | GO:0006355 | regulation of transcription, DNA-dependent |
| LOC_Os06g11970 | OsMADS63 - MADS-box family gene with MIKC* type-box | GO:0006350 | transcription |
| LOC_Os06g17450 | expressed protein | GO:0042309 | homoiothermy |
| LOC_Os06g17450 | expressed protein | GO:0050826 | response to freezing |
| Locus ID | RGAP Ver 6 Annotation | GO ID | GO Name |
| LOC_Os06g22980 | CSLD5 - cellulose synthase-like family D | GO:0007047 | cellular cell wall organization |
| LOC_Os06g22980 | CSLD5 - cellulose synthase-like family D | GO:0030244 | cellulose biosynthetic process |
| LOC_Os06g27850 | peroxidase precursor | GO:0006979 | response to oxidative stress |
| LOC_Os06g27850 | peroxidase precursor | GO:0055114 | oxidation reduction |
| LOC_Os06g29080 | expressed protein | GO:0006468 | protein amino acid phosphorylation |
| LOC_Os06g35320 | polygalacturonase | GO:0008152 | metabolic process |
| LOC_Os06g35320 | polygalacturonase | GO:0007047 | cellular cell wall organization |
| LOC_Os06g35320 | polygalacturonase | GO:0005975 | carbohydrate metabolic process |
| LOC_Os06g39050 | syntaxin | GO:0006886 | intracellular protein transport |
| LOC_Os06g39050 | syntaxin | GO:0016192 | vesicle-mediated transport |
| LOC_Os06g39060 | glucan endo-1,3-beta-glucosidase precursor | GO:0005975 | carbohydrate metabolic process |
| LOC_Os06g39060 | glucan endo-1,3-beta-glucosidase precursor | GO:0008152 | metabolic process |
| LOC_Os06g40570 | GRAM and C2 domains containing protein | GO:0048015 | phosphoinositide-mediated signaling |
| LOC_Os06g40570 | GRAM and C2 domains containing protein | GO:0019882 | antigen processing and presentation |
| LOC_Os06g40570 | GRAM and C2 domains containing protein | GO:0019722 | calcium-mediated signaling |
| LOC_Os06g40570 | GRAM and C2 domains containing protein | GO:0010152 | pollen maturation |
| LOC_Os06g40570 | GRAM and C2 domains containing protein | GO:0006955 | immune response |
| LOC_Os06g40580 | expressed protein | GO:0042309 | homoiothermy |
| LOC_Os06g40580 | expressed protein | GO:0050826 | response to freezing |
| LOC_Os06g40890 | polygalacturonase | GO:0005975 | carbohydrate metabolic process |
| LOC_Os06g40890 | polygalacturonase | GO:0007047 | cellular cell wall organization |
| LOC_Os06g40890 | polygalacturonase | GO:0008152 | metabolic process |
| LOC_Os06g44160 | heat shock protein DnaJ | GO:0006457 | protein folding |
| LOC_Os06g45240 | inactive receptor kinase At2g26730 precursor | GO:0006468 | protein amino acid phosphorylation |
| LOC_Os06g46560 | myb-like DNA-binding domain containing protein | GO:0006813 | potassium ion transport |
| LOC_Os06g46560 | myb-like DNA-binding domain containing protein | GO:0008152 | metabolic process |
| Locus ID | RGAP Ver 6 Annotation | GO ID | GO Name |
| LOC_Os06g46560 | myb-like DNA-binding domain containing protein | GO:0045449 | regulation of transcription |
| LOC_Os06g48050 | expressed protein | GO:0042309 | homoiothermy |
| LOC_Os06g48050 | expressed protein | GO:0050826 | response to freezing |
| LOC_Os06g48300 | protein phosphatase 2C | GO:0006470 | protein amino acid dephosphorylation |
| LOC_Os06g48980 | protein kinase APK1B, chloroplast precursor | GO:0050826 | response to freezing |
| LOC_Os06g48980 | protein kinase APK1B, chloroplast precursor | GO:0042309 | homoiothermy |
| LOC_Os06g48980 | protein kinase APK1B, chloroplast precursor | GO:0006468 | protein amino acid phosphorylation |
| LOC_Os06g49470 | peptidyl-prolyl cis-trans isomerase | GO:0006334 | nucleosome assembly |
| LOC_Os06g49470 | peptidyl-prolyl cis-trans isomerase | GO:0006457 | protein folding |
| LOC_Os06g50140 | endoglucanase | GO:0000272 | polysaccharide catabolic process |
| LOC_Os06g50140 | endoglucanase | GO:0005975 | carbohydrate metabolic process |
| LOC_Os06g50140 | endoglucanase | GO:0007047 | cellular cell wall organization |
| LOC_Os06g50140 | endoglucanase | GO:0008152 | metabolic process |
| LOC_Os06g50140 | endoglucanase | GO:0030245 | cellulose catabolic process |
| LOC_Os06g51130 | spotted leaf 11 | GO:0016567 | protein ubiquitination |
| LOC_Os07g01260 | la domain containing protein | GO:0006396 | RNA processing |
| LOC_Os07g01770 | expressed protein | GO:0005975 | carbohydrate metabolic process |
| LOC_Os07g02780 | STE_MEKK_ste11_MAP3K.20 | GO:0006468 | protein amino acid phosphorylation |
| LOC_Os07g03200 | phytosulfokines precursor | GO:0007275 | multicellular organismal development |
| LOC_Os07g03200 | phytosulfokines precursor | GO:0008283 | cell proliferation |
| LOC_Os07g03200 | phytosulfokines precursor | GO:0030154 | cell differentiation |
| LOC_Os07g03200 | phytosulfokines precursor | GO:0042309 | homoiothermy |
| LOC_Os07g03200 | phytosulfokines precursor | GO:0050826 | response to freezing |
| LOC_Os07g06800 | 3-oxo-5-alpha-steroid 4-dehydrogenase | GO:0006629 | lipid metabolic process |
| LOC_Os07g07420 | gibberellin 20 oxidase 1-B | GO:0009685 | gibberellin metabolic process |
| LOC_Os07g07420 | gibberellin 20 oxidase 1-B | GO:0017000 | antibiotic biosynthetic process |
| Locus ID | RGAP Ver 6 Annotation | GO ID | GO Name |
| LOC_Os07g08000 | expressed protein | GO:0006468 | protein amino acid phosphorylation |
| LOC_Os07g13160 | expressed protein | GO:0051260 | protein homooligomerization |
| LOC_Os07g13580 | glucan endo-1,3-beta-glucosidase precursor | GO:0005975 | carbohydrate metabolic process |
| LOC_Os07g13980 | glucose-1-phosphate adenylyltransferase large subunit | GO:0050826 | response to freezing |
| LOC_Os07g13980 | glucose-1-phosphate adenylyltransferase large subunit | GO:0042309 | homoiothermy |
| LOC_Os07g13980 | glucose-1-phosphate adenylyltransferase large subunit | GO:0019252 | starch biosynthetic process |
| LOC_Os07g13980 | glucose-1-phosphate adenylyltransferase large subunit | GO:0009058 | biosynthetic process |
| LOC_Os07g13980 | glucose-1-phosphate adenylyltransferase large subunit | GO:0005978 | glycogen biosynthetic process |
| LOC_Os07g14340 | retrotransposon protein, unclassified | GO:0006412 | translation |
| LOC_Os07g14340 | retrotransposon protein, unclassified | GO:0006418 | tRNA aminoacylation for protein translation |
| LOC_Os07g15680 | phospholipase D | GO:0008152 | metabolic process |
| LOC_Os07g15680 | phospholipase D | GO:0046470 | phosphatidylcholine metabolic process |
| LOC_Os07g17120 | late embryogenesis abundant protein | GO:0009269 | response to desiccation |
| LOC_Os07g30160 | trehalose phosphatase | GO:0008152 | metabolic process |
| LOC_Os07g30160 | trehalose phosphatase | GO:0005992 | trehalose biosynthetic process |
| LOC_Os07g30210 | integral membrane protein DUF6 domain containing protein | GO:0015904 | tetracycline transport |
| LOC_Os07g30210 | integral membrane protein DUF6 domain containing protein | GO:0046677 | response to antibiotic |
| LOC_Os07g30640 | ubiquitin fusion protein | GO:0006412 | translation |
| LOC_Os07g34130 | TBC domain containing protein | GO:0032313 | regulation of Rab GTPase activity |
| LOC_Os07g47120 | beta-amylase | GO:0000272 | polysaccharide catabolic process |
| LOC_Os07g47120 | beta-amylase | GO:0005975 | carbohydrate metabolic process |
| LOC_Os07g49100 | pectinesterase | GO:0042545 | cell wall modification |
| LOC_Os08g02450 | ATCHX | GO:0006812 | cation transport |
| LOC_Os08g02450 | ATCHX | GO:0006810 | transport |
| LOC_Os08g04890 | OsCML32 - Calmodulin-related calcium sensor protein | GO:0001539 | ciliary or flagellar motility |
| LOC_Os08g06440 | secretory carrier-associated membrane protein 5 | GO:0015031 | protein transport |
| Locus ID | RGAP Ver 6 Annotation | GO ID | GO Name |
| LOC_Os08g06450 | cytidylyltransferase domain containing protein | GO:0009058 | biosynthetic process |
| LOC_Os08g08070 | transporter family protein | GO:0006810 | transport |
| LOC_Os08g08070 | transporter family protein | GO:0055085 | transmembrane transport |
| LOC_Os08g12160 | expressed protein | GO:0008150 | biological_process |
| LOC_Os08g12520 | expressed protein | GO:0007155 | cell adhesion |
| LOC_Os08g13980 | glycosyl hydrolases family 16 | GO:0006073 | cellular glucan metabolic process |
| LOC_Os08g13980 | glycosyl hydrolases family 16 | GO:0005975 | carbohydrate metabolic process |
| LOC_Os08g15090 | diacylglycerol kinase catalytic domain family protein | GO:0007205 | activation of protein kinase C activity by G-protein coupled receptor protein signaling pathway |
| LOC_Os08g25710 | CSLD3 - cellulose synthase-like family D | GO:0030244 | cellulose biosynthetic process |
| LOC_Os08g28700 | dnaJ domain containing protein | GO:0006457 | protein folding |
| LOC_Os08g28700 | dnaJ domain containing protein | GO:0006950 | response to stress |
| LOC_Os08g32920 | dynamin-2B | GO:0007018 | microtubule-based movement |
| LOC_Os08g34340 | DUF593 domain containing protein | GO:0045449 | regulation of transcription |
| LOC_Os08g34340 | DUF593 domain containing protein | GO:0006355 | regulation of transcription, DNA-dependent |
| LOC_Os08g34340 | DUF593 domain containing protein | GO:0006350 | transcription |
| LOC_Os08g34780 | adenylyl cyclase-associated protein | GO:0007010 | cytoskeleton organization |
| LOC_Os08g34900 | pectinesterase | GO:0042545 | cell wall modification |
| LOC_Os08g36560 | emp24/gp25L/p24 family protein | GO:0006810 | transport |
| LOC_Os08g37570 | spotted leaf 11 | GO:0016567 | protein ubiquitination |
| LOC_Os08g38250 | skin secretory protein xP2 precursor | GO:0006355 | regulation of transcription, DNA-dependent |
| LOC_Os08g38250 | skin secretory protein xP2 precursor | GO:0042309 | homoiothermy |
| LOC_Os08g38250 | skin secretory protein xP2 precursor | GO:0050826 | response to freezing |
| LOC_Os08g38590 | OsMADS62 - MADS-box family gene with MIKC* type-box | GO:0045449 | regulation of transcription |
| LOC_Os08g38590 | OsMADS62 - MADS-box family gene with MIKC* type-box | GO:0006355 | regulation of transcription, DNA-dependent |
| LOC_Os08g38590 | OsMADS62 - MADS-box family gene with MIKC* type-box | GO:0006350 | transcription |
| LOC_Os08g38610 | pentatricopeptide | GO:0008152 | metabolic process |
| Locus ID | RGAP Ver 6 Annotation | GO ID | GO Name |
| LOC_Os08g39370 | citrate transporter | GO:0006814 | sodium ion transport |
| LOC_Os08g39370 | citrate transporter | GO:0006835 | dicarboxylic acid transport |
| LOC_Os08g39370 | citrate transporter | GO:0015746 | citrate transport |
| LOC_Os08g39460 | AGC_PVPK_like_CDK8.2 | GO:0006468 | protein amino acid phosphorylation |
| LOC_Os08g39530 | hypothetical protein | GO:0006955 | immune response |
| LOC_Os08g40990 | receptor-like protein kinase 1 | GO:0006468 | protein amino acid phosphorylation |
| LOC_Os08g43570 | beta-galactosidase precursor | GO:0008152 | metabolic process |
| LOC_Os08g43570 | beta-galactosidase precursor | GO:0005975 | carbohydrate metabolic process |
| LOC_Os08g44790 | expansin precursor | GO:0007047 | cellular cell wall organization |
| LOC_Os08g44790 | expansin precursor | GO:0009664 | plant-type cell wall organization |
| LOC_Os08g44790 | expansin precursor | GO:0019953 | sexual reproduction |
| LOC_Os09g09630 | WRKY DNA-binding domain containing protein | GO:0006350 | transcription |
| LOC_Os09g09630 | WRKY DNA-binding domain containing protein | GO:0045449 | regulation of transcription |
| LOC_Os09g10740 | mitochondrial import inner membrane translocase subunit Tim17 | GO:0015031 | protein transport |
| LOC_Os09g12620 | GPI-anchored protein | GO:0050826 | response to freezing |
| LOC_Os09g12620 | GPI-anchored protein | GO:0042309 | homoiothermy |
| LOC_Os09g19950 | DUF260 domain containing protein | GO:0006355 | regulation of transcription, DNA-dependent |
| LOC_Os09g21370 | cysteine proteinase EP-B 1 precursor | GO:0006508 | proteolysis |
| LOC_Os09g22000 | hydrolase, HAD superfamily, Cof family | GO:0006754 | ATP biosynthetic process |
| LOC_Os09g22000 | hydrolase, HAD superfamily, Cof family | GO:0008152 | metabolic process |
| LOC_Os09g23899 | pollen allergen Cyn d 23 | GO:0008150 | biological_process |
| LOC_Os09g26360 | pectinesterase | GO:0042545 | cell wall modification |
| LOC_Os09g27040 | GEX1 | GO:0006935 | chemotaxis |
| LOC_Os09g27040 | GEX1 | GO:0007165 | signal transduction |
| LOC_Os09g28130 | carbonic anhydrase family protein | GO:0006730 | one-carbon metabolic process |
| LOC_Os09g28150 | monodehydroascorbate reductase and carbonic anhydrasenectarin-3 | GO:0006730 | one-carbon metabolic process |
| Locus ID | RGAP Ver 6 Annotation | GO ID | GO Name |
| LOC_Os09g29990 | skin secretory protein xP2 precursor | GO:0016068 | type I hypersensitivity |
| LOC_Os09g29990 | skin secretory protein xP2 precursor | GO:0042309 | homoiothermy |
| LOC_Os09g29990 | skin secretory protein xP2 precursor | GO:0050826 | response to freezing |
| LOC_Os09g33910 | CAMK_CAMK_like.39 - CAMK includes calcium/calmodulin depedent protein kinases | GO:0006468 | protein amino acid phosphorylation |
| LOC_Os09g36810 | beta-galactosidase precursor | GO:0005975 | carbohydrate metabolic process |
| LOC_Os09g36810 | beta-galactosidase precursor | GO:0008152 | metabolic process |
| LOC_Os09g37300 | transporter, monovalent cation:proton antiporter-2 family | GO:0006812 | cation transport |
| LOC_Os09g37300 | transporter, monovalent cation:proton antiporter-2 family | GO:0006810 | transport |
| LOC_Os09g38700 | STRUBBELIG-RECEPTOR FAMILY 5 precursor | GO:0006468 | protein amino acid phosphorylation |
| LOC_Os09g39930 | tyrosine protein kinase domain containing protein | GO:0006468 | protein amino acid phosphorylation |
| LOC_Os10g01560 | serine/threonine-protein kinase | GO:0006468 | protein amino acid phosphorylation |
| LOC_Os10g07970 | anthocyanidin 5,3-O-glucosyltransferase | GO:0006813 | potassium ion transport |
| LOC_Os10g07970 | anthocyanidin 5,3-O-glucosyltransferase | GO:0008152 | metabolic process |
| LOC_Os10g08022 | fructose-bisphospate aldolase isozyme | GO:0006096 | glycolysis |
| LOC_Os10g08022 | fructose-bisphospate aldolase isozyme | GO:0008152 | metabolic process |
| LOC_Os10g11750 | LTPL89 - Protease inhibitor/seed storage/LTP family protein precursor | GO:0050826 | response to freezing |
| LOC_Os10g11750 | LTPL89 - Protease inhibitor/seed storage/LTP family protein precursor | GO:0042309 | homoiothermy |
| LOC_Os10g17660 | profilin domain containing protein | GO:0007010 | cytoskeleton organization |
| LOC_Os10g17660 | profilin domain containing protein | GO:0030036 | actin cytoskeleton organization |
| LOC_Os10g21110 | glycosyl hydrolase family 10 protein | GO:0005975 | carbohydrate metabolic process |
| LOC_Os10g21110 | glycosyl hydrolase family 10 protein | GO:0045493 | xylan catabolic process |
| LOC_Os10g26470 | sucrose transporter, putativ | GO:0007186 | G-protein coupled receptor protein signaling pathway |
| LOC_Os10g26470 | sucrose transporter, putativ | GO:0015770 | sucrose transport |
| LOC_Os10g26600 | soluble inorganic pyrophosphatase | GO:0006796 | phosphate metabolic process |
| LOC_Os10g32590 | universal stress protein domain containing protein | GO:0006950 | response to stress |
| LOC_Os10g32810 | beta-amylase | GO:0000272 | polysaccharide catabolic process |
| Locus ID | RGAP Ver 6 Annotation | GO ID | GO Name |
| LOC_Os10g32810 | beta-amylase | GO:0005975 | carbohydrate metabolic process |
| LOC_Os10g32810 | beta-amylase | GO:0008152 | metabolic process |
| LOC_Os10g32810 | beta-amylase | GO:0042309 | homoiothermy |
| LOC_Os10g32810 | beta-amylase | GO:0050826 | response to freezing |
| LOC_Os10g33640 | AGC_AGC_other_NDRh_TRCd.3 | GO:0006468 | protein amino acid phosphorylation |
| LOC_Os10g33650 | CK1_CaseinKinase_1.9 - CK1 includes the casein kinase 1 kinases | GO:0006468 | protein amino acid phosphorylation |
| LOC_Os10g39950 | viral A-type inclusion protein repeat containing protein | GO:0007165 | signal transduction |
| LOC_Os10g40090 | expansin precursor | GO:0007047 | cellular cell wall organization |
| LOC_Os10g40090 | expansin precursor | GO:0019953 | sexual reproduction |
| LOC_Os11g01820 | ATCHX | GO:0006810 | transport |
| LOC_Os11g01820 | ATCHX | GO:0006812 | cation transport |
| LOC_Os11g04170 | CAMK_CAMK_like.42 | GO:0006468 | protein amino acid phosphorylation |
| LOC_Os11g04840 | phosphatidylinositol-4-phosphate 5-kinase | GO:0046488 | phosphatidylinositol metabolic process |
| LOC_Os11g05970 | pyridine nucleotide-disulphide oxidoreductase family protein | GO:0006813 | potassium ion transport |
| LOC_Os11g06700 | exo70 exocyst complex subunit | GO:0006887 | exocytosis |
| LOC_Os11g11710 | expressed protein | GO:0042309 | homoiothermy |
| LOC_Os11g11710 | expressed protein | GO:0050826 | response to freezing |
| LOC_Os11g11730 | cell wall adhesin | GO:0016068 | type I hypersensitivity |
| LOC_Os11g11730 | cell wall adhesin | GO:0042309 | homoiothermy |
| LOC_Os11g11730 | cell wall adhesin | GO:0050826 | response to freezing |
| LOC_Os11g13860 | cyclin-dependent kinase | GO:0006468 | protein amino acid phosphorylation |
| LOC_Os11g16350 | expressed protein | GO:0050826 | response to freezing |
| LOC_Os11g16350 | expressed protein | GO:0042309 | homoiothermy |
| LOC_Os11g16350 | expressed protein | GO:0016068 | type I hypersensitivity |
| LOC_Os11g28610 | transporter family protein | GO:0006810 | transport |
| LOC_Os11g28610 | transporter family protein | GO:0055085 | transmembrane transport |
| Locus ID | RGAP Ver 6 Annotation | GO ID | GO Name |
| LOC_Os11g32490 | expressed protein | GO:0006835 | dicarboxylic acid transport |
| LOC_Os11g32960 | zinc finger DHHC domain-containing protein | GO:0007275 | multicellular organismal development |
| LOC_Os11g40550 | receptor kinase | GO:0006468 | protein amino acid phosphorylation |
| LOC_Os11g40550 | receptor kinase | GO:0042309 | homoiothermy |
| LOC_Os11g40550 | receptor kinase | GO:0050826 | response to freezing |
| LOC_Os11g42440 | expressed protein | GO:0006468 | protein amino acid phosphorylation |
| LOC_Os11g43740 | OsMADS68 - MADS-box family gene with MIKC* type-box | GO:0006350 | transcription |
| LOC_Os11g43740 | OsMADS68 - MADS-box family gene with MIKC* type-box | GO:0006355 | regulation of transcription, DNA-dependent |
| LOC_Os11g43740 | OsMADS68 - MADS-box family gene with MIKC* type-box | GO:0045449 | regulation of transcription |
| LOC_Os11g44880 | kinesin-4 | GO:0007018 | microtubule-based movement |
| LOC_Os11g45720 | pectinesterase | GO:0042545 | cell wall modification |
| LOC_Os11g45730 | pectinesterase | GO:0042545 | cell wall modification |
| LOC_Os12g05730 | monodehydroascorbate reductase and carbonic anhydrasenectarin-3 | GO:0006730 | one-carbon metabolic process |
| LOC_Os12g06570 | cyclic nucleotide-gated ion channel | GO:0006813 | potassium ion transport |
| LOC_Os12g06570 | cyclic nucleotide-gated ion channel | GO:0006811 | ion transport |
| LOC_Os12g06570 | cyclic nucleotide-gated ion channel | GO:0006810 | transport |
| LOC_Os12g06840 | ATEXO70C2 | GO:0006887 | exocytosis |
| LOC_Os12g07700 | nifU | GO:0016226 | iron-sulfur cluster assembly |
| LOC_Os12g07874 | WD-40 repeat family protein | GO:0006412 | translation |
| LOC_Os12g07874 | WD-40 repeat family protein | GO:0016068 | type I hypersensitivity |
| LOC_Os12g07874 | WD-40 repeat family protein | GO:0042309 | homoiothermy |
| LOC_Os12g07874 | WD-40 repeat family protein | GO:0050826 | response to freezing |
| LOC_Os12g10190 | transposon protein, unclassified | GO:0006468 | protein amino acid phosphorylation |
| LOC_Os12g12730 | OsCML28 - Calmodulin-related calcium sensor protein | GO:0001539 | ciliary or flagellar motility |
| LOC_Os12g12860 | CAMK_CAMK_like.46 | GO:0005978 | glycogen biosynthetic process |
| LOC_Os12g12860 | CAMK_CAMK_like.46 | GO:0006468 | protein amino acid phosphorylation |
| Locus ID | RGAP Ver 6 Annotation | GO ID | GO Name |
| LOC_Os12g23170 | Os12bglu38 - beta-glucosidase/beta-mannosidase/exoglucanase homologue | GO:0005975 | carbohydrate metabolic process |
| LOC_Os12g28260 | cyclic nucleotide-gated ion channel | GO:0006810 | transport |
| LOC_Os12g28260 | cyclic nucleotide-gated ion channel | GO:0006811 | ion transport |
| LOC_Os12g28260 | cyclic nucleotide-gated ion channel | GO:0006813 | potassium ion transport |
| LOC_Os12g30150 | CAMK_CAMK_like.47 | GO:0007186 | G-protein coupled receptor protein signaling pathway |
| LOC_Os12g30150 | CAMK_CAMK_like.47 | GO:0006468 | protein amino acid phosphorylation |
| LOC_Os12g30500 | DUF593 domain containing protein | GO:0006457 | protein folding |
| LOC_Os12g30500 | DUF593 domain containing protein | GO:0015031 | protein transport |
| LOC_Os12g32760 | transporter family protein | GO:0006810 | transport |
| LOC_Os12g32760 | transporter family protein | GO:0008643 | carbohydrate transport |
| LOC_Os12g32760 | transporter family protein | GO:0055085 | transmembrane transport |
| LOC_Os12g35710 | uncharacterized protein At4g06744 precursor | GO:0055114 | oxidation reduction |
| LOC_Os12g35710 | uncharacterized protein At4g06744 precursor | GO:0050826 | response to freezing |
| LOC_Os12g35710 | uncharacterized protein At4g06744 precursor | GO:0042309 | homoiothermy |
| LOC_Os12g35710 | uncharacterized protein At4g06744 precursor | GO:0006725 | cellular aromatic compound metabolic process |
| LOC_Os12g36040 | expansin precursor | GO:0007047 | cellular cell wall organization |
| LOC_Os12g36040 | expansin precursor | GO:0009664 | plant-type cell wall organization |
| LOC_Os12g36040 | expansin precursor | GO:0051260 | protein homooligomerization |
| LOC_Os12g36630 | universal stress protein domain containing protein | GO:0006950 | response to stress |
| LOC_Os12g37480 | invertase/pectin methylesterase inhibitor family protein | GO:0050826 | response to freezing |
| LOC_Os12g37480 | invertase/pectin methylesterase inhibitor family protein | GO:0042309 | homoiothermy |
| LOC_Os12g37480 | invertase/pectin methylesterase inhibitor family protein | GO:0006355 | regulation of transcription, DNA-dependent |
| LOC_Os12g37660 | pectinesterase | GO:0042545 | cell wall modification |
| LOC_Os12g38260 | expressed protein | GO:0005975 | carbohydrate metabolic process |
| LOC_Os12g41780 | glycosyl transferase family 17 protein | GO:0006487 | protein amino acid N-linked glycosylation |
| LOC_Os12g42660 | AGC_AGC_other_GWLd.1 | GO:0006468 | protein amino acid phosphorylation |
| Locus ID | RGAP Ver 6 Annotation | GO ID | GO Name |
| LOC_Os12g44300 | CHX28 | GO:0006355 | regulation of transcription, DNA-dependent |
| LOC_Os12g44300 | CHX28 | GO:0006810 | transport |
| LOC_Os12g44300 | CHX28 | GO:0006812 | cation transport |
| LOC_Os12g44300 | CHX28 | GO:0015986 | ATP synthesis coupled proton transport |
| LOC_Os04g46910 | actin-depolymerizing factor |  | unmapped by GO annotation |
| LOC_Os02g44470 | actin-depolymerizing factor |  | unmapped by GO annotation |
| LOC_Os07g30090 | actin-depolymerizing factor |  | unmapped by GO annotation |
| LOC_Os05g07720 | alliin lyase precursor |  | unmapped by GO annotation |
| LOC_Os06g36210 | amino acid transporter |  | unmapped by GO annotation |
| LOC_Os02g48370 | ARID/BRIGHT DNA-binding domain-containing protein |  | unmapped by GO annotation |
| LOC_Os08g43500 | armadillo/beta-catenin repeat family protein |  | unmapped by GO annotation |
| LOC_Os05g50170 | ATPCME |  | unmapped by GO annotation |
| LOC_Os01g48410 | ATROPGEF7/ROPGEF7 |  | unmapped by GO annotation |
| LOC_Os02g17240 | ATROPGEF7/ROPGEF7 |  | unmapped by GO annotation |
| LOC_Os05g48640 | ATROPGEF7/ROPGEF7 |  | unmapped by GO annotation |
| LOC_Os01g55520 | ATROPGEF7/ROPGEF7 |  | unmapped by GO annotation |
| LOC_Os07g17310 | B12D protein |  | unmapped by GO annotation |
| LOC_Os03g18360 | BTBT1- Bric-a-Brac, Tramtrack, Broad Complex BTB domain with tetratricopeptide |  | unmapped by GO annotation |
| LOC_Os02g42710 | C2 domain containing protein |  | unmapped by GO annotation |
| LOC_Os05g05650 | C2 domain containing protein |  | unmapped by GO annotation |
| LOC_Os02g57000 | C2 domain containing protein |  | unmapped by GO annotation |
| LOC_Os01g12680 | C4-dicarboxylate transporter/malic acid transport protein |  | unmapped by GO annotation |
| LOC_Os05g45810 | calcineurin B |  | unmapped by GO annotation |
| LOC_Os03g07600 | calcium-binding protein |  | unmapped by GO annotation |
| LOC_Os02g50150 | caleosin related protein |  | unmapped by GO annotation |
| LOC_Os02g50140 | caleosin related protein |  | unmapped by GO annotation |
| Locus ID | RGAP Ver 6 Annotation | GO ID | GO Name |
| LOC_Os10g27170 | calmodulin-binding protein |  | unmapped by GO annotation |
| LOC_Os11g05510 | carbonic anhydrase family protein |  | unmapped by GO annotation |
| LOC_Os10g32900 | CCT motif family protein |  | unmapped by GO annotation |
| LOC_Os03g04620 | CCT motif family protein |  | unmapped by GO annotation |
| LOC_Os05g45370 | cell cycle control protein |  | unmapped by GO annotation |
| LOC_Os06g47110 | COBRA-like protein precursor |  | unmapped by GO annotation |
| LOC_Os05g51860 | conserved hypothetical protein |  | unmapped by GO annotation |
| LOC_Os04g10500 | conserved hypothetical protein |  | unmapped by GO annotation |
| LOC_Os01g74480 | cupin domain containing protein |  | unmapped by GO annotation |
| LOC_Os08g02880 | CXXXC11 - Cysteine-rich protein with paired CXXXC motifs precursor |  | unmapped by GO annotation |
| LOC_Os02g55649 | CXXXC8 - Cysteine-rich protein with paired CXXXC motifs precursor |  | unmapped by GO annotation |
| LOC_Os07g10550 | cyclin-related protein |  | unmapped by GO annotation |
| LOC_Os02g53990 | cyclin-related protein |  | unmapped by GO annotation |
| LOC_Os10g20550 | DEFL70 - Defensin and Defensin-like DEFL family |  | unmapped by GO annotation |
| LOC_Os03g59440 | dirigent |  | unmapped by GO annotation |
| LOC_Os08g12890 | DNA binding protein |  | unmapped by GO annotation |
| LOC_Os04g20420 | DNA binding protein |  | unmapped by GO annotation |
| LOC_Os02g51730 | dnaJ homolog subfamily C member 7 |  | unmapped by GO annotation |
| LOC_Os03g51620 | DUF221 domain containing protein |  | unmapped by GO annotation |
| LOC_Os08g31080 | DUF260 domain containing protein |  | unmapped by GO annotation |
| LOC_Os01g38500 | DUF538 domain containing protein |  | unmapped by GO annotation |
| LOC_Os04g49650 | DUF581 domain containing protein |  | unmapped by GO annotation |
| LOC_Os02g46180 | DUF581 domain containing protein |  | unmapped by GO annotation |
| LOC_Os04g49650 | DUF581 domain containing protein |  | unmapped by GO annotation |
| LOC_Os11g36740 | DUF593 domain containing protein |  | unmapped by GO annotation |
| LOC_Os04g54600 | DUF617 domain containing protein |  | unmapped by GO annotation |
| Locus ID | RGAP Ver 6 Annotation | GO ID | GO Name |
| LOC_Os08g07500 | DUF617 domain containing protein |  | unmapped by GO annotation |
| LOC_Os08g44660 | EF hand family protein |  | unmapped by GO annotation |
| LOC_Os04g58480 | EF hand family protein |  | unmapped by GO annotation |
| LOC_Os04g57350 | EH domain-containing protein 1 |  | unmapped by GO annotation |
| LOC_Os09g32440 | endonuclease/exonuclease/phosphatase family domain containing protein |  | unmapped by GO annotation |
| LOC_Os06g11920 | endonuclease/exonuclease/phosphatase family domain containing protein |  | unmapped by GO annotation |
| LOC_Os02g51600 | endonuclease/exonuclease/phosphatase family domain containing protein |  | unmapped by GO annotation |
| LOC_Os08g45230 | ENTH domain containing protein |  | unmapped by GO annotation |
| LOC_Os11g02580 | esterase |  | unmapped by GO annotation |
| LOC_Os06g01780 | ethylene-responsive element-binding protein |  | unmapped by GO annotation |
| LOC_Os02g43840 | ethylene-responsive element-binding protein |  | unmapped by GO annotation |
| LOC_Os01g40150 | eukaryotic translation initiation factor 5B |  | unmapped by GO annotation |
| LOC_Os12g38450 | exostosin family domain containing protein |  | unmapped by GO annotation |
| LOC_Os01g45350 | exostosin family protein |  | unmapped by GO annotation |
| LOC_Os06g03390 | expressed protein |  | unmapped by GO annotation |
| LOC_Os10g33240 | expressed protein |  | unmapped by GO annotation |
| LOC_Os11g31400 | expressed protein |  | unmapped by GO annotation |
| LOC_Os09g30030 | expressed protein |  | unmapped by GO annotation |
| LOC_Os10g27480 | expressed protein |  | unmapped by GO annotation |
| LOC_Os03g61410 | expressed protein |  | unmapped by GO annotation |
| LOC_Os01g46850 | expressed protein |  | unmapped by GO annotation |
| LOC_Os01g23880 | expressed protein |  | unmapped by GO annotation |
| LOC_Os03g10460 | expressed protein |  | unmapped by GO annotation |
| LOC_Os12g27190 | expressed protein |  | unmapped by GO annotation |
| LOC_Os07g18070 | expressed protein |  | unmapped by GO annotation |
| LOC_Os12g22920 | expressed protein |  | unmapped by GO annotation |
| Locus ID | RGAP Ver 6 Annotation | GO ID | GO Name |
| LOC_Os10g10760 | expressed protein |  | unmapped by GO annotation |
| LOC_Os03g51210 | expressed protein |  | unmapped by GO annotation |
| LOC_Os06g44810 | expressed protein |  | unmapped by GO annotation |
| LOC_Os01g05100 | expressed protein |  | unmapped by GO annotation |
| LOC_Os08g41650 | expressed protein |  | unmapped by GO annotation |
| LOC_Os03g10334 | expressed protein |  | unmapped by GO annotation |
| LOC_Os02g58800 | expressed protein |  | unmapped by GO annotation |
| LOC_Os09g12630 | expressed protein |  | unmapped by GO annotation |
| LOC_Os07g37480 | expressed protein |  | unmapped by GO annotation |
| LOC_Os04g01330 | expressed protein |  | unmapped by GO annotation |
| LOC_Os09g30040 | expressed protein |  | unmapped by GO annotation |
| LOC_Os04g21340 | expressed protein |  | unmapped by GO annotation |
| LOC_Os04g33710 | expressed protein |  | unmapped by GO annotation |
| LOC_Os11g08680 | expressed protein |  | unmapped by GO annotation |
| LOC_Os01g06620 | expressed protein |  | unmapped by GO annotation |
| LOC_Os11g08707 | expressed protein |  | unmapped by GO annotation |
| LOC_Os02g09540 | expressed protein |  | unmapped by GO annotation |
| LOC_Os03g58190 | expressed protein |  | unmapped by GO annotation |
| LOC_Os07g39930 | expressed protein |  | unmapped by GO annotation |
| LOC_Os02g05670 | expressed protein |  | unmapped by GO annotation |
| LOC_Os06g43060 | expressed protein |  | unmapped by GO annotation |
| LOC_Os07g15530 | expressed protein |  | unmapped by GO annotation |
| LOC_Os04g57270 | expressed protein |  | unmapped by GO annotation |
| LOC_Os04g57280 | expressed protein |  | unmapped by GO annotation |
| LOC_Os05g51900 | expressed protein |  | unmapped by GO annotation |
| LOC_Os03g64310 | expressed protein |  | unmapped by GO annotation |
| Locus ID | RGAP Ver 6 Annotation | GO ID | GO Name |
| LOC_Os01g42024 | expressed protein |  | unmapped by GO annotation |
| LOC_Os06g03980 | expressed protein |  | unmapped by GO annotation |
| LOC_Os03g61590 | expressed protein |  | unmapped by GO annotation |
| LOC_Os04g58100 | expressed protein |  | unmapped by GO annotation |
| LOC_Os07g09384 | expressed protein |  | unmapped by GO annotation |
| LOC_Os05g41150 | expressed protein |  | unmapped by GO annotation |
| LOC_Os06g10480 | expressed protein |  | unmapped by GO annotation |
| LOC_Os12g43990 | expressed protein |  | unmapped by GO annotation |
| LOC_Os05g23194 | expressed protein |  | unmapped by GO annotation |
| LOC_Os03g43100 | expressed protein |  | unmapped by GO annotation |
| LOC_Os11g09030 | expressed protein |  | unmapped by GO annotation |
| LOC_Os02g04210 | expressed protein |  | unmapped by GO annotation |
| LOC_Os07g28920 | expressed protein |  | unmapped by GO annotation |
| LOC_Os05g20914 | expressed protein |  | unmapped by GO annotation |
| LOC_Os01g71040 | expressed protein |  | unmapped by GO annotation |
| LOC_Os01g56764 | expressed protein |  | unmapped by GO annotation |
| LOC_Os01g55850 | expressed protein |  | unmapped by GO annotation |
| LOC_Os02g50980 | expressed protein |  | unmapped by GO annotation |
| LOC_Os05g40310 | expressed protein |  | unmapped by GO annotation |
| LOC_Os01g70120 | expressed protein |  | unmapped by GO annotation |
| LOC_Os01g42060 | expressed protein |  | unmapped by GO annotation |
| LOC_Os07g20270 | expressed protein |  | unmapped by GO annotation |
| LOC_Os02g54100 | expressed protein |  | unmapped by GO annotation |
| LOC_Os08g07600 | expressed protein |  | unmapped by GO annotation |
| LOC_Os10g40170 | expressed protein |  | unmapped by GO annotation |
| LOC_Os09g09350 | expressed protein |  | unmapped by GO annotation |
| Locus ID | RGAP Ver 6 Annotation | GO ID | GO Name |
| LOC_Os11g40800 | expressed protein |  | unmapped by GO annotation |
| LOC_Os09g12510 | expressed protein |  | unmapped by GO annotation |
| LOC_Os05g38960 | expressed protein |  | unmapped by GO annotation |
| LOC_Os03g04560 | expressed protein |  | unmapped by GO annotation |
| LOC_Os03g17270 | expressed protein |  | unmapped by GO annotation |
| LOC_Os01g09730 | expressed protein |  | unmapped by GO annotation |
| LOC_Os03g08980 | expressed protein |  | unmapped by GO annotation |
| LOC_Os02g01290 | expressed protein |  | unmapped by GO annotation |
| LOC_Os11g36230 | expressed protein |  | unmapped by GO annotation |
| LOC_Os11g08400 | expressed protein |  | unmapped by GO annotation |
| LOC_Os02g38170 | expressed protein |  | unmapped by GO annotation |
| LOC_Os11g05950 | expressed protein |  | unmapped by GO annotation |
| LOC_Os06g05730 | expressed protein |  | unmapped by GO annotation |
| LOC_Os02g10530 | expressed protein |  | unmapped by GO annotation |
| LOC_Os02g26390 | expressed protein |  | unmapped by GO annotation |
| LOC_Os08g01324 | expressed protein |  | unmapped by GO annotation |
| LOC_Os05g28710 | expressed protein |  | unmapped by GO annotation |
| LOC_Os04g37520 | extracellular ligand-gated ion channel |  | unmapped by GO annotation |
| LOC_Os06g44660 | fasciclin-like arabinogalactan precursor protein |  | unmapped by GO annotation |
| LOC_Os04g21570 | fasciclin-like arabinogalactan precursor protein |  | unmapped by GO annotation |
| LOC_Os02g26320 | fasciclin-like arabinogalactan precursor protein |  | unmapped by GO annotation |
| LOC_Os02g26290 | fasciclin-like arabinogalactan protein 8 precursor |  | unmapped by GO annotation |
| LOC_Os02g52840 | flavonol synthase/flavanone 3-hydroxylase |  | unmapped by GO annotation |
| LOC_Os10g39020 | fringe-related protein |  | unmapped by GO annotation |
| LOC_Os12g43820 | GCRP5 - Glycine and cysteine rich family protein precursor |  | unmapped by GO annotation |
| LOC_Os09g25650 | GEX2 |  | unmapped by GO annotation |
| Locus ID | RGAP Ver 6 Annotation | GO ID | GO Name |
| LOC_Os08g38740 | glycosyl transferase 8 domain containing protein |  | unmapped by GO annotation |
| LOC_Os07g45260 | glycosyl transferase 8 domain containing protein |  | unmapped by GO annotation |
| LOC_Os01g48540 | glyoxal oxidase-related |  | unmapped by GO annotation |
| LOC_Os05g48520 | glyoxal oxidase-related |  | unmapped by GO annotation |
| LOC_Os04g42210 | GPI-anchored protein |  | unmapped by GO annotation |
| LOC_Os04g42220 | GPI-anchored protein |  | unmapped by GO annotation |
| LOC_Os02g33740 | GPI-anchored protein |  | unmapped by GO annotation |
| LOC_Os02g10630 | GRAM and C2 domains containing protein |  | unmapped by GO annotation |
| LOC_Os06g45200 | group 3 pollen allergen |  | unmapped by GO annotation |
| LOC_Os05g37880 | growth regulator related protein |  | unmapped by GO annotation |
| LOC_Os07g31830 | GTPase activating protein |  | unmapped by GO annotation |
| LOC_Os07g01780 | GTPase activating protein |  | unmapped by GO annotation |
| LOC_Os06g04790 | HAD superfamily phosphatase |  | unmapped by GO annotation |
| LOC_Os08g23460 | harpin-induced protein 1 domain containing protein |  | unmapped by GO annotation |
| LOC_Os03g27110 | hydrolase protein |  | unmapped by GO annotation |
| LOC_Os03g08110 | hydrolase protein |  | unmapped by GO annotation |
| LOC_Os02g32060 | hydrolase, NUDIX family, domain containing protein |  | unmapped by GO annotation |
| LOC_Os03g09160 | hydroxyproline-rich glycoprotein family protein |  | unmapped by GO annotation |
| LOC_Os08g35770 | hypothetical protein |  | unmapped by GO annotation |
| LOC_Os11g11240 | hypothetical protein |  | unmapped by GO annotation |
| LOC_Os01g26290 | hypothetical protein |  | unmapped by GO annotation |
| LOC_Os01g23370 | hypothetical protein |  | unmapped by GO annotation |
| LOC_Os03g49590 | hypothetical protein |  | unmapped by GO annotation |
| LOC_Os02g07580 | hypothetical protein |  | unmapped by GO annotation |
| LOC_Os12g32310 | hypothetical protein |  | unmapped by GO annotation |
| LOC_Os07g07130 | hypothetical protein |  | unmapped by GO annotation |
| Locus ID | RGAP Ver 6 Annotation | GO ID | GO Name |
| LOC_Os03g13120 | hypothetical protein |  | unmapped by GO annotation |
| LOC_Os02g32210 | hypothetical protein |  | unmapped by GO annotation |
| LOC_Os09g16970 | hypothetical protein |  | unmapped by GO annotation |
| LOC_Os04g29050 | hypothetical protein |  | unmapped by GO annotation |
| LOC_Os11g08698 | hypothetical protein |  | unmapped by GO annotation |
| LOC_Os08g42740 | IBR domain containing protein |  | unmapped by GO annotation |
| LOC_Os01g59880 | inositol-1, 4, 5-trisphosphate 5-phosphatase |  | unmapped by GO annotation |
| LOC_Os11g37720 | integral membrane protein DUF6 domain containing protein |  | unmapped by GO annotation |
| LOC_Os05g20570 | invertase/pectin methylesterase inhibitor family protein |  | unmapped by GO annotation |
| LOC_Os01g50810 | invertase/pectin methylesterase inhibitor family protein |  | unmapped by GO annotation |
| LOC_Os05g46530 | invertase/pectin methylesterase inhibitor family protein |  | unmapped by GO annotation |
| LOC_Os02g01310 | invertase/pectin methylesterase inhibitor family protein |  | unmapped by GO annotation |
| LOC_Os03g61530 | invertase/pectin methylesterase inhibitor family protein |  | unmapped by GO annotation |
| LOC_Os01g20970 | invertase/pectin methylesterase inhibitor family protein |  | unmapped by GO annotation |
| LOC_Os10g10700 | invertase/pectin methylesterase inhibitor family protein |  | unmapped by GO annotation |
| LOC_Os01g14940 | invertase/pectin methylesterase inhibitor family protein |  | unmapped by GO annotation |
| LOC_Os11g45220 | IWS1 homolog A |  | unmapped by GO annotation |
| LOC_Os08g30800 | KED |  | unmapped by GO annotation |
| LOC_Os05g39000 | KIP1 |  | unmapped by GO annotation |
| LOC_Os01g61910 | KIP1 |  | unmapped by GO annotation |
| LOC_Os01g74510 | KIP1 |  | unmapped by GO annotation |
| LOC_Os06g44900 | leaf senescence related protein |  | unmapped by GO annotation |
| LOC_Os03g08610 | leucine-rich repeat family protein |  | unmapped by GO annotation |
| LOC_Os02g38040 | leucine-rich repeat family protein |  | unmapped by GO annotation |
| LOC_Os12g16180 | lipase |  | unmapped by GO annotation |
| LOC_Os04g42960 | Lung seven transmembrane receptor domain containing protein |  | unmapped by GO annotation |
| Locus ID | RGAP Ver 6 Annotation | GO ID | GO Name |
| LOC_Os02g40550 | Lung seven transmembrane receptor domain containing protein |  | unmapped by GO annotation |
| LOC_Os03g12180 | MA3 domain containing protein |  | unmapped by GO annotation |
| LOC_Os02g03400 | microtubule associated protein |  | unmapped by GO annotation |
| LOC_Os01g60080 | monocopper oxidase |  | unmapped by GO annotation |
| LOC_Os05g40740 | monocopper oxidase |  | unmapped by GO annotation |
| LOC_Os10g25674 | mps one binder kinase activator-like 1A |  | unmapped by GO annotation |
| LOC_Os10g40140 | MSP domain containing protein |  | unmapped by GO annotation |
| LOC_Os08g38280 | mucin-associated surface protein |  | unmapped by GO annotation |
| LOC_Os02g06510 | myosin heavy chain |  | unmapped by GO annotation |
| LOC_Os06g29350 | myosin |  | unmapped by GO annotation |
| LOC_Os11g23220 | myosin |  | unmapped by GO annotation |
| LOC_Os06g43000 | nitrate-induced NOI protein |  | unmapped by GO annotation |
| LOC_Os02g49340 | nitrate-induced NOI protein |  | unmapped by GO annotation |
| LOC_Os01g58640 | nucleotide pyrophosphatase/phosphodiesterase |  | unmapped by GO annotation |
| LOC_Os09g03890 | octicosapeptide/Phox/Bem1p |  | unmapped by GO annotation |
| LOC_Os05g50110 | oleosin |  | unmapped by GO annotation |
| LOC_Os08g23130 | oligopeptide transporter |  | unmapped by GO annotation |
| LOC_Os10g04370 | OsFBX357 - F-box domain containing protein |  | unmapped by GO annotation |
| LOC_Os02g42820 | OsPLIM2a - LIM domain protein actin-binding protein and transcription factor |  | unmapped by GO annotation |
| LOC_Os04g45010 | OsPLIM2b - LIM domain protein actin-binding protein and transcription factor |  | unmapped by GO annotation |
| LOC_Os10g35930 | OsPLIM2c - LIM domain protein actin-binding protein and transcription factor |  | unmapped by GO annotation |
| LOC_Os03g25460 | OsRCI2-4 - Putative low temperature and salt responsive protein |  | unmapped by GO annotation |
| LOC_Os01g67490 | OTU-like cysteine protease family protein |  | unmapped by GO annotation |
| LOC_Os07g26480 | P21-Rho-binding domain containing protein |  | unmapped by GO annotation |
| LOC_Os09g28450 | paramyosin |  | unmapped by GO annotation |
| LOC_Os11g30350 | PB1 domain containing protein |  | unmapped by GO annotation |
| Locus ID | RGAP Ver 6 Annotation | GO ID | GO Name |
| LOC_Os06g38510 | pectate lyase precursor |  | unmapped by GO annotation |
| LOC_Os06g05260 | pectate lyase precursor |  | unmapped by GO annotation |
| LOC_Os02g12300 | pectate lyase precursor |  | unmapped by GO annotation |
| LOC_Os06g05209 | pectate lyase precursor |  | unmapped by GO annotation |
| LOC_Os08g04650 | pectinesterase inhibitor domain containing protein |  | unmapped by GO annotation |
| LOC_Os05g05640 | pectinesterase inhibitor domain containing protein |  | unmapped by GO annotation |
| LOC_Os08g38200 | phosphatidylinositol 3- and 4-kinase family protein |  | unmapped by GO annotation |
| LOC_Os05g51230 | phosphatidylinositol 3- and 4-kinase family protein |  | unmapped by GO annotation |
| LOC_Os01g16470 | phosphatidylinositol kinase |  | unmapped by GO annotation |
| LOC_Os11g29780 | plant-specific domain TIGR01627 family protein |  | unmapped by GO annotation |
| LOC_Os04g57750 | plastocyanin-like domain containing protein |  | unmapped by GO annotation |
| LOC_Os02g49350 | plastocyanin-like domain containing protein |  | unmapped by GO annotation |
| LOC_Os05g49580 | plastocyanin-like domain containing protein |  | unmapped by GO annotation |
| LOC_Os03g44630 | plastocyanin-like domain containing protein |  | unmapped by GO annotation |
| LOC_Os12g03510 | PME/invertase inhibitor |  | unmapped by GO annotation |
| LOC_Os06g36240 | POEI21 - Pollen Ole e I allergen and extensin family protein precursor |  | unmapped by GO annotation |
| LOC_Os09g39950 | POEI23 - Pollen Ole e I allergen and extensin family protein precursor |  | unmapped by GO annotation |
| LOC_Os09g27710 | pollen allergen Cyn d 23 |  | unmapped by GO annotation |
| LOC_Os04g25150 | pollen allergen |  | unmapped by GO annotation |
| LOC_Os06g45160 | pollen allergen |  | unmapped by GO annotation |
| LOC_Os04g25160 | pollen allergen |  | unmapped by GO annotation |
| LOC_Os06g44470 | pollen allergen |  | unmapped by GO annotation |
| LOC_Os04g25190 | pollen allergen |  | unmapped by GO annotation |
| LOC_Os06g45150 | pollen allergen |  | unmapped by GO annotation |
| LOC_Os06g45180 | pollen allergen |  | unmapped by GO annotation |
| LOC_Os06g45190 | pollen allergen |  | unmapped by GO annotation |
| Locus ID | RGAP Ver 6 Annotation | GO ID | GO Name |
| LOC_Os12g42650 | pollen preferential protein |  | unmapped by GO annotation |
| LOC_Os03g17030 | polyadenylate-binding protein |  | unmapped by GO annotation |
| LOC_Os12g30550 | protein of unknown function domain containing protein |  | unmapped by GO annotation |
| LOC_Os04g49739 | purine permease |  | unmapped by GO annotation |
| LOC_Os07g13440 | RALFL12 - Rapid ALkalinization Factor RALF family protein precursor |  | unmapped by GO annotation |
| LOC_Os07g13450 | RALFL13 - Rapid ALkalinization Factor RALF family protein precursor |  | unmapped by GO annotation |
| LOC_Os01g10470 | RALFL17 - Rapid ALkalinization Factor RALF family protein precursor |  | unmapped by GO annotation |
| LOC_Os05g11330 | RALFL19 - Rapid ALkalinization Factor RALF family protein precursor |  | unmapped by GO annotation |
| LOC_Os12g35690 | RALFL5 - Rapid ALkalinization Factor RALF family protein precursor |  | unmapped by GO annotation |
| LOC_Os05g46740 | regulator of chromosome condensation |  | unmapped by GO annotation |
| LOC_Os01g50470 | regulator of chromosome condensation |  | unmapped by GO annotation |
| LOC_Os11g29210 | resistance-gene-interacting protein |  | unmapped by GO annotation |
| LOC_Os06g35590 | reticuline oxidase-like protein precursor |  | unmapped by GO annotation |
| LOC_Os05g24770 | reticulon domain containing protein |  | unmapped by GO annotation |
| LOC_Os06g45184 | retrotransposon protein, Ty3-gypsy subclass |  | unmapped by GO annotation |
| LOC_Os02g41940 | retrotransposon protein, unclassified |  | unmapped by GO annotation |
| LOC_Os01g11800 | retrotransposon protein, unclassified |  | unmapped by GO annotation |
| LOC_Os03g16420 | retrotransposon protein, unclassified |  | unmapped by GO annotation |
| LOC_Os01g69020 | retrotransposon protein, unclassified |  | unmapped by GO annotation |
| LOC_Os05g20620 | retrotransposon protein, unclassified |  | unmapped by GO annotation |
| LOC_Os03g01930 | retrotransposon protein, unclassified |  | unmapped by GO annotation |
| LOC_Os03g53550 | retrotransposon protein, unclassified |  | unmapped by GO annotation |
| LOC_Os01g68540 | rho GDP-dissociation inhibitor 1 |  | unmapped by GO annotation |
| LOC_Os11g20384 | SacI homology domain containing protein |  | unmapped by GO annotation |
| LOC_Os12g43700 | SCP-like extracellular protein |  | unmapped by GO annotation |
| LOC_Os05g51680 | SCP-like extracellular protein |  | unmapped by GO annotation |
| Locus ID | RGAP Ver 6 Annotation | GO ID | GO Name |
| LOC_Os05g51660 | SCP-like extracellular protein |  | unmapped by GO annotation |
| LOC_Os08g35440 | Ser/Thr protein phosphatase family protein |  | unmapped by GO annotation |
| LOC_Os11g06690 | serine esterase family protein |  | unmapped by GO annotation |
| LOC_Os12g41170 | SEY1 |  | unmapped by GO annotation |
| LOC_Os02g32740 | SNARE domain containing protein |  | unmapped by GO annotation |
| LOC_Os07g48790 | SNF1-related protein kinase regulatory subunit beta-1 |  | unmapped by GO annotation |
| LOC_Os02g04630 | sodium/calcium exchanger protein |  | unmapped by GO annotation |
| LOC_Os02g01270 | START domain containing protein |  | unmapped by GO annotation |
| LOC_Os02g45890 | sulfotransferase domain containing protein |  | unmapped by GO annotation |
| LOC_Os02g51310 | TCP family transcription factor |  | unmapped by GO annotation |
| LOC_Os01g72280 | tetratricopeptide repeat containing protein |  | unmapped by GO annotation |
| LOC_Os11g46230 | tetratricopeptide repeat domain containing protein |  | unmapped by GO annotation |
| LOC_Os07g49250 | thiamine pyrophosphate enzyme, C-terminal TPP binding domain containing protein |  | unmapped by GO annotation |
| LOC_Os02g03520 | THION25 - Plant thionin family protein precursor |  | unmapped by GO annotation |
| LOC_Os03g64300 | THION30 - Plant thionin family protein precursor |  | unmapped by GO annotation |
| LOC_Os03g49270 | THION36 - Plant thionin family protein precursor |  | unmapped by GO annotation |
| LOC_Os12g38570 | transmembrane amino acid transporter protein |  | unmapped by GO annotation |
| LOC_Os03g58140 | transmembrane BAX inhibitor motif-containing protein |  | unmapped by GO annotation |
| LOC_Os07g08060 | transmembrane BAX inhibitor motif-containing protein |  | unmapped by GO annotation |
| LOC_Os10g27370 | transposon protein, Pong sub-class |  | unmapped by GO annotation |
| LOC_Os02g36950 | uncharacterized Cys-rich domain containing protein |  | unmapped by GO annotation |
| LOC_Os04g47320 | uncharacterized mscS family protein |  | unmapped by GO annotation |
| LOC_Os05g30940 | vacuolar calcium binding protein |  | unmapped by GO annotation |
| LOC_Os10g34680 | vacuolar protein sorting-associated protein 52 |  | unmapped by GO annotation |
| LOC_Os01g71780 | WD domain, G-beta repeat domain containing protein |  | unmapped by GO annotation |
| LOC_Os03g52870 | WD-40 repeat family protein |  | unmapped by GO annotation |
| Locus ID | RGAP Ver 6 Annotation | GO ID | GO Name |
| LOC_Os11g22350 | white-brown complex homolog protein |  | unmapped by GO annotation |
| LOC_Os06g51460 | white-brown complex homolog protein |  | unmapped by GO annotation |
| LOC_Os10g25560 | XI-I |  | unmapped by GO annotation |
| LOC_Os01g10440 | xylosyltransferase |  | unmapped by GO annotation |
| LOC_Os03g27900 | ZIM motif family protein |  | unmapped by GO annotation |
| LOC_Os05g11720 | zinc finger, C3HC4 type domain containing protein |  | unmapped by GO annotation |
| LOC_Os10g42390 | zinc finger, C3HC4 type domain containing protein |  | unmapped by GO annotation |
| LOC_Os01g68900 | zinc finger, C3HC4 type family protein |  | unmapped by GO annotation |
| LOC_Os08g33860 | zinc finger, C3HC4 type |  | unmapped by GO annotation |
| LOC_Os02g44120 | ZOS2-13 - C2H2 zinc finger protein |  | unmapped by GO annotation |
| LOC_Os03g17150 | ZOS3-09 - C2H2 zinc finger protein |  | unmapped by GO annotation |
| LOC_Os04g46670 | ZOS4-09 - C2H2 zinc finger protein |  | unmapped by GO annotation |

**Table S5.** MapMan classification of late pollen-preferred genes.

| Bin.Code | Bin.Name | Locus_ID |
| --- | --- | --- |
| 1.1.30 | PS.lightreaction.state transition | LOC_Os05g40180.1 |
| 1.3.6 | PS.calvin cycle.aldolase | LOC_Os10g08022.1 |
| 2.1.2.1 | major CHO metabolism.synthesis.starch.AGPase | LOC_Os07g13980.1 |
| 2.1.2.2 | major CHO metabolism.synthesis.starch.starch synthase | LOC_Os06g04200.1 |
| 2.2.1.3.3 | major CHO metabolism.degradation.sucrose.invertases.vacuolar | LOC_Os02g01590.1 |
| 2.2.1.3.3 | major CHO metabolism.degradation.sucrose.invertases.vacuolar | LOC_Os04g45290.1 |
| 2.2.2.1.2 | major CHO metabolism.degradation.starch.starch cleavage.beta amylase | LOC_Os03g04770.1 |
| 2.2.2.1.2 | major CHO metabolism.degradation.starch.starch cleavage.beta amylase | LOC_Os10g32810.1 |
| 2.2.2.1.2 | major CHO metabolism.degradation.starch.starch cleavage.beta amylase | LOC_Os07g47120.1 |
| 3.2.2 | minor CHO metabolism.trehalose.TPP | LOC_Os07g30160.1 |
| 3.6 | minor CHO metabolism.callose | LOC_Os06g08380.1 |
| 5.2 | fermentation.PDC | LOC_Os07g49250.1 |
| 8.2.10 | TCA / org. transformation.other organic acid transformaitons.malic | LOC_Os05g09440.1 |
| 8.3 | TCA / org. transformation.carbonic anhydrases | LOC_Os09g28150.1 |
| 8.3 | TCA / org. transformation.carbonic anhydrases | LOC_Os12g05730.1 |
| 8.3 | TCA / org. transformation.carbonic anhydrases | LOC_Os09g28130.1 |
| 8.3 | TCA / org. transformation.carbonic anhydrases | LOC_Os11g05510.1 |
| 10.1.2 | cell wall.precursor synthesis.UGE | LOC_Os05g51670.1 |
| 10.1.6 | cell wall.precursor synthesis.GAE | LOC_Os06g08810.1 |
| 10.2.1 | cell wall.cellulose synthesis.cellulose synthase | LOC_Os08g25710.1 |
| 10.2.1 | cell wall.cellulose synthesis.cellulose synthase | LOC_Os04g35020.1 |
| 10.2.1 | cell wall.cellulose synthesis.cellulose synthase | LOC_Os06g22980.1 |
| 10.2.2 | cell wall.cellulose synthesis.COBRA | LOC_Os06g47110.1 |
| 10.3 | cell wall.hemicellulose synthesis | LOC_Os06g10970.1 |
| Bin.Code | Bin.Name | Locus_ID |
| 10.5 | cell wall.cell wall proteins | LOC_Os01g69240.1 |
| 10.5.1.1 | cell wall.cell wall proteins.AGPs.AGP | LOC_Os06g44660.1 |
| 10.5.1.1 | cell wall.cell wall proteins.AGPs.AGP | LOC_Os02g26320.1 |
| 10.5.3 | cell wall.cell wall proteins.LRR | LOC_Os12g35710.1 |
| 10.5.3 | cell wall.cell wall proteins.LRR | LOC_Os01g25460.1 |
| 10.6.2 | cell wall.degradation.mannan-xylose-arabinose-fucose | LOC_Os10g21110.1 |
| 10.6.3 | cell wall.degradation.pectate lyases and polygalacturonases | LOC_Os01g33300.1 |
| 10.6.3 | cell wall.degradation.pectate lyases and polygalacturonases | LOC_Os06g05209.1 |
| 10.6.3 | cell wall.degradation.pectate lyases and polygalacturonases | LOC_Os02g10300.1 |
| 10.6.3 | cell wall.degradation.pectate lyases and polygalacturonases | LOC_Os06g40890.1 |
| 10.6.3 | cell wall.degradation.pectate lyases and polygalacturonases | LOC_Os06g05260.1 |
| 10.6.3 | cell wall.degradation.pectate lyases and polygalacturonases | LOC_Os06g35320.1 |
| 10.6.3 | cell wall.degradation.pectate lyases and polygalacturonases | LOC_Os02g12300.1 |
| 10.6.3 | cell wall.degradation.pectate lyases and polygalacturonases | LOC_Os06g38510.1 |
| 10.7 | cell wall.modification | LOC_Os04g25190.1 |
| 10.7 | cell wall.modification | LOC_Os04g25160.1 |
| 10.7 | cell wall.modification | LOC_Os03g01610.1 |
| 10.7 | cell wall.modification | LOC_Os06g45190.1 |
| 10.7 | cell wall.modification | LOC_Os02g03550.1 |
| 10.7 | cell wall.modification | LOC_Os06g45160.1 |
| 10.7 | cell wall.modification | LOC_Os06g45150.1 |
| 10.7 | cell wall.modification | LOC_Os08g44790.1 |
| 10.7 | cell wall.modification | LOC_Os06g45180.1 |
| 10.7 | cell wall.modification | LOC_Os08g13980.1 |
| 10.7 | cell wall.modification | LOC_Os12g36040.1 |
| 10.7 | cell wall.modification | LOC_Os03g01640.1 |
| Bin.Code | Bin.Name | Locus_ID |
| 10.7 | cell wall.modification | LOC_Os06g44470.1 |
| 10.7 | cell wall.modification | LOC_Os10g40090.1 |
| 10.7 | cell wall.modification | LOC_Os04g25150.1 |
| 10.7 | cell wall.modification | LOC_Os06g45200.1 |
| 10.8.1 | cell wall.pectin*esterases.PME | LOC_Os03g18860.1 |
| 10.8.1 | cell wall.pectin*esterases.PME | LOC_Os03g19610.1 |
| 10.8.1 | cell wall.pectin*esterases.PME | LOC_Os04g38560.1 |
| 10.8.1 | cell wall.pectin*esterases.PME | LOC_Os07g49100.1 |
| 10.8.1 | cell wall.pectin*esterases.PME | LOC_Os09g26360.1 |
| 10.8.1 | cell wall.pectin*esterases.PME | LOC_Os11g45720.1 |
| 10.8.1 | cell wall.pectin*esterases.PME | LOC_Os11g45730.1 |
| 10.8.1 | cell wall.pectin*esterases.PME | LOC_Os03g28090.1 |
| 10.8.1 | cell wall.pectin*esterases.PME | LOC_Os12g37660.1 |
| 10.8.1 | cell wall.pectin*esterases.PME | LOC_Os08g34900.1 |
| 10.8.1 | cell wall.pectin*esterases.PME | LOC_Os04g54850.1 |
| 11.3.5 | lipid metabolism.Phospholipid synthesis.diacylglycerol kinase | LOC_Os08g15090.1 |
| 11.3.6 | lipid metabolism.Phospholipid synthesis.choline-phosphate cytidylyltransferase | LOC_Os08g06450.1 |
| 11.4 | lipid metabolism.TAG synthesis | LOC_Os05g50110.1 |
| 11.8 | lipid metabolism.''exotics'' (steroids, squalene etc) | LOC_Os02g45890.1 |
| 11.9.2 | lipid metabolism.lipid degradation.lipases | LOC_Os12g16180.1 |
| 11.9.3.1 | lipid metabolism.lipid degradation.lysophospholipases.phospholipase D | LOC_Os03g02740.1 |
| 11.9.3.1 | lipid metabolism.lipid degradation.lysophospholipases.phospholipase D | LOC_Os03g62410.1 |
| 11.9.3.1 | lipid metabolism.lipid degradation.lysophospholipases.phospholipase D | LOC_Os05g07880.1 |
| 11.9.3.1 | lipid metabolism.lipid degradation.lysophospholipases.phospholipase D | LOC_Os07g15680.1 |
| 11.9.3.3 | lipid metabolism.lipid degradation.lysophospholipases.glycerophosphodiester phosphodiesterase | LOC_Os02g09450.1 |
| 13.1.1.1.1 | amino acid metabolism.synthesis.central amino acid metabolism.GABA.Glutamate decarboxylase | LOC_Os04g37460.1 |
| Bin.Code | Bin.Name | Locus_ID |
| 13.99 | amino acid metabolism.misc | LOC_Os03g51990.1 |
| 16.1.5 | secondary metabolism.isoprenoids.terpenoids | LOC_Os02g17780.1 |
| 16.5.99.1 | secondary metabolism.sulfur-containing.misc.alliinase | LOC_Os05g07720.1 |
| 16.8.4 | secondary metabolism.flavonoids.flavonols | LOC_Os02g52840.1 |
| 17.1.2 | hormone metabolism.abscisic acid.signal transduction | LOC_Os01g51610.1 |
| 17.2.1 | hormone metabolism.auxin.synthesis-degradation | LOC_Os01g51060.1 |
| 17.2.3 | hormone metabolism.auxin.induced-regulated-responsive-activated | LOC_Os01g55940.1 |
| 17.2.3 | hormone metabolism.auxin.induced-regulated-responsive-activated | LOC_Os05g37880.1 |
| 17.3.1.1.1 | hormone metabolism.brassinosteroid.synthesis-degradation.BRs.DET2 | LOC_Os07g06800.1 |
| 17.5.1 | hormone metabolism.ethylene.synthesis-degradation | LOC_Os02g52840.1 |
| 17.5.1 | hormone metabolism.ethylene.synthesis-degradation | LOC_Os05g08540.1 |
| 17.5.2 | hormone metabolism.ethylene.signal transduction | LOC_Os06g01780.1 |
| 17.5.2 | hormone metabolism.ethylene.signal transduction | LOC_Os02g43840.1 |
| 17.5.3 | hormone metabolism.ethylene.induced-regulated-responsive-activated | LOC_Os12g36630.1 |
| 17.6.1.11 | hormone metabolism.gibberelin.synthesis-degradation.GA20 oxidase | LOC_Os07g07420.1 |
| 17.6.1.13 | hormone metabolism.gibberelin.synthesis-degradation.GA2 oxidase | LOC_Os05g06670.1 |
| 20.1 | stress.biotic | LOC_Os02g01270.1 |
| 20.1 | stress.biotic | LOC_Os05g51660.1 |
| 20.1 | stress.biotic | LOC_Os05g24770.1 |
| 20.1 | stress.biotic | LOC_Os08g23460.1 |
| 20.1 | stress.biotic | LOC_Os12g43700.1 |
| 20.1 | stress.biotic | LOC_Os05g51680.2 |
| 20.1 | stress.biotic | LOC_Os07g01770.1 |
| 20.1.1 | stress.biotic.respiratory burst | LOC_Os05g38980.1 |
| 20.1.3.1 | stress.biotic.signalling.MLO-like | LOC_Os05g09050.1 |
| 20.1.7 | stress.biotic.PR-proteins | LOC_Os03g59440.1 |
| Bin.Code | Bin.Name | Locus_ID |
| 20.2 | stress.abiotic | LOC_Os02g10990.1 |
| 20.2.1 | stress.abiotic.heat | LOC_Os08g28700.1 |
| 20.2.3 | stress.abiotic.drought/salt | LOC_Os03g51620.1 |
| 20.2.99 | stress.abiotic.unspecified | LOC_Os09g39950.1 |
| 21.1 | redox.thioredoxin | LOC_Os02g51730.1 |
| 21.1 | redox.thioredoxin | LOC_Os05g40190.1 |
| 21.4 | redox.glutaredoxins | LOC_Os04g54860.1 |
| 21.4 | redox.glutaredoxins | LOC_Os05g28530.1 |
| 23.3.3 | nucleotide metabolism.salvage.NUDIX hydrolases | LOC_Os02g32060.1 |
| 23.4.99 | nucleotide metabolism.phosphotransfer and pyrophosphatases.misc | LOC_Os10g26600.1 |
| 24 | Biodegradation of Xenobiotics | LOC_Os01g48540.1 |
| 24 | Biodegradation of Xenobiotics | LOC_Os05g48520.1 |
| 26.2 | misc.UDP glucosyl and glucoronyl transferases | LOC_Os03g24510.1 |
| 26.2 | misc.UDP glucosyl and glucoronyl transferases | LOC_Os10g07970.1 |
| 26.2 | misc.UDP glucosyl and glucoronyl transferases | LOC_Os08g38740.1 |
| 26.2 | misc.UDP glucosyl and glucoronyl transferases | LOC_Os03g59350.1 |
| 26.2 | misc.UDP glucosyl and glucoronyl transferases | LOC_Os01g10440.1 |
| 26.2 | misc.UDP glucosyl and glucoronyl transferases | LOC_Os07g45260.1 |
| 26.2 | misc.UDP glucosyl and glucoronyl transferases | LOC_Os01g45350.1 |
| 26.2 | misc.UDP glucosyl and glucoronyl transferases | LOC_Os12g38450.1 |
| 26.3 | misc.gluco-, galacto- and mannosidases | LOC_Os12g23170.1 |
| 26.3.2 | misc.gluco-, galacto- and mannosidases.beta-galactosidase | LOC_Os09g36810.1 |
| 26.3.2 | misc.gluco-, galacto- and mannosidases.beta-galactosidase | LOC_Os08g43570.1 |
| 26.3.4 | misc.gluco-, galacto- and mannosidases.endoglucanase | LOC_Os06g50140.1 |
| 26.4 | misc.beta 1,3 glucan hydrolases | LOC_Os10g21110.1 |
| 26.4.1 | misc.beta 1,3 glucan hydrolases.glucan endo-1,3-beta-glucosidase | LOC_Os06g39060.1 |
| Bin.Code | Bin.Name | Locus_ID |
| 26.4.1 | misc.beta 1,3 glucan hydrolases.glucan endo-1,3-beta-glucosidase | LOC_Os07g13580.1 |
| 26.7 | misc.oxidases - copper, flavone etc. | LOC_Os01g12490.1 |
| 26.7 | misc.oxidases - copper, flavone etc. | LOC_Os05g40740.1 |
| 26.7 | misc.oxidases - copper, flavone etc. | LOC_Os01g60080.1 |
| 26.8 | misc.nitrilases, *nitrile lyases, berberine bridge enzymes, reticuline oxidases, troponine reductases | LOC_Os12g41780.1 |
| 26.8 | misc.nitrilases, *nitrile lyases, berberine bridge enzymes, reticuline oxidases, troponine reductases | LOC_Os11g02580.1 |
| 26.8 | misc.nitrilases, *nitrile lyases, berberine bridge enzymes, reticuline oxidases, troponine reductases | LOC_Os06g35590.1 |
| 26.1 | misc.cytochrome P450 | LOC_Os06g02019.1 |
| 26.12 | misc.peroxidases | LOC_Os03g05770.1 |
| 26.12 | misc.peroxidases | LOC_Os06g27850.1 |
| 26.12 | misc.peroxidases | LOC_Os02g50770.1 |
| 26.13 | misc.acid and other phosphatases | LOC_Os01g58640.1 |
| 26.13 | misc.acid and other phosphatases | LOC_Os06g04790.1 |
| 26.14 | misc.oxygenases | LOC_Os01g08570.1 |
| 26.17 | misc.dynamin | LOC_Os08g32920.1 |
| 26.18 | misc.invertase/pectin methylesterase inhibitor family protein | LOC_Os01g50810.1 |
| 26.18 | misc.invertase/pectin methylesterase inhibitor family protein | LOC_Os02g01310.1 |
| 26.18 | misc.invertase/pectin methylesterase inhibitor family protein | LOC_Os05g20570.1 |
| 26.18 | misc.invertase/pectin methylesterase inhibitor family protein | LOC_Os11g45220.1 |
| 26.18 | misc.invertase/pectin methylesterase inhibitor family protein | LOC_Os12g37480.1 |
| 26.18 | misc.invertase/pectin methylesterase inhibitor family protein | LOC_Os11g08680.1 |
| 26.18 | misc.invertase/pectin methylesterase inhibitor family protein | LOC_Os05g29740.1 |
| 26.18 | misc.invertase/pectin methylesterase inhibitor family protein | LOC_Os01g20970.1 |
| 26.18 | misc.invertase/pectin methylesterase inhibitor family protein | LOC_Os12g03510.1 |
| 26.18 | misc.invertase/pectin methylesterase inhibitor family protein | LOC_Os03g61510.1 |
| 26.18 | misc.invertase/pectin methylesterase inhibitor family protein | LOC_Os05g46530.1 |
| Bin.Code | Bin.Name | Locus_ID |
| 26.18 | misc.invertase/pectin methylesterase inhibitor family protein | LOC_Os03g61530.1 |
| 26.18 | misc.invertase/pectin methylesterase inhibitor family protein | LOC_Os07g14340.1 |
| 26.18 | misc.invertase/pectin methylesterase inhibitor family protein | LOC_Os10g10700.1 |
| 26.18 | misc.invertase/pectin methylesterase inhibitor family protein | LOC_Os05g05640.1 |
| 26.19 | misc.plastocyanin-like | LOC_Os03g44630.1 |
| 26.19 | misc.plastocyanin-like | LOC_Os02g49350.1 |
| 26.19 | misc.plastocyanin-like | LOC_Os04g57750.1 |
| 26.19 | misc.plastocyanin-like | LOC_Os05g49580.1 |
| 26.22 | misc.short chain dehydrogenase/reductase (SDR) | LOC_Os06g03830.1 |
| 26.28 | misc.GDSL-motif lipase | LOC_Os03g38390.1 |
| 27.1 | RNA.processing | LOC_Os09g03890.1 |
| 27.1 | RNA.processing | LOC_Os03g17030.1 |
| 27.3.3 | RNA.regulation of transcription.AP2/EREBP, APETALA2/Ethylene-responsive element binding protein family | LOC_Os04g37520.1 |
| 27.3.7 | RNA.regulation of transcription.C2C2(Zn) CO-like, Constans-like zinc finger family | LOC_Os03g04620.1 |
| 27.3.11 | RNA.regulation of transcription.C2H2 zinc finger family | LOC_Os07g31830.1 |
| 27.3.11 | RNA.regulation of transcription.C2H2 zinc finger family | LOC_Os05g31720.1 |
| 27.3.11 | RNA.regulation of transcription.C2H2 zinc finger family | LOC_Os04g46670.1 |
| 27.3.11 | RNA.regulation of transcription.C2H2 zinc finger family | LOC_Os02g57790.1 |
| 27.3.11 | RNA.regulation of transcription.C2H2 zinc finger family | LOC_Os03g17150.1 |
| 27.3.11 | RNA.regulation of transcription.C2H2 zinc finger family | LOC_Os07g01780.1 |
| 27.3.24 | RNA.regulation of transcription.MADS box transcription factor family | LOC_Os02g36924.1 |
| 27.3.24 | RNA.regulation of transcription.MADS box transcription factor family | LOC_Os08g38590.1 |
| 27.3.24 | RNA.regulation of transcription.MADS box transcription factor family | LOC_Os11g43740.1 |
| 27.3.24 | RNA.regulation of transcription.MADS box transcription factor family | LOC_Os06g11970.1 |
| 27.3.25 | RNA.regulation of transcription.MYB domain transcription factor family | LOC_Os06g46560.1 |
| 27.3.29 | RNA.regulation of transcription.TCP transcription factor family | LOC_Os02g51310.1 |
| Bin.Code | Bin.Name | Locus_ID |
| 27.3.32 | RNA.regulation of transcription.WRKY domain transcription factor family | LOC_Os09g09630.1 |
| 27.3.35 | RNA.regulation of transcription.bZIP transcription factor family | LOC_Os01g11350.1 |
| 27.3.37 | RNA.regulation of transcription.AS2,Lateral Organ Boundaries Gene Family | LOC_Os09g19950.1 |
| 27.3.37 | RNA.regulation of transcription.AS2,Lateral Organ Boundaries Gene Family | LOC_Os08g31080.1 |
| 27.3.38 | RNA.regulation of transcription.AT-rich interaction domain containing transcription factor family | LOC_Os02g48370.1 |
| 27.3.58 | RNA.regulation of transcription.LUG | LOC_Os03g64300.1 |
| 27.3.67 | RNA.regulation of transcription.putative transcription regulator | LOC_Os02g38040.1 |
| 27.3.99 | RNA.regulation of transcription.unclassified | LOC_Os11g32960.1 |
| 27.3.99 | RNA.regulation of transcription.unclassified | LOC_Os10g32900.1 |
| 27.3.99 | RNA.regulation of transcription.unclassified | LOC_Os05g51750.1 |
| 27.3.99 | RNA.regulation of transcription.unclassified | LOC_Os03g04620.1 |
| 27.3.99 | RNA.regulation of transcription.unclassified | LOC_Os10g25674.1 |
| 27.4 | RNA.RNA binding | LOC_Os04g53330.1 |
| 27.4 | RNA.RNA binding | LOC_Os07g01260.1 |
| 28.1 | DNA.synthesis/chromatin structure | LOC_Os02g51600.1 |
| 28.1 | DNA.synthesis/chromatin structure | LOC_Os06g11920.1 |
| 28.1 | DNA.synthesis/chromatin structure | LOC_Os01g59880.1 |
| 28.1 | DNA.synthesis/chromatin structure | LOC_Os09g32440.1 |
| 28.1 | DNA.synthesis/chromatin structure | LOC_Os03g52870.1 |
| 28.99 | DNA.unspecified | LOC_Os03g12180.1 |
| 28.99 | DNA.unspecified | LOC_Os08g34780.1 |
| 29.2.3 | protein.synthesis.initiation | LOC_Os01g40150.1 |
| 29.3.2 | protein.targeting.mitochondria | LOC_Os09g10740.1 |
| 29.3.3 | protein.targeting.chloroplast | LOC_Os03g62750.1 |
| 29.3.4 | protein.targeting.secretory pathway | LOC_Os08g36560.1 |
| 29.3.4.99 | protein.targeting.secretory pathway.unspecified | LOC_Os02g32740.1 |
| Bin.Code | Bin.Name | Locus_ID |
| 29.3.4.99 | protein.targeting.secretory pathway.unspecified | LOC_Os03g59660.1 |
| 29.4 | protein.postranslational modification | LOC_Os06g48300.1 |
| 29.4 | protein.postranslational modification | LOC_Os12g42660.1 |
| 29.4 | protein.postranslational modification | LOC_Os03g57510.1 |
| 29.4 | protein.postranslational modification | LOC_Os02g35300.1 |
| 29.4 | protein.postranslational modification | LOC_Os05g39000.1 |
| 29.4 | protein.postranslational modification | LOC_Os12g12860.1 |
| 29.4 | protein.postranslational modification | LOC_Os08g39460.1 |
| 29.4 | protein.postranslational modification | LOC_Os07g48790.1 |
| 29.4 | protein.postranslational modification | LOC_Os01g55440.1 |
| 29.4 | protein.postranslational modification | LOC_Os05g40180.1 |
| 29.4 | protein.postranslational modification | LOC_Os01g13270.1 |
| 29.4 | protein.postranslational modification | LOC_Os10g33650.1 |
| 29.4 | protein.postranslational modification | LOC_Os01g10890.1 |
| 29.4 | protein.postranslational modification | LOC_Os05g14750.1 |
| 29.4 | protein.postranslational modification | LOC_Os01g61910.1 |
| 29.4 | protein.postranslational modification | LOC_Os10g33640.1 |
| 29.4 | protein.postranslational modification | LOC_Os05g50830.1 |
| 29.4 | protein.postranslational modification | LOC_Os11g13860.1 |
| 29.4 | protein.postranslational modification | LOC_Os08g35440.1 |
| 29.4 | protein.postranslational modification | LOC_Os01g74510.1 |
| 29.4 | protein.postranslational modification | LOC_Os09g33910.1 |
| 29.4 | protein.postranslational modification | LOC_Os12g10190.1 |
| 29.4 | protein.postranslational modification | LOC_Os07g02780.1 |
| 29.4 | protein.postranslational modification | LOC_Os05g11790.1 |
| 29.4 | protein.postranslational modification | LOC_Os03g53410.1 |
| Bin.Code | Bin.Name | Locus_ID |
| 29.4 | protein.postranslational modification | LOC_Os07g08000.1 |
| 29.4.1.57 | protein.postranslational modification.kinase.receptor like cytoplasmatic kinase VII | LOC_Os01g39970.1 |
| 29.4.1.57 | protein.postranslational modification.kinase.receptor like cytoplasmatic kinase VII | LOC_Os01g21970.1 |
| 29.4.1.57 | protein.postranslational modification.kinase.receptor like cytoplasmatic kinase VII | LOC_Os02g35760.1 |
| 29.4.1.57 | protein.postranslational modification.kinase.receptor like cytoplasmatic kinase VII | LOC_Os05g41950.1 |
| 29.4.1.57 | protein.postranslational modification.kinase.receptor like cytoplasmatic kinase VII | LOC_Os02g12660.1 |
| 29.4.1.57 | protein.postranslational modification.kinase.receptor like cytoplasmatic kinase VII | LOC_Os03g37120.1 |
| 29.4.1.57 | protein.postranslational modification.kinase.receptor like cytoplasmatic kinase VII | LOC_Os02g58610.1 |
| 29.4.1.57 | protein.postranslational modification.kinase.receptor like cytoplasmatic kinase VII | LOC_Os06g48980.1 |
| 29.4.1.57 | protein.postranslational modification.kinase.receptor like cytoplasmatic kinase VII | LOC_Os03g12520.1 |
| 29.4.1.57 | protein.postranslational modification.kinase.receptor like cytoplasmatic kinase VII | LOC_Os11g42440.1 |
| 29.4.1.57 | protein.postranslational modification.kinase.receptor like cytoplasmatic kinase VII | LOC_Os02g05820.1 |
| 29.4.1.57 | protein.postranslational modification.kinase.receptor like cytoplasmatic kinase VII | LOC_Os06g03610.1 |
| 29.4.1.57 | protein.postranslational modification.kinase.receptor like cytoplasmatic kinase VII | LOC_Os05g38770.1 |
| 29.4.1.57 | protein.postranslational modification.kinase.receptor like cytoplasmatic kinase VII | LOC_Os03g12570.1 |
| 29.4.1.57 | protein.postranslational modification.kinase.receptor like cytoplasmatic kinase VII | LOC_Os06g29080.1 |
| 29.4.1.57 | protein.postranslational modification.kinase.receptor like cytoplasmatic kinase VII | LOC_Os09g38700.1 |
| 29.4.1.57 | protein.postranslational modification.kinase.receptor like cytoplasmatic kinase VII | LOC_Os01g57940.1 |
| 29.4.1.57 | protein.postranslational modification.kinase.receptor like cytoplasmatic kinase VII | LOC_Os01g14926.1 |
| 29.4.1.57 | protein.postranslational modification.kinase.receptor like cytoplasmatic kinase VII | LOC_Os09g39930.1 |
| 29.4.1.57 | protein.postranslational modification.kinase.receptor like cytoplasmatic kinase VII | LOC_Os10g01560.1 |
| 29.4.1.57 | protein.postranslational modification.kinase.receptor like cytoplasmatic kinase VII | LOC_Os05g20150.1 |
| 29.5 | protein.degradation | LOC_Os01g68620.1 |
| 29.5.1 | protein.degradation.subtilases | LOC_Os04g47150.1 |
| 29.5.1 | protein.degradation.subtilases | LOC_Os02g44590.1 |
| 29.5.3 | protein.degradation.cysteine protease | LOC_Os09g21370.1 |
| Bin.Code | Bin.Name | Locus_ID |
| 29.5.3 | protein.degradation.cysteine protease | LOC_Os01g67490.1 |
| 29.5.5 | protein.degradation.serine protease | LOC_Os03g09190.1 |
| 29.5.11 | protein.degradation.ubiquitin | LOC_Os01g16470.1 |
| 29.5.11 | protein.degradation.ubiquitin | LOC_Os05g51230.1 |
| 29.5.11 | protein.degradation.ubiquitin | LOC_Os08g38200.1 |
| 29.5.11.1 | protein.degradation.ubiquitin.ubiquitin | LOC_Os07g30640.1 |
| 29.5.11.4.2 | protein.degradation.ubiquitin.E3.RING | LOC_Os06g40570.1 |
| 29.5.11.4.2 | protein.degradation.ubiquitin.E3.RING | LOC_Os08g33860.1 |
| 29.5.11.4.2 | protein.degradation.ubiquitin.E3.RING | LOC_Os08g42740.2 |
| 29.5.11.4.2 | protein.degradation.ubiquitin.E3.RING | LOC_Os02g10630.1 |
| 29.5.11.4.2 | protein.degradation.ubiquitin.E3.RING | LOC_Os04g41250.1 |
| 29.5.11.4.2 | protein.degradation.ubiquitin.E3.RING | LOC_Os08g37570.1 |
| 29.5.11.4.2 | protein.degradation.ubiquitin.E3.RING | LOC_Os01g68900.1 |
| 29.5.11.4.2 | protein.degradation.ubiquitin.E3.RING | LOC_Os06g51130.1 |
| 30.1 | signalling.in sugar and nutrient physiology | LOC_Os06g43000.1 |
| 30.1 | signalling.in sugar and nutrient physiology | LOC_Os02g49340.1 |
| 30.1 | signalling.in sugar and nutrient physiology | LOC_Os04g52950.1 |
| 30.2 | signalling.receptor kinases | LOC_Os02g38040.1 |
| 30.2.3 | signalling.receptor kinases.leucine rich repeat III | LOC_Os08g40990.1 |
| 30.2.3 | signalling.receptor kinases.leucine rich repeat III | LOC_Os06g09860.1 |
| 30.2.3 | signalling.receptor kinases.leucine rich repeat III | LOC_Os06g45240.1 |
| 30.2.3 | signalling.receptor kinases.leucine rich repeat III | LOC_Os11g40550.1 |
| 30.2.16 | signalling.receptor kinases.Catharanthus roseus-like RLK1 | LOC_Os06g03610.1 |
| 30.2.16 | signalling.receptor kinases.Catharanthus roseus-like RLK1 | LOC_Os05g20150.1 |
| 30.2.99 | signalling.receptor kinases.misc | LOC_Os02g54590.1 |
| 30.2.99 | signalling.receptor kinases.misc | LOC_Os03g08610.1 |
| Bin.Code | Bin.Name | Locus_ID |
| 30.2.99 | signalling.receptor kinases.misc | LOC_Os01g10900.1 |
| 30.3 | signalling.calcium | LOC_Os04g58480.1 |
| 30.3 | signalling.calcium | LOC_Os11g46230.1 |
| 30.3 | signalling.calcium | LOC_Os10g27170.1 |
| 30.3 | signalling.calcium | LOC_Os04g57350.1 |
| 30.3 | signalling.calcium | LOC_Os05g45810.1 |
| 30.3 | signalling.calcium | LOC_Os08g44660.1 |
| 30.3 | signalling.calcium | LOC_Os11g04170.1 |
| 30.3 | signalling.calcium | LOC_Os01g71240.1 |
| 30.3 | signalling.calcium | LOC_Os05g41270.1 |
| 30.3 | signalling.calcium | LOC_Os12g30150.1 |
| 30.3 | signalling.calcium | LOC_Os01g59360.1 |
| 30.3 | signalling.calcium | LOC_Os02g58520.1 |
| 30.3 | signalling.calcium | LOC_Os08g04890.1 |
| 30.3 | signalling.calcium | LOC_Os12g12730.1 |
| 30.4 | signalling.phosphinositides | LOC_Os11g20384.1 |
| 30.4 | signalling.phosphinositides | LOC_Os01g16470.1 |
| 30.4.1 | signalling.phosphinositides.phosphatidylinositol-4-phosphate 5-kinase | LOC_Os11g04840.1 |
| 30.4.1 | signalling.phosphinositides.phosphatidylinositol-4-phosphate 5-kinase | LOC_Os03g24160.1 |
| 30.4.1 | signalling.phosphinositides.phosphatidylinositol-4-phosphate 5-kinase | LOC_Os03g24170.1 |
| 30.5 | signalling.G-proteins | LOC_Os02g17240.1 |
| 30.5 | signalling.G-proteins | LOC_Os03g16900.1 |
| 30.5 | signalling.G-proteins | LOC_Os05g48640.1 |
| 30.5 | signalling.G-proteins | LOC_Os07g34130.1 |
| 30.5 | signalling.G-proteins | LOC_Os03g11140.1 |
| 30.5 | signalling.G-proteins | LOC_Os01g68540.1 |
| Bin.Code | Bin.Name | Locus_ID |
| 30.5 | signalling.G-proteins | LOC_Os01g55520.1 |
| 30.5 | signalling.G-proteins | LOC_Os01g48410.1 |
| 30.6 | signalling.MAP kinases | LOC_Os04g59310.1 |
| 30.6 | signalling.MAP kinases | LOC_Os05g50120.1 |
| 30.6 | signalling.MAP kinases | LOC_Os01g45620.1 |
| 30.99 | signalling.unspecified | LOC_Os04g47320.1 |
| 30.99 | signalling.unspecified | LOC_Os03g11140.1 |
| 31.1 | cell.organisation | LOC_Os10g25560.1 |
| 31.1 | cell.organisation | LOC_Os04g51440.1 |
| 31.1 | cell.organisation | LOC_Os06g29350.1 |
| 31.1 | cell.organisation | LOC_Os01g71780.1 |
| 31.1 | cell.organisation | LOC_Os02g44470.1 |
| 31.1 | cell.organisation | LOC_Os02g03400.1 |
| 31.1 | cell.organisation | LOC_Os02g37580.1 |
| 31.1 | cell.organisation | LOC_Os10g17660.1 |
| 31.1 | cell.organisation | LOC_Os11g23220.1 |
| 31.1 | cell.organisation | LOC_Os03g45920.1 |
| 31.1 | cell.organisation | LOC_Os02g13570.1 |
| 31.1 | cell.organisation | LOC_Os04g46910.1 |
| 31.1 | cell.organisation | LOC_Os11g44880.1 |
| 31.1 | cell.organisation | LOC_Os06g10480.1 |
| 31.1 | cell.organisation | LOC_Os02g13580.1 |
| 31.2 | cell.division | LOC_Os01g50470.1 |
| 31.2 | cell.division | LOC_Os01g72280.1 |
| 31.2 | cell.division | LOC_Os05g46740.1 |
| 31.2.5 | cell.division.plastid | LOC_Os03g62750.1 |
| Bin.Code | Bin.Name | Locus_ID |
| 31.3 | cell.cycle | LOC_Os07g10550.1 |
| 31.3 | cell.cycle | LOC_Os02g05040.1 |
| 31.3 | cell.cycle | LOC_Os02g53990.1 |
| 31.3.1 | cell.cycle.peptidylprolyl isomerase | LOC_Os06g49470.1 |
| 31.4 | cell.vesicle transport | LOC_Os11g06700.1 |
| 31.4 | cell.vesicle transport | LOC_Os02g32740.1 |
| 31.4 | cell.vesicle transport | LOC_Os02g55990.1 |
| 31.4 | cell.vesicle transport | LOC_Os06g39050.1 |
| 31.4 | cell.vesicle transport | LOC_Os10g40140.1 |
| 31.4 | cell.vesicle transport | LOC_Os12g06840.1 |
| 33.1 | development.storage proteins | LOC_Os01g74480.1 |
| 33.1 | development.storage proteins | LOC_Os03g27610.1 |
| 33.2 | development.late embryogenesis abundant | LOC_Os07g17120.1 |
| 33.99 | development.unspecified | LOC_Os12g41170.1 |
| 33.99 | development.unspecified | LOC_Os07g30210.1 |
| 33.99 | development.unspecified | LOC_Os11g08400.1 |
| 33.99 | development.unspecified | LOC_Os05g51090.1 |
| 33.99 | development.unspecified | LOC_Os11g37720.1 |
| 33.99 | development.unspecified | LOC_Os12g07874.1 |
| 33.99 | development.unspecified | LOC_Os02g50150.1 |
| 33.99 | development.unspecified | LOC_Os04g45010.1 |
| 33.99 | development.unspecified | LOC_Os02g42820.1 |
| 33.99 | development.unspecified | LOC_Os02g50140.1 |
| 33.99 | development.unspecified | LOC_Os04g02690.1 |
| 33.99 | development.unspecified | LOC_Os03g06760.1 |
| 33.99 | development.unspecified | LOC_Os10g35930.1 |
| Bin.Code | Bin.Name | Locus_ID |
| 33.99 | development.unspecified | LOC_Os07g17310.1 |
| 34.1 | transport.p- and v-ATPases | LOC_Os06g08310.1 |
| 34.1 | transport.p- and v-ATPases | LOC_Os02g55400.1 |
| 34.1 | transport.p- and v-ATPases | LOC_Os05g45370.1 |
| 34.1.1.4 | transport.p- and v-ATPases.H+-transporting two-sector ATPase.subunit E | LOC_Os05g40230.1 |
| 34.2 | transport.sugars | LOC_Os02g06540.1 |
| 34.2 | transport.sugars | LOC_Os12g32760.1 |
| 34.2 | transport.sugars | LOC_Os08g08070.1 |
| 34.2 | transport.sugars | LOC_Os11g28610.1 |
| 34.2.1 | transport.sugars.sucrose | LOC_Os10g26470.1 |
| 34.3 | transport.amino acids | LOC_Os02g49510.1 |
| 34.3 | transport.amino acids | LOC_Os12g38570.1 |
| 34.3 | transport.amino acids | LOC_Os06g36210.1 |
| 34.6 | transport.sulphate | LOC_Os06g05160.1 |
| 34.9 | transport.metabolite transporters at the mitochondrial membrane | LOC_Os01g12680.2 |
| 34.1 | transport.nucleotides | LOC_Os04g49739.1 |
| 34.12 | transport.metal | LOC_Os05g19500.1 |
| 34.12 | transport.metal | LOC_Os01g60140.1 |
| 34.12 | transport.metal | LOC_Os12g44300.1 |
| 34.12 | transport.metal | LOC_Os09g37300.1 |
| 34.12 | transport.metal | LOC_Os11g01820.1 |
| 34.12 | transport.metal | LOC_Os05g39600.1 |
| 34.12 | transport.metal | LOC_Os08g02450.1 |
| 34.12 | transport.metal | LOC_Os02g58660.1 |
| 34.12 | transport.metal | LOC_Os05g35050.1 |
| 34.12 | transport.metal | LOC_Os05g40650.1 |
| Bin.Code | Bin.Name | Locus_ID |
| 34.13 | transport.peptides and oligopeptides | LOC_Os02g02460.1 |
| 34.13 | transport.peptides and oligopeptides | LOC_Os01g13710.1 |
| 34.13 | transport.peptides and oligopeptides | LOC_Os01g61390.1 |
| 34.13 | transport.peptides and oligopeptides | LOC_Os05g16290.1 |
| 34.13 | transport.peptides and oligopeptides | LOC_Os02g02450.1 |
| 34.14 | transport.unspecified cations | LOC_Os04g51830.1 |
| 34.14 | transport.unspecified cations | LOC_Os08g39370.1 |
| 34.15 | transport.potassium | LOC_Os02g31940.1 |
| 34.16 | transport.ABC transporters and multidrug resistance systems | LOC_Os11g22350.1 |
| 34.16 | transport.ABC transporters and multidrug resistance systems | LOC_Os06g51460.1 |
| 34.16 | transport.ABC transporters and multidrug resistance systems | LOC_Os04g40570.1 |
| 34.19.2 | transport.Major Intrinsic Proteins.TIP | LOC_Os02g44080.1 |
| 34.19.2 | transport.Major Intrinsic Proteins.TIP | LOC_Os01g74450.1 |
| 34.19.2 | transport.Major Intrinsic Proteins.TIP | LOC_Os04g46490.1 |
| 34.21 | transport.calcium | LOC_Os02g04630.1 |
| 34.22 | transport.cyclic nucleotide or calcium regulated channels | LOC_Os12g06570.1 |
| 34.22 | transport.cyclic nucleotide or calcium regulated channels | LOC_Os03g44440.1 |
| 34.22 | transport.cyclic nucleotide or calcium regulated channels | LOC_Os12g28260.1 |
| 34.99 | transport.misc | LOC_Os08g36560.1 |
| 34.99 | transport.misc | LOC_Os11g32490.1 |
| 34.99 | transport.misc | LOC_Os08g06440.1 |
| 34.99 | transport.misc | LOC_Os02g24430.1 |
| 35.2 | not assigned.unknown | LOC_Os12g30550.1 |
| 35.2 | not assigned.unknown | LOC_Os04g33710.1 |
| 35.2 | not assigned.unknown | LOC_Os01g09730.1 |
| 35.2 | not assigned.unknown | LOC_Os05g50170.1 |
| Bin.Code | Bin.Name | Locus_ID |
| 35.2 | not assigned.unknown | LOC_Os08g41650.1 |
| 35.2 | not assigned.unknown | LOC_Os04g21570.1 |
| 35.2 | not assigned.unknown | LOC_Os09g28450.1 |
| 35.2 | not assigned.unknown | LOC_Os01g46850.1 |
| 35.2 | not assigned.unknown | LOC_Os11g40800.1 |
| 35.2 | not assigned.unknown | LOC_Os10g33240.1 |
| 35.2 | not assigned.unknown | LOC_Os10g40170.1 |
| 35.2 | not assigned.unknown | LOC_Os05g05650.1 |
| 35.2 | not assigned.unknown | LOC_Os02g50980.1 |
| 35.2 | not assigned.unknown | LOC_Os05g38790.1 |
| 35.2 | not assigned.unknown | LOC_Os07g03200.1 |
| 35.2 | not assigned.unknown | LOC_Os04g20420.1 |
| 35.2 | not assigned.unknown | LOC_Os07g20270.1 |
| 35.2 | not assigned.unknown | LOC_Os04g57270.1 |
| 35.2 | not assigned.unknown | LOC_Os03g53550.1 |
| 35.2 | not assigned.unknown | LOC_Os10g39020.1 |
| 35.2 | not assigned.unknown | LOC_Os01g26290.1 |
| 35.2 | not assigned.unknown | LOC_Os05g35160.1 |
| 35.2 | not assigned.unknown | LOC_Os05g30940.1 |
| 35.2 | not assigned.unknown | LOC_Os05g23194.1 |
| 35.2 | not assigned.unknown | LOC_Os01g38500.1 |
| 35.2 | not assigned.unknown | LOC_Os04g30300.1 |
| 35.2 | not assigned.unknown | LOC_Os03g64310.1 |
| 35.2 | not assigned.unknown | LOC_Os05g41150.1 |
| 35.2 | not assigned.unknown | LOC_Os03g61590.1 |
| 35.2 | not assigned.unknown | LOC_Os09g22000.1 |
| Bin.Code | Bin.Name | Locus_ID |
| 35.2 | not assigned.unknown | LOC_Os12g43990.1 |
| 35.2 | not assigned.unknown | LOC_Os04g49650.1 |
| 35.2 | not assigned.unknown | LOC_Os05g10800.1 |
| 35.2 | not assigned.unknown | LOC_Os04g21340.1 |
| 35.2 | not assigned.unknown | LOC_Os07g08060.1 |
| 35.2 | not assigned.unknown | LOC_Os02g05670.1 |
| 35.2 | not assigned.unknown | LOC_Os11g29210.1 |
| 35.2 | not assigned.unknown | LOC_Os04g54600.1 |
| 35.2 | not assigned.unknown | LOC_Os10g04370.1 |
| 35.2 | not assigned.unknown | LOC_Os07g39930.1 |
| 35.2 | not assigned.unknown | LOC_Os06g45184.1 |
| 35.2 | not assigned.unknown | LOC_Os03g25460.1 |
| 35.2 | not assigned.unknown | LOC_Os03g18360.1 |
| 35.2 | not assigned.unknown | LOC_Os08g02880.1 |
| 35.2 | not assigned.unknown | LOC_Os12g32310.1 |
| 35.2 | not assigned.unknown | LOC_Os07g09384.1 |
| 35.2 | not assigned.unknown | LOC_Os05g33580.1 |
| 35.2 | not assigned.unknown | LOC_Os08g12890.1 |
| 35.2 | not assigned.unknown | LOC_Os10g11750.1 |
| 35.2 | not assigned.unknown | LOC_Os02g03510.1 |
| 35.2 | not assigned.unknown | LOC_Os08g45230.1 |
| 35.2 | not assigned.unknown | LOC_Os07g13160.1 |
| 35.2 | not assigned.unknown | LOC_Os02g04210.1 |
| 35.2 | not assigned.unknown | LOC_Os01g71040.1 |
| 35.2 | not assigned.unknown | LOC_Os07g26480.1 |
| 35.2 | not assigned.unknown | LOC_Os08g30800.1 |
| Bin.Code | Bin.Name | Locus_ID |
| 35.2 | not assigned.unknown | LOC_Os02g42710.1 |
| 35.2 | not assigned.unknown | LOC_Os05g13830.1 |
| 35.2 | not assigned.unknown | LOC_Os09g30030.1 |
| 35.2 | not assigned.unknown | LOC_Os03g17270.1 |
| 35.2 | not assigned.unknown | LOC_Os03g27900.1 |
| 35.2 | not assigned.unknown | LOC_Os01g11800.1 |
| 35.2 | not assigned.unknown | LOC_Os07g18070.1 |
| 35.2 | not assigned.unknown | LOC_Os01g27190.1 |
| 35.2 | not assigned.unknown | LOC_Os02g55649.1 |
| 35.2 | not assigned.unknown | LOC_Os11g36230.1 |
| 35.2 | not assigned.unknown | LOC_Os04g29050.1 |
| 35.2 | not assigned.unknown | LOC_Os08g38280.1 |
| 35.2 | not assigned.unknown | LOC_Os12g22920.1 |
| 35.2 | not assigned.unknown | LOC_Os02g02560.1 |
| 35.2 | not assigned.unknown | LOC_Os08g12160.1 |
| 35.2 | not assigned.unknown | LOC_Os02g26390.1 |
| 35.2 | not assigned.unknown | LOC_Os02g07580.1 |
| 35.2 | not assigned.unknown | LOC_Os03g08980.1 |
| 35.2 | not assigned.unknown | LOC_Os02g03480.1 |
| 35.2 | not assigned.unknown | LOC_Os03g07600.1 |
| 35.2 | not assigned.unknown | LOC_Os09g25650.1 |
| 35.2 | not assigned.unknown | LOC_Os11g11730.1 |
| 35.2 | not assigned.unknown | LOC_Os02g10530.1 |
| 35.2 | not assigned.unknown | LOC_Os05g20914.1 |
| 35.2 | not assigned.unknown | LOC_Os11g31400.1 |
| 35.2 | not assigned.unknown | LOC_Os08g34340.1 |
| Bin.Code | Bin.Name | Locus_ID |
| 35.2 | not assigned.unknown | LOC_Os09g12620.1 |
| 35.2 | not assigned.unknown | LOC_Os10g27370.1 |
| 35.2 | not assigned.unknown | LOC_Os01g23880.1 |
| 35.2 | not assigned.unknown | LOC_Os01g55850.1 |
| 35.2 | not assigned.unknown | LOC_Os01g10470.1 |
| 35.2 | not assigned.unknown | LOC_Os05g51900.1 |
| 35.2 | not assigned.unknown | LOC_Os02g01290.1 |
| 35.2 | not assigned.unknown | LOC_Os11g29780.1 |
| 35.2 | not assigned.unknown | LOC_Os03g51210.1 |
| 35.2 | not assigned.unknown | LOC_Os04g46079.1 |
| 35.2 | not assigned.unknown | LOC_Os02g38170.1 |
| 35.2 | not assigned.unknown | LOC_Os04g10500.1 |
| 35.2 | not assigned.unknown | LOC_Os03g27110.1 |
| 35.2 | not assigned.unknown | LOC_Os01g14940.1 |
| 35.2 | not assigned.unknown | LOC_Os09g27710.1 |
| 35.2 | not assigned.unknown | LOC_Os02g26290.1 |
| 35.2 | not assigned.unknown | LOC_Os09g16970.1 |
| 35.2 | not assigned.unknown | LOC_Os06g43060.1 |
| 35.2 | not assigned.unknown | LOC_Os05g11330.1 |
| 35.2 | not assigned.unknown | LOC_Os01g70120.1 |
| 35.2 | not assigned.unknown | LOC_Os03g58140.1 |
| 35.2 | not assigned.unknown | LOC_Os03g62210.1 |
| 35.2 | not assigned.unknown | LOC_Os02g03520.1 |
| 35.2 | not assigned.unknown | LOC_Os03g01930.1 |
| 35.2 | not assigned.unknown | LOC_Os04g42960.1 |
| 35.2 | not assigned.unknown | LOC_Os02g54100.1 |
| Bin.Code | Bin.Name | Locus_ID |
| 35.2 | not assigned.unknown | LOC_Os07g13450.1 |
| 35.2 | not assigned.unknown | LOC_Os06g44810.1 |
| 35.2 | not assigned.unknown | LOC_Os04g42210.1 |
| 35.2 | not assigned.unknown | LOC_Os05g11720.1 |
| 35.2 | not assigned.unknown | LOC_Os07g28920.1 |
| 35.2 | not assigned.unknown | LOC_Os10g34680.1 |
| 35.2 | not assigned.unknown | LOC_Os03g13120.1 |
| 35.2 | not assigned.unknown | LOC_Os11g16350.1 |
| 35.2 | not assigned.unknown | LOC_Os04g57280.1 |
| 35.2 | not assigned.unknown | LOC_Os06g05710.1 |
| 35.2 | not assigned.unknown | LOC_Os07g37480.1 |
| 35.2 | not assigned.unknown | LOC_Os12g38260.1 |
| 35.2 | not assigned.unknown | LOC_Os03g58190.1 |
| 35.2 | not assigned.unknown | LOC_Os04g11130.1 |
| 35.2 | not assigned.unknown | LOC_Os01g02050.1 |
| 35.2 | not assigned.unknown | LOC_Os07g15530.1 |
| 35.2 | not assigned.unknown | LOC_Os01g42060.1 |
| 35.2 | not assigned.unknown | LOC_Os03g43100.2 |
| 35.2 | not assigned.unknown | LOC_Os02g32210.1 |
| 35.2 | not assigned.unknown | LOC_Os02g58800.1 |
| 35.2 | not assigned.unknown | LOC_Os10g10760.1 |
| 35.2 | not assigned.unknown | LOC_Os09g23899.1 |
| 35.2 | not assigned.unknown | LOC_Os06g05730.1 |
| 35.2 | not assigned.unknown | LOC_Os11g05970.1 |
| 35.2 | not assigned.unknown | LOC_Os01g23370.1 |
| 35.2 | not assigned.unknown | LOC_Os01g05100.1 |
| Bin.Code | Bin.Name | Locus_ID |
| 35.2 | not assigned.unknown | LOC_Os01g13440.1 |
| 35.2 | not assigned.unknown | LOC_Os02g03490.1 |
| 35.2 | not assigned.unknown | LOC_Os03g49270.1 |
| 35.2 | not assigned.unknown | LOC_Os08g07600.1 |
| 35.2 | not assigned.unknown | LOC_Os03g49590.1 |
| 35.2 | not assigned.unknown | LOC_Os05g40310.1 |
| 35.2 | not assigned.unknown | LOC_Os06g17450.1 |
| 35.2 | not assigned.unknown | LOC_Os11g06690.1 |
| 35.2 | not assigned.unknown | LOC_Os03g09160.1 |
| 35.2 | not assigned.unknown | LOC_Os07g07130.1 |
| 35.2 | not assigned.unknown | LOC_Os04g01330.1 |
| 35.2 | not assigned.unknown | LOC_Os06g03980.1 |
| 35.2 | not assigned.unknown | LOC_Os11g05950.1 |
| 35.2 | not assigned.unknown | LOC_Os12g42650.1 |
| 35.2 | not assigned.unknown | LOC_Os11g09030.1 |
| 35.2 | not assigned.unknown | LOC_Os04g58100.1 |
| 35.2 | not assigned.unknown | LOC_Os11g36740.1 |
| 35.2 | not assigned.unknown | LOC_Os08g35770.1 |
| 35.2 | not assigned.unknown | LOC_Os03g61410.1 |
| 35.2 | not assigned.unknown | LOC_Os12g35690.1 |
| 35.2 | not assigned.unknown | LOC_Os01g65860.1 |
| 35.2 | not assigned.unknown | LOC_Os02g44120.1 |
| 35.2 | not assigned.unknown | LOC_Os08g12520.1 |
| 35.2 | not assigned.unknown | LOC_Os10g20550.1 |
| 35.2 | not assigned.unknown | LOC_Os11g11710.1 |
| 35.2 | not assigned.unknown | LOC_Os04g01150.1 |
| Bin.Code | Bin.Name | Locus_ID |
| 35.2 | not assigned.unknown | LOC_Os06g48050.1 |
| 35.2 | not assigned.unknown | LOC_Os11g08707.1 |
| 35.2 | not assigned.unknown | LOC_Os02g36950.1 |
| 35.2 | not assigned.unknown | LOC_Os04g55110.1 |
| 35.2 | not assigned.unknown | LOC_Os11g11240.1 |
| 35.2 | not assigned.unknown | LOC_Os10g39950.1 |
| 35.2 | not assigned.unknown | LOC_Os11g30350.1 |
| 35.2 | not assigned.unknown | LOC_Os01g69020.1 |
| 35.2 | not assigned.unknown | LOC_Os05g51860.1 |
| 35.2 | not assigned.unknown | LOC_Os06g44900.1 |
| 35.2 | not assigned.unknown | LOC_Os04g42220.1 |
| 35.2 | not assigned.unknown | LOC_Os02g57000.1 |
| 35.2 | not assigned.unknown | LOC_Os12g30500.1 |
| 35.2 | not assigned.unknown | LOC_Os02g41940.1 |
| 35.2 | not assigned.unknown | LOC_Os05g20620.1 |
| 35.2 | not assigned.unknown | LOC_Os06g40580.1 |
| 35.2 | not assigned.unknown | LOC_Os02g55500.1 |
| 35.2 | not assigned.unknown | LOC_Os09g12630.1 |
| 35.2 | not assigned.unknown | LOC_Os01g42024.1 |
| 35.2 | not assigned.unknown | LOC_Os02g06510.1 |
| 35.2 | not assigned.unknown | LOC_Os07g13440.1 |
| 35.2 | not assigned.unknown | LOC_Os03g07510.1 |
| 35.2 | not assigned.unknown | LOC_Os12g27190.1 |
| 35.2 | not assigned.unknown | LOC_Os10g27480.1 |
| 35.2 | not assigned.unknown | LOC_Os02g33740.1 |
| 35.2 | not assigned.unknown | LOC_Os08g43500.1 |
| Bin.Code | Bin.Name | Locus_ID |
| 35.2 | not assigned.unknown | LOC_Os08g38250.1 |
| 35.2 | not assigned.unknown | LOC_Os03g04560.1 |
| 35.2 | not assigned.unknown | LOC_Os06g44160.1 |
| 35.2 | not assigned.unknown | LOC_Os02g40550.1 |
| 35.2 | not assigned.unknown | LOC_Os03g10460.1 |
| 35.2 | not assigned.unknown | LOC_Os03g60470.1 |
| 35.2 | not assigned.unknown | LOC_Os02g09540.1 |
| 35.2 | not assigned.unknown | LOC_Os09g30040.1 |
| 35.2 | not assigned.unknown | LOC_Os12g43820.1 |
| 35.2 | not assigned.unknown | LOC_Os03g21880.1 |
| 35.2 | not assigned.unknown | LOC_Os09g27040.1 |
| 35.2 | not assigned.unknown | LOC_Os02g46180.1 |
| 35.2 | not assigned.unknown | LOC_Os08g07500.1 |
| 35.2 | not assigned.unknown | LOC_Os08g39530.1 |
| 35.2 | not assigned.unknown | LOC_Os05g38960.1 |
| 35.2 | not assigned.unknown | LOC_Os09g12510.1 |
| 35.2 | not assigned.unknown | LOC_Os01g06620.1 |
| 35.2 | not assigned.unknown | LOC_Os09g29990.1 |
| 35.2 | not assigned.unknown | LOC_Os09g09350.1 |
| 35.2 | not assigned.unknown | LOC_Os03g16420.1 |
| 35.2 | not assigned.unknown | LOC_Os08g04650.1 |
| 35.2 | not assigned.unknown | LOC_Os02g07900.1 |
| 35.2 | not assigned.unknown | LOC_Os06g03390.1 |
| 35.2 | not assigned.unknown | LOC_Os03g10334.1 |

\

**Table S6**. Genes related to hormone metabolism term in MapMan.

| Bin | putative function | Locus number |
| --- | --- | --- |
| 17.1.2 | hormone metabolism. abscisic acid. signal transduction | Loc_Os01g51610.1 |
| 17.2.1 | hormone metabolism. auxin. synthesis-degradation | Loc_Os01g51060.1 |
| 17.2.3 | hormone metabolism. auxin. induced-regulated-responsive-activated | Loc_Os01g55940.1 |
| 17.2.3 | hormone metabolism. auxin. induced-regulated-responsive-activated | Loc_Os05g37880.1 |
| 17.3.1.1.1 | hormone metabolism.brassinosteroid.synthesis-degradation.BRs.DET2 | Loc_Os07g06800.1 |
| 17.5.1 | hormone metabolism. ethylene. synthesis-degradation | Loc_Os02g52840.1 |
| 17.5.1 | hormone metabolism. ethylene. synthesis-degradation | Loc_Os05g08540.1 |
| 17.5.2 | hormone metabolism. ethylene. signal transduction | Loc_Os06g01780.1 |
| 17.5.2 | hormone metabolism. ethylene. signal transduction | Loc_Os02g43840.1 |
| 17.5.3 | hormone metabolism. ethylene. induced-regulated-responsive-activated | Loc_Os12g36630.1 |
| 17.6.1.11 | hormone metabolism.gibberelin.synthesis-degradation.GA20 oxidase | Loc_Os07g07420.1 |
| 17.6.1.13 | hormone metabolism.gibberelin.synthesis-degradation.GA2 oxidase | Loc_Os05g06670.1 |

**Table S7.** Late pollen-preferred genes in *Arabidopsis*.

| Locus id | TAIR Annotation |  |
| --- | --- | --- |
| AT2G10970 | Plant invertase/pectin methylesterase inhibitor superfamily protein |  |
| AT3G05150 | Major facilitator superfamily protein |  |
| AT1G51410 | NAD(P)-binding Rossmann-fold superfamily protein |  |
| AT1G52680 | late embryogenesis abundant protein-related / LEA protein-related |  |
| AT1G49270 | Protein kinase superfamily protein |  |
| AT1G03050 | ENTH/ANTH/VHS superfamily protein |  |
| AT1G30710 | FAD-binding Berberine family protein |  |
| AT2G04220 | Plant protein of unknown function (DUF868) |  |
| AT2G46360 | unknown protein |  |
| AT1G61290 | syntaxin of plants 124 |  |
| AT2G07560 | H(+)-ATPase 6 |  |
| AT2G03410 | Mo25 family protein |  |
| AT2G07040 | Leucine-rich repeat protein kinase family protein |  |
| AT2G46860 | pyrophosphorylase 3 |  |
| AT2G38500 | 2-oxoglutarate (2OG) and Fe(II)-dependent oxygenase superfamily protein |  |
| AT2G44560 | glycosyl hydrolase 9B11 |  |
| AT2G41860 | calcium-dependent protein kinase 14 |  |
| AT4G25590 | actin depolymerizing factor 7 |  |
| AT5G10660 | calmodulin-binding protein-related |  |
| AT5G20460 | unknown protein |  |
| AT5G28680 | Malectin/receptor-like protein kinase family protein |  |
| AT5G36260 | Eukaryotic aspartyl protease family protein |  |
| AT5G26060 | Plant self-incompatibility protein S1 family |  |
| Locus id | TAIR Annotation |  |
| AT5G24940 | Protein phosphatase 2C family protein |  |
| AT5G64690 | neurofilament triplet H protein-related |  |
| AT5G54095 | unknown protein |  |
| AT5G53820 | Late embryogenesis abundant protein (LEA) family protein |  |
| AT5G52360 | actin depolymerizing factor 10 |  |
| AT5G48270 | Plant protein of unknown function (DUF868) |  |
| AT5G46770 | unknown protein |  |
| AT5G46200 | Protein of Unknown Function (DUF239) |  |
| AT5G45810 | CBL-interacting protein kinase 19 |  |
| AT5G42490 | ATP binding microtubule motor family protein |  |
| AT5G40155 | Defensin-like (DEFL) family protein |  |
| AT5G39310 | expansin A24 |  |
| AT5G38760 | Late embryogenesis abundant protein (LEA) family protein |  |
| AT5G18910 | Protein kinase superfamily protein |  |
| AT5G16500 | Protein kinase superfamily protein |  |
| AT5G12180 | calcium-dependent protein kinase 17 |  |
| AT5G10090 | Tetratricopeptide repeat (TPR)-like superfamily protein |  |
| AT5G07420 | Pectin lyase-like superfamily protein |  |
| AT3G59830 | Integrin-linked protein kinase family |  |
| AT3G54240 | alpha/beta-Hydrolases superfamily protein |  |
| AT3G52620 | unknown protein |  |
| AT3G52000 | serine carboxypeptidase-like 36 |  |
| AT3G48010 | cyclic nucleotide-gated channel 16 |  |
| AT3G46520 | actin-12 |  |
| Locus id | TAIR Annotation |  |
| AT4G35700 | zinc finger (C2H2 type) family protein |  |
| AT4G26930 | myb domain protein 97 |  |
| AT4G24170 | ATP binding microtubule motor family protein |  |
| AT4G12860 | EF hand calcium-binding protein family |  |
| AT4G03290 | EF hand calcium-binding protein family |  |
| AT1G16760 | Protein kinase protein with adenine nucleotide alpha hydrolases-like domain |  |
| AT1G19890 | male-gamete-specific histone H3 |  |
| AT2G33320 | Calcium-dependent lipid-binding (CaLB domain) family protein |  |
| AT1G06970 | cation/hydrogen exchanger 14 |  |
| AT3G28770 | Protein of unknown function (DUF1216) |  |
| AT3G28780 | Protein of unknown function (DUF1216) |  |
| AT3G18220 | Phosphatidic acid phosphatase (PAP2) family protein |  |
| AT3G20530 | Protein kinase superfamily protein |  |
| AT3G20190 | Leucine-rich repeat protein kinase family protein |  |
| AT1G24620 | EF hand calcium-binding protein family |  |
| AT3G13065 | STRUBBELIG-receptor family 4 |  |
| AT3G26860 | Plant self-incompatibility protein S1 family |  |
| AT3G22650 | F-box and associated interaction domains-containing protein |  |
| AT3G06560 | poly(A) polymerase 3 |  |
| AT3G02810 | Protein kinase superfamily protein |  |
| AT3G04690 | Malectin/receptor-like protein kinase family protein |  |
| AT3G07490 | ARF-GAP domain 11 |  |
| AT3G01630 | Major facilitator superfamily protein |  |
| AT3G01020 | ISCU-like 2 |  |
| Locus id | TAIR Annotation |  |
| AT3G01250 | unknown protein |  |
| AT1G01460 | Phosphatidylinositol-4-phosphate 5-kinase, core |  |
| AT1G13890 | soluble N-ethylmaleimide-sensitive factor adaptor protein 30 |  |
| AT1G18990 | Protein of unknown function, DUF593 |  |
| AT1G77730 | Pleckstrin homology (PH) domain superfamily protein |  |
| AT1G52240 | RHO guanyl-nucleotide exchange factor 11 |  |
| AT1G76370 | Protein kinase superfamily protein |  |
| AT1G67623 | F-box family protein |  |
| AT1G74220 | unknown protein |  |
| AT1G70540 | Plant invertase/pectin methylesterase inhibitor superfamily protein |  |
| AT1G63930 | from the Czech 'roh' meaning 'corner' |  |
| AT1G51490 | beta glucosidase 36 |  |
| AT2G47340 | Plant invertase/pectin methylesterase inhibitor superfamily protein |  |
| AT1G26480 | general regulatory factor 12 |  |
| AT1G01310 | CAP (Cysteine-rich secretory proteins, Antigen 5, and Pathogenesis-related 1 protein) superfamily protein |  |
| AT1G19780 | cyclic nucleotide gated channel 8 |  |
| AT1G47280 | unknown protein |  |
| AT1G10620 | Protein kinase superfamily protein |  |
| AT1G80660 | H(+)-ATPase 9 |  |
| AT2G48150 | glutathione peroxidase 4 |  |
| AT1G28550 | RAB GTPase homolog A1I |  |
| AT2G13620 | cation/hydrogen exchanger 15 |  |
| AT1G60240 | NAC (No Apical Meristem) domain transcriptional regulator superfamily protein |  |
| Locus id | TAIR Annotation |  |
| AT1G11990 | O-fucosyltransferase family protein |  |
| AT1G08860 | Calcium-dependent phospholipid-binding Copine family protein |  |
| AT1G55410 |  |  |
| AT1G23540 | Protein kinase superfamily protein |  |
| AT2G19980 | CAP (Cysteine-rich secretory proteins, Antigen 5, and Pathogenesis-related 1 protein) superfamily protein |  |
| AT2G18080 | Serine carboxypeptidase S28 family protein |  |
| AT2G46300 | Late embryogenesis abundant (LEA) hydroxyproline-rich glycoprotein family |  |
| AT2G29790 | Maternally expressed gene (MEG) family protein |  |
| AT2G34440 | AGAMOUS-like 29 |  |
| AT2G33870 | RAB GTPase homolog A1H |  |
| AT4G15650 | unknown protein |  |
| AT5G13150 | exocyst subunit exo70 family protein C1 |  |
| AT5G17480 | pollen calcium-binding protein 1 |  |
| AT5G35090 | unknown protein |  |
| AT5G26150 | protein kinase family protein |  |
| AT5G62750 | unknown protein |  |
| AT5G61710 | unknown protein |  |
| AT5G41780 | myosin heavy chain-related |  |
| AT5G39650 | Protein of unknown function (DUF679) |  |
| AT5G10500 | Kinase interacting (KIP1-like) family protein |  |
| AT5G05070 | DHHC-type zinc finger family protein |  |
| AT3G60780 | Protein of unknown function (DUF1442) |  |
| AT3G60760 | unknown protein |  |
| Locus id | TAIR Annotation |  |
| AT3G42880 | Leucine-rich repeat protein kinase family protein |  |
| AT4G40020 | Myosin heavy chain-related protein |  |
| AT4G33860 | Glycosyl hydrolase family 10 protein |  |
| AT4G33390 | Plant protein of unknown function (DUF827) |  |
| AT4G31230 | Protein kinase protein with adenine nucleotide alpha hydrolases-like domain |  |
| AT4G21420 | gypsy-like retrotransposon family |  |
| AT4G04980 | unknown protein |  |
| AT4G04080 | ISCU-like 3 |  |
| AT3G12160 | RAB GTPase homolog A4D |  |
| AT3G19690 | CAP (Cysteine-rich secretory proteins, Antigen 5, and Pathogenesis-related 1 protein) superfamily protein |  |
| AT3G24280 | small acidic protein 2 |  |
| AT3G28490 | Oxoglutarate/iron-dependent oxygenase |  |
| AT3G24620 | RHO guanyl-nucleotide exchange factor 8 |  |
| AT3G07070 | Protein kinase superfamily protein |  |
| AT1G63060 | unknown protein |  |
| AT1G76640 | Calcium-binding EF-hand family protein |  |
| AT1G68090 | annexin 5 |  |
| AT1G67540 | unknown protein |  |
| AT1G06750 | P-loop containing nucleoside triphosphate hydrolases superfamily protein |  |
| AT1G20080 | Calcium-dependent lipid-binding (CaLB domain) family protein |  |
| AT2G40180 | phosphatase 2C5 |  |
| AT2G22290 | RAB GTPase homolog H1D |  |
| AT1G23210 | glycosyl hydrolase 9B6 |  |
| Locus id | TAIR Annotation |  |
| AT2G28440 | proline-rich family protein |  |
| AT2G15650 | copia-like retrotransposon family |  |
| AT2G42380 | Basic-leucine zipper (bZIP) transcription factor family protein |  |
| AT5G19610 | GNOM-like 2 |  |
| AT5G20330 | beta-1,3-glucanase 4 |  |
| AT3G44930 | cation/H+ exchanger 10 |  |
| AT5G44640 | beta glucosidase 13 |  |
| AT4G35650 | isocitrate dehydrogenase III |  |
| AT1G64320 | myosin heavy chain-related |  |
| AT3G10780 | emp24/gp25L/p24 family/GOLD family protein |  |
| AT1G12150 | Plant protein of unknown function (DUF827) |  |
| AT5G52340 | exocyst subunit exo70 family protein A2 |  |
| AT5G40645 | RPM1-interacting protein 4 (RIN4) family protein |  |
| AT4G03620 | myosin heavy chain-related |  |
| AT1G58210 | kinase interacting family protein |  |
| AT1G61080 | Hydroxyproline-rich glycoprotein family protein |  |
| AT2G40790 | C-terminal cysteine residue is changed to a serine 2 |  |
| AT3G52080 | cation/hydrogen exchanger 28 |  |
| AT4G12930 | unknown protein |  |
| AT3G07840 | Pectin lyase-like superfamily protein |  |
| AT1G54240 | winged-helix DNA-binding transcription factor family protein |  |
| AT5G53520 | oligopeptide transporter 8 |  |
| AT5G42880 | Plant protein of unknown function (DUF827) |  |
| AT4G28670 | Protein kinase family protein with domain of unknown function (DUF26) |  |
| Locus id | TAIR Annotation |  |
| AT4G20570 | Protein with domains of unknown function (DUF26 and DUF1204) |  |
| AT2G17090 | Protein kinase protein with tetratricopeptide repeat domain |  |
| AT2G15535 | low-molecular-weight cysteine-rich 10 |  |
| AT5G60070 | ankyrin repeat family protein |  |
| AT5G54010 | UDP-Glycosyltransferase superfamily protein |  |
| AT5G39870 | Protein of unknown function (DUF1216) |  |
| AT5G37060 | cation/H+ exchanger 24 |  |
| AT3G55190 | alpha/beta-Hydrolases superfamily protein |  |
| AT4G25950 | vacuolar ATP synthase G3 |  |
| AT3G23270 | Regulator of chromosome condensation (RCC1) family with FYVE zinc finger domain |  |
| AT1G19090 | receptor-like serine/threonine kinase 2 |  |
| AT1G15990 | cyclic nucleotide gated channel 7 |  |
| AT5G28970 | similar to Ulp1 protease family protein |  |
| AT1G04670 | unknown protein |  |
| AT2G29620 | unknown protein |  |
| AT4G14370 | Disease resistance protein (TIR-NBS-LRR class) family |  |
| AT5G41730 | Protein kinase family protein |  |
| AT5G41310 | P-loop nucleoside triphosphate hydrolases superfamily protein with CH (Calponin Homology) domain |  |
| AT1G18530 | EF hand calcium-binding protein family |  |
| AT3G22360 | alternative oxidase 1B |  |
| AT1G18410 | P-loop containing nucleoside triphosphate hydrolases superfamily protein |  |
| AT1G10680 | P-glycoprotein 10 |  |
| AT4G26770 | Phosphatidate cytidylyltransferase family protein |  |
| Locus id | TAIR Annotation |  |
| AT4G20500 | #N/A |  |
| AT2G03630 | unknown protein |  |
| AT5G18510 | Aminotransferase-like, plant mobile domain family protein |  |
| AT2G27120 | DNA polymerase epsilon catalytic subunit |  |
| AT2G33690 | Late embryogenesis abundant protein, group 6 |  |
| AT3G45810 | ferric reductase-like transmembrane component family protein |  |
| AT1G63190 | Cystatin/monellin superfamily protein |  |
| AT2G44550 | glycosyl hydrolase 9B10 |  |
| AT5G35380 | Protein kinase protein with adenine nucleotide alpha hydrolases-like domain |  |
| AT3G08560 | vacuolar H+-ATPase subunit E isoform 2 |  |
| AT2G01920 | ENTH/VHS/GAT family protein |  |
| AT2G45610 | alpha/beta-Hydrolases superfamily protein |  |
| AT3G26870 | Plant self-incompatibility protein S1 family |  |
| AT2G38920 | SPX (SYG1/Pho81/XPR1) domain-containing protein / zinc finger (C3HC4-type RING finger) protein-related |  |
| AT4G37840 | hexokinase-like 3 |  |
| AT3G47130 | F-box associated ubiquitination effector family protein |  |
| AT5G66740 | Protein of unknown function (DUF620) |  |
| AT1G22130 | AGAMOUS-like 104 |  |
| AT3G26880 | Plant self-incompatibility protein S1 family |  |
| AT5G09430 | alpha/beta-Hydrolases superfamily protein |  |
| AT5G63720 | kokopelli |  |
| AT4G13190 | Protein kinase superfamily protein |  |
| AT2G40990 | DHHC-type zinc finger family protein |  |
| Locus id | TAIR Annotation |  |
| AT3G07830 | Pectin lyase-like superfamily protein |  |
| AT4G39590 | Galactose oxidase/kelch repeat superfamily protein |  |
| AT2G18340 | late embryogenesis abundant domain-containing protein / LEA domain-containing protein |  |
| AT3G17760 | glutamate decarboxylase 5 |  |
| AT5G19360 | calcium-dependent protein kinase 34 |  |
| AT2G18180 | Sec14p-like phosphatidylinositol transfer family protein |  |
| AT2G43230 | Protein kinase superfamily protein |  |
| AT5G25340 | Ubiquitin-like superfamily protein |  |
| AT5G64510 | unknown protein |  |
| AT5G59370 | actin 4 |  |
| AT5G23270 | sugar transporter 11 |  |
| AT5G12000 | Protein kinase protein with adenine nucleotide alpha hydrolases-like domain |  |
| AT4G39670 | Glycolipid transfer protein (GLTP) family protein |  |
| AT4G26260 | myo-inositol oxygenase 4 |  |
| AT2G33460 | ROP-interactive CRIB motif-containing protein 1 |  |
| AT2G33270 | atypical CYS HIS rich thioredoxin 3 |  |
| AT3G28840 | Protein of unknown function (DUF1216) |  |
| AT3G04700 | Protein of unknown function (DUF1685) |  |
| AT3G20300 | Protein of unknown function (DUF3537) |  |
| AT3G19310 | PLC-like phosphodiesterases superfamily protein |  |
| AT3G17980 | Calcium-dependent lipid-binding (CaLB domain) family protein |  |
| AT1G68110 | ENTH/ANTH/VHS superfamily protein |  |
| AT1G79910 | Regulator of Vps4 activity in the MVB pathway protein |  |
| AT1G17540 | Protein kinase protein with adenine nucleotide alpha hydrolases-like domain |  |
| Locus id | TAIR Annotation |  |
| AT1G52580 | RHOMBOID-like protein 5 |  |
| AT1G68610 | PLANT CADMIUM RESISTANCE 11 |  |
| AT1G06540 | unknown protein |  |
| AT1G13970 | Protein of unknown function (DUF1336) |  |
| AT2G21480 | Malectin/receptor-like protein kinase family protein |  |
| AT2G17890 | calcium-dependent protein kinase 16 |  |
| AT1G67290 | glyoxal oxidase-related protein |  |
| AT2G13350 | Calcium-dependent lipid-binding (CaLB domain) family protein |  |
| AT2G15880 | Leucine-rich repeat (LRR) family protein |  |
| AT2G26850 | F-box family protein |  |
| AT4G14810 | unknown protein |  |
| AT1G25240 | ENTH/VHS/GAT family protein |  |
| AT5G19580 | glyoxal oxidase-related protein |  |
| AT5G58170 | SHV3-like 5 |  |
| AT5G55980 | serine-rich protein-related |  |
| AT5G15140 | Galactose mutarotase-like superfamily protein |  |
| AT5G02570 | Histone superfamily protein |  |
| AT3G61230 | GATA type zinc finger transcription factor family protein |  |
| AT3G56600 | Protein kinase superfamily protein |  |
| AT3G54800 | Pleckstrin homology (PH) and lipid-binding START domains-containing protein |  |
| AT4G39610 | Protein of unknown function, DUF617 |  |
| AT4G30140 | GDSL-like Lipase/Acylhydrolase superfamily protein |  |
| AT4G27790 | Calcium-binding EF hand family protein |  |
| AT4G13560 | Late embryogenesis abundant protein (LEA) family protein |  |
| Locus id | TAIR Annotation |  |
| AT4G10010 | Protein kinase superfamily protein |  |
| AT2G33420 | Protein of unknown function (DUF810) |  |
| AT3G06260 | galacturonosyltransferase-like 4 |  |
| AT3G13390 | SKU5 similar 11 |  |
| AT3G13400 | SKU5 similar 13 |  |
| AT3G20865 | arabinogalactan protein 40 |  |
| AT3G21180 | autoinhibited Ca(2+)-ATPase 9 |  |
| AT3G21570 | unknown protein |  |
| AT3G06830 | Plant invertase/pectin methylesterase inhibitor superfamily |  |
| AT3G04360 | Calcium-dependent lipid-binding (CaLB domain) family protein |  |
| AT3G02970 | EXORDIUM like 6 |  |
| AT3G01270 | Pectate lyase family protein |  |
| AT1G66210 | Subtilisin-like serine endopeptidase family protein |  |
| AT1G79860 | RHO guanyl-nucleotide exchange factor 12 |  |
| AT1G74000 | strictosidine synthase 3 |  |
| AT1G50610 | Leucine-rich repeat protein kinase family protein |  |
| AT1G10770 | Plant invertase/pectin methylesterase inhibitor superfamily protein |  |
| AT1G65970 | thioredoxin-dependent peroxidase 2 |  |
| AT1G61563 | ralf-like 8 |  |
| AT2G28180 | Cation/hydrogen exchanger family protein |  |
| AT5G04180 | alpha carbonic anhydrase 3 |  |
| AT4G36490 | SEC14-like 12 |  |
| AT5G57690 | diacylglycerol kinase 4 |  |
| AT5G50030 | Plant invertase/pectin methylesterase inhibitor superfamily protein |  |
| Locus id | TAIR Annotation |  |
| AT5G44300 | Dormancy/auxin associated family protein |  |
| AT5G39400 | Calcium/lipid-binding (CaLB) phosphatase |  |
| AT5G14380 | arabinogalactan protein 6 |  |
| AT5G13990 | exocyst subunit exo70 family protein C2 |  |
| AT3G43860 | glycosyl hydrolase 9A4 |  |
| AT4G35180 | LYS/HIS transporter 7 |  |
| AT4G28280 | LORELEI-LIKE-GPI ANCHORED PROTEIN 3 |  |
| AT4G28000 | P-loop containing nucleoside triphosphate hydrolases superfamily protein |  |
| AT4G25780 | CAP (Cysteine-rich secretory proteins, Antigen 5, and Pathogenesis-related 1 protein) superfamily protein |  |
| AT4G24640 | Plant invertase/pectin methylesterase inhibitor superfamily protein |  |
| AT4G04930 | fatty acid desaturase family protein |  |
| AT4G00350 | MATE efflux family protein |  |
| AT3G28750 | unknown protein |  |
| AT3G28790 | Protein of unknown function (DUF1216) |  |
| AT3G20580 | COBRA-like protein 10 precursor |  |
| AT2G21990 | Protein of unknown function, DUF617 |  |
| AT3G02480 | Late embryogenesis abundant protein (LEA) family protein |  |
| AT1G74010 | Calcium-dependent phosphotriesterase superfamily protein |  |
| AT1G02790 | polygalacturonase 4 |  |
| AT2G27180 | unknown protein |  |
| AT1G04540 | Calcium-dependent lipid-binding (CaLB domain) family protein |  |
| AT1G51240 | Plant self-incompatibility protein S1 family |  |
| AT2G16730 | glycosyl hydrolase family 35 protein |  |
| Locus id | TAIR Annotation |  |
| AT2G18470 | roline-rich extensin-like receptor kinase 4 |  |
| AT2G02140 | low-molecular-weight cysteine-rich 72 |  |
| AT2G26450 | Plant invertase/pectin methylesterase inhibitor superfamily |  |
| AT2G33100 | cellulose synthase-like D1 |  |
| AT5G20390 | Glycosyl hydrolase superfamily protein |  |
| AT5G26700 | RmlC-like cupins superfamily protein |  |
| AT5G64790 | O-Glycosyl hydrolases family 17 protein |  |
| AT5G45880 | Pollen Ole e 1 allergen and extensin family protein |  |
| AT3G62710 | Glycosyl hydrolase family protein |  |
| AT3G62170 | VANGUARD 1 homolog 2 |  |
| AT3G50310 | mitogen-activated protein kinase kinase kinase 20 |  |
| AT3G47440 | tonoplast intrinsic protein 5 |  |
| AT3G43120 | SAUR-like auxin-responsive protein family |  |
| AT4G39110 | Malectin/receptor-like protein kinase family protein |  |
| AT4G18395 | unknown protein |  |
| AT3G20220 | SAUR-like auxin-responsive protein family |  |
| AT3G07850 | Pectin lyase-like superfamily protein |  |
| AT3G05930 | germin-like protein 8 |  |
| AT1G29140 | Pollen Ole e 1 allergen and extensin family protein |  |
| AT2G22180 | hydroxyproline-rich glycoprotein family protein |  |
| AT2G36020 | HVA22-like protein J |  |
| AT1G61860 | Protein kinase superfamily protein |  |
| AT1G24520 | homolog of Brassica campestris pollen protein 1 |  |
| AT2G24370 | Protein kinase protein with adenine nucleotide alpha hydrolases-like domain |  |
| Locus id | TAIR Annotation |  |
| AT2G35210 | root and pollen arfgap |  |
| AT2G23900 | Pectin lyase-like superfamily protein |  |
| AT2G45800 | GATA type zinc finger transcription factor family protein |  |
| AT2G19000 | unknown protein |  |
| AT5G56640 | myo-inositol oxygenase 5 |  |
| AT5G50830 | unknown protein |  |
| AT5G48140 | Pectin lyase-like superfamily protein |  |
| AT5G47000 | Peroxidase superfamily protein |  |
| AT5G24240 | Phosphatidylinositol 3- and 4-kinase |  |
| AT3G52600 | cell wall invertase 2 |  |
| AT4G27580 | unknown protein |  |
| AT4G02250 | Plant invertase/pectin methylesterase inhibitor superfamily protein |  |
| AT3G19020 | Leucine-rich repeat (LRR) family protein |  |
| AT1G49290 | unknown protein |  |
| AT3G25165 | ralf-like 25 |  |
| AT3G25170 | ralf-like 26 |  |
| AT3G28980 | Protein of unknown function (DUF1216) |  |
| AT3G03430 | Calcium-binding EF-hand family protein |  |
| AT1G49490 | Leucine-rich repeat (LRR) family protein |  |
| AT1G23350 | Plant invertase/pectin methylesterase inhibitor superfamily protein |  |
| AT2G47050 | Plant invertase/pectin methylesterase inhibitor superfamily protein |  |
| AT2G26410 | IQ-domain 4 |  |
| AT2G26490 | Transducin/WD40 repeat-like superfamily protein |  |
| AT5G15110 | Pectate lyase family protein |  |
| Locus id | TAIR Annotation |  |
| AT5G27980 | Seed maturation protein |  |
| AT5G39420 | CDC2C |  |
| AT5G07430 | Pectin lyase-like superfamily protein |  |
| AT3G62180 | Plant invertase/pectin methylesterase inhibitor superfamily protein |  |
| AT3G62230 | F-box family protein |  |
| AT3G45280 | syntaxin of plants 72 |  |
| AT4G33970 | Leucine-rich repeat (LRR) family protein |  |
| AT2G24450 | FASCICLIN-like arabinogalactan protein 3 precursor |  |
| AT3G26110 | Anther-specific protein agp1-like |  |
| AT3G01240 | unknown protein |  |
| AT3G03800 | syntaxin of plants 131 |  |
| AT1G75870 | unknown protein |  |
| AT2G22340 | unknown protein |  |
| AT2G19770 | profilin 5 |  |
| AT2G47040 | Plant invertase/pectin methylesterase inhibitor superfamily |  |
| AT3G62640 | Protein of unknown function (DUF3511) |  |
| AT4G35010 | beta-galactosidase 11 |  |
| AT3G07820 | Pectin lyase-like superfamily protein |  |
| AT3G01700 | arabinogalactan protein 11 |  |
| AT2G13570 | nuclear factor Y, subunit B7 |  |
| AT1G55570 | SKU5 similar 12 |  |
| AT2G25600 | Shaker pollen inward K+ channel |  |
| AT1G58120 | BEST Arabidopsis thaliana protein match is: methyltransferases (TAIR:AT5G01710.1) |  |
| AT5G27870 | Plant invertase/pectin methylesterase inhibitor superfamily |  |
| Locus id | TAIR Annotation |  |
| AT5G39880 | unknown protein |  |
| AT1G69940 | Pectin lyase-like superfamily protein |  |
| AT1G55560 | SKU5 similar 14 |  |
| AT1G28270 | ralf-like 4 |  |
| AT4G11760 | low-molecular-weight cysteine-rich 17 |  |
| AT1G14420 | Pectate lyase family protein |  |
| AT1G11770 | FAD-binding Berberine family protein |  |
| AT5G14890 | NHL domain-containing protein |  |
| AT5G58050 | SHV3-like 4 |  |
| AT3G28810 | Protein of unknown function (DUF1216) |  |
| AT3G28830 | Protein of unknown function (DUF1216) |  |
| AT1G19500 | unknown protein |  |
| AT2G31500 | calcium-dependent protein kinase 24 |  |
| AT1G35490 | bZIP family transcription factor |  |
| AT5G20410 | monogalactosyldiacylglycerol synthase 2 |  |
| AT3G05610 | Plant invertase/pectin methylesterase inhibitor superfamily |  |
| AT2G20700 | LORELEI-LIKE-GPI ANCHORED PROTEIN 2 |  |
| AT2G02720 | Pectate lyase family protein |  |
| AT5G61720 | Protein of unknown function (DUF1216) |  |
| AT3G17060 | Pectin lyase-like superfamily protein |  |
| AT3G09530 | exocyst subunit exo70 family protein H3 |  |
| AT1G54070 | Dormancy/auxin associated family protein |  |
| AT3G16040 | Translation machinery associated TMA7 |  |
| AT1G01980 | FAD-binding Berberine family protein |  |
| Locus id | TAIR Annotation |  |
| AT2G05850 | serine carboxypeptidase-like 38 |  |
| AT3G27330 | zinc finger (C3HC4-type RING finger) family protein |  |
| AT2G44190 | Family of unknown function (DUF566) |  |
| AT5G45700 | Haloacid dehalogenase-like hydrolase (HAD) superfamily protein |  |
| AT5G05510 | Mad3/BUB1 homology region 1 |  |
| AT3G54930 | Protein phosphatase 2A regulatory B subunit family protein |  |
| AT3G51470 | Protein phosphatase 2C family protein |  |
| AT4G31020 | alpha/beta-Hydrolases superfamily protein |  |
| AT1G14970 | O-fucosyltransferase family protein |  |
| AT1G26840 | origin recognition complex protein 6 |  |
| AT5G21150 | Argonaute family protein |  |
| AT5G15070 | Phosphoglycerate mutase-like family protein |  |
| AT5G58760 | damaged DNA binding 2 |  |
| AT4G11920 | cell cycle switch protein 52 A2 |  |
| AT4G02900 | ERD (early-responsive to dehydration stress) family protein |  |
| AT3G01330 | DP-E2F-like protein 3 |  |
| AT1G68120 | basic pentacysteine 3 |  |
| AT1G52920 | G protein coupled receptor |  |
| AT1G35530 | DEAD/DEAH box RNA helicase family protein |  |
| AT1G75770 | unknown protein |  |
| AT2G28590 | Protein kinase superfamily protein |  |
| AT1G04160 | myosin XI B |  |
| AT1G65590 | beta-hexosaminidase 3 |  |
| AT1G03457 | RNA-binding (RRM/RBD/RNP motifs) family protein |  |
| Locus id | TAIR Annotation |  |
| AT2G36660 | poly(A) binding protein 7 |  |
| AT2G06040 | CONTAINS InterPro DOMAIN/s: Leucine-rich repeat, cysteine-containing subtype (InterPro:IPR006553) |  |
| AT2G43900 | Endonuclease/exonuclease/phosphatase family protein |  |
| AT2G23660 | LOB domain-containing protein 10 |  |
| AT2G34920 | RING/U-box superfamily protein |  |
| AT2G32670 | vesicle-associated membrane protein 725 |  |
| AT4G25610 | C2H2-like zinc finger protein |  |
| AT5G04110 | DNA GYRASE B3 |  |
| AT5G58690 | phosphatidylinositol-speciwc phospholipase C5 |  |
| AT5G49680 | Golgi-body localisation protein domain |  |
| AT5G38450 | cytochrome P450, family 735, subfamily A, polypeptide 1 |  |
| AT5G17780 | alpha/beta-Hydrolases superfamily protein |  |
| AT5G02430 | Transducin/WD40 repeat-like superfamily protein |  |
| AT3G58160 | P-loop containing nucleoside triphosphate hydrolases superfamily protein |  |
| AT3G51150 | ATP binding microtubule motor family protein |  |
| AT3G45870 | nodulin MtN21 /EamA-like transporter family protein |  |
| AT4G32500 | K+ transporter 5 |  |
| AT4G27980 | Domain of unknown function (DUF3444) |  |
| AT4G08590 | ORTHRUS-like |  |
| AT4G01470 | tonoplast intrinsic protein 1 |  |
| AT3G11020 | DRE/CRT-binding protein 2B |  |
| AT3G14070 | cation exchanger 9 |  |
| AT3G19230 | Leucine-rich repeat (LRR) family protein |  |
| Locus id | TAIR Annotation |  |
| AT3G04530 | phosphoenolpyruvate carboxylase kinase 2 |  |
| AT3G09480 | Histone superfamily protein |  |
| AT1G72460 | Leucine-rich repeat protein kinase family protein |  |
| AT1G07410 | RAB GTPase homolog A2B |  |
| AT1G59860 | HSP20-like chaperones superfamily protein |  |
| AT1G03200 | unknown protein |  |
| AT1G08730 | Myosin family protein with Dil domain |  |
| AT1G24330 | ARM repeat superfamily protein |  |
| AT2G22600 | RNA-binding KH domain-containing protein |  |
| AT2G18600 | Ubiquitin-conjugating enzyme family protein |  |
| AT2G33240 | myosin XI D |  |
| AT5G57830 | Protein of unknown function, DUF593 |  |
| AT5G55950 | Nucleotide/sugar transporter family protein |  |
| AT5G24560 | phloem protein 2-B12 |  |
| AT5G02720 | unknown protein |  |
| AT5G01180 | peptide transporter 5 |  |
| AT3G61760 | DYNAMIN-like 1B |  |
| AT3G55500 | expansin A16 |  |
| AT4G35070 | SBP (S-ribonuclease binding protein) family protein |  |
| AT4G27370 | P-loop containing nucleoside triphosphate hydrolases superfamily protein |  |
| AT1G17830 | Protein of unknown function (DUF789) |  |
| AT3G30380 | alpha/beta-Hydrolases superfamily protein |  |
| AT3G23870 | Protein of unknown function (DUF803) |  |
| AT3G27290 | RNI-like superfamily protein |  |
| Locus id | TAIR Annotation |  |
| AT3G24840 | Sec14p-like phosphatidylinositol transfer family protein |  |
| AT3G17630 | cation/H+ exchanger 19 |  |
| AT1G49160 | Protein kinase superfamily protein |  |
| AT2G31540 | GDSL-like Lipase/Acylhydrolase superfamily protein |  |
| AT2G22560 | Kinase interacting (KIP1-like) family protein |  |
| AT1G78980 | STRUBBELIG-receptor family 5 |  |
| AT2G06005 | FRIGIDA interacting protein 1 |  |
| AT2G29500 | HSP20-like chaperones superfamily protein |  |
| AT2G38520 | copia-like retrotransposon family |  |
| AT2G32930 | zinc finger nuclease 2 |  |
| AT4G15120 | VQ motif-containing protein |  |
| AT5G57190 | phosphatidylserine decarboxylase 2 |  |
| AT5G56160 | Sec14p-like phosphatidylinositol transfer family protein |  |
| AT5G42010 | Transducin/WD40 repeat-like superfamily protein |  |
| AT5G37230 | RING/U-box superfamily protein |  |
| AT5G14670 | ADP-ribosylation factor A1B |  |
| AT4G35380 | SEC7-like guanine nucleotide exchange family protein |  |
| AT4G26830 | O-Glycosyl hydrolases family 17 protein |  |
| AT4G18050 | P-glycoprotein 9 |  |
| AT1G19940 | glycosyl hydrolase 9B5 |  |
| AT3G13965 | hypothetical protein |  |
| AT1G75110 | Nucleotide-diphospho-sugar transferase family protein |  |
| AT1G79900 | Mitochondrial substrate carrier family protein |  |
| AT1G18520 | tetraspanin11 |  |
| Locus id | TAIR Annotation |  |
| AT1G16290 | FUNCTIONS IN: molecular_function unknown |  |
| AT1G04090 | Plant protein of unknown function (DUF946) |  |
| AT1G03620 | ELMO/CED-12 family protein |  |
| AT2G41210 | phosphatidylinositol- 4-phosphate 5-kinase 5 |  |
| AT5G35410 | Protein kinase superfamily protein |  |
| AT5G56780 | effector of transcription2 |  |
| AT5G24655 | response to low sulfur 4 |  |
| AT5G13700 | polyamine oxidase 1 |  |
| AT5G09800 | ARM repeat superfamily protein |  |
| AT5G08240 | unknown protein |  |
| AT3G56960 | phosphatidyl inositol monophosphate 5 kinase 4 |  |
| AT3G47780 | ABC2 homolog 6 |  |
| AT3G44070 | Glycosyl hydrolase family 35 protein |  |
| AT4G37990 | elicitor-activated gene 3-2 |  |
| AT4G25150 | HAD superfamily, subfamily IIIB acid phosphatase |  |
| AT4G25040 | Uncharacterised protein family (UPF0497) |  |
| AT4G07960 | Cellulose-synthase-like C12 |  |
| AT4G02660 | Beige/BEACH domain |  |
| AT1G50990 | Protein kinase protein with tetratricopeptide repeat domain |  |
| AT3G09550 | Ankyrin repeat family protein |  |
| AT3G16380 | poly(A) binding protein 6 |  |
| AT1G64300 | Protein kinase family protein |  |
| AT1G01340 | cyclic nucleotide gated channel 10 |  |
| AT1G18280 | Bifunctional inhibitor/lipid-transfer protein/seed storage 2S albumin superfamily protein |  |
| Locus id | TAIR Annotation |  |
| AT1G08170 | Histone superfamily protein |  |
| AT1G68630 | PLAC8 family protein |  |
| AT1G73010 | phosphate starvation-induced gene 2 |  |
| AT2G31830 | endonuclease/exonuclease/phosphatase family protein |  |
| AT1G54560 | Myosin family protein with Dil domain |  |
| AT1G09600 | Protein kinase superfamily protein |  |
| AT1G70170 | matrix metalloproteinase |  |
| AT1G05020 | ENTH/ANTH/VHS superfamily protein |  |
| AT2G37980 | O-fucosyltransferase family protein |  |
| AT2G22860 | phytosulfokine 2 precursor |  |
| AT2G19400 | AGC (cAMP-dependent, cGMP-dependent and protein kinase C) kinase family protein |  |
| AT2G19330 | plant intracellular ras group-related LRR 6 |  |
| AT1G67810 | sulfur E2 |  |
| AT4G17690 | Peroxidase superfamily protein |  |
| AT4G17483 | alpha/beta-Hydrolases superfamily protein |  |
| AT1G26320 | Zinc-binding dehydrogenase family protein |  |
| AT5G21050 | LOCATED IN: chloroplast |  |
| AT5G15100 | Auxin efflux carrier family protein |  |
| AT5G26100 | unknown protein |  |
| AT5G67520 | adenosine-5'-phosphosulfate (APS) kinase 4 |  |
| AT5G65090 | DNAse I-like superfamily protein |  |
| AT5G61620 | myb-like transcription factor family protein |  |
| AT5G61730 | ABC2 homolog 11 |  |
| AT5G60250 | zinc finger (C3HC4-type RING finger) family protein |  |
| Locus id | TAIR Annotation |  |
| AT5G57320 | villin, putative |  |
| AT5G37810 | NOD26-like intrinsic protein 4 |  |
| AT5G01610 | Protein of unknown function, DUF538 |  |
| AT3G13782 | nucleosome assembly protein1 |  |
| AT3G13900 | ATPase E1-E2 type family protein / haloacid dehalogenase-like hydrolase family protein |  |
| AT3G01015 | TPX2 (targeting protein for Xklp2) protein family |  |
| AT1G64110 | P-loop containing nucleoside triphosphate hydrolases superfamily protein |  |
| AT1G03920 | Protein kinase family protein |  |
| AT2G25460 | CONTAINS InterPro DOMAIN/s: C2 calcium-dependent membrane targeting (InterPro:IPR000008) |  |
| AT3G54700 | phosphate transporter 1 |  |
| AT5G27000 | kinesin 4 |  |
| AT5G61980 | ARF-GAP domain 1 |  |
| AT3G46230 | heat shock protein 17.4 |  |
| AT3G20200 | Protein kinase protein with adenine nucleotide alpha hydrolases-like domain |  |
| AT3G02850 | STELAR K+ outward rectifier |  |
| AT3G10470 | C2H2-type zinc finger family protein |  |
| AT1G71770 | poly(A)-binding protein 5 |  |
| AT2G13680 | callose synthase 5 |  |
| AT5G61700 | ABC2 homolog 16 |  |
| AT5G01690 | cation/H+ exchanger 27 |  |
| AT5G01700 | Protein phosphatase 2C family protein |  |
| AT3G60330 | H(+)-ATPase 7 |  |
| AT4G38230 | calcium-dependent protein kinase 26 |  |
| Locus id | TAIR Annotation |  |
| AT4G26470 | Calcium-binding EF-hand family protein |  |
| AT4G09950 | P-loop containing nucleoside triphosphate hydrolases superfamily protein |  |
| AT2G20430 | ROP-interactive CRIB motif-containing protein 6 |  |
| AT3G07960 | Phosphatidylinositol-4-phosphate 5-kinase family protein |  |
| AT1G17710 | Pyridoxal phosphate phosphatase-related protein |  |
| AT1G67640 | Transmembrane amino acid transporter family protein |  |
| AT1G53540 | HSP20-like chaperones superfamily protein |  |
| AT1G04700 | PB1 domain-containing protein tyrosine kinase |  |
| AT2G29940 | pleiotropic drug resistance 3 |  |
| AT2G22950 | Cation transporter/ E1-E2 ATPase family protein |  |
| AT5G62850 | Nodulin MtN3 family protein |  |
| AT5G44610 | microtubule-associated protein 18 |  |
| AT5G09720 | Magnesium transporter CorA-like family protein |  |
| AT3G54580 | Proline-rich extensin-like family protein |  |
| AT4G32630 | ArfGap/RecO-like zinc finger domain-containing protein |  |
| AT3G03080 | Zinc-binding dehydrogenase family protein |  |
| AT1G71380 | cellulase 3 |  |
| AT2G37010 | non-intrinsic ABC protein 12 |  |
| AT2G24320 | alpha/beta-Hydrolases superfamily protein |  |
| AT5G47470 | Nodulin MtN21 /EamA-like transporter family protein |  |
| AT3G28630 | Protein of unknown function (DUF569) |  |
| AT3G24715 | Protein kinase superfamily protein with octicosapeptide/Phox/Bem1p domain |  |
| AT3G05140 | ROP binding protein kinases 2 |  |
| AT1G69050 | unknown protein |  |
| Locus id | TAIR Annotation |  |
| AT1G04880 | HMG (high mobility group) box protein with ARID/BRIGHT DNA-binding domain |  |
| AT1G22460 | O-fucosyltransferase family protein |  |
| AT1G43630 | Protein of unknown function (DUF793) |  |
| AT1G16360 | LEM3 (ligand-effect modulator 3) family protein / CDC50 family protein |  |
| AT1G09170 | P-loop nucleoside triphosphate hydrolases superfamily protein with CH (Calponin Homology) domain |  |
| AT1G04600 | myosin XI A |  |
| AT2G45750 | S-adenosyl-L-methionine-dependent methyltransferases superfamily protein |  |
| AT5G28010 | Polyketide cyclase/dehydrase and lipid transport superfamily protein |  |
| AT5G25880 | NADP-malic enzyme 3 |  |
| AT5G60010 | ferric reductase-like transmembrane component family protein |  |
| AT5G10260 | RAB GTPase homolog H1E |  |
| AT4G38190 | cellulose synthase like D4 |  |
| AT4G29550 | Protein of unknown function (DUF626) |  |
| AT4G26730 | S-adenosyl-L-methionine-dependent methyltransferases superfamily protein |  |
| AT3G28210 | zinc finger (AN1-like) family protein |  |
| AT1G08135 | cation/H+ exchanger 6B |  |
| AT2G36190 | cell wall invertase 4 |  |
| AT1G03445 | Serine/threonine protein phosphatase family protein |  |
| AT4G15200 | formin 3 |  |
| AT4G14780 | Protein kinase superfamily protein |  |
| AT5G28470 | Major facilitator superfamily protein |  |
| AT1G57550 | Low temperature and salt responsive protein family |  |
| AT5G60260 | unknown protein |  |
| Locus id | TAIR Annotation |  |
| AT1G69430 | unknown protein |  |
| AT3G13660 | Disease resistance-responsive (dirigent-like protein) family protein |  |
| AT1G29630 | 5'-3' exonuclease family protein |  |
| AT5G14870 | cyclic nucleotide-gated channel 18 |  |
| AT3G01620 | beta-1,4-N-acetylglucosaminyltransferase family protein |  |
| AT4G15980 | Plant invertase/pectin methylesterase inhibitor superfamily |  |
| AT5G64990 | RAB GTPase homolog H1A |  |
| AT5G57240 | OSBP(oxysterol binding protein)-related protein 4C |  |
| AT3G60010 | SKP1-like 13 |  |
| AT3G60100 | citrate synthase 5 |  |
| AT5G20340 | beta-1,3-glucanase 5 |  |
| AT5G04390 | C2H2-type zinc finger family protein |  |
| AT5G60740 | ABC transporter family protein |  |
| AT3G08810 | Galactose oxidase/kelch repeat superfamily protein |  |
| AT3G05950 | RmlC-like cupins superfamily protein |  |
| AT2G33670 | Seven transmembrane MLO family protein |  |
| AT5G55490 | gamete expressed protein 1 |  |
| AT5G53250 | arabinogalactan protein 22 |  |
| AT3G44400 | Disease resistance protein (TIR-NBS-LRR class) family |  |
| AT4G35280 | C2H2-like zinc finger protein |  |
| AT4G25940 | ENTH/ANTH/VHS superfamily protein |  |
| AT2G33350 | CCT motif family protein |  |
| AT3G20710 | F-box family protein |  |
| AT1G79450 | ALA-interacting subunit 5 |  |
| Locus id | TAIR Annotation | |
| AT1G51260 | lysophosphatidyl acyltransferase 3 | |
| AT2G37070 | unknown protein | |
| AT2G03060 | AGAMOUS-like 30 | |
| AT2G19010 | GDSL-like Lipase/Acylhydrolase superfamily protein | |
| AT5G16100 | unknown protein | |
| AT5G62310 | AGC (cAMP-dependent, cGMP-dependent and protein kinase C) kinase family | |
| AT5G48780 | disease resistance protein (TIR-NBS class) | |
| AT4G00240 | phospholipase D beta 2 | |
| AT3G05960 | sugar transporter 6 | |
| AT1G14640 | SWAP (Suppressor-of-White-APricot)/surp domain-containing protein | |
| AT1G62450 | Immunoglobulin E-set superfamily protein | |
| AT2G28355 | low-molecular-weight cysteine-rich 5 | |
| AT2G38910 | calcium-dependent protein kinase 20 | |
| AT2G30690 | Protein of unknown function, DUF593 | |
| AT2G47550 | Plant invertase/pectin methylesterase inhibitor superfamily | |
| AT5G25400 | Nucleotide-sugar transporter family protein | |
| AT3G51070 | S-adenosyl-L-methionine-dependent methyltransferases superfamily protein | |
| AT3G46750 | unknown protein | |
| AT4G13230 | Late embryogenesis abundant protein (LEA) family protein | |
| AT4G08670 | Bifunctional inhibitor/lipid-transfer protein/seed storage 2S albumin superfamily | |
| AT3G16320 | Tetratricopeptide repeat (TPR)-like superfamily protein | |
| AT1G33770 | Protein kinase superfamily protein | |
| AT2G03840 | tetraspanin13 | |
| AT2G15340 | glycine-rich protein | |
| Locus id | TAIR Annotation |  |
| AT1G04500 | CCT motif family protein |  |
| AT1G60040 | AGAMOUS-like 49 |  |
| AT2G21510 | DNAJ heat shock N-terminal domain-containing protein |  |
| AT2G37670 | Transducin/WD40 repeat-like superfamily protein |  |
| AT5G20690 | Leucine-rich repeat protein kinase family protein |  |
| AT5G20310 | Adenine nucleotide alpha hydrolases-like superfamily protein |  |
| AT5G35390 | Leucine-rich repeat protein kinase family protein |  |
| AT5G66020 | Phosphoinositide phosphatase family protein |  |
| AT5G14180 | Myzus persicae-induced lipase 1 |  |
| AT5G02390 | Protein of unknown function (DUF3741) |  |
| AT3G60580 | C2H2-like zinc finger protein |  |
| AT3G55940 | Phosphoinositide-specific phospholipase C family protein |  |
| AT4G02160 | unknown protein |  |
| AT3G12060 | Plant protein of unknown function (DUF828) |  |
| AT3G29070 | emp24/gp25L/p24 family/GOLD family protein |  |
| AT3G27440 | uridine kinase-like 5 |  |
| AT3G10660 | calmodulin-domain protein kinase cdpk isoform 2 |  |
| AT1G19640 | jasmonic acid carboxyl methyltransferase |  |
| AT1G77980 | AGAMOUS-like 66 |  |
| AT2G05160 | CCCH-type zinc fingerfamily protein with RNA-binding domain |  |
| AT2G28640 | exocyst subunit exo70 family protein H5 |  |
| AT1G61700 | RNA polymerases N / 8 kDa subunit |  |
| AT2G46040 | ARID/BRIGHT DNA-binding domain |  |
| AT2G19050 | GDSL-like Lipase/Acylhydrolase superfamily protein |  |
| Locus id | TAIR Annotation |  |
| AT5G51030 | NAD(P)-binding Rossmann-fold superfamily protein |  |
| AT4G18990 | xyloglucan endotransglucosylase/hydrolase 29 |  |
| AT4G13240 | RHO guanyl-nucleotide exchange factor 9 |  |
| AT4G13450 | Adenine nucleotide alpha hydrolases-like superfamily protein |  |
| AT1G79400 | cation/H+ exchanger 2 |  |
| AT1G65240 | Eukaryotic aspartyl protease family protein |  |
| AT2G41970 | Protein kinase superfamily protein |  |
| AT1G44120 | Armadillo/beta-catenin-like repeat |  |
| AT5G27610 | DIRP |  |
| AT5G67080 | mitogen-activated protein kinase kinase kinase 19 |  |
| AT5G65530 | Protein kinase superfamily protein |  |
| AT5G42170 | SGNH hydrolase-type esterase superfamily protein |  |
| AT5G03000 | Galactose oxidase/kelch repeat superfamily protein |  |
| AT1G22110 | structural constituent of ribosome |  |
| AT3G11180 | 2-oxoglutarate (2OG) and Fe(II)-dependent oxygenase superfamily protein |  |
| AT3G04620 | Alba DNA/RNA-binding protein |  |
| AT1G73066 | Leucine-rich repeat family protein |  |
| AT1G79250 | AGC kinase 1.7 |  |
| AT1G24110 | Peroxidase superfamily protein |  |
| AT5G20810 | SAUR-like auxin-responsive protein family |  |
| AT5G25530 | DNAJ heat shock family protein |  |
| AT3G28150 | TRICHOME BIREFRINGENCE-LIKE 22 |  |
| AT3G27410 | unknown protein |  |
| AT1G75050 | Pathogenesis-related thaumatin superfamily protein |  |
| Locus id | TAIR Annotation |  |
| AT1G78940 | Protein kinase protein with adenine nucleotide alpha hydrolases-like domain |  |
| AT2G29410 | metal tolerance protein B1 |  |
| AT4G16745 | Exostosin family protein |  |
| AT5G25430 | HCO3- transporter family |  |
| AT4G34940 | armadillo repeat only 1 |  |
| AT4G34440 | Protein kinase superfamily protein |  |
| AT4G30770 | Putative membrane lipoprotein |  |
| AT4G27110 | COBRA-like protein 11 precursor |  |
| AT4G20160 | LOCATED IN: chloroplast |  |
| AT2G30290 | VACUOLAR SORTING RECEPTOR 2 |  |
| AT3G28230 | FUNCTIONS IN: molecular_function unknown |  |
| AT3G02440 | TRICHOME BIREFRINGENCE-LIKE 20 |  |
| AT1G11040 | HSP40/DnaJ peptide-binding protein |  |
| AT1G11250 | syntaxin of plants 125 |  |
| AT1G44160 | HSP40/DnaJ peptide-binding protein |  |
| AT1G45545 | Plant protein of unknown function (DUF827) |  |
| AT5G45840 | Leucine-rich repeat protein kinase family protein |  |
| AT5G12030 | heat shock protein 17.6A |  |
| AT3G57140 | sugar-dependent 1-like |  |
| AT3G55180 | alpha/beta-Hydrolases superfamily protein |  |
| AT3G47660 | Regulator of chromosome condensation (RCC1) family protein |  |
| AT4G18790 | NRAMP metal ion transporter family protein |  |
| AT3G12510 | MADS-box family protein |  |
| AT3G18810 | Protein kinase superfamily protein |  |
| Locus id | TAIR Annotation |  |
| AT1G70430 | Protein kinase superfamily protein |  |
| AT5G61350 | Protein kinase superfamily protein |  |
| AT5G57200 | ENTH/ANTH/VHS superfamily protein |  |
| AT5G09550 | GDP dissociation inhibitor family protein / Rab GTPase activator family protein |  |
| AT3G60570 | expansin B5 |  |
| AT3G29060 | EXS (ERD1/XPR1/SYG1) family protein |  |
| AT3G19610 | Plant protein of unknown function (DUF936) |  |
| AT3G05820 | invertase H |  |
| AT1G73860 | P-loop containing nucleoside triphosphate hydrolases superfamily protein |  |
| AT1G07330 | unknown protein |  |
| AT3G11740 | Protein of unknown function (DUF567) |  |
| AT2G29040 | Exostosin family protein |  |
| AT3G48450 | RPM1-interacting protein 4 (RIN4) family protein |  |
| AT4G39180 | Sec14p-like phosphatidylinositol transfer family protein |  |
| AT4G29340 | profilin 4 |  |
| AT1G75160 | Protein of unknown function (DUF620) |  |
| AT3G09590 | CAP (Cysteine-rich secretory proteins, Antigen 5, and Pathogenesis-related 1 protein) superfamily protein |  |
| AT2G17660 | RPM1-interacting protein 4 (RIN4) family protein |  |
| AT2G25630 | beta glucosidase 14 |  |
| AT5G65160 | tetratricopeptide repeat (TPR)-containing protein |  |
| AT4G39753 | Galactose oxidase/kelch repeat superfamily protein |  |
| AT4G26330 | Subtilisin-like serine endopeptidase family protein |  |
| AT4G24580 | Rho GTPase activation protein (RhoGAP) with PH domain |  |
| Locus id | TAIR Annotation |  |
| AT3G13420 | unknown protein |  |
| AT3G10460 | Plant self-incompatibility protein S1 family |  |
| AT1G12070 | Immunoglobulin E-set superfamily protein |  |
| AT5G55020 | myb domain protein 120 |  |
| AT4G33230 | Plant invertase/pectin methylesterase inhibitor superfamily |  |
| AT4G23960 | F-box family protein |  |
| AT1G74490 | Protein kinase superfamily protein |  |
| AT1G54280 | ATPase E1-E2 type family protein / haloacid dehalogenase-like hydrolase family protein |  |
| AT2G32460 | myb domain protein 101 |  |
| AT3G52460 | hydroxyproline-rich glycoprotein family protein |  |
| AT1G68750 | phosphoenolpyruvate carboxylase 4 |  |
| AT5G24880 | BEST Arabidopsis thaliana protein match is: calmodulin-binding protein-related (TAIR:AT5G10660.1) |  |
| AT5G42340 | Plant U-Box 15 |  |
| AT2G39820 | Translation initiation factor IF6 |  |
| AT1G31750 | proline-rich family protein |  |
| AT2G30640 | Encodes a member of a domesticated transposable element gene family MUSTANG |  |

**Table S8.** Assignment of rice orthologs to *Arabidopsis* late pollen-preferred genes. Locus numbers are shown in by red.

| Locus number | Orthologue |  |  |  |  |  |  |  |  |
| --- | --- | --- | --- | --- | --- | --- | --- | --- | --- |
| AT1G51410 | LOC_Os01g34480 |  |  |  |  |  |  |  |  |
| AT1G03050 | LOC_Os05g35160 |  |  |  |  |  |  |  |  |
| AT2G04220 | LOC_Os01g68140 | LOC_Os02g10530 |  |  |  |  |  |  |  |
| AT1G61290 | LOC_Os06g39050 |  |  |  |  |  |  |  |  |
| AT2G07560 | LOC_Os02g55400 | LOC_Os03g01120 | LOC_Os03g08560 | LOC_Os03g48310 | LOC_Os04g56160 | LOC_Os05g25550 | LOC_Os06g08310 | LOC_Os07g09340 | LOC_Os12g44150 |
| AT2G07040 | LOC_Os08g40990 |  |  |  |  |  |  |  |  |
| AT2G46860 | LOC_Os04g59040 | LOC_Os05g36260 |  |  |  |  |  |  |  |
| AT2G38500 | LOC_Os01g60800 |  |  |  |  |  |  |  |  |
| AT4G25590 | LOC_Os02g44470 | LOC_Os04g46910 |  |  |  |  |  |  |  |
| AT5G48270 | LOC_Os01g68140 | LOC_Os02g10530 |  |  |  |  |  |  |  |
| AT5G18910 | LOC_Os06g45350 |  |  |  |  |  |  |  |  |
| AT5G12180 | LOC_Os01g59360 | LOC_Os05g41270 |  |  |  |  |  |  |  |
| AT5G10090 | LOC_Os02g51730 |  |  |  |  |  |  |  |  |
| AT3G59830 | LOC_Os02g39560 |  |  |  |  |  |  |  |  |
| AT3G48010 | LOC_Os12g06570 |  |  |  |  |  |  |  |  |
| AT1G16760 | LOC_Os02g54590 | LOC_Os06g09230 |  |  |  |  |  |  |  |
| AT2G33320 | LOC_Os02g57000 |  |  |  |  |  |  |  |  |
| AT3G20190 | LOC_Os08g40990 |  |  |  |  |  |  |  |  |
| AT1G24620 | LOC_Os01g72100 |  |  |  |  |  |  |  |  |
| AT3G06560 | LOC_Os03g19920 | LOC_Os07g48890 |  |  |  |  |  |  |  |
| AT3G07490 | LOC_Os12g12730 |  |  |  |  |  |  |  |  |
| AT3G01020 | LOC_Os01g47340 |  |  |  |  |  |  |  |  |
| Locus number | Orthologue |  |  |  |  |  |  |  |  |
| AT1G13890 | LOC_Os02g24080 | LOC_Os02g32740 |  |  |  |  |  |  |  |
| AT1G52240 | LOC_Os02g17240 |  |  |  |  |  |  |  |  |
| AT1G80660 | LOC_Os02g55400 | LOC_Os03g01120 | LOC_Os03g08560 | LOC_Os03g48310 | LOC_Os04g56160 | LOC_Os05g25550 | LOC_Os06g08310 | LOC_Os07g09340 | LOC_Os12g44150 |
| AT2G48150 | LOC_Os03g24380 |  |  |  |  |  |  |  |  |
| AT2G13620 | LOC_Os01g60140 | LOC_Os05g40650 |  |  |  |  |  |  |  |
| AT1G08860 | LOC_Os05g30970 |  |  |  |  |  |  |  |  |
| AT1G23540 | LOC_Os06g29080 |  |  |  |  |  |  |  |  |
| AT2G18080 | LOC_Os10g36760 | LOC_Os10g36780 |  |  |  |  |  |  |  |
| AT5G13150 | LOC_Os11g06700 | LOC_Os12g06840 |  |  |  |  |  |  |  |
| AT5G17480 | LOC_Os08g44660 |  |  |  |  |  |  |  |  |
| AT5G26150 | LOC_Os01g39970 | LOC_Os02g12660 |  |  |  |  |  |  |  |
| AT5G05070 | LOC_Os01g17160 |  |  |  |  |  |  |  |  |
| AT3G60780 | LOC_Os02g25780 |  |  |  |  |  |  |  |  |
| AT3G42880 | LOC_Os06g09860 |  |  |  |  |  |  |  |  |
| AT4G40020 | LOC_Os08g37500 |  |  |  |  |  |  |  |  |
| AT4G33860 | LOC_Os03g10478 |  |  |  |  |  |  |  |  |
| AT4G33390 | LOC_Os07g42700 |  |  |  |  |  |  |  |  |
| AT4G31230 | LOC_Os02g54590 | LOC_Os06g09230 |  |  |  |  |  |  |  |
| AT4G04080 | LOC_Os01g47340 |  |  |  |  |  |  |  |  |
| AT3G19690 | LOC_Os01g28450 | LOC_Os01g28500 | LOC_Os07g03690 | LOC_Os07g03710 | LOC_Os07g03730 | LOC_Os07g03740 | LOC_Os10g11500 |  |  |
| AT3G28490 | LOC_Os03g58890 | LOC_Os10g27340 |  |  |  |  |  |  |  |
| AT3G24620 | LOC_Os05g48640 |  |  |  |  |  |  |  |  |
| AT1G76640 | LOC_Os01g72530 | LOC_Os01g72550 |  |  |  |  |  |  |  |
| AT1G68090 | LOC_Os09g23160 |  |  |  |  |  |  |  |  |
| AT1G06750 | LOC_Os05g43300 |  |  |  |  |  |  |  |  |
| AT1G20080 | LOC_Os02g25060 | LOC_Os09g36770 |  |  |  |  |  |  |  |
| Locus number | Orthologue |  |  |  |  |  |  |  |  |
| AT1G23210 | LOC_Os02g50040 | LOC_Os06g14540 |  |  |  |  |  |  |  |
| AT2G42380 | LOC_Os01g11350 | LOC_Os01g55150 |  |  |  |  |  |  |  |
| AT5G19610 | LOC_Os04g02690 |  |  |  |  |  |  |  |  |
| AT5G20330 | LOC_Os01g58730 |  |  |  |  |  |  |  |  |
| AT5G44640 | LOC_Os04g39880 |  |  |  |  |  |  |  |  |
| AT5G52340 | LOC_Os11g05880 |  |  |  |  |  |  |  |  |
| AT1G58210 | LOC_Os01g61910 | LOC_Os05g39000 |  |  |  |  |  |  |  |
| AT3G52080 | LOC_Os12g44300 |  |  |  |  |  |  |  |  |
| AT3G07840 | LOC_Os06g35320 | LOC_Os06g35370 |  |  |  |  |  |  |  |
| AT3G23270 | LOC_Os01g50470 | LOC_Os05g46740 |  |  |  |  |  |  |  |
| AT4G14370 | LOC_Os01g71106 |  |  |  |  |  |  |  |  |
| AT5G41730 | LOC_Os01g43350 | LOC_Os05g50830 |  |  |  |  |  |  |  |
| AT5G41310 | LOC_Os01g54080 | LOC_Os05g44560 |  |  |  |  |  |  |  |
| AT1G18530 | LOC_Os05g50180 |  |  |  |  |  |  |  |  |
| AT1G10680 | LOC_Os02g46680 | LOC_Os04g38570 | LOC_Os08g45030 |  |  |  |  |  |  |
| AT4G26770 | LOC_Os10g17990 |  |  |  |  |  |  |  |  |
| AT2G03630 | LOC_Os07g20270 |  |  |  |  |  |  |  |  |
| AT5G18510 | LOC_Os03g36830 | LOC_Os03g60170 |  |  |  |  |  |  |  |
| AT2G27120 | LOC_Os02g30800 |  |  |  |  |  |  |  |  |
| AT3G45810 | LOC_Os01g61880 | LOC_Os05g38980 |  |  |  |  |  |  |  |
| AT3G08560 | LOC_Os05g40230 |  |  |  |  |  |  |  |  |
| AT2G38920 | LOC_Os03g44810 |  |  |  |  |  |  |  |  |
| AT5G66740 | LOC_Os01g55795 |  |  |  |  |  |  |  |  |
| AT1G22130 | LOC_Os06g11970 |  |  |  |  |  |  |  |  |
| AT5G09430 | LOC_Os03g27110 | LOC_Os10g22960 |  |  |  |  |  |  |  |
| AT3G07830 | LOC_Os06g35320 | LOC_Os06g35370 |  |  |  |  |  |  |  |
| Locus number | Orthologue |  |  |  |  |  |  |  |  |
| AT3G17760 | LOC_Os04g37460 |  |  |  |  |  |  |  |  |
| AT5G19360 | LOC_Os01g59360 | LOC_Os05g41270 |  |  |  |  |  |  |  |
| AT2G18180 | LOC_Os02g04030 | LOC_Os02g24430 | LOC_Os10g03400 |  |  |  |  |  |  |
| AT2G43230 | LOC_Os01g67340 | LOC_Os03g62700 | LOC_Os09g33860 |  |  |  |  |  |  |
| AT5G25340 | LOC_Os02g09530 |  |  |  |  |  |  |  |  |
| AT5G64510 | LOC_Os01g56180 |  |  |  |  |  |  |  |  |
| AT5G12000 | LOC_Os01g39970 | LOC_Os02g12660 |  |  |  |  |  |  |  |
| AT4G39670 | LOC_Os03g50280 |  |  |  |  |  |  |  |  |
| AT4G26260 | LOC_Os06g36560 |  |  |  |  |  |  |  |  |
| AT2G33460 | LOC_Os02g56580 |  |  |  |  |  |  |  |  |
| AT3G04700 | LOC_Os04g21340 |  |  |  |  |  |  |  |  |
| AT3G20300 | LOC_Os04g37520 | LOC_Os06g42850 |  |  |  |  |  |  |  |
| AT3G19310 | LOC_Os04g35100 |  |  |  |  |  |  |  |  |
| AT3G17980 | LOC_Os02g22130 |  |  |  |  |  |  |  |  |
| AT1G68110 | LOC_Os08g36410 |  |  |  |  |  |  |  |  |
| AT1G79910 | LOC_Os02g06430 |  |  |  |  |  |  |  |  |
| AT1G68610 | LOC_Os02g36940 |  |  |  |  |  |  |  |  |
| AT1G13970 | LOC_Os12g38850 |  |  |  |  |  |  |  |  |
| AT2G21480 | LOC_Os06g03610 |  |  |  |  |  |  |  |  |
| AT2G17890 | LOC_Os02g03410 | LOC_Os07g22710 |  |  |  |  |  |  |  |
| AT1G67290 | LOC_Os01g48540 |  |  |  |  |  |  |  |  |
| AT2G15880 | LOC_Os01g25460 | LOC_Os12g35710 |  |  |  |  |  |  |  |
| AT2G26850 | LOC_Os01g17390 |  |  |  |  |  |  |  |  |
| AT1G25240 | LOC_Os08g36410 |  |  |  |  |  |  |  |  |
| AT5G19580 | LOC_Os01g48540 |  |  |  |  |  |  |  |  |
| AT5G58170 | LOC_Os02g09450 |  |  |  |  |  |  |  |  |
| Locus number | Orthologue |  |  |  |  |  |  |  |  |
| AT3G61230 | LOC_Os02g42820 |  |  |  |  |  |  |  |  |
| AT3G56600 | LOC_Os01g16470 |  |  |  |  |  |  |  |  |
| AT4G39610 | LOC_Os04g54600 |  |  |  |  |  |  |  |  |
| AT4G27790 | LOC_Os04g58480 |  |  |  |  |  |  |  |  |
| AT2G33420 | LOC_Os03g04560 | LOC_Os10g33240 |  |  |  |  |  |  |  |
| AT3G06260 | LOC_Os03g24510 | LOC_Os07g45260 |  |  |  |  |  |  |  |
| AT3G13390 | LOC_Os01g60080 | LOC_Os05g40740 |  |  |  |  |  |  |  |
| AT3G13400 | LOC_Os01g60080 | LOC_Os05g40740 |  |  |  |  |  |  |  |
| AT3G21180 | LOC_Os04g51610 |  |  |  |  |  |  |  |  |
| AT3G06830 | LOC_Os04g38560 |  |  |  |  |  |  |  |  |
| AT3G01270 | LOC_Os02g12300 | LOC_Os06g38510 |  |  |  |  |  |  |  |
| AT1G66210 | LOC_Os02g16940 | LOC_Os02g17090 | LOC_Os02g17150 |  |  |  |  |  |  |
| AT1G79860 | LOC_Os02g17240 |  |  |  |  |  |  |  |  |
| AT1G74000 | LOC_Os09g20684 |  |  |  |  |  |  |  |  |
| AT1G50610 | LOC_Os08g40990 |  |  |  |  |  |  |  |  |
| AT1G65970 | LOC_Os01g48420 |  |  |  |  |  |  |  |  |
| AT4G36490 | LOC_Os02g04030 | LOC_Os02g24430 | LOC_Os10g03400 |  |  |  |  |  |  |
| AT5G57690 | LOC_Os08g15090 |  |  |  |  |  |  |  |  |
| AT5G13990 | LOC_Os11g06700 | LOC_Os12g06840 |  |  |  |  |  |  |  |
| AT3G43860 | LOC_Os02g53820 |  |  |  |  |  |  |  |  |
| AT4G28000 | LOC_Os01g12660 |  |  |  |  |  |  |  |  |
| AT4G25780 | LOC_Os02g54560 |  |  |  |  |  |  |  |  |
| AT4G04930 | LOC_Os02g42660 |  |  |  |  |  |  |  |  |
| AT4G00350 | LOC_Os08g44870 |  |  |  |  |  |  |  |  |
| AT3G20580 | LOC_Os06g47110 |  |  |  |  |  |  |  |  |
| AT2G21990 | LOC_Os04g54600 |  |  |  |  |  |  |  |  |
| Locus number | Orthologue |  |  |  |  |  |  |  |  |
| AT1G74010 | LOC_Os09g20684 |  |  |  |  |  |  |  |  |
| AT1G04540 | LOC_Os02g57000 |  |  |  |  |  |  |  |  |
| AT2G16730 | LOC_Os08g43570 |  |  |  |  |  |  |  |  |
| AT2G26450 | LOC_Os08g34910 | LOC_Os09g26360 |  |  |  |  |  |  |  |
| AT2G33100 | LOC_Os06g02180 | LOC_Os06g22980 | LOC_Os08g25710 | LOC_Os10g42750 | LOC_Os12g36890 |  |  |  |  |
| AT5G20390 | LOC_Os01g58730 |  |  |  |  |  |  |  |  |
| AT5G64790 | LOC_Os06g39060 |  |  |  |  |  |  |  |  |
| AT5G45880 | LOC_Os04g32680 | LOC_Os06g36240 |  |  |  |  |  |  |  |
| AT3G47440 | LOC_Os04g46490 |  |  |  |  |  |  |  |  |
| AT3G43120 | LOC_Os09g26610 |  |  |  |  |  |  |  |  |
| AT4G39110 | LOC_Os06g03610 |  |  |  |  |  |  |  |  |
| AT3G20220 | LOC_Os02g05050 | LOC_Os06g48860 |  |  |  |  |  |  |  |
| AT3G07850 | LOC_Os06g35320 | LOC_Os06g35370 |  |  |  |  |  |  |  |
| AT1G29140 | LOC_Os04g32680 | LOC_Os06g36240 |  |  |  |  |  |  |  |
| AT2G22180 | LOC_Os03g09160 |  |  |  |  |  |  |  |  |
| AT2G24370 | LOC_Os02g54590 | LOC_Os06g09230 |  |  |  |  |  |  |  |
| AT2G23900 | LOC_Os03g03350 |  |  |  |  |  |  |  |  |
| AT2G45800 | LOC_Os02g42820 |  |  |  |  |  |  |  |  |
| AT5G56640 | LOC_Os06g36560 |  |  |  |  |  |  |  |  |
| AT5G48140 | LOC_Os06g35320 | LOC_Os06g35370 |  |  |  |  |  |  |  |
| AT5G47000 | LOC_Os02g50770 |  |  |  |  |  |  |  |  |
| AT5G24240 | LOC_Os02g18840 | LOC_Os05g51230 | LOC_Os06g23290 |  |  |  |  |  |  |
| AT3G52600 | LOC_Os04g33720 |  |  |  |  |  |  |  |  |
| AT4G02250 | LOC_Os01g20970 |  |  |  |  |  |  |  |  |
| AT3G19020 | LOC_Os01g25460 | LOC_Os12g35710 |  |  |  |  |  |  |  |
| AT3G03430 | LOC_Os08g44660 |  |  |  |  |  |  |  |  |
| Locus number | Orthologue |  |  |  |  |  |  |  |  |
| AT1G49490 | LOC_Os01g25460 | LOC_Os12g35710 |  |  |  |  |  |  |  |
| AT2G26490 | LOC_Os03g52870 |  |  |  |  |  |  |  |  |
| AT5G15110 | LOC_Os02g12300 | LOC_Os06g38510 |  |  |  |  |  |  |  |
| AT3G45280 | LOC_Os01g49150 | LOC_Os05g48020 |  |  |  |  |  |  |  |
| AT4G33970 | LOC_Os01g25460 | LOC_Os12g35710 |  |  |  |  |  |  |  |
| AT2G24450 | LOC_Os02g26320 |  |  |  |  |  |  |  |  |
| AT2G19770 | LOC_Os10g17660 | LOC_Os10g17680 |  |  |  |  |  |  |  |
| AT4G35010 | LOC_Os08g43570 |  |  |  |  |  |  |  |  |
| AT3G07820 | LOC_Os06g35320 | LOC_Os06g35370 |  |  |  |  |  |  |  |
| AT1G55570 | LOC_Os01g60080 | LOC_Os05g40740 |  |  |  |  |  |  |  |
| AT5G27870 | LOC_Os03g18860 |  |  |  |  |  |  |  |  |
| AT1G55560 | LOC_Os01g60080 | LOC_Os05g40740 |  |  |  |  |  |  |  |
| AT1G14420 | LOC_Os02g12300 | LOC_Os06g38510 |  |  |  |  |  |  |  |
| AT5G14890 | LOC_Os02g49770 |  |  |  |  |  |  |  |  |
| AT5G58050 | LOC_Os02g09450 |  |  |  |  |  |  |  |  |
| AT5G20410 | LOC_Os02g55910 |  |  |  |  |  |  |  |  |
| AT3G05610 | LOC_Os03g18860 |  |  |  |  |  |  |  |  |
| AT2G02720 | LOC_Os02g12300 | LOC_Os06g38510 |  |  |  |  |  |  |  |
| AT3G17060 | LOC_Os03g19610 |  |  |  |  |  |  |  |  |
| AT1G54070 | LOC_Os08g35190 |  |  |  |  |  |  |  |  |
| AT3G27330 | LOC_Os08g02850 |  |  |  |  |  |  |  |  |
| AT2G44190 | LOC_Os09g11440 |  |  |  |  |  |  |  |  |
| AT5G45700 | LOC_Os05g11570 |  |  |  |  |  |  |  |  |
| AT5G05510 | LOC_Os11g03460 | LOC_Os12g03180 |  |  |  |  |  |  |  |
| AT4G31020 | LOC_Os02g55330 |  |  |  |  |  |  |  |  |
| AT1G14970 | LOC_Os05g38490 |  |  |  |  |  |  |  |  |
| Locus number | Orthologue |  |  |  |  |  |  |  |  |
| AT1G26840 | LOC_Os07g43540 |  |  |  |  |  |  |  |  |
| AT5G21150 | LOC_Os01g16870 | LOC_Os04g06770 |  |  |  |  |  |  |  |
| AT5G15070 | LOC_Os01g56980 | LOC_Os03g48300 |  |  |  |  |  |  |  |
| AT5G58760 | LOC_Os01g04870 |  |  |  |  |  |  |  |  |
| AT4G11920 | LOC_Os03g03150 |  |  |  |  |  |  |  |  |
| AT4G02900 | LOC_Os05g32720 |  |  |  |  |  |  |  |  |
| AT1G52920 | LOC_Os04g44730 | LOC_Os10g35480 |  |  |  |  |  |  |  |
| AT1G04160 | LOC_Os02g53740 | LOC_Os02g57190 | LOC_Os03g48140 | LOC_Os03g53660 | LOC_Os03g64290 | LOC_Os05g46030 | LOC_Os06g29350 | LOC_Os10g19860 |  |
| AT1G65590 | LOC_Os01g66700 | LOC_Os05g34320 |  |  |  |  |  |  |  |
| AT1G03457 | LOC_Os01g71200 |  |  |  |  |  |  |  |  |
| AT2G36660 | LOC_Os03g17030 |  |  |  |  |  |  |  |  |
| AT2G06040 | LOC_Os08g35700 |  |  |  |  |  |  |  |  |
| AT2G43900 | LOC_Os08g41270 | LOC_Os09g32440 |  |  |  |  |  |  |  |
| AT2G32670 | LOC_Os07g09600 |  |  |  |  |  |  |  |  |
| AT4G25610 | LOC_Os09g39660 |  |  |  |  |  |  |  |  |
| AT5G49680 | LOC_Os03g23030 | LOC_Os03g47754 | LOC_Os03g47760 |  |  |  |  |  |  |
| AT5G38450 | LOC_Os08g33300 | LOC_Os09g23820 |  |  |  |  |  |  |  |
| AT5G17780 | LOC_Os07g48610 |  |  |  |  |  |  |  |  |
| AT5G02430 | LOC_Os12g07874 |  |  |  |  |  |  |  |  |
| AT3G58160 | LOC_Os02g53740 | LOC_Os02g57190 | LOC_Os03g48140 | LOC_Os03g53660 | LOC_Os03g64290 | LOC_Os05g46030 | LOC_Os06g29350 | LOC_Os10g19860 |  |
| AT3G45870 | LOC_Os02g47500 |  |  |  |  |  |  |  |  |
| AT4G01470 | LOC_Os01g74450 |  |  |  |  |  |  |  |  |
| AT3G11020 | LOC_Os05g27930 |  |  |  |  |  |  |  |  |
| AT3G14070 | LOC_Os03g08230 |  |  |  |  |  |  |  |  |
| AT3G19230 | LOC_Os03g08610 | LOC_Os09g28470 |  |  |  |  |  |  |  |
| AT3G04530 | LOC_Os02g56310 |  |  |  |  |  |  |  |  |
| Locus number | Orthologue |  |  |  |  |  |  |  |  |
| AT1G72460 | LOC_Os06g09860 |  |  |  |  |  |  |  |  |
| AT1G07410 | LOC_Os03g60870 |  |  |  |  |  |  |  |  |
| AT1G08730 | LOC_Os02g53740 | LOC_Os02g57190 | LOC_Os03g48140 | LOC_Os03g53660 | LOC_Os03g64290 | LOC_Os05g46030 | LOC_Os06g29350 | LOC_Os10g19860 |  |
| AT1G24330 | LOC_Os01g67500 |  |  |  |  |  |  |  |  |
| AT2G33240 | LOC_Os02g53740 | LOC_Os02g57190 | LOC_Os03g48140 | LOC_Os03g53660 | LOC_Os03g64290 | LOC_Os05g46030 | LOC_Os06g29350 | LOC_Os10g19860 |  |
| AT5G57830 | LOC_Os08g34340 |  |  |  |  |  |  |  |  |
| AT5G55950 | LOC_Os02g49260 |  |  |  |  |  |  |  |  |
| AT5G24560 | LOC_Os02g56750 | LOC_Os02g56840 | LOC_Os12g03594 | LOC_Os12g03740 |  |  |  |  |  |
| AT5G01180 | LOC_Os01g04950 |  |  |  |  |  |  |  |  |
| AT4G27370 | LOC_Os07g37560 | LOC_Os10g34710 |  |  |  |  |  |  |  |
| AT1G17830 | LOC_Os10g35150 |  |  |  |  |  |  |  |  |
| AT3G30380 | LOC_Os02g09770 |  |  |  |  |  |  |  |  |
| AT3G23870 | LOC_Os04g30450 |  |  |  |  |  |  |  |  |
| AT3G17630 | LOC_Os05g19500 |  |  |  |  |  |  |  |  |
| AT1G49160 | LOC_Os02g45130 |  |  |  |  |  |  |  |  |
| AT2G31540 | LOC_Os01g61200 |  |  |  |  |  |  |  |  |
| AT2G22560 | LOC_Os01g74510 |  |  |  |  |  |  |  |  |
| AT2G06005 | LOC_Os02g53780 |  |  |  |  |  |  |  |  |
| AT5G57190 | LOC_Os01g72940 |  |  |  |  |  |  |  |  |
| AT4G35380 | LOC_Os03g14260 |  |  |  |  |  |  |  |  |
| AT4G26830 | LOC_Os02g53200 |  |  |  |  |  |  |  |  |
| AT4G18050 | LOC_Os01g18670 | LOC_Os01g34970 | LOC_Os01g35030 | LOC_Os01g50080 | LOC_Os01g50100 | LOC_Os01g50160 | LOC_Os05g47490 | LOC_Os05g47500 |  |
| AT1G19940 | LOC_Os06g50140 | LOC_Os09g36350 |  |  |  |  |  |  |  |
| AT1G75110 | LOC_Os07g39840 |  |  |  |  |  |  |  |  |
| AT1G79900 | LOC_Os01g12520 |  |  |  |  |  |  |  |  |
| AT1G16290 | LOC_Os02g07480 |  |  |  |  |  |  |  |  |
| Locus number | Orthologue |  |  |  |  |  |  |  |  |
| AT1G04090 | LOC_Os03g04930 | LOC_Os03g51150 |  |  |  |  |  |  |  |
| AT1G03620 | LOC_Os04g01150 |  |  |  |  |  |  |  |  |
| AT2G41210 | LOC_Os07g46490 |  |  |  |  |  |  |  |  |
| AT5G35410 | LOC_Os06g40370 |  |  |  |  |  |  |  |  |
| AT5G56780 | LOC_Os07g39800 |  |  |  |  |  |  |  |  |
| AT5G13700 | LOC_Os09g20260 |  |  |  |  |  |  |  |  |
| AT5G09800 | LOC_Os02g33680 | LOC_Os04g34140 |  |  |  |  |  |  |  |
| AT3G56960 | LOC_Os07g46490 |  |  |  |  |  |  |  |  |
| AT3G47780 | LOC_Os08g30770 |  |  |  |  |  |  |  |  |
| AT4G37990 | LOC_Os09g23540 | LOC_Os09g23550 |  |  |  |  |  |  |  |
| AT4G25150 | LOC_Os06g04790 |  |  |  |  |  |  |  |  |
| AT4G07960 | LOC_Os05g43530 | LOC_Os07g03260 |  |  |  |  |  |  |  |
| AT4G02660 | LOC_Os03g53280 |  |  |  |  |  |  |  |  |
| AT3G09550 | LOC_Os03g17240 |  |  |  |  |  |  |  |  |
| AT1G64300 | LOC_Os01g43350 | LOC_Os05g50830 |  |  |  |  |  |  |  |
| AT1G08170 | LOC_Os09g39730 |  |  |  |  |  |  |  |  |
| AT1G73010 | LOC_Os01g52230 |  |  |  |  |  |  |  |  |
| AT2G31830 | LOC_Os08g41270 | LOC_Os09g32440 |  |  |  |  |  |  |  |
| AT1G54560 | LOC_Os02g53740 | LOC_Os02g57190 | LOC_Os03g48140 | LOC_Os03g53660 | LOC_Os03g64290 | LOC_Os05g46030 | LOC_Os06g29350 | LOC_Os10g19860 |  |
| AT1G09600 | LOC_Os08g02050 |  |  |  |  |  |  |  |  |
| AT1G70170 | LOC_Os02g50730 | LOC_Os06g13180 |  |  |  |  |  |  |  |
| AT2G37980 | LOC_Os01g63230 | LOC_Os05g37880 |  |  |  |  |  |  |  |
| AT2G19400 | LOC_Os01g09200 |  |  |  |  |  |  |  |  |
| AT2G19330 | LOC_Os04g51580 |  |  |  |  |  |  |  |  |
| AT4G17690 | LOC_Os02g50770 |  |  |  |  |  |  |  |  |
| AT1G26320 | LOC_Os04g41960 | LOC_Os12g12470 | LOC_Os12g12560 | LOC_Os12g12580 | LOC_Os12g12590 |  |  |  |  |
| Locus number | Orthologue |  |  |  |  |  |  |  |  |
| AT5G21050 | LOC_Os03g51210 |  |  |  |  |  |  |  |  |
| AT5G15100 | LOC_Os01g51780 |  |  |  |  |  |  |  |  |
| AT5G65090 | LOC_Os02g51600 |  |  |  |  |  |  |  |  |
| AT5G61730 | LOC_Os08g30740 |  |  |  |  |  |  |  |  |
| AT5G60250 | LOC_Os11g01240 | LOC_Os12g01230 |  |  |  |  |  |  |  |
| AT5G57320 | LOC_Os04g51440 | LOC_Os06g44890 |  |  |  |  |  |  |  |
| AT5G37810 | LOC_Os05g11560 |  |  |  |  |  |  |  |  |
| AT5G01610 | LOC_Os05g27950 |  |  |  |  |  |  |  |  |
| AT3G13900 | LOC_Os05g01030 | LOC_Os06g29380 | LOC_Os06g36990 | LOC_Os08g29150 |  |  |  |  |  |
| AT3G01015 | LOC_Os03g11400 |  |  |  |  |  |  |  |  |
| AT1G64110 | LOC_Os01g12660 |  |  |  |  |  |  |  |  |
| AT1G03920 | LOC_Os10g33640 |  |  |  |  |  |  |  |  |
| AT3G54700 | LOC_Os04g10690 | LOC_Os04g10750 | LOC_Os08g45000 | LOC_Os10g30790 |  |  |  |  |  |
| AT3G46230 | LOC_Os01g04360 | LOC_Os01g04370 | LOC_Os01g04380 | LOC_Os03g15960 | LOC_Os03g16030 |  |  |  |  |
| AT3G02850 | LOC_Os06g14030 |  |  |  |  |  |  |  |  |
| AT3G10470 | LOC_Os02g57790 |  |  |  |  |  |  |  |  |
| AT2G13680 | LOC_Os01g34890 | LOC_Os01g55040 | LOC_Os02g14900 | LOC_Os02g58560 | LOC_Os03g03610 | LOC_Os06g08380 | LOC_Os06g51270 |  |  |
| AT5G61700 | LOC_Os08g30770 |  |  |  |  |  |  |  |  |
| AT5G01700 | LOC_Os11g01790 | LOC_Os12g01770 |  |  |  |  |  |  |  |
| AT3G60330 | LOC_Os02g55400 | LOC_Os03g01120 | LOC_Os03g08560 | LOC_Os03g48310 | LOC_Os04g56160 | LOC_Os05g25550 | LOC_Os06g08310 | LOC_Os07g09340 | LOC_Os12g44150 |
| AT4G26470 | LOC_Os06g40720 |  |  |  |  |  |  |  |  |
| AT2G20430 | LOC_Os07g26480 |  |  |  |  |  |  |  |  |
| AT3G07960 | LOC_Os07g46490 |  |  |  |  |  |  |  |  |
| AT1G17710 | LOC_Os01g52230 |  |  |  |  |  |  |  |  |
| AT1G67640 | LOC_Os12g14100 |  |  |  |  |  |  |  |  |
| AT1G53540 | LOC_Os01g04360 | LOC_Os01g04370 | LOC_Os01g04380 | LOC_Os03g15960 | LOC_Os03g16030 |  |  |  |  |
| Locus number | Orthologue |  |  |  |  |  |  |  |  |
| AT2G29940 | LOC_Os01g08260 | LOC_Os01g24010 | LOC_Os01g42350 | LOC_Os01g42370 | LOC_Os01g42380 | LOC_Os01g42410 | LOC_Os01g52560 | LOC_Os02g11760 | LOC_Os02g21340 |
|  | LOC_Os02g32690 | LOC_Os06g36090 | LOC_Os08g29570 | LOC_Os08g43120 | LOC_Os09g16290 | LOC_Os09g16330 | LOC_Os09g16380 | LOC_Os11g37700 | LOC_Os12g13720 |
| AT2G22950 | LOC_Os01g71240 | LOC_Os03g10640 | LOC_Os03g42020 | LOC_Os05g41580 | LOC_Os11g04460 | LOC_Os12g04220 | LOC_Os12g39660 |  |  |
| AT5G62850 | LOC_Os05g51090 |  |  |  |  |  |  |  |  |
| AT3G03080 | LOC_Os04g41960 | LOC_Os12g12470 | LOC_Os12g12560 | LOC_Os12g12580 | LOC_Os12g12590 |  |  |  |  |
| AT1G71380 | LOC_Os04g36610 |  |  |  |  |  |  |  |  |
| AT2G37010 | LOC_Os04g11820 | LOC_Os06g51460 | LOC_Os11g22350 |  |  |  |  |  |  |
| AT2G24320 | LOC_Os02g55330 |  |  |  |  |  |  |  |  |
| AT5G47470 | LOC_Os06g01660 | LOC_Os07g30210 |  |  |  |  |  |  |  |
| AT1G04880 | LOC_Os02g27060 |  |  |  |  |  |  |  |  |
| AT1G22460 | LOC_Os06g11600 |  |  |  |  |  |  |  |  |
| AT1G43630 | LOC_Os11g06440 | LOC_Os12g06780 |  |  |  |  |  |  |  |
| AT1G04600 | LOC_Os02g53740 | LOC_Os02g57190 | LOC_Os03g48140 | LOC_Os03g53660 | LOC_Os03g64290 | LOC_Os05g46030 | LOC_Os06g29350 | LOC_Os10g19860 |  |
| AT2G45750 | LOC_Os02g45310 | LOC_Os04g48230 |  |  |  |  |  |  |  |
| AT5G25880 | LOC_Os01g52500 | LOC_Os05g09440 |  |  |  |  |  |  |  |
| AT5G60010 | LOC_Os01g61880 | LOC_Os05g38980 |  |  |  |  |  |  |  |
| AT4G38190 | LOC_Os06g02180 | LOC_Os06g22980 | LOC_Os08g25710 | LOC_Os10g42750 | LOC_Os12g36890 |  |  |  |  |
| AT4G26730 | LOC_Os01g57620 | LOC_Os05g42140 |  |  |  |  |  |  |  |
| AT3G28210 | LOC_Os09g21710 |  |  |  |  |  |  |  |  |
| AT2G36190 | LOC_Os04g33720 |  |  |  |  |  |  |  |  |
| AT4G14780 | LOC_Os03g43760 |  |  |  |  |  |  |  |  |
| AT1G69430 | LOC_Os07g40470 |  |  |  |  |  |  |  |  |
| AT1G29630 | LOC_Os01g56940 |  |  |  |  |  |  |  |  |
| AT5G14870 | LOC_Os12g06570 |  |  |  |  |  |  |  |  |
| AT3G01620 | LOC_Os02g38140 | LOC_Os02g38160 | LOC_Os04g40150 |  |  |  |  |  |  |
| AT5G57240 | LOC_Os08g40590 |  |  |  |  |  |  |  |  |
| Locus number | Orthologue |  |  |  |  |  |  |  |  |
| AT5G20340 | LOC_Os01g58730 |  |  |  |  |  |  |  |  |
| AT5G04390 | LOC_Os02g57790 |  |  |  |  |  |  |  |  |
| AT5G60740 | LOC_Os04g11820 | LOC_Os06g51460 | LOC_Os11g22350 |  |  |  |  |  |  |
| AT3G05950 | LOC_Os08g08990 | LOC_Os08g09000 | LOC_Os08g09010 | LOC_Os08g09020 | LOC_Os08g09040 | LOC_Os08g09060 | LOC_Os08g09080 |  |  |
| AT2G33670 | LOC_Os05g09050 |  |  |  |  |  |  |  |  |
| AT5G55490 | LOC_Os09g27040 |  |  |  |  |  |  |  |  |
| AT3G44400 | LOC_Os01g71106 |  |  |  |  |  |  |  |  |
| AT4G35280 | LOC_Os02g19180 |  |  |  |  |  |  |  |  |
| AT4G25940 | LOC_Os02g07900 |  |  |  |  |  |  |  |  |
| AT2G33350 | LOC_Os03g04620 | LOC_Os10g32900 |  |  |  |  |  |  |  |
| AT2G03060 | LOC_Os11g43740 |  |  |  |  |  |  |  |  |
| AT5G62310 | LOC_Os12g42660 |  |  |  |  |  |  |  |  |
| AT4G00240 | LOC_Os03g02740 | LOC_Os10g38060 |  |  |  |  |  |  |  |
| AT3G05960 | LOC_Os07g10590 |  |  |  |  |  |  |  |  |
| AT1G14640 | LOC_Os02g14780 |  |  |  |  |  |  |  |  |
| AT2G38910 | LOC_Os03g57510 | LOC_Os12g30150 |  |  |  |  |  |  |  |
| AT2G47550 | LOC_Os01g20980 | LOC_Os05g29790 |  |  |  |  |  |  |  |
| AT5G25400 | LOC_Os05g41480 |  |  |  |  |  |  |  |  |
| AT3G51070 | LOC_Os06g49860 |  |  |  |  |  |  |  |  |
| AT1G04500 | LOC_Os03g04620 | LOC_Os10g32900 |  |  |  |  |  |  |  |
| AT2G21510 | LOC_Os05g46620 |  |  |  |  |  |  |  |  |
| AT5G20690 | LOC_Os06g09860 |  |  |  |  |  |  |  |  |
| AT5G35390 | LOC_Os08g40990 |  |  |  |  |  |  |  |  |
| AT5G66020 | LOC_Os02g34884 |  |  |  |  |  |  |  |  |
| AT5G14180 | LOC_Os08g41780 |  |  |  |  |  |  |  |  |
| AT5G02390 | LOC_Os12g38260 |  |  |  |  |  |  |  |  |
| Locus number | Orthologue |  |  |  |  |  |  |  |  |
| AT3G60580 | LOC_Os04g46680 | LOC_Os10g40660 |  |  |  |  |  |  |  |
| AT3G55940 | LOC_Os07g49330 |  |  |  |  |  |  |  |  |
| AT3G12060 | LOC_Os02g53380 | LOC_Os06g10560 |  |  |  |  |  |  |  |
| AT3G10660 | LOC_Os03g57450 |  |  |  |  |  |  |  |  |
| AT1G77980 | LOC_Os06g11970 |  |  |  |  |  |  |  |  |
| AT2G05160 | LOC_Os08g03310 |  |  |  |  |  |  |  |  |
| AT1G61700 | LOC_Os11g08940 |  |  |  |  |  |  |  |  |
| AT5G51030 | LOC_Os01g70430 |  |  |  |  |  |  |  |  |
| AT4G13240 | LOC_Os05g48640 |  |  |  |  |  |  |  |  |
| AT4G13450 | LOC_Os10g32590 |  |  |  |  |  |  |  |  |
| AT1G79400 | LOC_Os02g58660 |  |  |  |  |  |  |  |  |
| AT2G41970 | LOC_Os02g35760 | LOC_Os03g51040 |  |  |  |  |  |  |  |
| AT1G44120 | LOC_Os06g11990 | LOC_Os11g08090 |  |  |  |  |  |  |  |
| AT5G27610 | LOC_Os03g43800 |  |  |  |  |  |  |  |  |
| AT5G65530 | LOC_Os06g45350 |  |  |  |  |  |  |  |  |
| AT3G11180 | LOC_Os01g61610 | LOC_Os03g18030 | LOC_Os05g03640 |  |  |  |  |  |  |
| AT1G79250 | LOC_Os01g13270 | LOC_Os05g14750 |  |  |  |  |  |  |  |
| AT1G24110 | LOC_Os02g50770 |  |  |  |  |  |  |  |  |
| AT5G20810 | LOC_Os09g26610 |  |  |  |  |  |  |  |  |
| AT5G25530 | LOC_Os08g06460 |  |  |  |  |  |  |  |  |
| AT3G28150 | LOC_Os06g12820 |  |  |  |  |  |  |  |  |
| AT1G75050 | LOC_Os03g13070 |  |  |  |  |  |  |  |  |
| AT1G78940 | LOC_Os02g54590 | LOC_Os06g09230 |  |  |  |  |  |  |  |
| AT4G16745 | LOC_Os01g01780 |  |  |  |  |  |  |  |  |
| AT4G34940 | LOC_Os08g43500 | LOC_Os09g36550 |  |  |  |  |  |  |  |
| AT4G30770 | LOC_Os12g08150 |  |  |  |  |  |  |  |  |
| Locus number | Orthologue |  |  |  |  |  |  |  |  |
| AT4G27110 | LOC_Os06g47110 |  |  |  |  |  |  |  |  |
| AT1G11040 | LOC_Os01g65480 |  |  |  |  |  |  |  |  |
| AT1G11250 | LOC_Os06g39050 |  |  |  |  |  |  |  |  |
| AT5G45840 | LOC_Os01g10900 | LOC_Os01g48390 |  |  |  |  |  |  |  |
| AT5G12030 | LOC_Os01g08860 |  |  |  |  |  |  |  |  |
| AT3G55180 | LOC_Os12g16180 |  |  |  |  |  |  |  |  |
| AT5G61350 | LOC_Os03g55210 |  |  |  |  |  |  |  |  |
| AT5G57200 | LOC_Os02g07900 |  |  |  |  |  |  |  |  |
| AT5G09550 | LOC_Os03g16900 |  |  |  |  |  |  |  |  |
| AT3G11740 | LOC_Os07g12730 |  |  |  |  |  |  |  |  |
| AT3G48450 | LOC_Os02g49340 |  |  |  |  |  |  |  |  |
| AT4G39180 | LOC_Os02g04030 | LOC_Os02g24430 | LOC_Os10g03400 |  |  |  |  |  |  |
| AT4G29340 | LOC_Os10g17660 | LOC_Os10g17680 |  |  |  |  |  |  |  |
| AT1G75160 | LOC_Os01g55795 |  |  |  |  |  |  |  |  |
| AT2G25630 | LOC_Os04g39880 |  |  |  |  |  |  |  |  |
| AT5G65160 | LOC_Os02g51730 |  |  |  |  |  |  |  |  |
| AT4G26330 | LOC_Os03g06290 |  |  |  |  |  |  |  |  |
| AT4G33230 | LOC_Os08g34910 | LOC_Os09g26360 |  |  |  |  |  |  |  |
| AT1G54280 | LOC_Os05g01030 | LOC_Os06g29380 | LOC_Os06g36990 | LOC_Os08g29150 |  |  |  |  |  |
| AT1G68750 | LOC_Os01g02050 |  |  |  |  |  |  |  |  |
| AT5G42340 | LOC_Os06g51130 | LOC_Os08g37570 |  |  |  |  |  |  |  |

**Table S9**. Assignment of *Arabidopsis* orthologs to rice late pollen-preferred genes. Locus numbers are shown in red.

| Locus number | Orthologue |  |  |  |  |  |  |  |  |  |  |  |  |
| --- | --- | --- | --- | --- | --- | --- | --- | --- | --- | --- | --- | --- | --- |
| LOC_Os10g33240 | AT1G04470 | AT2G33420 |  |  |  |  |  |  |  |  |  |  |  |
| LOC_Os03g05770 | AT1G34510 | AT4G26010 |  |  |  |  |  |  |  |  |  |  |  |
| LOC_Os01g60080 | AT1G55560 | AT1G55570 | AT3G13390 | AT3G13400 |  |  |  |  |  |  |  |  |  |
| LOC_Os06g35590 | AT4G20820 | AT4G20860 | AT5G44360 | AT5G44410 | AT5G44440 |  |  |  |  |  |  |  |  |
| LOC_Os10g17660 | AT2G19770 | AT4G29340 |  |  |  |  |  |  |  |  |  |  |  |
| LOC_Os04g37460 | AT3G17760 |  |  |  |  |  |  |  |  |  |  |  |  |
| LOC_Os02g51730 | AT5G10090 | AT5G65160 |  |  |  |  |  |  |  |  |  |  |  |
| LOC_Os03g19610 | AT3G17060 |  |  |  |  |  |  |  |  |  |  |  |  |
| LOC_Os06g38510 | AT1G14420 | AT2G02720 | AT3G01270 | AT5G15110 |  |  |  |  |  |  |  |  |  |
| LOC_Os01g10440 | AT3G15350 | AT4G27480 |  |  |  |  |  |  |  |  |  |  |  |
| LOC_Os08g34340 | AT2G24140 | AT4G30830 | AT5G57830 |  |  |  |  |  |  |  |  |  |  |
| LOC_Os04g46910 | AT1G01750 | AT4G00680 | AT4G25590 |  |  |  |  |  |  |  |  |  |  |
| LOC_Os05g46740 | AT3G23270 | AT4G14368 |  |  |  |  |  |  |  |  |  |  |  |
| LOC_Os08g37570 | AT5G42340 |  |  |  |  |  |  |  |  |  |  |  |  |
| LOC_Os07g34130 | AT4G27100 | AT5G54780 |  |  |  |  |  |  |  |  |  |  |  |
| LOC_Os01g46850 | AT2G46330 | AT3G61640 |  |  |  |  |  |  |  |  |  |  |  |
| LOC_Os10g32900 | AT1G04500 | AT2G33350 |  |  |  |  |  |  |  |  |  |  |  |
| LOC_Os03g12180 | AT1G22730 |  |  |  |  |  |  |  |  |  |  |  |  |
| LOC_Os01g71240 | AT1G27770 | AT2G22950 | AT2G41560 | AT3G57330 | AT4G37640 |  |  |  |  |  |  |  |  |
| LOC_Os01g13270 | AT1G16440 | AT1G79250 | AT3G12690 |  |  |  |  |  |  |  |  |  |  |
| LOC_Os07g02780 | AT5G66850 |  |  |  |  |  |  |  |  |  |  |  |  |
| LOC_Os01g25460 | AT1G49490 | AT2G15880 | AT3G19020 | AT4G33970 |  |  |  |  |  |  |  |  |  |
| Locus number | Orthologue |  |  |  |  |  |  |  |  |  |  |  |  |
| LOC_Os10g08022 | AT2G36460 | AT3G52930 |  |  |  |  |  |  |  |  |  |  |  |
| LOC_Os04g54600 | AT2G21990 | AT4G39610 |  |  |  |  |  |  |  |  |  |  |  |
| LOC_Os06g39060 | AT5G64790 |  |  |  |  |  |  |  |  |  |  |  |  |
| LOC_Os03g59440 | AT2G21110 | AT4G38700 |  |  |  |  |  |  |  |  |  |  |  |
| LOC_Os01g39970 | AT5G12000 | AT5G26150 |  |  |  |  |  |  |  |  |  |  |  |
| LOC_Os03g52870 | AT2G26490 |  |  |  |  |  |  |  |  |  |  |  |  |
| LOC_Os12g42660 | AT5G62310 |  |  |  |  |  |  |  |  |  |  |  |  |
| LOC_Os03g18360 | AT3G51770 |  |  |  |  |  |  |  |  |  |  |  |  |
| LOC_Os07g17120 | AT2G44060 |  |  |  |  |  |  |  |  |  |  |  |  |
| LOC_Os03g10460 | AT2G18410 |  |  |  |  |  |  |  |  |  |  |  |  |
| LOC_Os06g50140 | AT1G19940 | AT1G75680 |  |  |  |  |  |  |  |  |  |  |  |
| LOC_Os08g39370 | AT5G47560 |  |  |  |  |  |  |  |  |  |  |  |  |
| LOC_Os11g22350 | AT1G53390 | AT2G37010 | AT5G60740 |  |  |  |  |  |  |  |  |  |  |
| LOC_Os05g50170 | AT1G26120 | AT3G02410 | AT5G15860 |  |  |  |  |  |  |  |  |  |  |
| LOC_Os07g10550 | AT2G41830 | AT5G21080 |  |  |  |  |  |  |  |  |  |  |  |
| LOC_Os03g51210 | AT5G21050 |  |  |  |  |  |  |  |  |  |  |  |  |
| LOC_Os03g18860 | AT3G05610 | AT5G27870 |  |  |  |  |  |  |  |  |  |  |  |
| LOC_Os12g41170 | AT1G72960 | AT3G13870 |  |  |  |  |  |  |  |  |  |  |  |
| LOC_Os08g04890 | AT4G20780 | AT5G44460 |  |  |  |  |  |  |  |  |  |  |  |
| LOC_Os03g24510 | AT3G06260 |  |  |  |  |  |  |  |  |  |  |  |  |
| LOC_Os05g38980 | AT3G45810 | AT5G60010 |  |  |  |  |  |  |  |  |  |  |  |
| LOC_Os06g36240 | AT1G29140 | AT4G18596 | AT5G45880 |  |  |  |  |  |  |  |  |  |  |
| LOC_Os02g35760 | AT2G41970 |  |  |  |  |  |  |  |  |  |  |  |  |
| LOC_Os04g51440 | AT4G30160 | AT5G57320 |  |  |  |  |  |  |  |  |  |  |  |
| LOC_Os08g38610 | AT1G26900 |  |  |  |  |  |  |  |  |  |  |  |  |
| LOC_Os05g40230 | AT3G08560 |  |  |  |  |  |  |  |  |  |  |  |  |
| Locus number | Orthologue |  |  |  |  |  |  |  |  |  |  |  |  |
| LOC_Os02g09450 | AT5G58050 | AT5G58170 |  |  |  |  |  |  |  |  |  |  |  |
| LOC_Os02g50770 | AT1G24110 | AT4G17690 | AT5G47000 |  |  |  |  |  |  |  |  |  |  |
| LOC_Os09g32440 | AT1G05630 | AT1G65580 | AT2G31830 | AT2G43900 |  |  |  |  |  |  |  |  |  |
| LOC_Os04g57750 | AT1G48940 | AT3G18590 |  |  |  |  |  |  |  |  |  |  |  |
| LOC_Os05g51090 | AT5G62850 |  |  |  |  |  |  |  |  |  |  |  |  |
| LOC_Os02g07900 | AT4G25940 | AT5G57200 |  |  |  |  |  |  |  |  |  |  |  |
| LOC_Os04g21340 | AT1G08790 | AT3G04700 | AT5G28690 |  |  |  |  |  |  |  |  |  |  |
| LOC_Os05g51230 | AT2G46500 | AT5G24240 |  |  |  |  |  |  |  |  |  |  |  |
| LOC_Os06g35320 | AT3G07820 | AT3G07830 | AT3G07840 | AT3G07850 | AT3G14040 | AT5G48140 |  |  |  |  |  |  |  |
| LOC_Os01g20970 | AT1G55770 | AT4G02250 |  |  |  |  |  |  |  |  |  |  |  |
| LOC_Os04g01150 | AT1G03620 |  |  |  |  |  |  |  |  |  |  |  |  |
| LOC_Os02g17240 | AT1G52240 | AT1G79860 | AT3G16130 |  |  |  |  |  |  |  |  |  |  |
| LOC_Os01g51610 | AT3G26790 |  |  |  |  |  |  |  |  |  |  |  |  |
| LOC_Os03g08610 | AT3G19230 |  |  |  |  |  |  |  |  |  |  |  |  |
| LOC_Os02g04030 | AT2G16380 | AT2G18180 | AT2G21540 | AT4G34580 | AT4G36490 | AT4G39180 |  |  |  |  |  |  |  |
| LOC_Os02g32740 | AT1G13890 | AT5G61210 |  |  |  |  |  |  |  |  |  |  |  |
| LOC_Os08g34900 | AT3G43270 | AT4G33220 |  |  |  |  |  |  |  |  |  |  |  |
| LOC_Os03g04620 | AT1G04500 | AT2G33350 |  |  |  |  |  |  |  |  |  |  |  |
| LOC_Os07g26480 | AT1G03982 | AT2G20430 | AT4G28556 |  |  |  |  |  |  |  |  |  |  |
| LOC_Os02g24430 | AT2G16380 | AT2G18180 | AT2G21540 | AT4G34580 | AT4G36490 | AT4G39180 |  |  |  |  |  |  |  |
| LOC_Os05g41270 | AT5G12180 | AT5G19360 |  |  |  |  |  |  |  |  |  |  |  |
| LOC_Os03g27110 | AT1G78210 | AT4G39955 | AT5G09430 |  |  |  |  |  |  |  |  |  |  |
| LOC_Os05g40740 | AT1G55560 | AT1G55570 | AT3G13390 | AT3G13400 |  |  |  |  |  |  |  |  |  |
| LOC_Os06g08380 | AT1G05570 | AT1G06490 | AT2G13680 | AT2G31960 | AT2G36850 | AT3G07160 | AT3G14570 | AT3G59100 | AT4G03550 | AT4G04970 | AT5G13000 | AT5G36870 |  |
| LOC_Os02g49340 | AT3G48450 | AT5G63270 |  |  |  |  |  |  |  |  |  |  |  |
| LOC_Os11g06690 | AT1G09980 | AT1G58350 |  |  |  |  |  |  |  |  |  |  |  |
| Locus number | Orthologue |  |  |  |  |  |  |  |  |  |  |  |  |
| LOC_Os02g10300 | AT1G17150 | AT1G43080 | AT1G43090 | AT1G43100 | AT1G78400 | AT2G15450 | AT2G15460 | AT2G15470 | AT2G26620 | AT2G33160 | AT2G40310 | AT4G13760 |  |
| LOC_Os04g41250 | AT1G27910 |  |  |  |  |  |  |  |  |  |  |  |  |
| LOC_Os03g04770 | AT3G23920 |  |  |  |  |  |  |  |  |  |  |  |  |
| LOC_Os06g02019 | AT1G05160 | AT2G32440 |  |  |  |  |  |  |  |  |  |  |  |
| LOC_Os06g04790 | AT4G25150 | AT5G51260 |  |  |  |  |  |  |  |  |  |  |  |
| LOC_Os08g44660 | AT3G03430 | AT5G17480 |  |  |  |  |  |  |  |  |  |  |  |
| LOC_Os01g55940 | AT1G59500 | AT2G14960 | AT2G23170 | AT4G37390 |  |  |  |  |  |  |  |  |  |
| LOC_Os01g10890 | AT5G45820 |  |  |  |  |  |  |  |  |  |  |  |  |
| LOC_Os02g17780 | AT4G02780 |  |  |  |  |  |  |  |  |  |  |  |  |
| LOC_Os08g43570 | AT2G16730 | AT4G35010 | AT4G38590 |  |  |  |  |  |  |  |  |  |  |
| LOC_Os04g45290 | AT1G12240 | AT1G62660 |  |  |  |  |  |  |  |  |  |  |  |
| LOC_Os03g62750 | AT1G24490 |  |  |  |  |  |  |  |  |  |  |  |  |
| LOC_Os06g03980 | AT4G11990 | AT4G22860 |  |  |  |  |  |  |  |  |  |  |  |
| LOC_Os06g05160 | AT3G15990 |  |  |  |  |  |  |  |  |  |  |  |  |
| LOC_Os12g41780 | AT2G13290 |  |  |  |  |  |  |  |  |  |  |  |  |
| LOC_Os01g10900 | AT4G18640 | AT5G45840 |  |  |  |  |  |  |  |  |  |  |  |
| LOC_Os08g34780 | AT4G34490 |  |  |  |  |  |  |  |  |  |  |  |  |
| LOC_Os01g02050 | AT1G68750 |  |  |  |  |  |  |  |  |  |  |  |  |
| LOC_Os06g29350 | AT1G04160 | AT1G04600 | AT1G08730 | AT1G17580 | AT1G54560 | AT2G20290 | AT2G31900 | AT2G33240 | AT3G58160 | AT4G28710 | AT4G33200 | AT5G20490 | AT5G43900 |
| LOC_Os02g53990 | AT2G41830 | AT5G21080 |  |  |  |  |  |  |  |  |  |  |  |
| LOC_Os05g31720 | AT3G07940 |  |  |  |  |  |  |  |  |  |  |  |  |
| LOC_Os05g50110 | AT1G48990 | AT3G18570 |  |  |  |  |  |  |  |  |  |  |  |
| LOC_Os02g44470 | AT1G01750 | AT4G00680 | AT4G25590 |  |  |  |  |  |  |  |  |  |  |
| LOC_Os02g05040 | AT2G41830 | AT5G21080 |  |  |  |  |  |  |  |  |  |  |  |
| LOC_Os05g39000 | AT1G09720 | AT1G58210 |  |  |  |  |  |  |  |  |  |  |  |
| LOC_Os01g68900 | AT3G07120 | AT3G25030 | AT4G13100 |  |  |  |  |  |  |  |  |  |  |
| Locus number | Orthologue |  |  |  |  |  |  |  |  |  |  |  |  |
| LOC_Os01g74450 | AT4G01470 |  |  |  |  |  |  |  |  |  |  |  |  |
| LOC_Os02g32060 | AT1G12880 | AT3G26690 |  |  |  |  |  |  |  |  |  |  |  |
| LOC_Os01g61910 | AT1G09720 | AT1G58210 |  |  |  |  |  |  |  |  |  |  |  |
| LOC_Os02g42710 | AT1G63220 |  |  |  |  |  |  |  |  |  |  |  |  |
| LOC_Os02g02560 | AT3G03250 | AT5G17310 |  |  |  |  |  |  |  |  |  |  |  |
| LOC_Os03g16900 | AT5G09550 |  |  |  |  |  |  |  |  |  |  |  |  |
| LOC_Os02g42820 | AT1G01780 | AT2G45800 | AT3G61230 |  |  |  |  |  |  |  |  |  |  |
| LOC_Os03g17030 | AT2G36660 |  |  |  |  |  |  |  |  |  |  |  |  |
| LOC_Os10g32590 | AT4G13450 |  |  |  |  |  |  |  |  |  |  |  |  |
| LOC_Os01g74510 | AT2G22560 |  |  |  |  |  |  |  |  |  |  |  |  |
| LOC_Os12g06840 | AT5G13150 | AT5G13990 |  |  |  |  |  |  |  |  |  |  |  |
| LOC_Os04g02690 | AT5G19610 |  |  |  |  |  |  |  |  |  |  |  |  |
| LOC_Os01g74480 | AT1G07750 | AT2G28680 |  |  |  |  |  |  |  |  |  |  |  |
| LOC_Os06g11970 | AT1G22130 | AT1G77980 |  |  |  |  |  |  |  |  |  |  |  |
| LOC_Os12g38260 | AT5G02390 |  |  |  |  |  |  |  |  |  |  |  |  |
| LOC_Os07g15680 | AT4G35790 |  |  |  |  |  |  |  |  |  |  |  |  |
| LOC_Os08g25710 | AT1G02730 | AT1G32180 | AT2G33100 | AT3G03050 | AT4G38190 | AT5G16910 |  |  |  |  |  |  |  |
| LOC_Os08g15090 | AT5G06540 | AT5G57690 |  |  |  |  |  |  |  |  |  |  |  |
| LOC_Os05g37880 | AT2G37980 | AT3G54100 |  |  |  |  |  |  |  |  |  |  |  |
| LOC_Os05g06670 | AT1G02400 |  |  |  |  |  |  |  |  |  |  |  |  |
| LOC_Os06g51460 | AT1G53390 | AT2G37010 | AT5G60740 |  |  |  |  |  |  |  |  |  |  |
| LOC_Os03g06760 | AT3G56640 |  |  |  |  |  |  |  |  |  |  |  |  |
| LOC_Os01g50470 | AT3G23270 | AT4G14368 |  |  |  |  |  |  |  |  |  |  |  |
| LOC_Os05g09440 | AT5G11670 | AT5G25880 |  |  |  |  |  |  |  |  |  |  |  |
| LOC_Os02g01590 | AT1G12240 | AT1G62660 |  |  |  |  |  |  |  |  |  |  |  |
| LOC_Os03g08110 | AT1G17430 | AT1G72620 |  |  |  |  |  |  |  |  |  |  |  |
| Locus number | Orthologue |  |  |  |  |  |  |  |  |  |  |  |  |
| LOC_Os02g52840 | AT5G08640 |  |  |  |  |  |  |  |  |  |  |  |  |
| LOC_Os02g58660 | AT1G16380 | AT1G79400 |  |  |  |  |  |  |  |  |  |  |  |
| LOC_Os12g07874 | AT5G02430 |  |  |  |  |  |  |  |  |  |  |  |  |
| LOC_Os05g19500 | AT3G17630 |  |  |  |  |  |  |  |  |  |  |  |  |
| LOC_Os03g62410 | AT4G35790 |  |  |  |  |  |  |  |  |  |  |  |  |
| LOC_Os06g29080 | AT1G23540 | AT1G70450 | AT1G70460 |  |  |  |  |  |  |  |  |  |  |
| LOC_Os05g35160 | AT1G03050 | AT4G02650 |  |  |  |  |  |  |  |  |  |  |  |
| LOC_Os01g60140 | AT2G13620 |  |  |  |  |  |  |  |  |  |  |  |  |
| LOC_Os02g57790 | AT3G10470 | AT5G04390 |  |  |  |  |  |  |  |  |  |  |  |
| LOC_Os11g06700 | AT5G13150 | AT5G13990 |  |  |  |  |  |  |  |  |  |  |  |
| LOC_Os12g37480 | AT3G36659 |  |  |  |  |  |  |  |  |  |  |  |  |
| LOC_Os06g45240 | AT4G31250 |  |  |  |  |  |  |  |  |  |  |  |  |
| LOC_Os01g42060 | AT5G16020 |  |  |  |  |  |  |  |  |  |  |  |  |
| LOC_Os06g03830 | AT4G24050 |  |  |  |  |  |  |  |  |  |  |  |  |
| LOC_Os07g30210 | AT4G16620 | AT5G47470 |  |  |  |  |  |  |  |  |  |  |  |
| LOC_Os12g16180 | AT2G39400 | AT2G39410 | AT2G39420 | AT3G55180 |  |  |  |  |  |  |  |  |  |
| LOC_Os07g20270 | AT2G03630 |  |  |  |  |  |  |  |  |  |  |  |  |
| LOC_Os04g54860 | AT3G28850 | AT5G39865 |  |  |  |  |  |  |  |  |  |  |  |
| LOC_Os06g39050 | AT1G11250 | AT1G61290 |  |  |  |  |  |  |  |  |  |  |  |
| LOC_Os05g50830 | AT1G64300 | AT5G41730 |  |  |  |  |  |  |  |  |  |  |  |
| LOC_Os04g40570 | AT3G28345 | AT3G28360 | AT3G28380 | AT3G28390 | AT3G28415 |  |  |  |  |  |  |  |  |
| LOC_Os06g09860 | AT1G72460 | AT3G42880 | AT5G20690 |  |  |  |  |  |  |  |  |  |  |
| LOC_Os03g09160 | AT2G22180 | AT4G39745 |  |  |  |  |  |  |  |  |  |  |  |
| LOC_Os07g06800 | AT5G16010 |  |  |  |  |  |  |  |  |  |  |  |  |
| LOC_Os11g43740 | AT1G18750 | AT2G03060 |  |  |  |  |  |  |  |  |  |  |  |
| LOC_Os12g30500 | AT3G11850 | AT5G06560 |  |  |  |  |  |  |  |  |  |  |  |
| Locus number | Orthologue |  |  |  |  |  |  |  |  |  |  |  |  |
| LOC_Os02g10990 | AT3G15620 |  |  |  |  |  |  |  |  |  |  |  |  |
| LOC_Os04g37520 | AT1G50630 | AT3G20300 |  |  |  |  |  |  |  |  |  |  |  |
| LOC_Os05g07880 | AT1G52570 | AT3G15730 |  |  |  |  |  |  |  |  |  |  |  |
| LOC_Os11g29780 | AT1G27930 | AT1G67330 |  |  |  |  |  |  |  |  |  |  |  |
| LOC_Os01g48540 | AT1G67290 | AT5G19580 |  |  |  |  |  |  |  |  |  |  |  |
| LOC_Os10g40140 | AT5G47180 |  |  |  |  |  |  |  |  |  |  |  |  |
| LOC_Os02g36924 | AT2G14210 | AT3G57230 |  |  |  |  |  |  |  |  |  |  |  |
| LOC_Os02g55400 | AT1G17260 | AT1G80660 | AT2G07560 | AT2G18960 | AT2G24520 | AT3G42640 | AT3G47950 | AT3G60330 | AT4G30190 | AT5G57350 | AT5G62670 |  |  |
| LOC_Os03g38390 | AT5G55050 |  |  |  |  |  |  |  |  |  |  |  |  |
| LOC_Os02g51600 | AT5G65090 |  |  |  |  |  |  |  |  |  |  |  |  |
| LOC_Os06g44900 | AT5G20590 |  |  |  |  |  |  |  |  |  |  |  |  |
| LOC_Os02g26320 | AT2G24450 | AT3G12660 | AT4G31370 |  |  |  |  |  |  |  |  |  |  |
| LOC_Os12g05730 | AT3G52720 |  |  |  |  |  |  |  |  |  |  |  |  |
| LOC_Os09g25650 | AT5G49150 |  |  |  |  |  |  |  |  |  |  |  |  |
| LOC_Os12g30150 | AT2G38910 |  |  |  |  |  |  |  |  |  |  |  |  |
| LOC_Os05g09050 | AT1G42560 | AT2G33670 |  |  |  |  |  |  |  |  |  |  |  |
| LOC_Os06g51130 | AT5G42340 |  |  |  |  |  |  |  |  |  |  |  |  |
| LOC_Os12g44300 | AT3G52080 |  |  |  |  |  |  |  |  |  |  |  |  |
| LOC_Os02g44080 | AT4G17340 | AT5G47450 |  |  |  |  |  |  |  |  |  |  |  |
| LOC_Os08g40990 | AT1G50610 | AT2G07040 | AT3G20190 | AT5G35390 |  |  |  |  |  |  |  |  |  |
| LOC_Os01g71780 | AT3G51930 |  |  |  |  |  |  |  |  |  |  |  |  |
| LOC_Os05g48640 | AT3G24620 | AT4G13240 |  |  |  |  |  |  |  |  |  |  |  |
| LOC_Os12g12730 | AT1G05990 | AT3G07490 |  |  |  |  |  |  |  |  |  |  |  |
| LOC_Os09g26360 | AT2G26450 | AT4G33230 |  |  |  |  |  |  |  |  |  |  |  |
| LOC_Os08g45230 | AT3G23350 |  |  |  |  |  |  |  |  |  |  |  |  |
| LOC_Os02g03550 | AT1G32170 |  |  |  |  |  |  |  |  |  |  |  |  |
| Locus number | Orthologue |  |  |  |  |  |  |  |  |  |  |  |  |
| LOC_Os02g57000 | AT1G04540 | AT2G33320 |  |  |  |  |  |  |  |  |  |  |  |
| LOC_Os06g08310 | AT1G17260 | AT1G80660 | AT2G07560 | AT2G18960 | AT2G24520 | AT3G42640 | AT3G47950 | AT3G60330 | AT4G30190 | AT5G57350 | AT5G62670 |  |  |
| LOC_Os08g31080 | AT3G13850 |  |  |  |  |  |  |  |  |  |  |  |  |
| LOC_Os03g04560 | AT1G04470 | AT2G33420 |  |  |  |  |  |  |  |  |  |  |  |
| LOC_Os07g47120 | AT5G18670 |  |  |  |  |  |  |  |  |  |  |  |  |
| LOC_Os10g33640 | AT1G03920 |  |  |  |  |  |  |  |  |  |  |  |  |
| LOC_Os03g12570 | AT1G69770 | AT1G80740 |  |  |  |  |  |  |  |  |  |  |  |
| LOC_Os02g12660 | AT5G12000 | AT5G26150 |  |  |  |  |  |  |  |  |  |  |  |
| LOC_Os07g08000 | AT1G54510 | AT3G04810 | AT5G28290 |  |  |  |  |  |  |  |  |  |  |
| LOC_Os07g45260 | AT3G06260 |  |  |  |  |  |  |  |  |  |  |  |  |
| LOC_Os03g02740 | AT2G42010 | AT4G00240 | AT4G11830 | AT4G11840 | AT4G11850 |  |  |  |  |  |  |  |  |
| LOC_Os01g59360 | AT5G12180 | AT5G19360 |  |  |  |  |  |  |  |  |  |  |  |
| LOC_Os01g16470 | AT2G40850 | AT3G56600 |  |  |  |  |  |  |  |  |  |  |  |
| LOC_Os06g03610 | AT2G21480 | AT4G39110 |  |  |  |  |  |  |  |  |  |  |  |
| LOC_Os03g44440 | AT2G23980 | AT4G30560 | AT5G57940 |  |  |  |  |  |  |  |  |  |  |
| LOC_Os06g22980 | AT1G02730 | AT1G32180 | AT2G33100 | AT3G03050 | AT4G38190 | AT5G16910 |  |  |  |  |  |  |  |
| LOC_Os02g12300 | AT1G14420 | AT2G02720 | AT3G01270 | AT5G15110 |  |  |  |  |  |  |  |  |  |
| LOC_Os04g58480 | AT4G27790 |  |  |  |  |  |  |  |  |  |  |  |  |
| LOC_Os05g40650 | AT2G13620 |  |  |  |  |  |  |  |  |  |  |  |  |
| LOC_Os02g44120 | AT1G02040 |  |  |  |  |  |  |  |  |  |  |  |  |
| LOC_Os05g14750 | AT1G16440 | AT1G79250 | AT3G12690 |  |  |  |  |  |  |  |  |  |  |
| LOC_Os03g57510 | AT2G38910 |  |  |  |  |  |  |  |  |  |  |  |  |
| LOC_Os06g47110 | AT3G20580 | AT4G27110 |  |  |  |  |  |  |  |  |  |  |  |
| LOC_Os05g51670 | AT4G10960 | AT4G23920 |  |  |  |  |  |  |  |  |  |  |  |
| LOC_Os07g01260 | AT3G19090 |  |  |  |  |  |  |  |  |  |  |  |  |
| LOC_Os08g43500 | AT4G34940 | AT5G66200 |  |  |  |  |  |  |  |  |  |  |  |
| Locus number | Orthologue |  |  |  |  |  |  |  |  |  |  |  |  |
| LOC_Os06g44160 | AT1G80920 |  |  |  |  |  |  |  |  |  |  |  |  |
| LOC_Os02g38170 | AT4G24700 |  |  |  |  |  |  |  |  |  |  |  |  |
| LOC_Os09g27040 | AT5G55490 |  |  |  |  |  |  |  |  |  |  |  |  |
| LOC_Os12g06570 | AT3G48010 | AT5G14870 |  |  |  |  |  |  |  |  |  |  |  |
| LOC_Os05g35050 | AT2G26975 | AT3G46900 | AT5G59030 |  |  |  |  |  |  |  |  |  |  |
| LOC_Os03g17150 | AT2G28710 | AT3G46070 | AT3G46080 | AT3G46090 | AT5G59820 |  |  |  |  |  |  |  |  |
| LOC_Os05g45810 | AT1G64480 |  |  |  |  |  |  |  |  |  |  |  |  |
| LOC_Os03g51990 | AT5G65890 |  |  |  |  |  |  |  |  |  |  |  |  |
| LOC_Os01g11350 | AT2G42380 | AT3G58120 |  |  |  |  |  |  |  |  |  |  |  |
| LOC_Os04g38560 | AT3G06830 | AT5G49180 |  |  |  |  |  |  |  |  |  |  |  |
| LOC_Os12g35710 | AT1G49490 | AT2G15880 | AT3G19020 | AT4G33970 |  |  |  |  |  |  |  |  |  |
| LOC_Os05g40180 | AT5G01920 |  |  |  |  |  |  |  |  |  |  |  |  |
| LOC_Os02g54590 | AT1G16760 | AT1G78940 | AT2G24370 | AT4G31230 |  |  |  |  |  |  |  |  |  |
| LOC_Os02g10530 | AT2G04220 | AT4G12690 | AT5G48270 |  |  |  |  |  |  |  |  |  |  |
| LOC_Os04g46490 | AT3G47440 |  |  |  |  |  |  |  |  |  |  |  |  |
| LOC_Os06g09230 | AT1G16760 | AT1G78940 | AT2G24370 | AT4G31230 |  |  |  |  |  |  |  |  |  |

**Table S10.** MapMan terms related to cell wall organization and modifications in rice and *Arabidopsis*.

|  | Rice |  |  |  | Arabidopsis |  |  |
| --- | --- | --- | --- | --- | --- | --- | --- |
| category | Bin.Code | Bin.Name | Locus_ID |  | Bin.Code | Bin.Name | Locus_ID |
| cell wall carbohydrate synthesis | 10.1.2 | cell wall.precursor synthesis.UGE | LOC_Os05g51670.1 |  | 10.2 | cell wall.cellulose synthesis | at4g07960 |
|  | 10.1.6 | cell wall.precursor synthesis.GAE | LOC_Os06g08810.1 |  | 10.2.1 | cell wall.cellulose synthesis.cellulose synthase | at2g33100 |
|  | 10.2.1 | cell wall.cellulose synthesis.cellulose synthase | LOC_Os08g25710.1 |  | 10.2.1 | cell wall.cellulose synthesis.cellulose synthase | at4g38190 |
|  | 10.2.1 | cell wall.cellulose synthesis.cellulose synthase | LOC_Os04g35020.1 |  | 10.2.2 | cell wall.cellulose synthesis.COBRA | at3g20580 |
|  | 10.2.1 | cell wall.cellulose synthesis.cellulose synthase | LOC_Os06g22980.1 |  | 10.2.2 | cell wall.cellulose synthesis.COBRA | at4g27110 |
|  | 10.2.2 | cell wall.cellulose synthesis.COBRA | LOC_Os06g47110.1 |  |  |  |  |
|  | 10.3 | cell wall.hemicellulose synthesis | LOC_Os06g10970.1 |  |  |  |  |
|  |  |  |  |  |  |  |  |
| cell wall protein synthesis | 10.5 | cell wall.cell wall proteins | LOC_Os01g69240.1 |  | 10.5.1.1 | cell wall.cell wall proteins.AGPs.AGP | at2g24450 |
|  | 10.5.1.1 | cell wall.cell wall proteins.AGPs.AGP | LOC_Os06g44660.1 |  | 10.5.1.1 | cell wall.cell wall proteins.AGPs.AGP | at3g01700 |
|  | 10.5.1.1 | cell wall.cell wall proteins.AGPs.AGP | LOC_Os02g26320.1 |  | 10.5.1.1 | cell wall.cell wall proteins.AGPs.AGP | at3g20865 |
|  | 10.5.3 | cell wall.cell wall proteins.LRR | LOC_Os12g35710.1 |  | 10.5.1.1 | cell wall.cell wall proteins.AGPs.AGP | at5g14380 |
|  | 10.5.3 | cell wall.cell wall proteins.LRR | LOC_Os01g25460.1 |  | 10.5.1.1 | cell wall.cell wall proteins.AGPs.AGP | at5g53250 |
|  |  |  |  |  | 10.5.3 | cell wall.cell wall proteins.LRR | at1g49490 |
|  |  |  |  |  | 10.5.3 | cell wall.cell wall proteins.LRR | at2g15880 |
|  |  |  |  |  | 10.5.3 | cell wall.cell wall proteins.LRR | at3g19020 |
|  |  |  |  |  | 10.5.3 | cell wall.cell wall proteins.LRR | at4g33970 |
|  |  |  |  |  |  |  |  |
| cell wall degradation | 10.6.2 | cell wall.degradation.mannan-xylose-arabinose-fucose | LOC_Os10g21110.1 |  | 10.6.1 | cell wall.degradation.cellulases and beta -1,4-glucanases | at1g19940 |
|  | 10.6.3 | cell wall.degradation.pectate lyases and polygalacturonases | LOC_Os01g33300.1 |  | 10.6.1 | cell wall.degradation.cellulases and beta -1,4-glucanases | at3g43860 |
|  | 10.6.3 | cell wall.degradation.pectate lyases and polygalacturonases | LOC_Os06g05209.1 |  | 10.6.1 | cell wall.degradation.cellulases and beta -1,4-glucanases | at3g62710 |
|  | 10.6.3 | cell wall.degradation.pectate lyases and polygalacturonases | LOC_Os02g10300.1 |  | 10.6.3 | cell wall.degradation.pectate lyases and polygalacturonases | at1g02790 |
|  | 10.6.3 | cell wall.degradation.pectate lyases and polygalacturonases | LOC_Os06g40890.1 |  | 10.6.3 | cell wall.degradation.pectate lyases and polygalacturonases | at1g14420 |
|  | 10.6.3 | cell wall.degradation.pectate lyases and polygalacturonases | LOC_Os06g05260.1 |  | 10.6.3 | cell wall.degradation.pectate lyases and polygalacturonases | at2g02720 |
|  | 10.6.3 | cell wall.degradation.pectate lyases and polygalacturonases | LOC_Os06g35320.1 |  | 10.6.3 | cell wall.degradation.pectate lyases and polygalacturonases | at2g23900 |
|  | 10.6.3 | cell wall.degradation.pectate lyases and polygalacturonases | LOC_Os02g12300.1 |  | 10.6.3 | cell wall.degradation.pectate lyases and polygalacturonases | at3g01270 |
|  | 10.6.3 | cell wall.degradation.pectate lyases and polygalacturonases | LOC_Os06g38510.1 |  | 10.6.3 | cell wall.degradation.pectate lyases and polygalacturonases | at3g07820 |
|  |  |  |  |  | 10.6.3 | cell wall.degradation.pectate lyases and polygalacturonases | at3g07830 |
|  |  |  |  |  | 10.6.3 | cell wall.degradation.pectate lyases and polygalacturonases | at3g07840 |
|  |  |  |  |  | 10.6.3 | cell wall.degradation.pectate lyases and polygalacturonases | at3g07850 |
|  |  |  |  |  | 10.6.3 | cell wall.degradation.pectate lyases and polygalacturonases | at5g15110 |
|  |  |  |  |  | 10.6.3 | cell wall.degradation.pectate lyases and polygalacturonases | at5g48140 |
|  |  |  |  |  |  |  |  |
| cell wall modification | 10.7 | cell wall.modification | LOC_Os04g25190.1 |  | 10.7 | cell wall.modification | at3g55500 |
|  | 10.7 | cell wall.modification | LOC_Os04g25160.1 |  | 10.7 | cell wall.modification | at3g60570 |
|  | 10.7 | cell wall.modification | LOC_Os03g01610.1 |  | 10.7 | cell wall.modification | at4g18990 |
|  | 10.7 | cell wall.modification | LOC_Os06g45190.1 |  | 10.7 | cell wall.modification | at5g39310 |
|  | 10.7 | cell wall.modification | LOC_Os02g03550.1 |  |  |  |  |
|  | 10.7 | cell wall.modification | LOC_Os06g45160.1 |  |  |  |  |
|  | 10.7 | cell wall.modification | LOC_Os06g45150.1 |  |  |  |  |
|  | 10.7 | cell wall.modification | LOC_Os08g44790.1 |  |  |  |  |
|  | 10.7 | cell wall.modification | LOC_Os06g45180.1 |  |  |  |  |
|  | 10.7 | cell wall.modification | LOC_Os08g13980.1 |  |  |  |  |
|  | 10.7 | cell wall.modification | LOC_Os12g36040.1 |  |  |  |  |
|  | 10.7 | cell wall.modification | LOC_Os03g01640.1 |  |  |  |  |
|  | 10.7 | cell wall.modification | LOC_Os06g44470.1 |  |  |  |  |
|  | 10.7 | cell wall.modification | LOC_Os10g40090.1 |  |  |  |  |
|  | 10.7 | cell wall.modification | LOC_Os04g25150.1 |  |  |  |  |
|  | 10.7 | cell wall.modification | LOC_Os06g45200.1 |  |  |  |  |
|  |  |  |  |  |  |  |  |
| petin esterase | 10.8.1 | cell wall.pectin*esterases.PME | LOC_Os03g18860.1 |  | 10.8.1 | cell wall.pectin*esterases.PME | at1g69940 |
|  | 10.8.1 | cell wall.pectin*esterases.PME | LOC_Os03g19610.1 |  | 10.8.1 | cell wall.pectin*esterases.PME | at2g26450 |
|  | 10.8.1 | cell wall.pectin*esterases.PME | LOC_Os04g38560.1 |  | 10.8.1 | cell wall.pectin*esterases.PME | at2g47040 |
|  | 10.8.1 | cell wall.pectin*esterases.PME | LOC_Os07g49100.1 |  | 10.8.1 | cell wall.pectin*esterases.PME | at2g47550 |
|  | 10.8.1 | cell wall.pectin*esterases.PME | LOC_Os09g26360.1 |  | 10.8.1 | cell wall.pectin*esterases.PME | at3g05610 |
|  | 10.8.1 | cell wall.pectin*esterases.PME | LOC_Os11g45720.1 |  | 10.8.1 | cell wall.pectin*esterases.PME | at3g06830 |
|  | 10.8.1 | cell wall.pectin*esterases.PME | LOC_Os11g45730.1 |  | 10.8.1 | cell wall.pectin*esterases.PME | at3g17060 |
|  | 10.8.1 | cell wall.pectin*esterases.PME | LOC_Os03g28090.1 |  | 10.8.1 | cell wall.pectin*esterases.PME | at3g62170 |
|  | 10.8.1 | cell wall.pectin*esterases.PME | LOC_Os12g37660.1 |  | 10.8.1 | cell wall.pectin*esterases.PME | at4g15980 |
|  | 10.8.1 | cell wall.pectin*esterases.PME | LOC_Os08g34900.1 |  | 10.8.1 | cell wall.pectin*esterases.PME | at4g33230 |
|  | 10.8.1 | cell wall.pectin*esterases.PME | LOC_Os04g54850.1 |  | 10.8.1 | cell wall.pectin*esterases.PME | at5g07420 |
|  |  |  |  |  | 10.8.1 | cell wall.pectin*esterases.PME | at5g07430 |
|  |  |  |  |  | 10.8.1 | cell wall.pectin*esterases.PME | at5g27870 |

| **Table S11.** Primer sequences used in genotyping and real-time PCR. | |
| --- | --- |
| Pimer name | Sequences |
| LOC_Os11g20384 RT F | CATCACATATCCTCAGTTAG |
| LOC_Os11g20384 RT R | TGGAAAACATCATTACGTGC |
| LOC_Os07g17310 RT F | ATGCAGCTGGTGCGAAACAT |
| LOC_Os07g17310 RT R | CATGCTGGGAGTACCTCTTC |
| LOC_Os11g45730 RT F | CACTACATCTACGGCGACTC |
| LOC_Os11g45730 RT R | ACGCAGTTGAGTAGCTAGTC |
| LOC_Os02g50770 RT F | GTGATTCGCTAAATCGATTG |
| LOC_Os02g50770 RT R | CAGTTCTCGTACGAGCAGAA |
| LOC_Os01g69020 RT F | GCTGGTTGATCCTTCACACG |
| LOC_Os01g69020 RT R | ATCTCCGAGATCACGAAGAA |
| LOC_Os05g46530 RT F | GGTGCCATCATCGACAATGG |
| LOC_Os05g46530 RT R | CTTCCTCAGCGTGTTGCACA |
| LOC_Os07g14340 RT F | GATTCCGAGGAGTACTGAGC |
| LOC_Os07g14340 RT R | TCTTCGCTAGTGTCAACGGA |
| LOC_Os04g25190 RT F | CTTTGTCCTCTTTCCGGCTC |
| LOC_Os04g25190 RT R | CCTCAGAGATGGCAACATTA |
| MTD1-1 F | AACGATGCGTGCTGCGTTCA |
| MTD1-1 R | GCCAATGATGTCTCCATGAG |
| MTD1 cas F | GGCAGATCTGCAATTGTCCAGTGA |
| MTD1 cas R | AAACTCACTGGACAATTGCAGATC |
